# Electron donor–acceptor complex offers a diverse approach for carbonyl alkylative amination

**DOI:** 10.1039/d5sc04087f

**Published:** 2025-09-24

**Authors:** Hrishikesh Paul, Arijit Chakraborty, Animesh Mandal, Dibyangshu Das, Sanat Kumar Mahapatra, Lisa Roy, Indranil Chatterjee

**Affiliations:** a Department of Chemistry, Indian Institute of Technology Ropar Nangal Road Rupnagar Punjab-140001 India indranil.chatterjee@iitrpr.ac.in; b Institute of Chemical Technology Mumbai-IOC Odisha Campus Bhubaneswar Bhubaneswar 751013 India; c Department of Education, Indian Institute of Technology Kharagpur Kharagpur 721302 India L.Roy@edu.iitkgp.ac.in

## Abstract

The synthesis of α-tertiary amino acids and amines is crucial in biochemistry and medicinal chemistry. However, creating tertiary carbon centers has traditionally been challenging due to the lack of effective, sustainable, and straightforward mild protocols. This current work presents a method for creating α-tertiary carbon centers that leverages the formation of an electron donor–acceptor (EDA) complex, where electron-poor imines act as the acceptor and electron-rich 1,4-dihydropyridine serves as the donor. This interaction facilitates the generation of α-amino radicals through a charge transfer phenomenon. In the presence of a suitable radical trapping reagent, these α-amino radicals can forge C–C bonds, where H-DHP acts solely as a reductant. Additionally, a radical cross-coupling process between an alkyl radical generated from 4-alkylated 1,4-dihydropyridine and the α-amino radical also produces reductive alkylation products. In this later scenario, 4-alkylated 1,4-dihydropyridine functions both as a reductant and as a source of alkyl radicals. Interestingly, both processes yield amino acids and amine derivatives having α-tertiary centres under mild reaction conditions, avoiding the need for photocatalysts or transition metals.

## Abstract 2

Visible-light-driven carbonyl alkylative amination has been reported here, utilizing 1,4-dihydropyridine as both a reductant and alkyl transfer reagent, efficiently syntheszing α-tertiary amino acids and amine derivatives.

## Graphical abstract

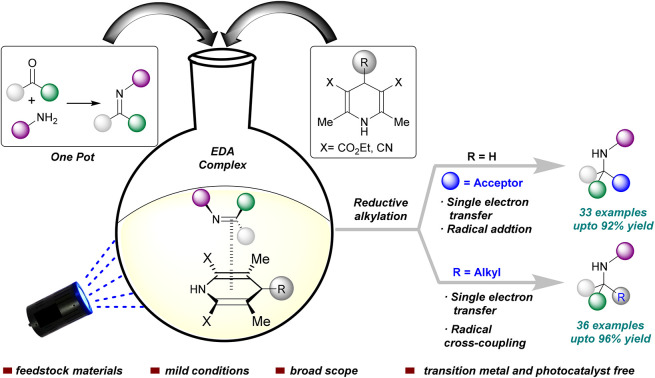

## Introduction

**Scheme 1:**
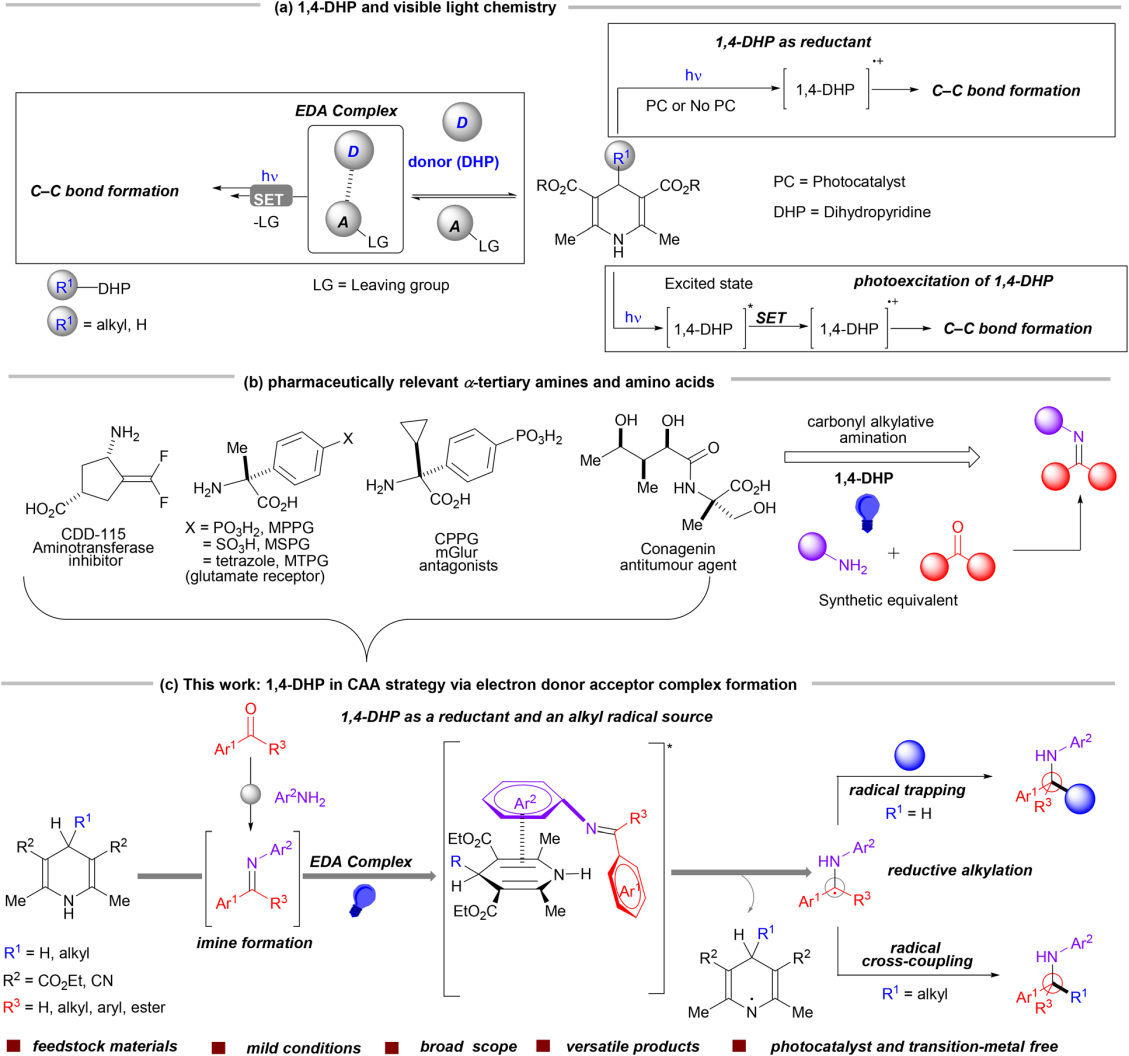
Visible light chemistry of 1,4-DHP and its application in the CAA strategy for the synthesis of α-tertiary amines and amino acids.

1,4-Dihydropyridine (H-DHP) derivatives have emerged as powerful reagents in modern organic synthesis, especially in visible-light-driven transformations.^1–4^ Their unique electronic and structural properties allow them to function as mild and efficient reductants, enabling selective reductions under mild reaction conditions without the need for harsh conditions. The capability of H-DHPs to act as photoreductants under visible-light activation has been explored significantly, with pioneering studies by researchers such as Tanaka, Cho, Dilman, Cheng and others.^5–10^ Beyond their role as conventional reducing agents, H-DHPs are also involved in electron transfer processes *via* the photoexcitation pathway. Inspired by the pioneering work of the Melchiorre group in 2017, our group developed, in 2023, a complementary strategy that also exploits the photoactive properties of alkyl-DHPs upon visible-light irradiation to facilitate the construction of diverse C–C bonds without using any external photocatalysts.^11–19^ In parallel, structurally modified alkyl dihydropyridines (alkyl-DHPs) have garnered attention due to their effectiveness in alkyl transfer reactions. The groups of Nishibayashi, Sakata, Molander and others have made significant advancements in utilizing alkyl-DHPs in efficient alkyl-transfer processes. Effective bond formation in those cases relies on the use of a photocatalyst or transition metal, where alkyl-DHPs serve as reductants.^20–30^ Moreover, their ability to form electron donor–acceptor (EDA) complexes with electronically deficient partners further enhances their synthetic utility.^31–36^ This multifaceted reactivity has established 1,4-dihydropyridine as an indispensable tool in modern photocatalysis and radical chemistry (Scheme 1a).

Imines are electrophilic in nature, so they can undergo reduction by accepting a single electron, thereby generating a highly reactive radical anion intermediate.^37–42^ These intermediates are key species in facilitating further transformations, making such imines valuable substrates in synthetic chemistry. While visible-light-driven transformations employing photocatalysts are well-established for generating these reactive intermediates, the photocatalyst-free activation of imines remains relatively unfamiliar. The current literature methods based on this type of activation involves either the use of a photocatalyst or highly reactive aldimine derivatives. A general strategy including broad carbonyl scope for the synthesis of α-tertiary amines and amino acid derivatives is still lacking. We envisioned forming an electron donor–acceptor (EDA) complex between electron-deficient imines (as an acceptor) and a suitable electron-rich donor, like H-DHP. This may trigger single-electron transfer (SET) from H-DHP to *in situ*-generated imines, providing an array of nucleophilic α-amino radicals. These radicals can then either combine with an electrophilic radical acceptor or engage in radical–radical cross-coupling to present an efficient and alternative carbonyl alkylative amination (CAA) process. This thought process may lead to a multi-component reaction (MCR), where amines, carbonyl compounds, and H-DHP can be leveraged to synthesize a diverse library of α-tertiary amines and amino acid derivatives, tailored to the nature of the carbonyl compounds. α-Tertiary amino acids and amines are essential in both chemistry and biology, with tertiary amines acting as vital intermediates in drug development and organic synthesis, while amino acids serve as the fundamental components of proteins, supporting numerous biochemical processes necessary for life (Scheme 1b).^43–48^

Traditional approaches to obtaining amino acids and amines typically involve the use of transition metal-catalyzed C–N bond formation,^49^ reductive amination of carbonyls,^50^ addition to imines, *N*-alkylation of amines,^51–54^ the aza-Morita–Baylis–Hillman reaction,^55^ and Mannich,^56^ Petasis,^57^ and Strecker reactions.^58^ Recently, the Gaunt group has developed effective strategies for accessing diverse amines and α-tertiary amino acid derivatives by adding nucleophilic alkyl radicals to *in situ*-generated electrophilic iminium ions.^59–70^

Herein, in this current strategy, we have utilized H-DHP as a reductant to facilitate the reactivity between α-amino radicals and radical trapping reagents. In addition, 4-alkyl-DHPs also serve a dual purpose by acting as reductants and alkyl transfer reagents (Scheme 1c). This protocol may bring a versatile solution for achieving comprehensive structural diversity in complex amine and α-tertiary amino acid synthesis. Modulated amines, carbonyls, radical acceptors, or 4-alkyl-DHP derivatives can offer a flexible approach to holistic molecular design.

## Results and discussion

To validate our hypothesis, we initiated exploratory reactions between the *in situ* generated imine 1a′ and alkene 3a, incorporating a base and H-DHP as a reductant. Our choice of the electron-deficient alkene 3a was intentional, as it allows us to investigate the concurrent cleavage of the C–F bond following the radical addition to 3a. This strategic approach may provide access to *gem*-difluoroolefin-containing α-tertiary amino acid derivatives 4.^71–78^ Interestingly, *gem*-difluoroolefins due to their metabolic stability are recognized as metabolically stable isosters for carbonyl groups. Successfully navigating the radical cross-coupling in tandem with C–F bond cleavage is critical, highlighting the complexity and significance in the case of alkene 3a. Nevertheless, due to the unique nature of *gem*-difluoroolefin it has been explored profoundly in drug discovery, medicinal chemistry and pharmaceuticals. For this reason, various bases, including potassium carbonate (K_2_CO_3_) and lithium carbonate (Li_2_CO_3_), were screened in combination with polar aprotic solvents such as acetonitrile (ACN) and dimethyl sulfoxide (DMSO). These conditions led to the formation of the desired product 4a in yields ranging from 25% to 70% (see Table 1, entries 2–6). Interestingly, we observed a significant drop in yield when a polar protic solvent such as ethanol (EtOH) was employed, with the desired product 4a formed only in trace amounts (Table 1, entry 7). This finding suggests that the choice of solvent plays a crucial role in the reaction efficiency. Further optimization of reaction parameters revealed that using H-DHP as the reductant (1.0 equiv.) and cesium carbonate (1.5 equiv.) as the base, with a 3-fold excess of alkene 3a in acetone at a 0.1 M concentration, provided the best conditions. Conducting the reaction under irradiation with 455 nm blue LEDs yielded the target product 4a in 94% yield, as measured by ^19^F NMR, and in 89% yield after isolation. Notably, the optimized conditions required an inert atmosphere and the concurrent presence of a base and light source to achieve high yields (Table 1, entries 8–10). However, other reductants such as DABCO, DIPEA, and NEt_3_ did not yield the desired product (Table 1, entries 11–13), whereas various light sources, including 370 nm, 390 nm, and 427 nm, produced the desired product with yields of up to 45% (Table 1, entries 14–16). A reduced amount of H-DHP (0.5 equiv.) still furnished the desired product 4a with a satisfactory yield of 67% (Table 1, entry 17). However, further exploration of H-DHP as a catalytic reductant still remained unsuccessful.

**Table 1:** Optimization study and reaction set-up^a^

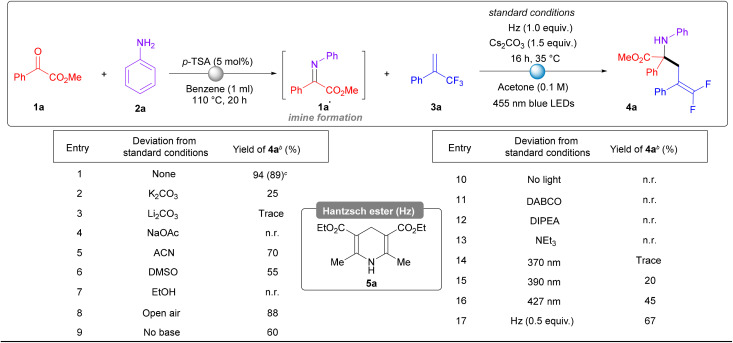

a Unless otherwise mentioned, all reactions were carried out on a 0.1 mmol scale under 455 nm blue LED irradiation using 1.0 equiv. of 1a, 1.2 equiv. of 2a and 3.0 equiv. of 4a.

b NMR yield using fluorobenzene as an internal standard.

c In brackets, yield refers to the isolated yield.

**Scheme 2:**
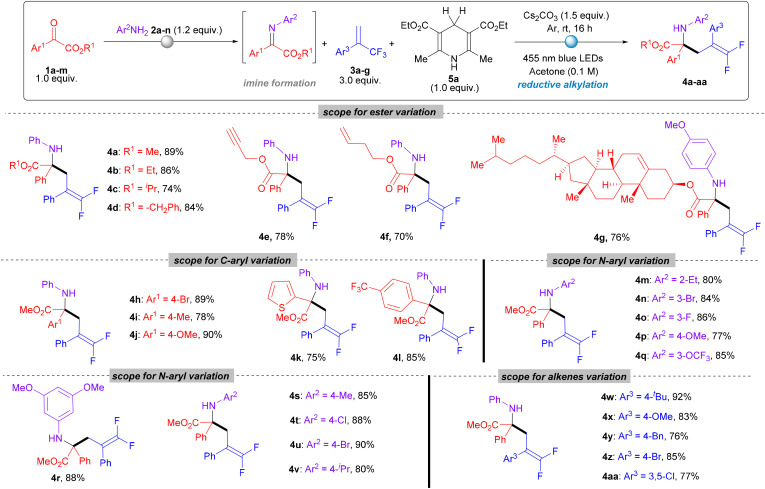
Substrate scope for radical addition^*a*^. ^*a*^All reactions were carried out on a 0.2 mmol scale under 455 nm LED irradiation using 1.0 equiv. of 1, 1.2 equiv. of 2, 3.0 equiv. of 3, and 1.0 equiv. of H-DHP. Yields refer to isolated yield for 4a–4aa.

To evaluate substrate generality, we explored variations in the ester group of the imines, including methyl, ethyl, isopropyl, benzyl, propargyl, and homoallyl esters. Natural product cholesterol, tagged with the ester part, also worked well. These substrates efficiently yielded the desired products (Scheme 2, entries 4a–4g) with yields ranging from 74% to 89%. We next examined the impact of substituents on the aryl ring at the C-position of the imines. Substituents such as bromo, methyl, methoxy and CF_3_ at the *para*-position of the aryl ring and thiophene instead of the phenyl ring were well-tolerated, delivering the corresponding products (4h–4l) in excellent yields of 75% to 90%. Further investigation focused on the *N*-aryl substituent of the imines. A variety of *N*-aryl groups were evaluated, producing the desired products (4m–4v) in yields ranging from 77% to 90%. Various alkene derivatives were assessed under the optimized conditions, with substituents such as *para-tert*-butyl, benzyl, methoxy, bromo, and 3,5-dichloro affording the desired products (4w–4aa) in yields ranging from 76% to 92%, thereby highlighting the versatility of the methodology.

The successful implementation of H-DHP as a reductant for synthesizing α-tertiary amino acid derivatives encouraged us to explore the direct alkylation of imines using 4-substituted 1,4-DHP. Here, we also choose the same *in situ* generated imine (1a′) derived from α-ketoester (1a) as the model substrate and 4-isopropyl 1,4-DHP (5a) as the secondary alkyl radical precursor for testing the hypothesis. To our delight, we were successfully able to obtain the desired radical coupling product (6a) (which is also a precursor of α-tertiary amino acid) in 86% isolated yield using Cs_2_CO_3_ as a base, acetone as solvent under an argon atmosphere and 390 nm irradiation. A similar optimization study, like that in Table 1, was also performed for this reaction (for a detailed optimization table, see Section 3.1B, SI).

**Scheme 3:**
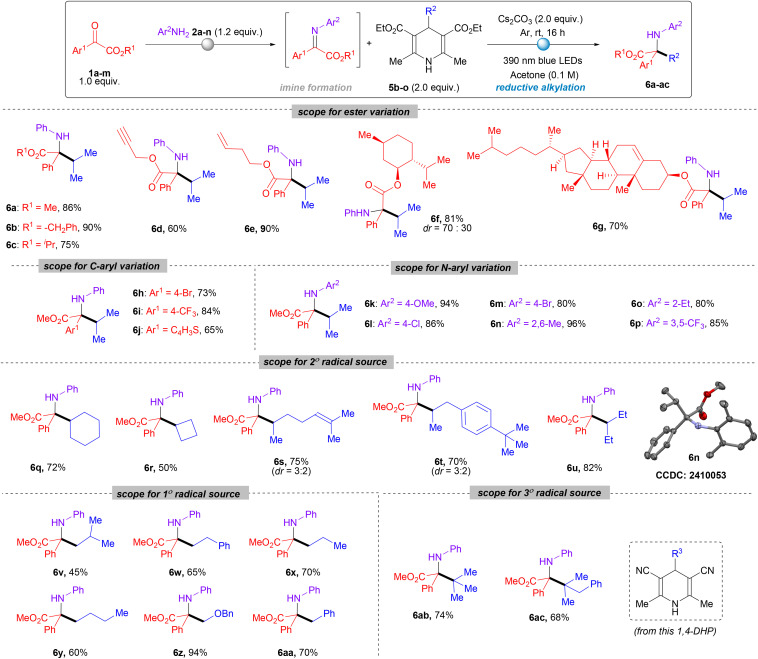
Substrate scope for radical–radical cross-coupling. All reactions were carried out on a 0.2 mmol scale under 390 nm LED irradiation using 1.0 equiv. of 1, 1.2 equiv. of 2, and 2.0 equiv. of 5. Yields refer to isolated yield for 6a–6ac. dr refers to the diastereomeric ratio of the isolated product.

Considering entry 5 (Table S5, SI) as the best reaction conditions, we investigated the viability of this alkylation reaction. At first, we varied the ester groups of α-ketoesters, including methyl, isopropyl, benzyl, propargyl and homoallyl esters. These substrates produced the desired alkylated products (Scheme 3, entries 6a–6e) with yields ranging from 60% to 90%. Esters derived from natural products menthol and cholesterol were also found to be suitable to produce reductive alkylation products 6f and 6g in 81% and 70% yields respectively. Next, we examined the effect of substituents in the aryl ring of the carbonyl and aniline. For *C*-aryl variation, 4-bromo, 4-CF_3_ at the phenyl ring and a heterocycle thiophene at the carbonyl carbon worked well furnishing the desired products 6h–6j in good yields. Anilines bearing substituents at different positions, for example halogens, electron-donating OMe, electron-withdrawing CF_3_, and alkyl groups, were well tolerated to provide desired products 6k–6p in good to excellent yields of 65% to 96%. The structure of 6n was further confirmed by single crystal XRD study (CCDC no.: 2410053).

After the successful variation of iminoesters, our main focus was to validate the scope of different types of radical precursors derived from various 4-alkyl-1,4-dihydropyridines under the standard reaction conditions. Apart from the isopropyl radical, the 2° cyclic radical *i.e.* the cyclohexyl radical was transferred successfully from 5b to deliver the desired product 6q in 72% yield. As we know bond strength increases upon reducing the bond angle which is clearly reflected in the low yield of 6r (50%) where a cyclobutyl radical precursor was used. 4-Alkyl-DHPs derived from a long chain aldehyde and lilial (both are secondary radical sources) worked efficiently to deliver the products (6s, 75% and 6t, 70%) albeit with low diastereoselectivity (3 : 2). 1,4-DHP derived from secondary alkyl radical precursor 5g produced 6u in 82% yield. Interestingly, 1° unactivated radicals derived from 4-alkyl-1,4-DHPs also worked well under current reaction conditions. In this regard, we were able to acquire the alkylated products using *n*-propyl, *n*-butyl, isobutyl, and β-phenyl ethyl radical precursors (6v–6y) in 45% to 70% yields. The primary radical adjacent to oxygen was found to be effective hence producing 6z in 94% owing to the stability of the radical. Apart from non-benzylic radicals, the benzylic primary radical also worked well to afford the product 6aa in 70% isolated yield. Another interesting finding was the successful transfer of a *tert*-butyl group and another customized tertiary radical to the imine providing the products 6ab (74%) and 6ac (68%) respectively in satisfactory yields. For this, we had to rely on a cyano analogue of 4-alkyl-1,4-DHP.

**Scheme 4:**
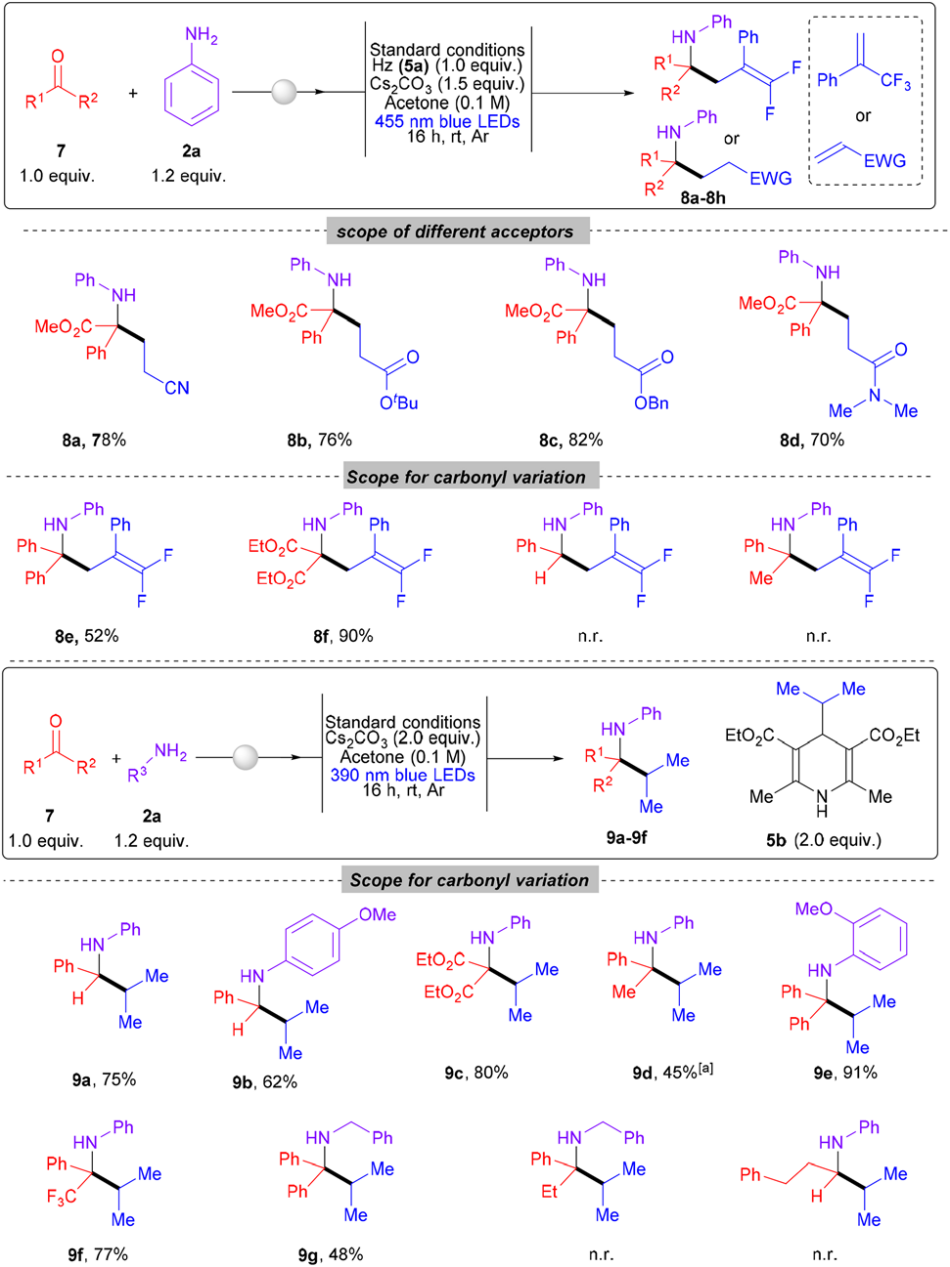
Substrate scope for further generalization. All reactions were carried out on a 0.2 mmol scale under 455 and 390 nm LED irradiation using 1.0 equiv. of 7, 1.2 equiv. of 2a. Yields refer to isolated yield for 8a–8f and 9a–9g. ^*a*^3.0 equiv. of 5b used.

Encouraged by the successful application of α-ketoesters in the carbonyl alkylative amination (CAA) for the synthesis of α-tertiary amino acid derivatives, we sought to further explore the versatility of this methodology. To demonstrate its applicability, at first, we investigated alternative radical acceptors, such as acrylonitrile, *tert*-butyl acrylate, benzyl acrylate, and *N*,*N*-dimethyl acrylate. Gratifyingly, all these substrates delivered the corresponding products (8a, 8b, 8c, and 8d) in good yields of 78%, 76%, 82%, and 70%, respectively. During our exploration, we assessed the reactivity of various classes of carbonyl compounds in the reactions we developed. Benzophenone and diester carbonyl derived *in situ* generated imines actively participated in the reductive alkylation process with alkene 3, leading to the formation of products 8e and 8f with yields ranging from moderate to good (Scheme 4). However, carbonyl compounds such as benzaldehyde and acetophenone remained unreactive, possibly due to the nature of the generated α-amino radicals. In the presence of an excellent HAT reagent, *i.e.*, oxidized H-DHP, these radicals may be quenched rapidly prior to the addition to the acceptor, preventing the formation of the desired product. In both the above cases, imine reduction was the major product, indicating a fast HAT process. Interestingly, the radical–radical coupling using 4-alkyl-1,4-DHP worked smoothly over a quite broad range of carbonyls producing the desired products, 9a–9g, with yields ranging from 45% to 91%. Imines derived from benzaldehyde and diester worked well to furnish products 9a, 9b and 9c with high yield. Interestingly, a higher equivalent of 5b was necessary for the formation of products 9d, albeit in moderate yields. Imines originated from benzophenone produced excellent outcome of product 9e. Additionally, imines produced from 2,2,2-trifluoroacetophenone and aniline also successfully alkylated to deliver 9f in 77% yield. The dependency upon anilines as the sole amine partner during the reaction pathway was also overcome smoothly. The imine derived from benzylamine, an aliphatic amine and benzophenone produced the desired *N*-alkyl α-tertiary amine product (9g) with a yield of 48%. This methodology has proven effective across a wide range of carbonyl compounds, allowing access to various amines, many of which feature an α-tertiary carbon center. Interestingly, carbonyls derived from aliphatic aldehydes or ketones remained unreactive, likely due to the instability of corresponding α-amino radicals.

**Scheme 5:**
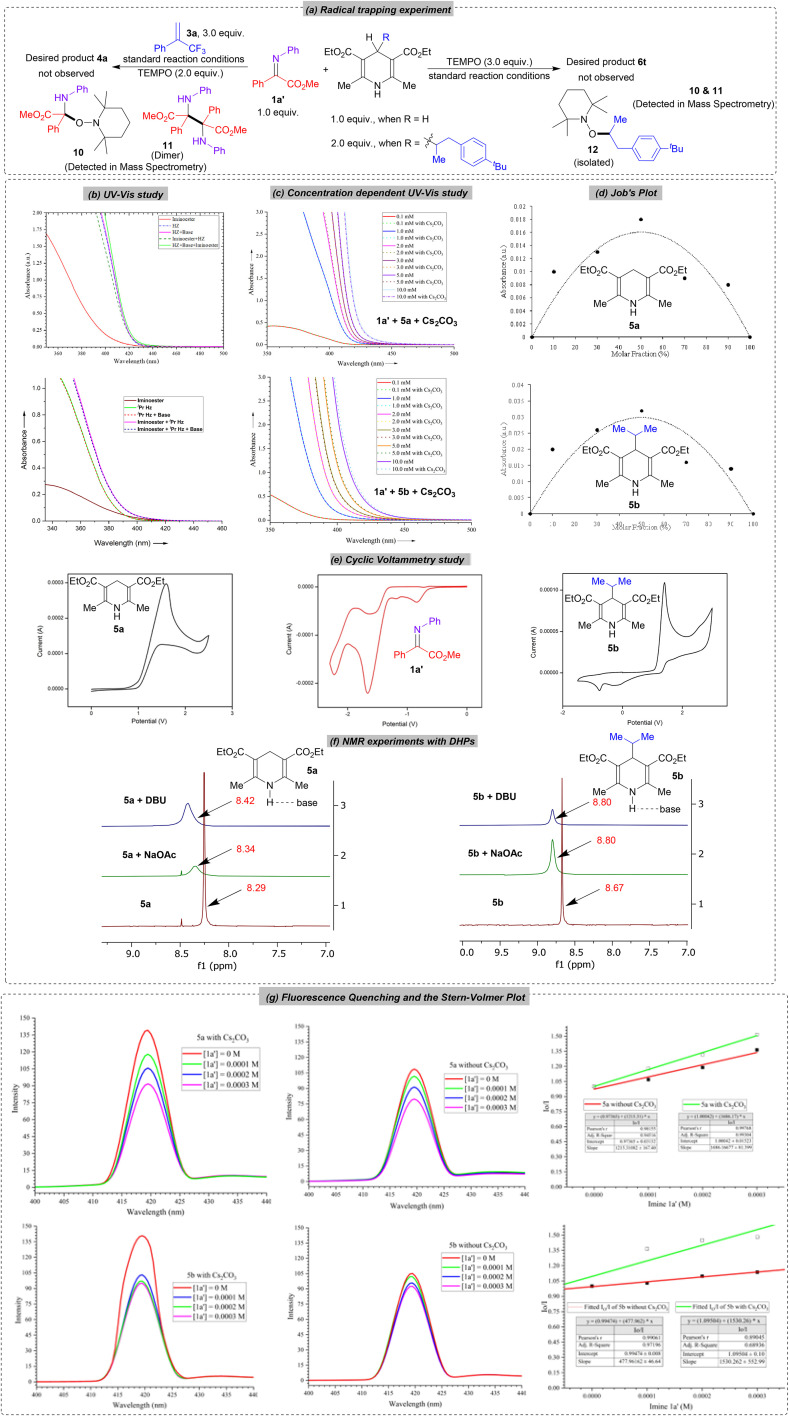
Mechanistic studies.

To have a deep insight into the mechanism of reductive alkylation, we performed several control experiments and mechanistic studies (for details, see the SI). The impact of light and base is already illustrated in the optimization studies (Table 1, entries 9–10 and Tables S4 and S8). Conducting the reaction under an air atmosphere resulted in a reduced yield with the reduction of the *in situ* generated imine, demonstrating the necessity of a fully inert atmosphere. To check whether the reaction proceeds *via* the radical pathway, we performed both reactions in the presence of a radical scavenger, TEMPO, under standard reaction conditions (Scheme 5a). Stoichiometric use of TEMPO completely quenched both the reactions and the desired product 4a or 6t was not observed. To our delight, the TEMPO adduct 10 from the imine was detected for both reactions in mass spectrometry and adduct 11 from the 1,4-DHP was isolated in 72% yield and fully characterized. This confirms the involvement of a radical intermediate in the reaction. Alongside, we also detected a dimer from the imine part for both protocols in mass spectrometry, which further confirms the single-electron reduction of imine to generate α-amino radicals. Additionally, we performed a series of controlled experiments involving 1,4-dihydropyridine (1,4-DHP) to explore whether the transformation proceeds *via* direct photoexcitation or through electron donor–acceptor (EDA) complex formation between the electron-rich 1,4-DHP and electron-deficient imines. Initially, UV-visible spectroscopic analysis revealed a notable bathochromic shift upon mixing imine, Hantzsch ester (5a or 5b), and base, indicating the formation of an electron donor–acceptor (EDA) complex (Scheme 5b). A gradual increase in the concentration of this ternary mixture led to a more pronounced red shift, with the absorption band tailing up to 510 nm. This clearly demonstrates that the formation of the EDA complex is strongly concentration-dependent (Scheme 5c).^34^ To understand the stoichiometry of this donor–acceptor interaction, Job's plot analysis was conducted through UV-visible titration, confirming the existence of an EDA complex between the Hantzsch ester, imine and base in a 1 : 1 : 1 ratio (Scheme 5d). To evaluate the redox compatibility of the involved species, cyclic voltammetry (CV) measurements were performed (Scheme 5e). Calculating the excited-state reduction potential of the Hantzsch ester *via* the Rehm–Weller equation (details available in the SI)^11,79^ demonstrates the viability of a photoinduced single-electron transfer (SET) process. To understand the essential role of the base in facilitating the reactions, ^1^H NMR studies were carried out in the presence of a base, revealing specific interactions with the N–H proton of the Hantzsch ester (Scheme 5f). These interactions likely alter the electronic structure of the Hantzsch ester, raising its HOMO energy and thereby accelerating the electron transfer. Additionally, fluorescence quenching experiments were undertaken to study the photophysical interactions (Scheme 5g). Stern–Volmer analysis showed that quenching of the Hantzsch ester fluorescence is more pronounced in the presence of base, aligning with its function as an enhanced photo-reductant under these conditions. Together, these findings indicate the formation of a well-defined EDA complex and support a reaction mechanism dominated by an EDA-mediated photochemical pathway, though a direct photoexcitation mechanism cannot be entirely ruled out.^80^ To further elucidate the mechanism, Density Functional Theory (DFT) calculations were carried out, providing complementary theoretical evidence supporting the proposed EDA pathway.

**Fig. 1:**
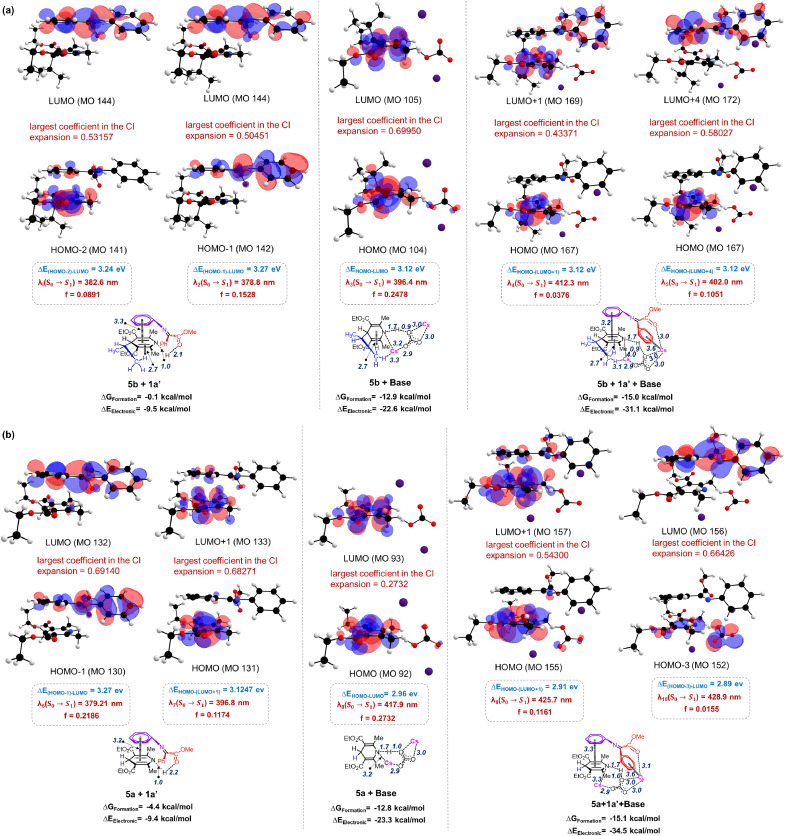
Frontier molecular orbitals with structures (vertical figures respectively) of (a) 5b with imine, 5b with Cs_2_CO_3_ base and a self-assembled EDA complex with 5b, imine and base and (b) 5a with imine, 5a with Cs_2_CO_3_ base and a self-assembled EDA complex with 5a, imine and base respectively. Excitations with two largest *f* values, corresponding to highest intensities, are shown. The distances are shown in Å.

**Scheme 6:**
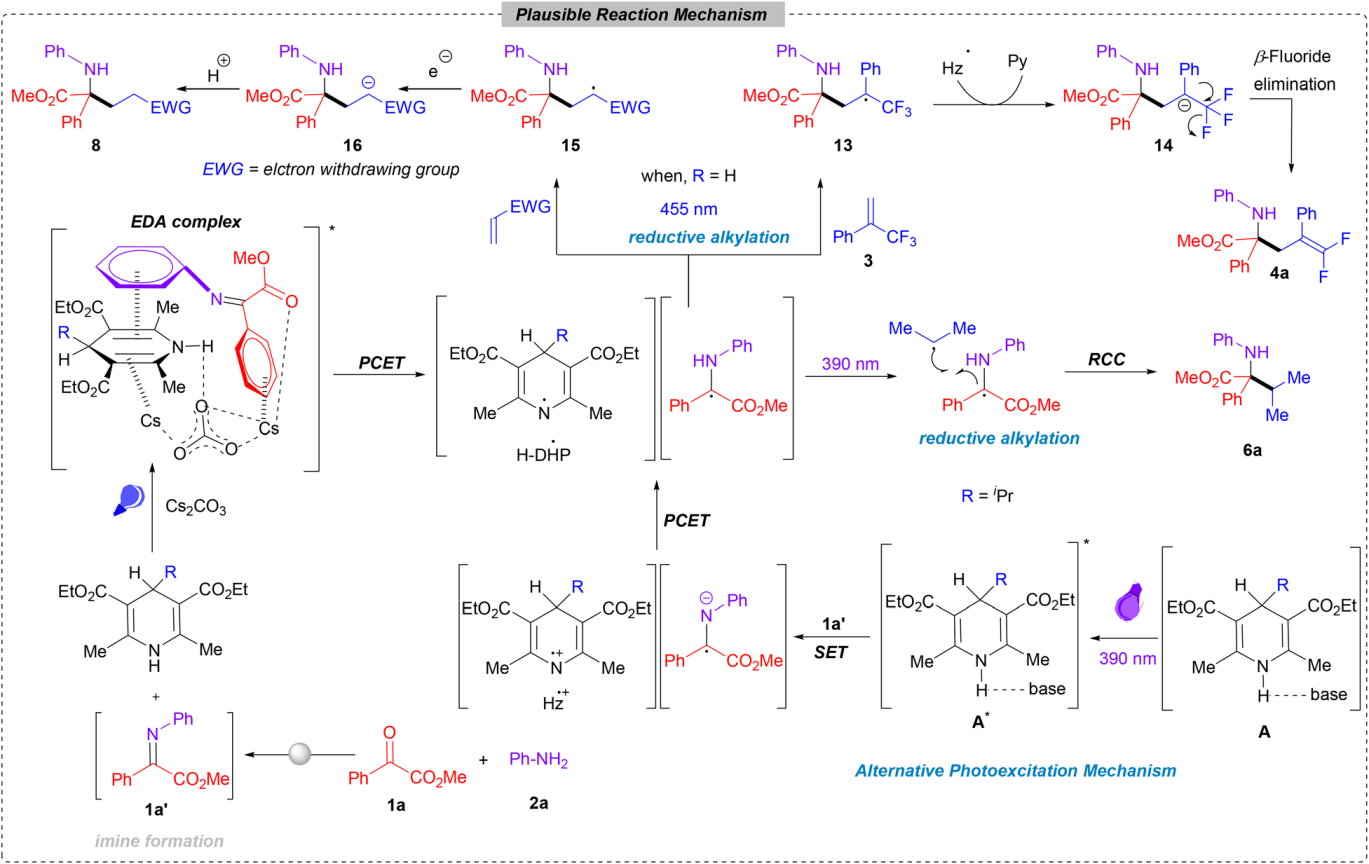
Plausible mechanism.

Density functional theory (DFT) calculations were conducted at M06-2X/SMD(acetone)/6-311+G(d, p) (for rest) and LANL2DZ (for Cs) to understand the nature of the EDA complex and reaction ensuing from it (see the SI for computational details). Our study reveals that the EDA complexation between the electron-rich 1,4-dihydropyridine or Hantzsch ester (5a or 5b) and the electron-deficient imine (1a′) is thermodynamically much favourable (Δ*G* = −15 kcal mol^−1^) in the presence of the base (Cs_2_CO_3_) (Fig. 1). This is primarily because of the various augmented non-covalent interactions like cation–π and H-bond interactions offered by the base that stabilizes the self-assembled EDA structure (see SI, Fig. S29 and S30). This is further supported by excitation energies and predicted wavelengths with the TDDFT formalism at M06L/SMD/6-311+G(d, p) (for rest) and LANL2DZ (for Cs). As observed from frontier molecular orbital pictures in Fig. 1a, *λ*_1_(S_0_ → S_1_) for intermolecular charge-transfer between 5b and 1a′ is predicted at 382.6 nm with oscillator strength *f* = 0.0891 which shows a bathochromic shift in the presence of the base with predicted *λ*_5_(S_0_ → S_1_) = 402.0 nm and a slight increment in absorbance with *f* = 0.1051. Moreover, the role of the base for EDA complexation between 5a and 1a′ is evident from its prominent contribution in the donor orbital (Fig. 1b) and results in significant bathochromic shift for *λ*_10_(S_0_ → S_1_) at 428.9 nm as compared to the pristine EDA complex between 5a and 1a′ at *λ*_7_(S_0_ → S_1_) = 378.8 nm, albeit with a lowered *f* value for the former (0.0155) as compared to the latter (0.1174), perhaps due to the enhanced spin–orbit coupling effect in the presence of heavy elements like Cs. Overall, our analysis emphasizes the favourable role of the base in stabilizing the self-assembled structure of the EDA complex that triggers bathochromic shift of intermolecular charge-transfer excitations to facilitate single electron transfer (SET). Given the fact that 1 : 1 : 1 interaction between the Hantzsch ester, imine and base is indicated in the Job's plot (Scheme 5d), the base-assisted self-assembled EDA complex therefore is a catalytic entity that triggers intermolecular charge transfer at an observed absorption wavelength of *λ*_max_ = 402.0 nm to transfer an electron from the available nitrogen lone pair of 5b to the vacant π* orbital of C=N at 1a′. This leads to concerted H-atom transfer to the electron-rich N-atom of 1a′. In total, proton-coupled electron transfer (PCET) is predicted. A gradual decrease in the gap between the donor–acceptor frontier orbitals is facilitated by the involvement of a gradual increase in NCIs (shown in RDG scatter plots in SI Fig. S29). Moreover, the entropic effect also contributes to greater stability of the intermediate formed after PCET from the base-assisted EDA complex.^81,82^ In the case of the Giese-type reaction, the highest ^1^H NMR yield (94%) was obtained under 455 nm light irradiation, which closely aligns with the calculated absorption maximum (428.9 nm) of the trimeric complex formed between the imine, Hantzsch ester (5a), and base. This suggests that the reaction is primarily driven by the EDA complex pathway. Conversely, for the alkylation reaction, the maximum ^1^H NMR yield (91%) was observed with 390 nm light, though comparable yields were also obtained with 455 nm light (75%). The calculated absorption wavelength of the trimeric complex involving the imine, Hantzsch ester (5b), and base was 402 nm. Additionally, the Hantzsch esters in acetone solution exhibited absorption at wavelengths greater than 390 nm, indicating their ability to undergo selective photoexcitation at this wavelength.^34^ In the excited state, these Hantzsch esters act as potent single-electron donors, as evidenced by their nearly complete conversion to pyridine under optimized conditions for alkylation reactions (relative to 2.0 equiv. of 5b). In fact, TDDFT calculations of the photoexcitation of 5b predicted it at 363.6 nm, which is enhanced to 396.4 nm in the presence of the base (Fig. 1b, middle column), in agreement with the alternative base-mediated SET pathway as shown in Scheme 6. Taken together, these observations suggest that the Giese-type reaction predominantly proceeds *via* the EDA complex pathway, although the contribution of direct photoexcitation cannot be ruled out. In contrast, the alkylation reaction appears to proceed through a dual pathway involving both EDA complex formation and direct photoexcitation of the Hantzsch ester.

Based on our mechanistic investigations and literature precedence, we proposed a plausible mechanism for the CAA reaction in Scheme 6. The condensation of ketoester 1a with aniline 2a initially generates imine 1a′ in the reaction medium. Upon addition of H-DHP and photo-irradiation, electron donor–acceptor (EDA) complex formation takes place, from where the subsequent proton-coupled electron transfer (PCET) generates oxidized H-DHP and α-amino radicals. Divergent reductive alkylation occurs through two main pathways. In the first pathway, when H-DHP is used solely as a reductant, the generated carbon-centred radical adds to the alkene 3 to form the radical intermediate 13. This intermediate then undergoes single electron reduction from oxidized H-DHP, resulting in the formation of the carbanion (14). Successive β-fluoride elimination from 14 yields the desired *gem*-difluorinated product (4a) along with Hantzsch pyridine as a by-product. Apart from this, the generated α-amino radical can also be trapped using different Michael acceptors to form alkyl radical intermediates (15). Subsequent electron transfer from the oxidized H-DHP generates a carbanionic intermediate (16), which, upon protonation, furnishes the desired product 8. However, when 4-alkyl-1,4-dihydropyridine (DHP) is used instead of H-DHP (5a), it generates the alkyl radical after the formation of the EDA complex *via* proton-coupled electron transfer (PCET). This newly formed alkyl radical, in concert with the carbon-centred radical derived from the reduced imine, engages in a radical cross-coupling (RCC) reaction. This process leads to the formation of a C–C bond, ultimately resulting in the expected α-tertiary amino acid precursor product 6a. An alternative mechanism is also proposed, where the combination of 4-alkyl-1,4-dihydropyridine and Cs_2_CO_3_ acts as a photoactive species A. This species upon irradiation with 390 nm LEDs forms an excited complex (A*) that operates as a potent reducing agent. This excited state complex A* donates an electron to the imine 1a′, initiating proton-coupled electron transfer (PCET) that produces both the α-amino radical and a carbon-centered radical. These radicals subsequently undergo a radical cross-coupling (RCC) process, leading to the formation of the alkylated product 6a.

## Conclusions

To summarize, this work reported a carbonyl alkylative amination (CAA) method employing readily accessible feedstock materials. This strategy offers a diverse protocol to access various α-tertiary amino acids and amines utilizing mild and operationally simple reaction conditions by modulating the amines, carbonyls and either radical trapping reagents or 4-alkyl-DHPs. The key to success relies on forming an EDA complex between electron-poor imines as acceptors and electron-rich H-DHP or alkyl-DHPs as donors to initiate the SET process, producing α-amino radicals. Interestingly, this methodology provides an alternative CAA approach that does not require any photoredox catalysts or transition metal, unlocking the potential of visible light beyond the traditional reactivity to generate molecular complexity for creating amine-containing molecules.

## Author contributions

The manuscript was written through the contributions of all authors. All authors have given approval to the final version of the manuscript.

## Conflicts of interest

There are no conflicts to declare.

## Supplementary Material

SC-OLF-D5SC04087F-s001

SC-OLF-D5SC04087F-s002

## Data Availability

All primary data are available in the supplementary information (SI). Supplementary information is available. See DOI: https://doi.org/10.1039/d5sc04087f. CCDC 2410053 contains the supplementary crystallographic data for this paper.^83^
