# Supplementary material for: Electron donor–acceptor complex offers a diverse approach for carbonyl alkylative amination
Source: Chem Sci. 2025 Sep 24;16(42):20061–72. doi: 10.1039/d5sc04087f (PMC12489803; doi:10.1039/d5sc04087f)
Supplement: SC-016-D5SC04087F-s001 [file SC-016-D5SC04087F-s001.pdf]

Supplementary Information (SI) for Chemical Science.
This journal is © The Royal Society of Chemistry 2025

Electron Donor-Acceptor Complex Offers a Diverse Approach for Carbonyl Alkylative Amination

Hrishikesh Paul,[a] # Arijit Chakraborty,[a] # Animesh Mandal,[a] § Dibyangshu Das,[a] § Sanat Kumar Mahapatra,[b] § Lisa Roy*[c] and Indranil Chatterjee*[a]

Department of Chemistry, Indian Institute of Technology Ropar

Ropar, Punjab-140001, India

Indranil.chatterjee@iitrpr.ac.in

Supporting Information

Table of Contents

**1.0**	**General Information**	**S1**
**2.0**	**List of starting materials**	**S2**
**3.0**	**Optimization Table (Tables S1–S9)**	**S4**
**4.0**	**General Procedure**	**S10**
4.1	General Procedure 1: Preparation of ketoesters (GP-1)	**S10**
4.2	General Procedure 2: Preparation of 1,4-dihydropyridines (GP-2)	**S11**
4.3	General Procedure 3: Preparation of trifluoromethyl styrenes (GP-3)	**S12**
4.4	General Procedure 4: Photoinduced carbonyl alkylative amination using radical trapper(GP-4)	**S13**
4.5	General Procedure 5: Photoinduced carbonyl alkylative amination using 4-alkyl-1,4-DHP (GP-5)	**S13**
**5.0**	**Mechanistic studies**	**S14**
5.1	Radical Trapping Experiment	**S14**
5.2	UV-Visible Experiment	**S17**
5.3	Job's Plot	**S21**
5.4	Cyclic Voltammetry Study	**S23**
5.5	NMR Experiments with DHPs	**S24**
5.6	Fluorescence Quenching Experiments (Stern-Volmer Study)	**S25**
5.7	Measurement of Stokes Shift for DHPs	**S30**
**6.0**	**Experimental Details for the Substrate Scope**	**S31**
6.1	Methyl 5,5-difluoro-2,4-diphenyl-2-(phenylamino)pent-4-enoate **(4a)**	**S31**
6.2	Ethyl 5,5-difluoro-2,4-diphenyl-2-(phenylamino)pent-4-enoate **(4b)**	**S31**
6.3	Isopropyl 5,5-difluoro-2,4-diphenyl-2-(phenylamino)pent-4-enoate **(4c)**	**S32**
6.4	Benzyl 5,5-difluoro-2,4-diphenyl-2-(phenylamino)pent-4-enoate **(4d)**	**S32**
6.5	Prop-2-yn-1-yl 5,5-difluoro-2,4-diphenyl-2-(phenylamino)pent-4-enoate **(4e)**	**S33**
6.6	But-3-en-1-yl 5,5-difluoro-2,4-diphenyl-2-(phenylamino)pent-4-enoate **(4f)**	**S33**
6.7	(3*S*,8*S*,9*S*,10*R*,13*R*,14*S*,16*S*)-10,13-dimethyl-16-((*S*)-6-methylheptan-2-yl)-2,3,4,7,8,9,10,11,12,13,14,15,16,17-tetradecahydro-1H-cyclopenta[a]phenanthren-3-yl 5,5-difluoro-2-((4-methoxyphenyl)amino)-2,4-diphenylpent-4-enoate **(4g)**	**S34**
6.8	Methyl 2-(4-bromophenyl)-5,5-difluoro-4-phenyl-2-(phenylamino)pent-4-enoate **(4h)**	**S35**
6.9	Methyl 5,5-difluoro-4-phenyl-2-(phenylamino)-2-(p-tolyl)pent-4-enoate **(4i)**	**S35**
6.10	Methyl 5,5-difluoro-2-(4-methoxyphenyl)-4-phenyl-2-(phenylamino)pent-4-enoate	**S36**

	**(4j)**	
6.11	Methyl 5,5-difluoro-4-phenyl-2-(phenylamino)-2-(thiophen-2-yl)pent-4-enoate **(4k)**	**S37**
6.12	Methyl 5,5-difluoro-4-phenyl-2-(phenylamino)-2-(4-(trifluoromethyl)phenyl)pent-4-enoate **(4l)**	**S37**
6.13	Methyl 2-((2-ethylphenyl)amino)-5,5-difluoro-2,4-diphenylpent-4-enoate **(4m)**	**S38**
6.14	Methyl 2-((3-bromophenyl)amino)-5,5-difluoro-2,4-diphenylpent-4-enoate **(4n)**	**S39**
6.15	Methyl 5,5-difluoro-2-((3-fluorophenyl)amino)-2,4-diphenylpent-4-enoate **(4o)**	**S39**
6.16	Methyl 5,5-difluoro-2-((4-methoxyphenyl)amino)-2,4-diphenylpent-4-enoate **(4p)**	**S40**
6.17	Methyl 5,5-difluoro-2,4-diphenyl-2-((3-(trifluoromethoxy)phenyl)amino)pent-4-enoate **(4q)**	**S41**
6.18	Methyl 2-((3,5-dimethoxyphenyl)amino)-5,5-difluoro-2,4-diphenylpent-4-enoate **(4r)**	**S41**
6.19	Methyl 5,5-difluoro-2,4-diphenyl-2-(p-tolylamino)pent-4-enoate **(4s)**	**S42**
6.20	Methyl 2-((4-chlorophenyl)amino)-5,5-difluoro-2,4-diphenylpent-4-enoate (**4t)**	**S43**
6.21	Methyl 2-((4-bromophenyl)amino)-5,5-difluoro-2,4-diphenylpent-4-enoate **(4u)**	**S43**
6.22	Methyl 5,5-difluoro-2-((4-isopropylphenyl)amino)-2,4-diphenylpent-4-enoate **(4v)**	**S44**
6.23	Methyl 4-(4-(tert-butyl)phenyl)-5,5-difluoro-2-phenyl-2-(phenylamino)pent-4-enoate **(4w)**	**S45**
6.24	Methyl 5,5-difluoro-4-(4-methoxyphenyl)-2-phenyl-2-(phenylamino)pent-4-enoate **(4x)**	**S45**
6.25	Methyl 4-(4-benzylphenyl)-5,5-difluoro-2-phenyl-2-(phenylamino)pent-4-enoate **(4y)**	**S46**
6.26	Methyl 4-(4-bromophenyl)-5,5-difluoro-2-phenyl-2-(phenylamino)pent-4-enoate **(4z)**	**S46**
6.27	Methyl 4-(3,5-dichlorophenyl)-5,5-difluoro-2-phenyl-2-(phenylamino)pent-4-enoate **(4aa)**	**S47**
6.28	Methyl 3-methyl-2-phenyl-2-(phenylamino)butanoate **(6a)**	**S48**
6.29	Benzyl 3-methyl-2-phenyl-2-(phenylamino)butanoate **(6b)**	**S48**
6.30	Isopropyl 3-methyl-2-phenyl-2-(phenylamino)butanoate **(6c)**	**S49**
6.31	Prop-2-yn-1-yl 3-methyl-2-phenyl-2-(phenylamino)butanoate **(6d)**	**S49**
6.32	But-3-en-1-yl 3-methyl-2-phenyl-2-(phenylamino)butanoate **(6e)**	**S50**
6.33	(1*S*,2*R*,5*S*)-2-Isopropyl-5-methylcyclohexyl 3-methyl-2-phenyl-2(phenylamino)butanoate **(6f)**	**S50**
6.34	(3*S*,8*S*,9*S*,10*R*,13*R*,14*S*,16*S*)-10,13-Dimethyl-16-((*S*)-6-methylheptan-2-yl)-2,3,4,7,8,9,10,11,12,13,14,15,16,17-tetradecahydro-1H-cyclopenta[a]phenanthren-3-	**S51**

yl 3-methyl-2-phenyl-2-(phenylamino)butanoate **(6g)**

6.35 Methyl 2-(4-bromophenyl)-3-methyl-2-(phenylamino)butanoate **(6h)** **S51**

6.36 Methyl 3-methyl-2-(phenylamino)-2-(4-(trifluoromethyl)phenyl)butanoate **(6i)** **S52**

6.37 Methyl 3-methyl-2-(phenylamino)-2-(thiophen-2-yl)butanoate **(6j)** **S52**

6.38 Methyl 2-((4-methoxyphenyl)amino)-3-methyl-2-phenylbutanoate **(6k)** **S53**

6.39 Methyl 2-((4-chlorophenyl)amino)-3-methyl-2-phenylbutanoate **(6l)** **S53**

6.40 Methyl 2-((4-bromophenyl)amino)-3-methyl-2-phenylbutanoate **(6m)** **S54**

6.41 Methyl-2-((2,6-dimethylphenyl)amino)-3-methyl-2-phenylbutanoate **(6n)** **S54**

6.42 Methyl 2-((2-ethylphenyl)amino)-3-methyl-2-phenylbutanoate **(6o)** **S55**

6.43 Methyl 2-((3,5-bis(trifluoromethyl)phenyl)amino)-3-methyl-2-phenylbutanoate **(6p)** **S55**

6.44 Methyl 2-cyclohexyl-2-phenyl-2-(phenylamino)acetate **(6q)** **S56**

6.45 Methyl 2-cyclobutyl-2-phenyl-2-(phenylamino)acetate **(6r)** **S56**

6.46 Methyl (3*R*)-3,7-dimethyl-2-phenyl-2-(phenylamino)oct-6-enoate **(6s)** **S57**

6.47 Methyl (3*R*)-4-(4-(tert-butyl)phenyl)-3-methyl-2-phenyl-2-(phenylamino)butanoate **(6t)** **S57**

6.48 Methyl (3*S*)-3-(1H-indol-1-yl)-2-phenyl-2-(phenylamino)butanoate **(6u)** **S58**

6.49 Methyl 4-methyl-2-phenyl-2-(phenylamino)pentanoate **(6v)** **S58**

6.50 Methyl 2,4-diphenyl-2-(phenylamino)butanoate **(6w)** **S59**

6.51 Methyl 2-phenyl-2-(phenylamino)pentanoate **(6x)** **S59**

6.52 Methyl 2-phenyl-2-(phenylamino)hexanoate **(6y)** **S60**

6.53 Methyl 3-(benzyloxy)-2-phenyl-2-(phenylamino)propanoate **(6z)** **S60**

6.54 Methyl 2,3-diphenyl-2-(phenylamino)propanoate **(6aa)** **S61**

6.55 Methyl 3,3-dimethyl-2-phenyl-2-(phenylamino)butanoate **(6ab)** **S61**

6.56 Methyl 3,3-dimethyl-2,4-diphenyl-2-(phenylamino)butanoate **(6ac)** **S62**

6.57 Methyl 4-cyano-2-phenyl-2-(phenylamino)butanoate **(8a)** **S62**

6.58 5-(Tert-butyl) 1-methyl 2-phenyl-2-(phenylamino)pentanedioate **(8b)** **S63**

6.59 5-Benzyl 1-methyl 2-phenyl-2-(phenylamino)pentanedioate **(8c)** **S63**

6.60 Methyl 5-(dimethylamino)-5-oxo-2-phenyl-2-(phenylamino)pentanoate **(8d)** **S64**

6.61 N-(4,4-difluoro-1,1,3-triphenylbut-3-en-1-yl)aniline (**8e**) **S64**

6.62 Diethyl 2-(3,3-difluoro-2-phenylallyl)-2-(phenylamino)malonate **(8f)** **S65**

6.63 (*S*)-N-(2-methyl-1-phenylpropyl)aniline **(9a)** **S65**

6.64 Diethyl 2-isopropyl-2-(phenylamino)malonate **(9b)** **S66**

6.65 *N*-(2-methyl-1,1-diphenylpropyl)aniline **(9c)** **S66**

6.66 *N*-(3-methyl-2-phenylbutan-2-yl)aniline **(9d)** **S67**

6.67 2-methoxy-*N*-(2-methyl-1,1-diphenylpropyl)aniline **(9e)** **S67**

6.68 *N*-(1,1,1-trifluoro-3-methyl-2-phenylbutan-2-yl)aniline **(9f)** **S68**

6.69 *N*-benzyl-2-methyl-1,1-diphenylpropan-1-amine **(9g)** **S68**

7.0 NMR Spectra S69

8.0 X-ray single crystal data S170

9.0 Computational details S175

10.0 References S203

1.0 General Information:

All the reactions were performed in flame-dried glassware under an argon atmosphere unless otherwise stated. Liquids and solutions were transferred with syringes. The solvents used were dried and purified by following standard procedures. Technical grade solvents for extraction or chromatography (ethyl acetate, and petroleum ether) were distilled before use. $CDCl_3$ was stored over 4Å molecular sieves. Chemicals used in this project were purchased from Sigma-Aldrich, TCI, Alfa-Aesar and Sisco Research Laboratories (SRL) and used without further purification. All the liquid chemicals were distilled freshly prior to use. Blue LEDs purchased from APSTRONICS and KESSIL. Analytical thin-layer chromatography (TLC) was performed on using pre-coated aluminium-backed plates (Merck Kieselgel 60 F254) and visualized by UV radiation, and basic aqueous potassium permangante ($KMnO_4$) stain as developing agents.

Column chromatography was performed on silica gel 60 (40–63 µm, 230–400 mesh, ASTM) from Merck using the indicated solvents. Organic solutions were concentrated under reduced pressure on Heidolph rotary evaporator. NMR spectra were acquired on a JEOL JNM ECS-400, instrument running at 400 MHz for ^{1}H, 101 MHz ^{13}C and 376 MHz for ^{19}F. Chemical shifts (δ) are reported in ppm relative to residual solvent signals ($CDCl_3$, 7.26 ppm for **^{1}H NMR**, $CDCl_3$, 77.16 ppm for **^{13}C NMR**). Fluorobenzene was used as an internal standard to calculate NMR yields. Data are reported as follows: chemical shift, multiplicity (br = broad singlet, s = singlet, d = doublet, dd = doublet of doublets, t = triplet, q = quartet, ddd = doublet of doublet of doublet, td = triplet of doublet, m = multiplet), coupling constants (Hz), and integration. All fluorescence data were recorded using Perkin Elmer LS55 fluorescence spectrophotometer instrument. UV-Visible data was recorded on Shimadzu UV-2600.

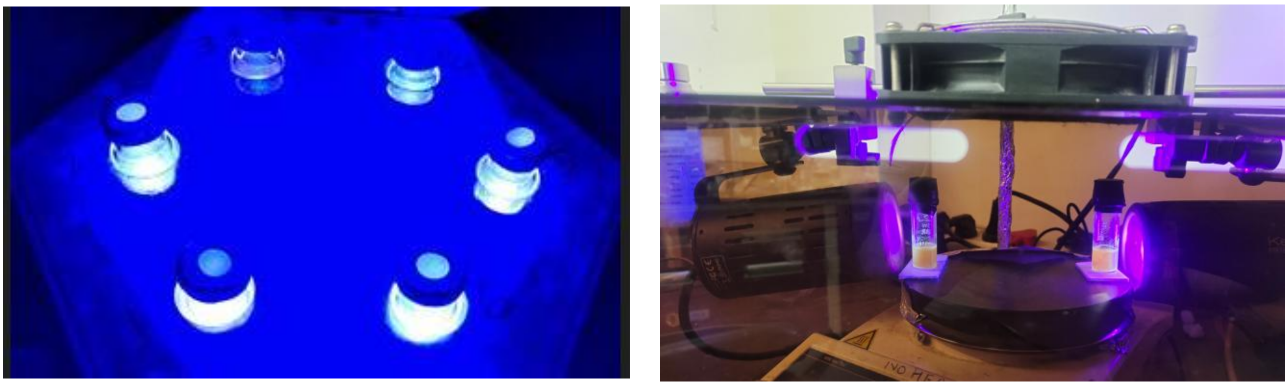

APSTRONICS 455 nm **KESSIL 390 nm**

Figure S1: *Photochemical reaction set up.*

2.0 List of starting substrates prepared:

The lists of substrates prepared according to **GP-1 to 3** are given below.

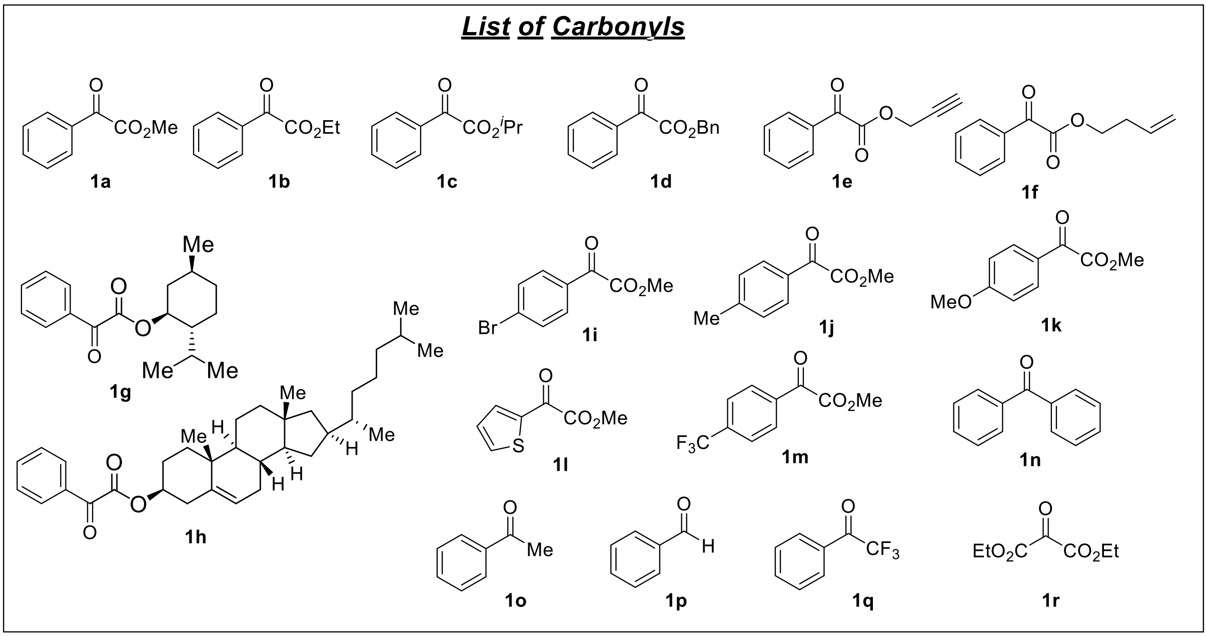

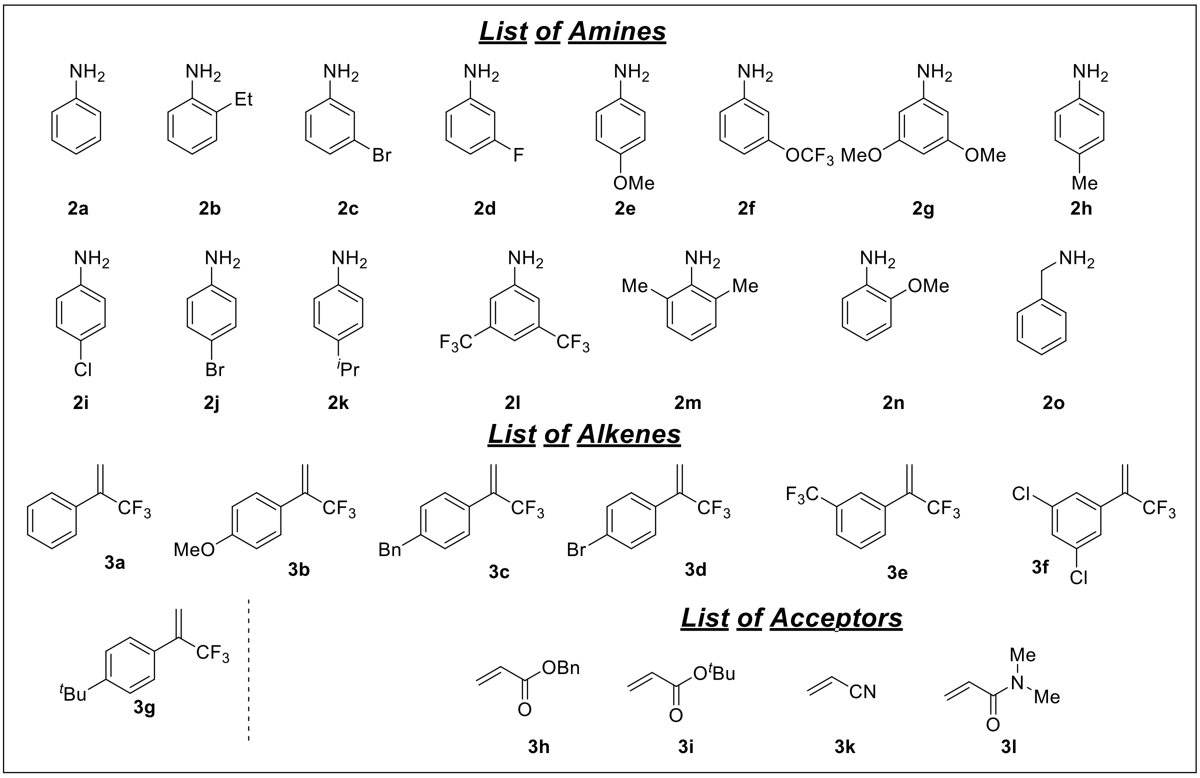

List of DHPs

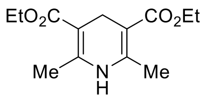
5a

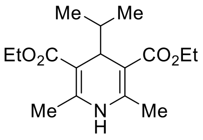
5b

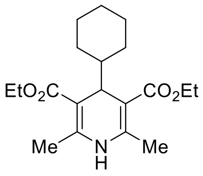
5c

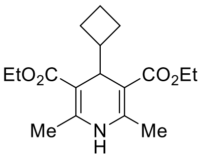
5d

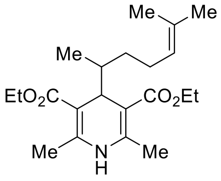
5e

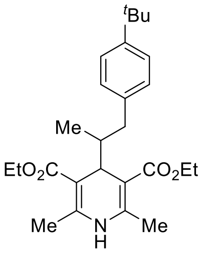
5f

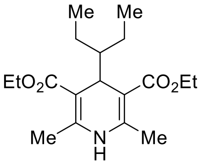
5g

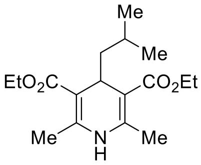
5h

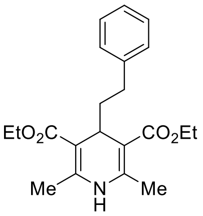
5i

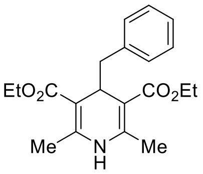
5j

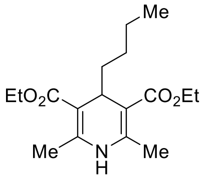
5k

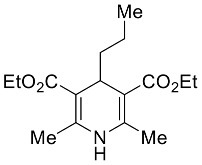
5l

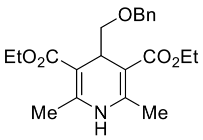
5m

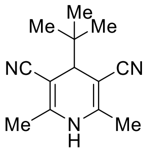
5n

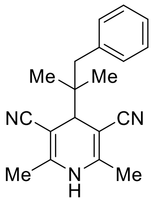
5o

3.0 Optimization Table:

Table S1: *Evaluation of bases*

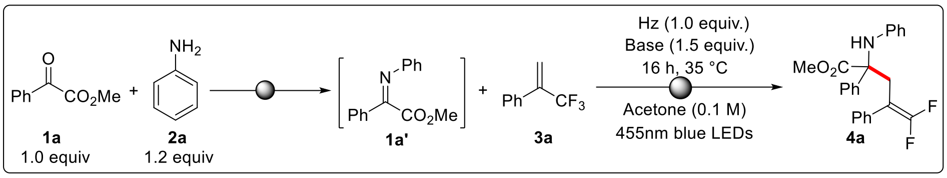

Entry	base	Yield (%)[a]
1	K_2CO_3	25
2	Na_2CO_3	10
3	K_3PO_4	n.r.
4	$MgSO_4$	n.r.
**5**	**Cs_2CO_3**	**94 (89)[b]**
6	Li_2CO_3	Trace
7	NEt_3	n.r.
8	DIPEA	n.r.
9	DABCO	n.r.
10	NaOAc	n.r.
11	Ag_2CO_3	5
12	KH_2PO_4	n.r.

[a]Reaction scale 0.1 mmol, yields reported are the NMR yield using Fluorobenzene as internal standard. [b]Yields reported are the isolated yield.

Table S2: *Evaluation of solvents*

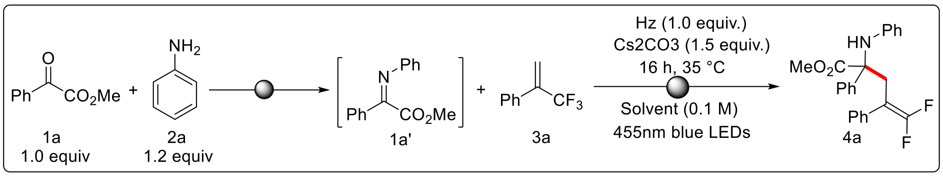

Entry	solvent	Yield (%)[a]
1	ACN	70
2	DCM	35
3	Benzene	20
4	HFIP	n.r.
5	EtOH	n.r.
6	DMF	45
7	DMSO	55
8	DMA	74
**9**	**Acetone**	**94 (89)[b]**
10	THF	27
11	EtOAc	Trace
12	1,4-Dioxane	44
13	$CHCl_3$	30
14	DMPU	27

[a]Reaction scale 0.1 mmol, yields reported are the NMR yield using Fluorobenzene as internal standard. [b]Yields reported are the isolated yield.

Table S3: *Evaluation of equivalency*

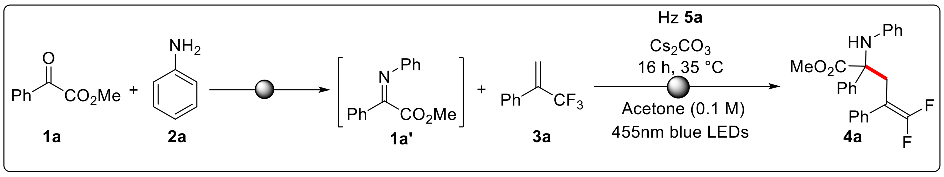

Entry	1a'	3a	5a	Cs_2CO_3	Yield (%)[a]
1	1	1	1	1	30%
2	1	2	1	1	56%
3	1	3	1	1	84%
4	1	3	2	1	86%
**5**	**1**	**3**	**1**	**1.5**	**94%**
6	1	3	0.5	1.5	67%

[a]Reaction scale 0.1 mmol, yields reported are the NMR yield using Fluorobenzene as internal standard.

Table S4: *Evaluation of light source*

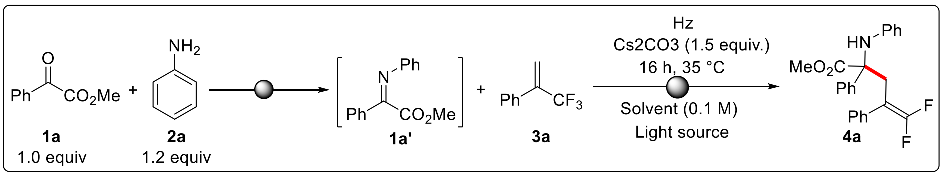

Entry	Light source	Yield (%)[a]
1	455 nm	94
2	427 nm	45
3	390 nm	20
4	370 nm	Trace

[a]Reaction scale 0.1 mmol, yields reported are the NMR yield using Fluorobenzene as internal standard.

Table S5: *Evaluation of bases*

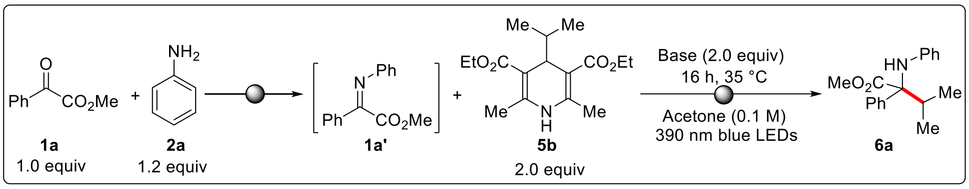

Entry	base	Yield (%)[a]
1	K_2CO_3	Trace
2	Li_2CO_3	n.r.
3	K_3PO_4	56
4	Na_2HPO_4	n.r.
**5**	**Cs_2CO_3**	**91 (86)[b]**
6	K_2HPO_4	30
7	NaOAc	n.r.
7	NEt_3	n.r.
8	DIPEA	n.r.
9	KO*t*Bu	n.r.

[a]Reaction scale 0.1 mmol, yields reported are the NMR yield using Dibromomethane as internal standard. [b]Yields reported are the isolated yield.

Table S6: *Evaluation of solvents*

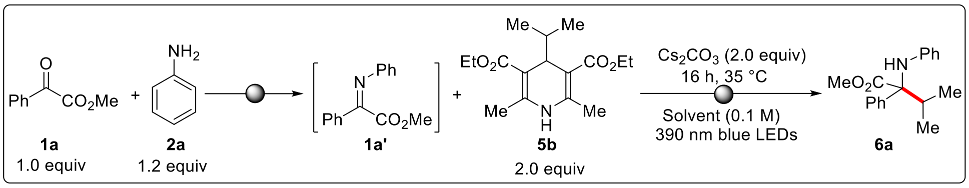

Entry	solvent	Yield (%)[a]
1	ACN	86
2	DCM	n.r.
3	DCE	30
4	HFIP	n.r.
5	EtOH	n.r.
6	DMF	n.r.
7	DMSO	25
**9**	**Acetone**	**91 (86)[b]**
10	THF	n.r.
11	EtOAc	n.r.

[a]Reaction scale 0.1 mmol, yields reported are the NMR yield using Dibromomethane as internal standard. [b]Yields reported are the isolated yield.

Table S7: *Evaluation of equivalency*

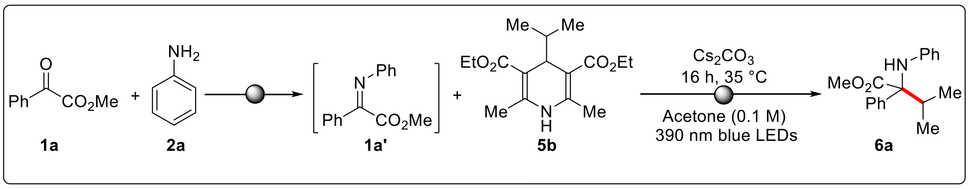

Entry	1a'	5b	Cs_2CO_3	Yield (%)[a]
1	2	1	2	45
2	1	1	2	55
**3**	**1**	**2**	**2**	**91 (86)[b]**
4	1	2	1	72
5	1	2	1.5	80

[a]Reaction scale 0.1 mmol, yields reported are the NMR yield using Dibromomethane as internal standard. [b]Yields reported are the isolated yield.

Table S8: *Evaluation of light source*

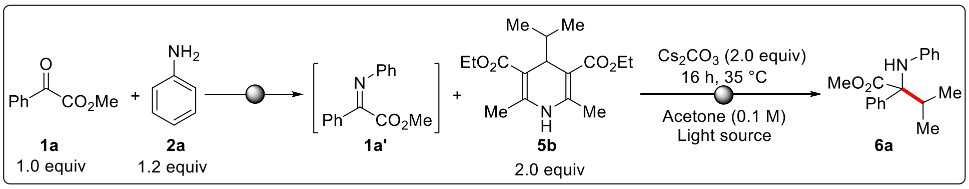

Entry	Light source	Yield (%)[a]
1	370 nm	trace
2	427 nm	77
3	455 nm	75
**4**	**390 nm**	**91 (86)[b]**

[a]Reaction scale 0.1 mmol, yields reported are the NMR yield using Dibromomethane as internal standard. [b]Yields reported are the isolated yield.

Table S9: *Control experiments*

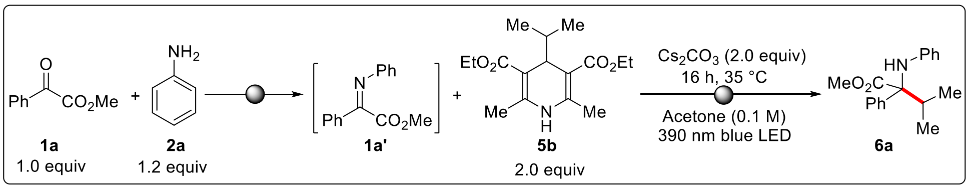

Entry	Deviation from Standard Conditions	Yield (%)[a]
1	open air	80
2	no base	60 [b]
3	no light	n.r.
**4**	**none**	**91 (86)[b]**

[a]Reaction scale 0.1 mmol, yields reported are the NMR yield using Dibromomethane as internal standard. [b]Yields reported are the isolated yield.

4.0 General Procedures

4.1 Preparation of ketoesters (**GP-1**)

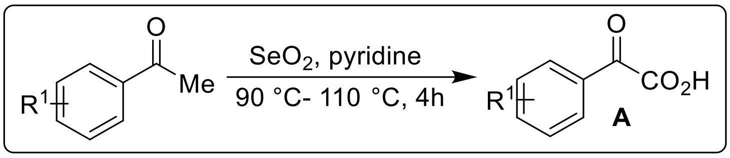

According to previous literature, [1] A round-bottomed flask was charged with Selenium Dioxide (1.5 equiv), aryl ketone derivative (1 equiv), and 20 ml pyridine was added to it. The reaction mixture was then stirred at 110°C for 1 h in an oil bath, and then the temperature was reduced to 90°C for 4 h. The desired product was isolated by column chromatography on silica gel using EtOAc-PE (5%) to give the substituted 2-Oxo-2- phenylacetic acid (**A**) in 65−90% yield.

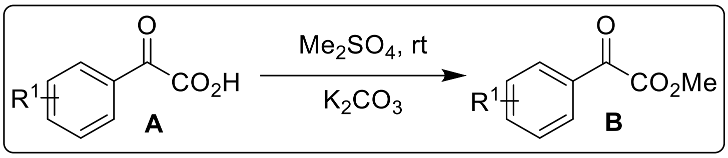

According to previous literature, [2] Dimethyl sulfate (DMS) is in the presence of K_2CO_3, with DMSO being taken as solvent. A 100 ml round bottom flask was charged with the phenylacetic acid (1 equiv), and combined with potassium carbonate (1.45 equiv) in 0.5 M of DMSO. Dimethyl sulfate (1.20 equiv) was added drop-wise. After 20 min of stirring at room temperature, the reaction mixture was then transferred to a separatory funnel, mixed with ether, and the organic layer was washed three times with a dilute potassium carbonate solution and once with brine. The organic layer was dried with anhydrous magnesium sulfate, filtered, and dried by rotary evaporation to yield the desired product **B** (40-90% yield).

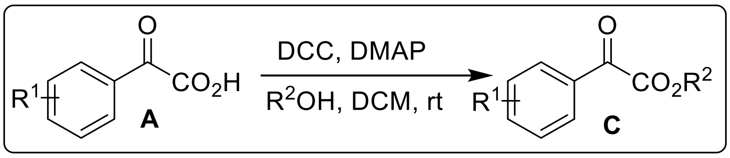

According to previous literature, [3] to a solution of 2-oxo-2-phenylacetic acid (1.0 equiv) in anhydrous DCM (0.3 M) under ice bath and N2 atmosphere, DMAP (0.1 equiv), DCC (1.0 equiv) and anhydrous EtOH (2.0 equiv) were added in turn. The mixture was then allowed to ambient temperature automatically and stirred until the full conversion of the starting material by TLC monitoring. The mixture was filtered through celite, with DCM as eluant. The

mother liquid and the DCM eluate were combined, and washed by 5% CuSO4 aqueous solution, water and brine, then dried over Na_2SO_4. The solvent was concentrated in vacuo and purified by column chromatography to afford the corresponding α-keto ester.

4.2 Preparation of 1,4-Dihydropyridines (**GP-2**)

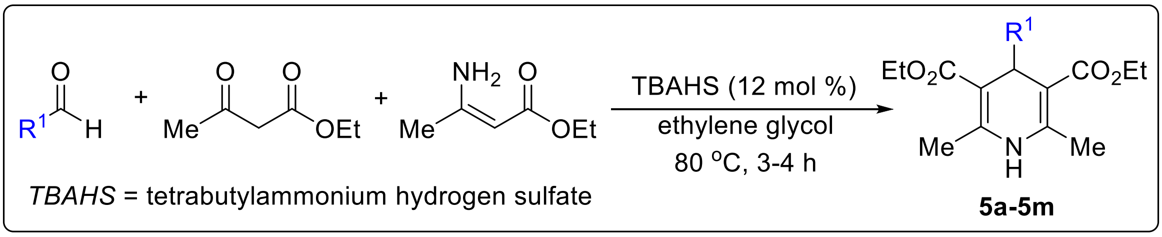

According to previous literature, [4], into a round-bottom flask charged with ethyl 3-aminocrotonate (1.0 equiv) was added ethylene glycol (2.5 M). Next, ethyl acetoacetate (1.0 equiv) was added followed by the aldehyde (1.0 equiv). Finally, Bu_4NHSO_4 (12 mol %) was added in one portion. The flask was closed with a septum and heated at 80 ºC for 3-4 h. At this time, the reaction was cooled to r.t. and diluted with EtOAc. The solution was poured into a separatory funnel containing brine and extracted three times with EtOAc. After drying over $MgSO_4$, it was filtered and taken to dryness. The crude reaction mixture was purified by column chromatography using hexanes/EtOAc (0 to 40%) as eluent.

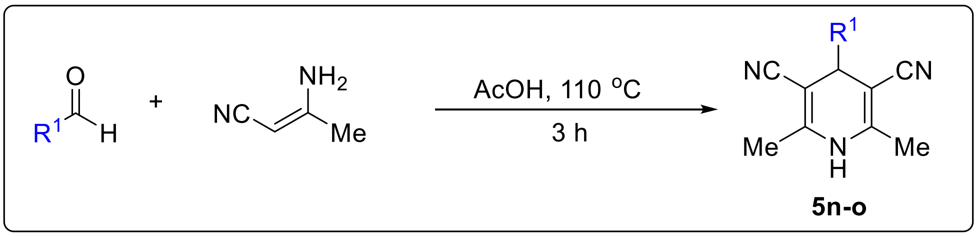

According to previous literature, [5,6] a round-bottom flask was charged with 3-aminocrotononitrile (2.0 equiv), aldehyde (1.0 equiv) in glacial AcOH. The reaction was heated at 110 ºC with stirring for 3 h. Then it was allowed to cool to r.t., diluted with H_2O, and extracted with EtOAc three times. The combined organic layers were neutralized with a saturated solution of $NaHCO_3$ until a neutral pH was reached, washed with brine, dried (MgSO4), and filtered. The crude reaction mixture was purified by column chromatography using hexanes/EtOAc (0 to 40%) as eluent.

4.3 Preparation of trifluoromethyl styrenes (**GP-3**)

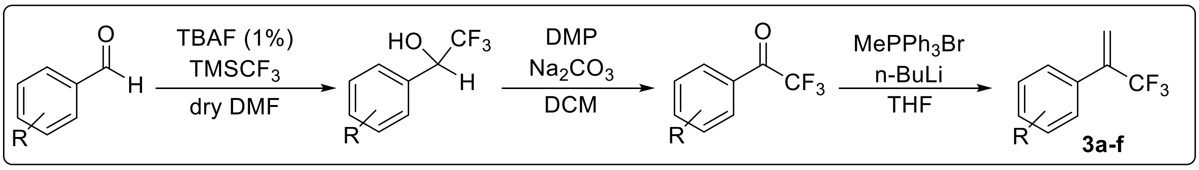

Step 1: According to previous literature, [7] to a solution of aldehyde (5 mmol) in DMF (5 mL) in a 25 mL round-bottom flask equipped under nitrogen atmosphere, $TMSCF_3$ (6.5 mmol, 1.3 equiv.) was added and the mixture was stirred in an ice bath. After approximately 10 min, TBAF (1 M in THF, 0.05 mmol, 0.05 mL, 0.01 equiv.) was added dropwise via a syringe. After 10 min, the ice bath was removed and the solution was stirred for approximately 6 h at room temperature. To cleave the silyl ether intermediate, the reaction mixture was cooled to 0 °C in an ice bath and after 10 min; water and TBAF (1 M in THF, 0.5 mL, 0.5 mmol, 0.1 equiv.) were added. The ice bath was removed and the reaction mixture was stirred at room temperature. Finally, the mixture was extracted with ethyl acetate (20 mL×3). The organic phase was washed with brine and then dried over anhydrous Na_2SO_4. After filtration and evaporation under vacuum, the residue was subjected to silica gel column chromatography using hexane/ethyl acetate as eluent to give trifluoromethyl alcohols.

Step 2: According to previous literature, [8] to a solution of the α-CF_3 alcohol (5 mmol, 1 equiv.) in DCM (30 mL), was added DMP (17.5 mmol, 3.5 equiv.) and Na_2CO_3 (20 mmol, 4 equiv.). The solution was stirred at room temperature for 3 h. Then water was added and the obtained suspension was stirred for an additional hour, the mixture was extracted with DCM (20 mL × 3). The organic phase was washed with brine and then dried over anhydrous Na_2SO_4. The solvent was removed *in vacuo* by rotary evaporation in a room-temperature water bath to give α-CF_3 ketones 1 (yield up to 97 %).

Step 3: According to previous literature, [9] an oven dried flask was charged with methyl triphenylphosphonium bromide (1.25 equiv.) and THF (0.8 M) was added under an argon atmosphere. The resulting suspension was cooled to 0 °C and n-BuLi (1.2 equiv.) was added dropwise. The orange solution was stirred for 10 minutes and cooled to -78 °C at which point a solution of trifluoromethyl ketone (1 equiv.) in THF (1 M) was added. The cooling bath was removed and the solution was allowed to warm to r.t. After full conversion of the ketone monitored by TLC, the reaction was quenched with an aqueous solution of NH_4Cl (sat.). Et_2O was added and the layers were separated. The organic layer was dried over anhydrous Na_2SO_4. Silica was added to the organic layer, and the solvent was removed under vacuum.

The residue was purified by column chromatography over silica gel (hexane) to give the desired styrene.

4.4 Photoinduced carbonyl alkylative amination using radical trapper (**GP-4**).

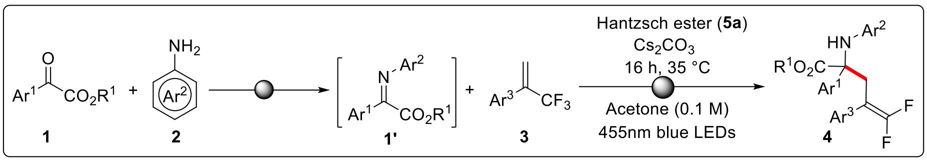

In an oven dried vial with a magnetic stirrer bar, carbonyl **1** (0.2 mmol, 1.0 equiv), amine **2** (0.24 mmol, 1.2 equiv) and *p*-TSA (0.01 mmol, 0.05 equiv) was mixed with 4**Å** molecular sieves in 1 ml benzene and was placed in 110 °C for 20 h. Then the mixture was cooled to room temperature and benzene was evaporated. The resulting crude mixture was taken inside the glove box to add the hantzsch ester **5a** (0.2 mmol, 1.0 equiv), Cs_2CO_3 (0.3 mmol, 1.5 equiv) and olefin (0.6 mmol, 3.0 equiv). After that, the reaction mixture was dissolved in 0.1 M of freshly distilled Acetone, followed by the vial being sealed with a teflon cap and wrapped with parafilm. The reaction mixture was then stirred under the irradiation of 455 nm light for about 16 h at 35 °C. After that, the crude mixture was concentrated and purified by flash column chromatography to afford the corresponding coupling product.

4.5 Photoinduced carbonyl alkylative amination using 4-alkyl-1,4-DHP (**GP-5**).

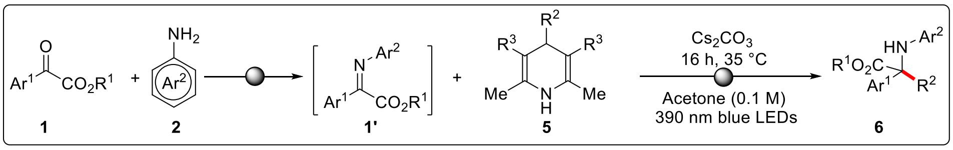

In an oven dried vial with a magnetic stirrer bar, carbonyl **1** (0.2 mmol, 1.0 equiv), amine **2** (0.24 mmol, 1.2 equiv) and *p*-TSA (0.01 mmol, 0.05 equiv) was mixed with 4**Å** molecular sieves in 1 ml benzene and was placed in 110 °C for 20 h. Then the mixture was cooled to room temperature and benzene was evaporated. The resulting crude mixture was taken inside the glove box to add the 4-alkyl-1,4-DHP **5** (0.4 mmol, 2.0 equiv), and Cs_2CO_3 (0.4 mmol, 2.0 equiv). After that, the reaction mixture was dissolved in 0.1 M of freshly distilled Acetone, followed by the vial being sealed with a teflon cap and wrapped with parafilm. The reaction mixture was then stirred under the irradiation of 390 nm light for about 16 h at 35 °C. After that, the crude mixture was concentrated and purified by flash column chromatography to afford the corresponding coupling product.

5. Mechanistic Studies

5.1 Using TEMPO as a radical scavenger

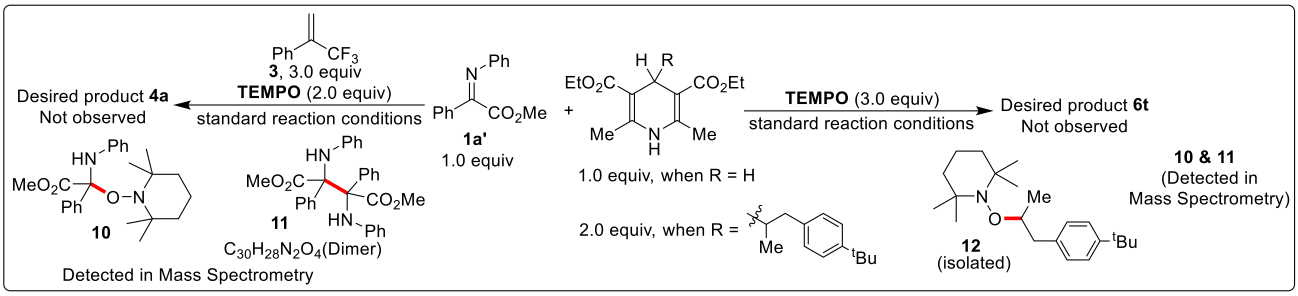

Inside the glove box, an oven-dried 5 mL glass vial was charged with **1a'** (48.0 mg, 0.2 mmol, 1.0 equiv), **5a** (50.5 mg, 0.2 mmol, 1.0 equiv), **3a** (105 µL, 0.6 mmol, 3.0 equiv), Cs_2CO_3 (97.5 mg, 0.3 mmol, 1.5 equiv) and **TEMPO** (63.0 mg, 0.4 mmol, 2.0 equiv) The reaction mixture was dissolved in 0.1 M dry Acetone and the vial was sealed with a Teflon cap and wrapped with parafilm in glove box. The resulting mixture was stirred under the irradiation of 455 nm LEDs for 16 h at 35 °C. The corresponding product **4a** was not observed based on **^{1}H** and **^{19}F NMR** analysis. The **TEMPO** adduct **10** was detected in mass spectrometry.

Similarly, inside the glove box, another oven-dried 5 mL glass vial was charged with **1a'** (48.0 mg, 0.2 mmol, 1.0 equiv), **5f** (171.0 mg, 0.4 mmol, 2.0 equiv), Cs_2CO_3 (130.0 mg, 0.4 mmol, 2.0 equiv) and **TEMPO** (93.5 mg, 0.6 mmol, 3.0 equiv) The reaction mixture was dissolved in 0.1 M dry Acetone and the vial was sealed with a Teflon cap and wrapped with parafilm in glove box. The resulting mixture was stirred under the irradiation of 390 nm LEDs for 16 h at 35 °C. The corresponding coupling product **6t** was not observed based on **^{1}H NMR** analysis. However, the **TEMPO** adduct **12** was isolated from the reaction mixture and was fully characterized. Here also, the **TEMPO** adduct **10** was detected in mass spectrometry.

A dimer **11** from the imine part was also detected in mass spectrometry for both the reactions.

Experimental details for the TEMPO adduct:

1-((1-(4-(Tert-butyl)phenyl)propan-2-yl)oxy)-2,2,6,6-tetramethylpiperidine (**12**)

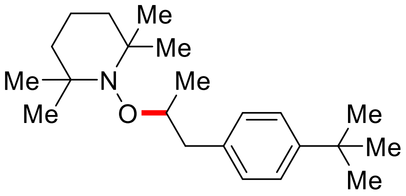

$C_{22}H_{37}NO$
M (g/mol): 331.54

^{1}H NMR (400 MHz, CDCl$_3$) δ 7.32 (d, *J* = 8.0 Hz, 2H), 7.16 (d, *J* = 8.0 Hz, 2H), 4.15–4.07 (m, 1H), 3.18 (dd, *J* = 12.9, 4.9 Hz, 1H), 2.52 (dd, *J* = 13.0, 8.5 Hz, 1H), 1.54–1.44 (m, 4H), 1.34 (s, 9H), 1.19–1.10 (m, 17H).

^{13}C NMR (101 MHz, CDCl$_3$) δ 148.6, 136.8, 129.3, 125.1, 79.9, 60.1, 59.5, 42.5, 40.4, 34.8, 34.5, 34.5, 31.6, 20.6, 20.5, 19.6, 17.5.

HRMS (ESI): m/z [M+H]$^+$ Calculated for [$C_{22}H_{38}NO$]$^+$: 332.2953; Found: 332.2965.

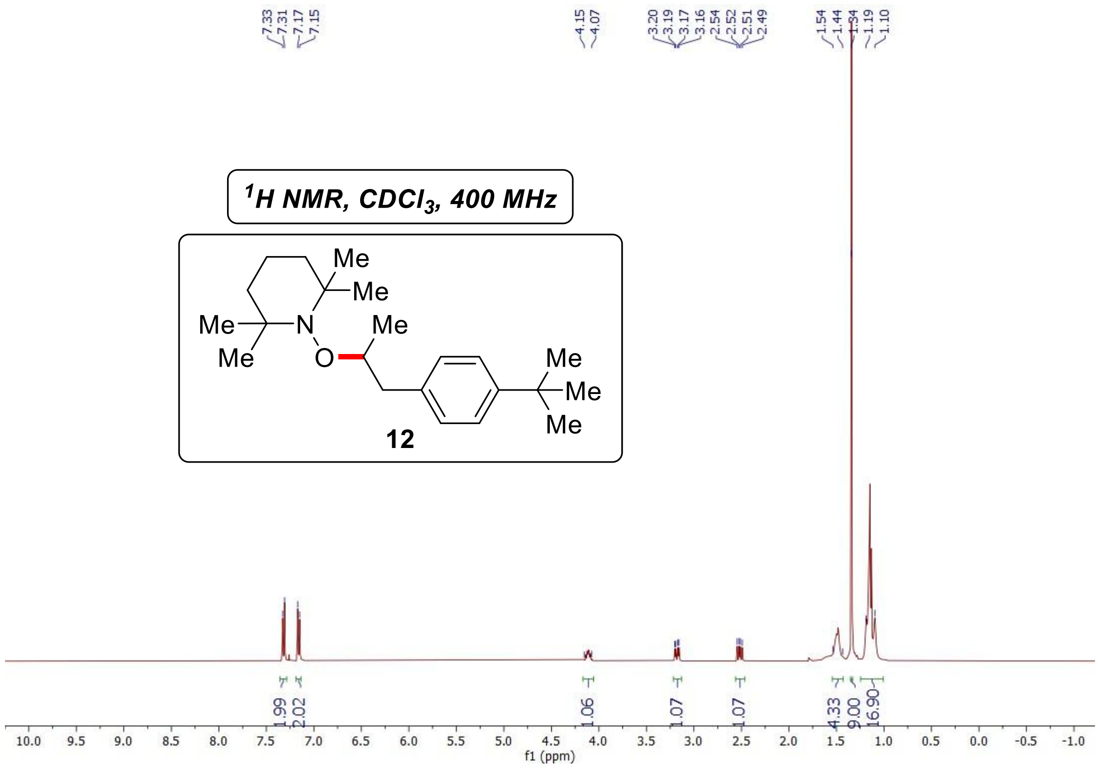

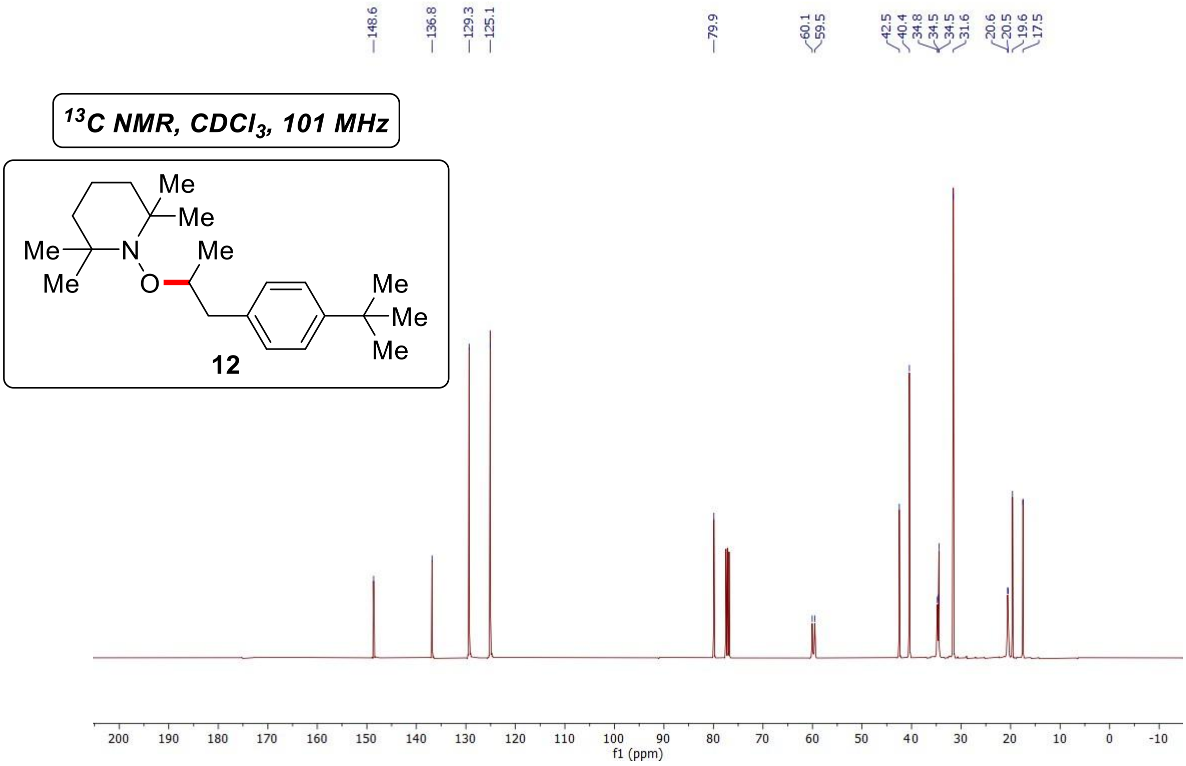

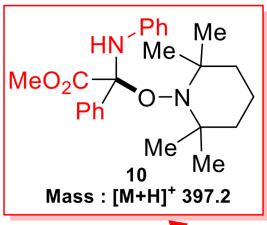

Elemental Composition Report **Page 1**

Single Mass Analysis
Tolerance = 100.0 PPM / DBE: min = -1.5, max = 50.0
Element prediction: Off
Number of isotope peaks used for i-FIT = 3

Monoisotopic Mass, Even Electron Ions
22 formula(e) evaluated with 1 results within limits (up to 50 best isotopic matches for each mass)
Elements Used:
C: 24-25 H: 32-100 N: 2-10 O: 3-5
300525_ICHP_TEMPO_1
300525_ICHP_TEMPO_1 21 (0.356) Cm (21:27)

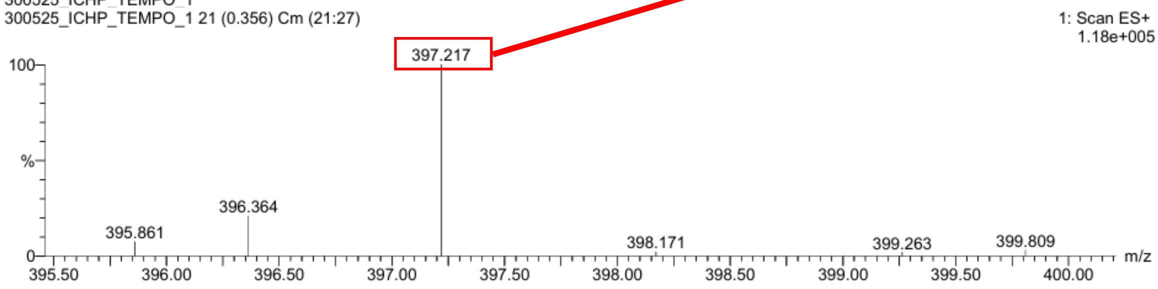

Minimum:				-1.5				
Maximum:		5.0	100.0	50.0				
Mass	Calc. Mass	mDa	PPM	DBE	i-FIT	Norm	Conf(%)	Formula
397.2175	397.2491	-31.6	-79.6	9.5	38.4	n/a	n/a	C24 H33 N2 O3

Elemental Composition Report **Page 1**

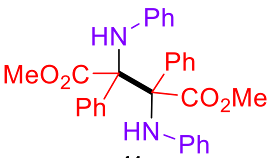

11
Mass : $[M+H]^+$ 481.21

Single Mass Analysis
Tolerance = 1000.0 PPM / DBE: min = -1.5, max = 50.0
Element prediction: Off
Number of isotope peaks used for i-FIT = 3

Monoisotopic Mass, Even Electron Ions
14 formula(e) evaluated with 1 results within limits (up to 50 best isotopic matches for each mass)
Elements Used:
C: 30-35 H: 28-100 N: 2-10 O: 4-5
300525_ICHP_TEMPO_2
300525_ICHP_TEMPO_2 14 (0.234)

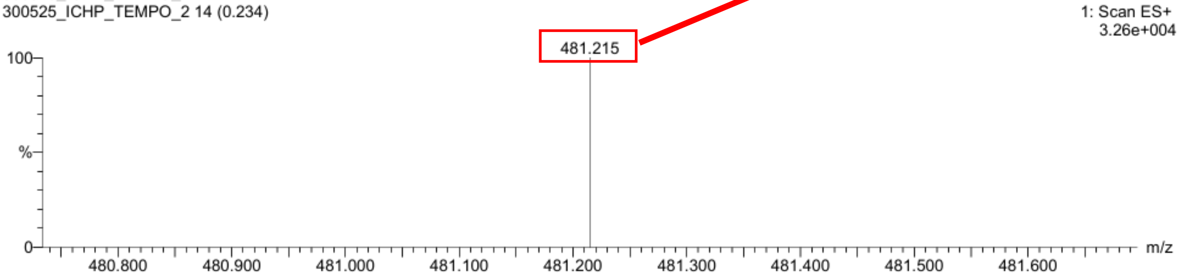

Minimum:				-1.5				
Maximum:		5.0	1000.0	50.0				
Mass	Calc. Mass	mDa	PPM	DBE	i-FIT	Norm	Conf(%)	Formula
481.2149	481.2127	2.2	4.6	17.5	32.6	n/a	n/a	C30 H29 N2 O4

5.2 UV-Visible Experiment

UV-Vis spectra were recorded using a Shimadzu UV-2600 UV-Vis Spectrophotometer. In a typical experiment, the samples were prepared in a 3 mL quartz cuvette with a path length of 1 cm. The final concentration of the single components: H-DHP (**5a**) (1.0•10-3 M), **1a**' (1.0•10-3 M), and Cs_2CO_3 (1.0•10-3 M) in DMA. All the combination was taken 1:1 mixture.

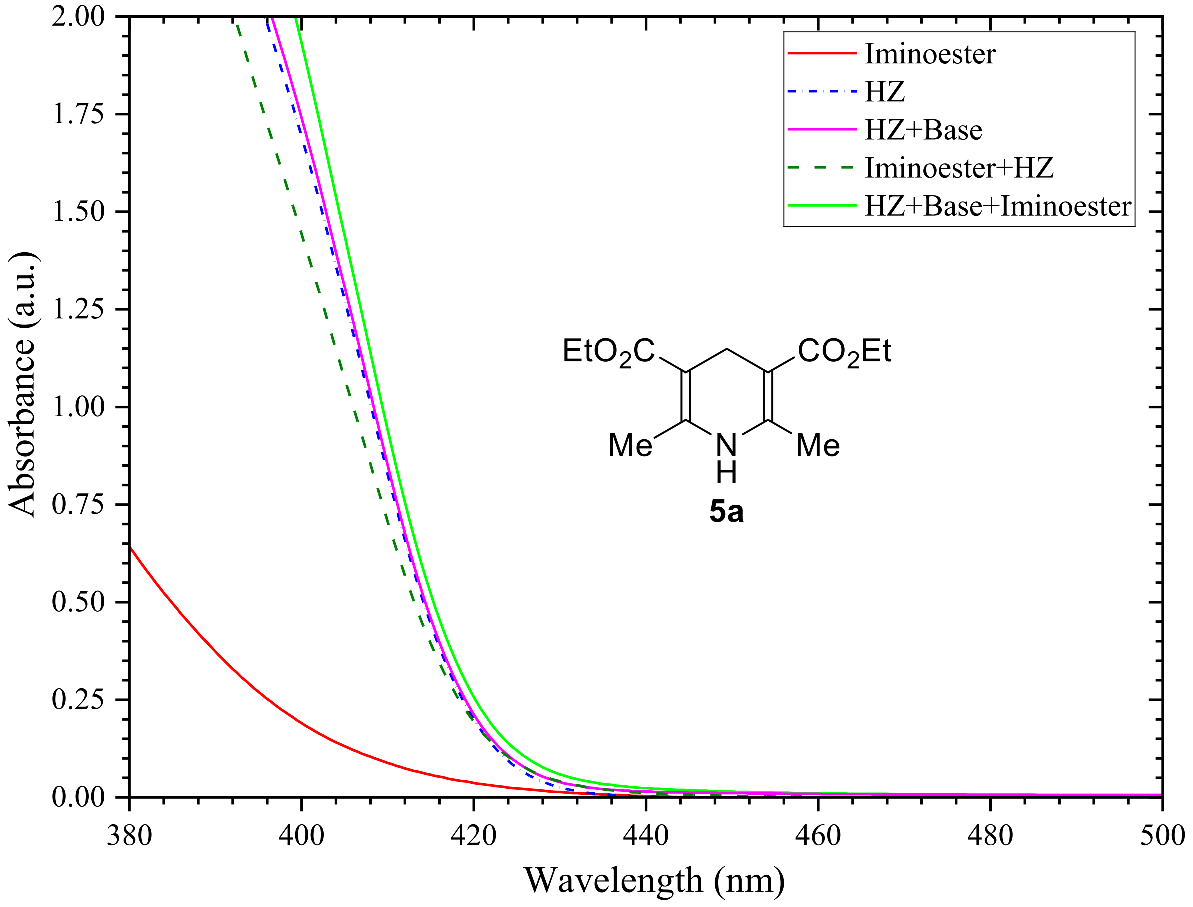

Figure S2: *UV-visible spectra of H-DHP **5a** with iminoester **1a'** in DMA*

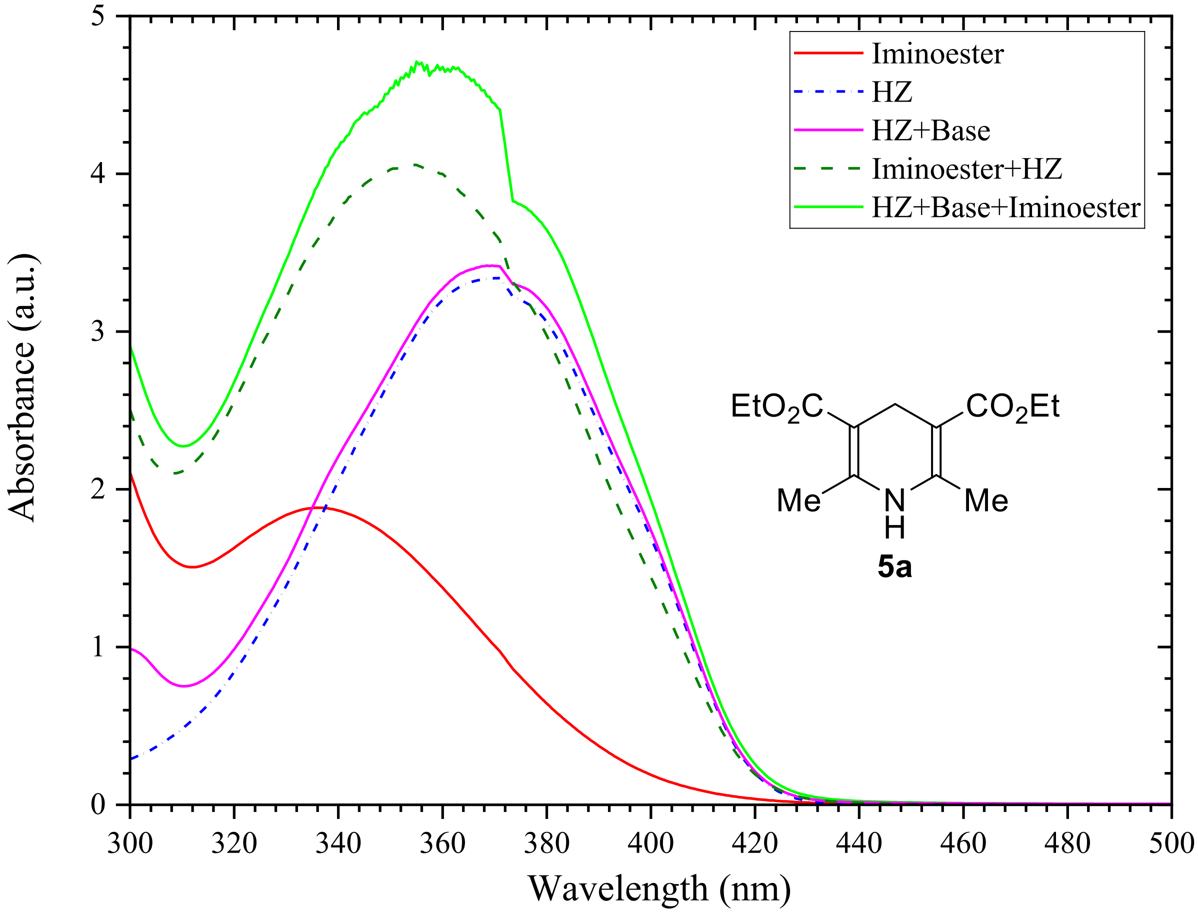

Figure S3: *UV-visible spectra of H-DHP* ***5a*** *with iminoester* ***1a'*** *in DMA*

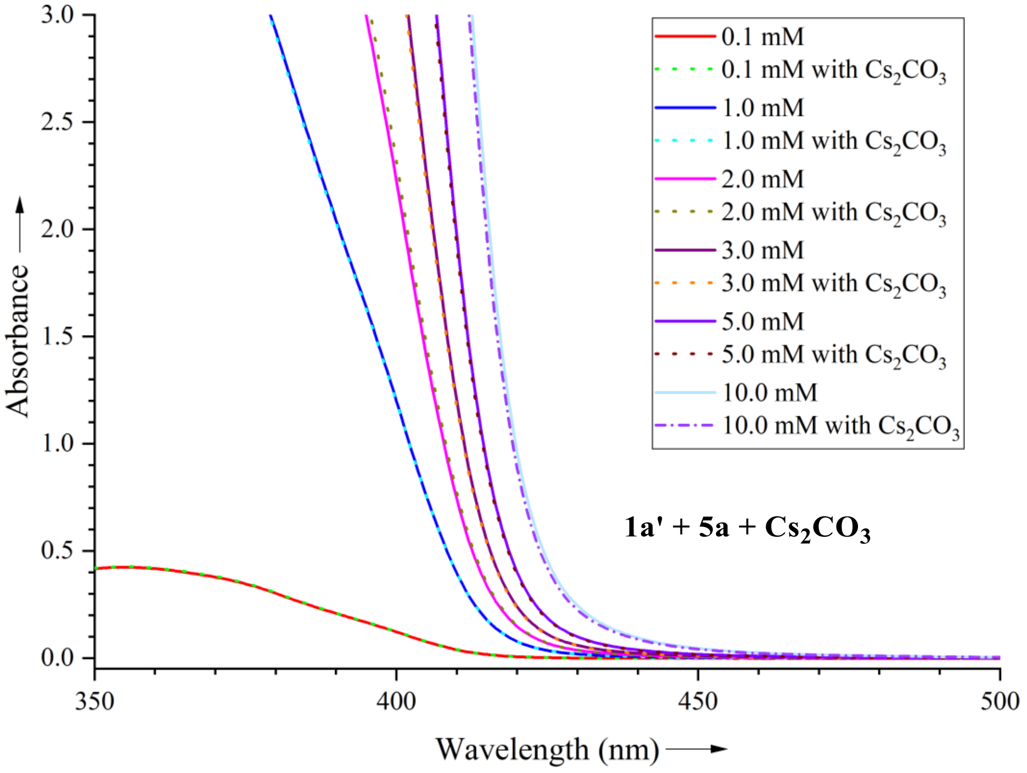

Figure S4: *UV-visible spectra with increasing concentration of H-DHP **5a**, iminoester **1a'** and Cs_2CO_3 in acetone*

UV-Vis spectra were recorded using a Shimadzu UV-2600 UV-Vis Spectrophotometer. In a typical experiment, the samples were prepared in a 3 mL quartz cuvette with a path length of 1 cm. The final concentration of the single components: 1,4-DHP (**5b**) (1.0•10-3 M), **1a'** (1.0•10-3 M) and Cs_2CO_3(1.0•10-3 M) in acetone. All the combination was taken 1:1 mixture.

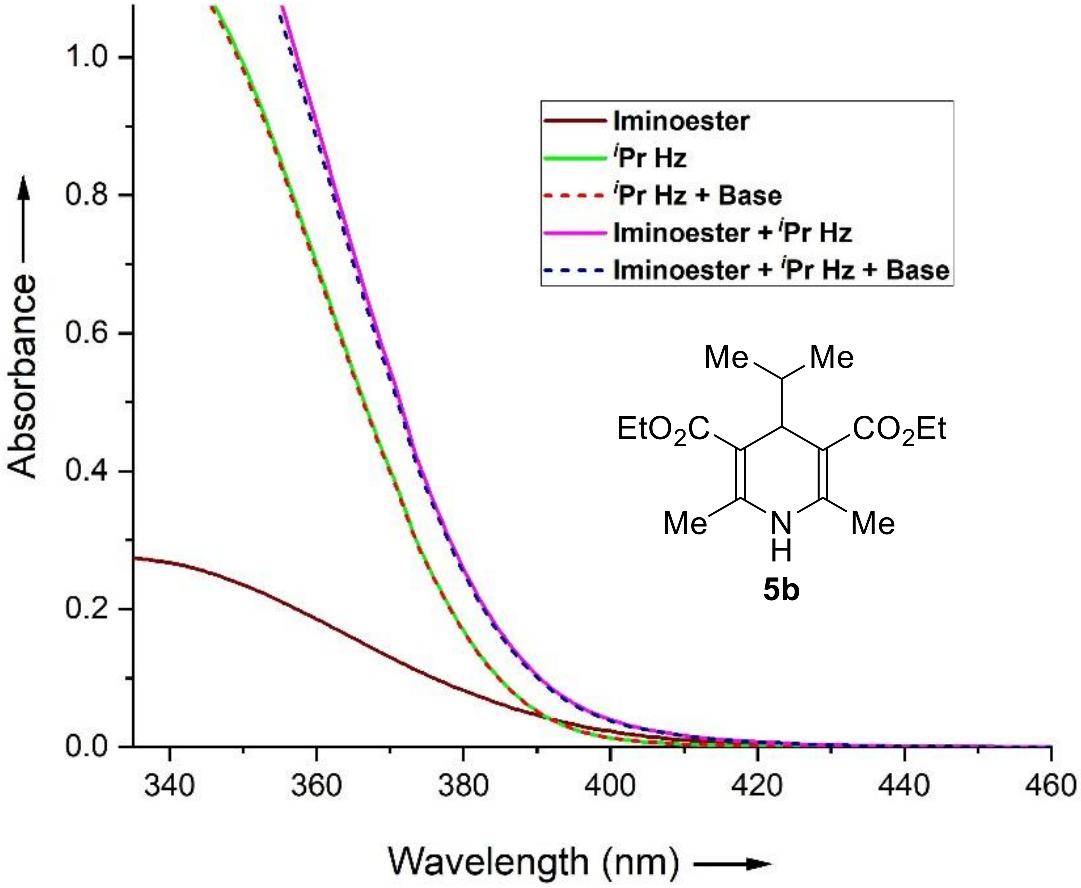

Figure S5: *UV-visible spectra of alkyl-DHP **5b** with iminoester **1a'** in acetone*

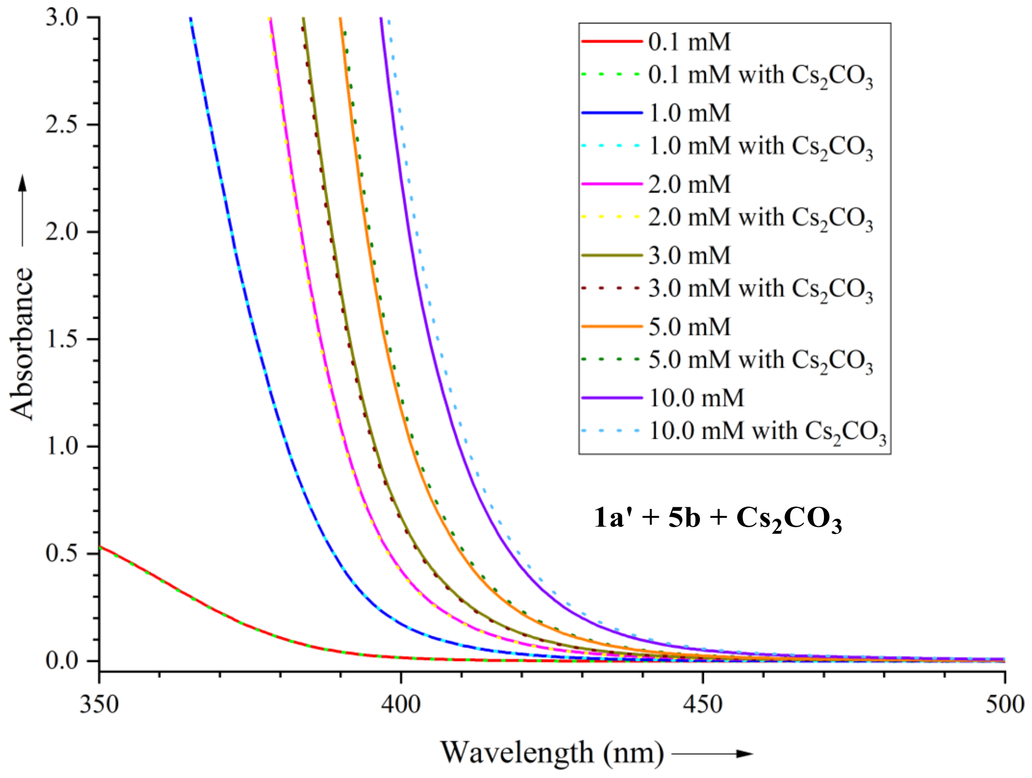

Figure S6: *UV-visible spectra with increasing concentration of Alkyl-DHP **5b**, iminoester **1a'** and Cs_2CO_3 in acetone*

5.3 Job's Plot

The Job's plot was constructed to evaluate the stoichiometry of the EDA complex, [10] between Hantzsch ester derivative **5a**, imine derivative **1a'** and base Cs_2CO_3 We measured the absorption of acetone solutions at 430 nm with different donor/ base/acceptor ratios, with constant concentration (0.01 M) of the three components. Initially we took the equimolar concentration of Hantzsch ester **5a** with base and gradually adding iminoester. All the absorption spectra were recorded in 1 cm path quartz cuvettes using Shimadzu UV-2600 UV-Vis Spectrophotometer. The absorbance values were plotted against the molar fraction (%) of H-DHP **5a**. The maximal absorbance at 50% molar fraction of **5a** indicated the 1:1:1 stoichiometry of the EDA complex in solution.

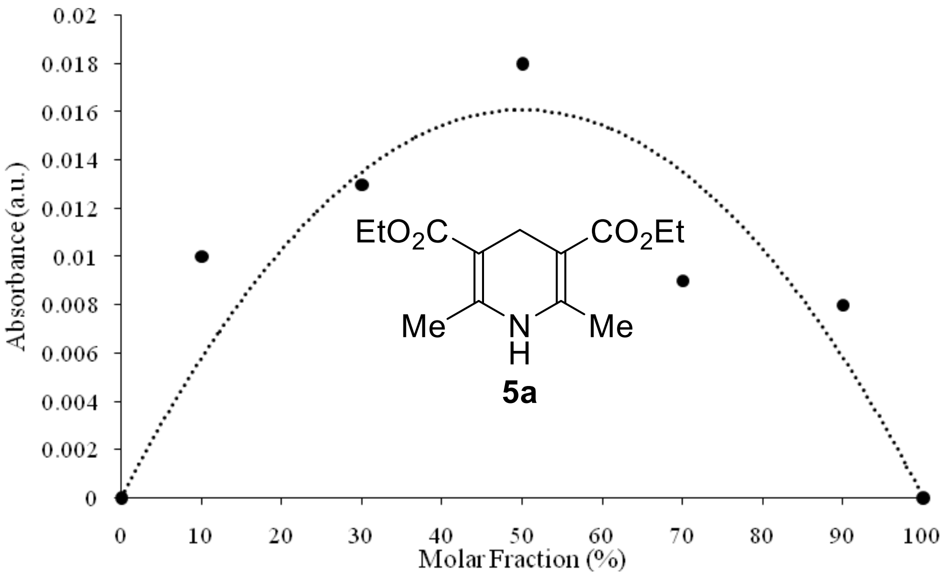

Figure S7: *Job's plots of the EDA complexes between H-DHP **5a**, imine **1a'*** and Cs_2CO_3

The Job's plot was constructed to evaluate the stoichiometry of the EDA complex between isopropyl Hantzsch ester derivative **5b** and imine **1a**' and base Cs_2CO_3 We measured the absorption of acetone solutions at 430 nm with different donor/base/acceptor ratios with constant concentration (0.01 M) of the three components. Initially we took the equimolar concentration of Hantzsch ester **5a** with base and gradually adding iminoester. All the absorption spectra were recorded in 1 cm path quartz cuvettes using Shimadzu UV-2600 UV-Vis Spectrophotometer. The absorbance values were plotted against the molar fraction (%) of Hantzsch ester derivative **5b**. The maximal absorbance at 50% molar fraction of **5b** indicated the 1:1:1 stoichiometry of the EDA complex in solution.

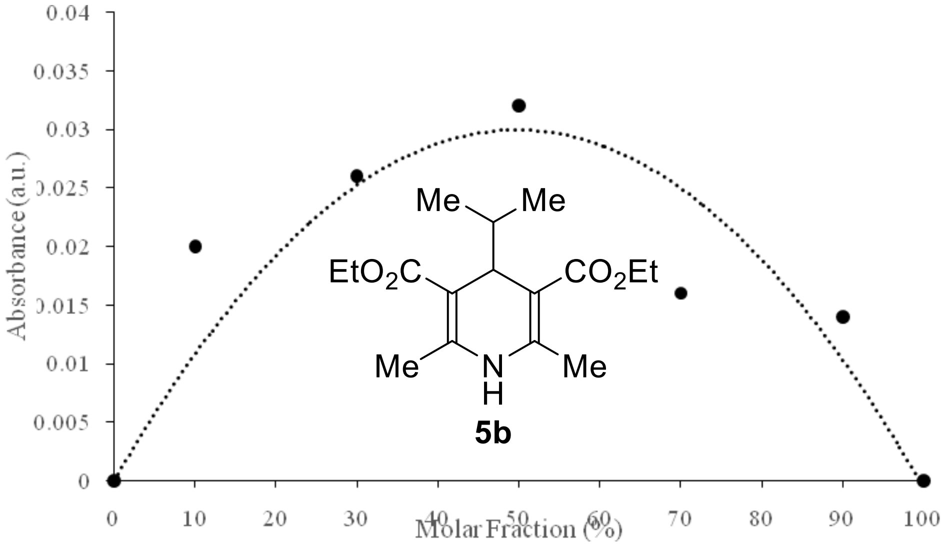

Figure S8: *Job's plots of the EDA complexes between alkyl-DHP **5b** and imine **1a**'* and Cs_2CO_3

5.4 Cyclic Voltammetry Study

Cyclic voltammograms were recorded for **1a'**, **5a**, and **5b**. This electrochemical cell contains a glassy carbon (disc-shaped with 3-mm diameter) as a working electrode, Pt wire as a counter electrode, and Ag wire as a pseudo-reference electrode. The glassy carbon working electrode was polished with 1.0 micron α-alumina polishing powder using a figure-eight motion. Electrolyte solution (0.1 M) was prepared from ACN and tetra-n-butylammonium hexafluorophosphate (Bu_4NPF_6). The solutions of **1a'**, **5a**, and **5b** (0.01 M) in ACN was degassed by nitrogen gas sparging for 10 minutes prior to measurements at room temperature at a scan rate of 50 mVs-1. The potential was externally calibrated against the ferrocene/ferrocenium couple (0.43 V) and the IUPAC convention was used for representing the cyclic voltammogram.

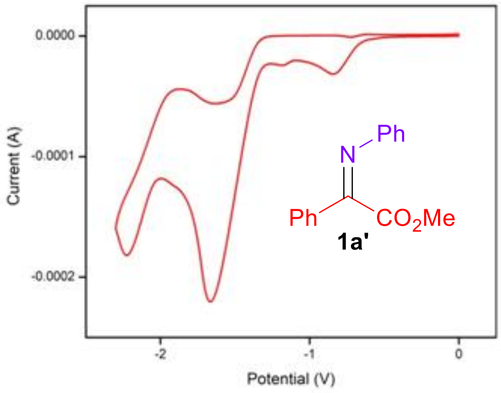

E_{red}(**1a'**) = -1.67 V vs. Ag wire

Figure S9: CV for **1a'**

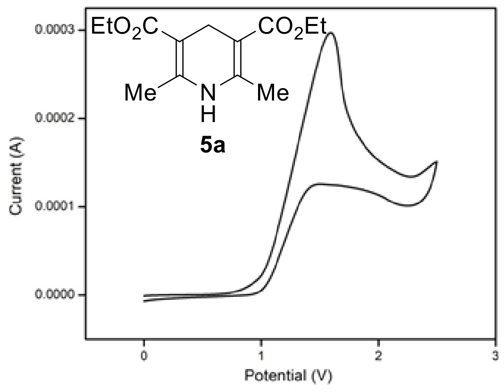

Figure S10: CV for **5a**

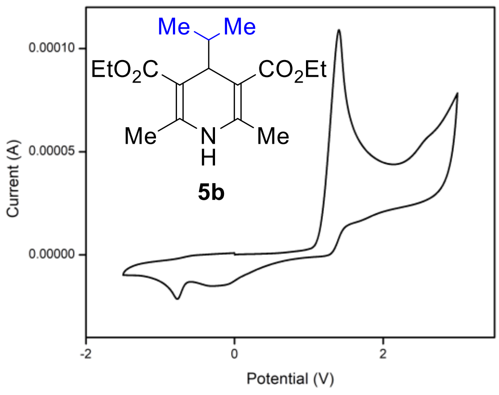

Figure S11: CV for **5b**

According to Rehm-Weller equation

$E(\mathbf{5a}^{\bullet+}/\mathbf{5a}^{*}) = E(\mathbf{5a}^{\bullet+}/\mathbf{5a}^{*}) - E(\mathbf{5a}^{*}/\mathbf{5a})$

$E(\mathbf{5a}^{\bullet+}/\mathbf{5a}^{*}) = 1.59\ \mathrm{V} - 3.50\ \mathrm{V}$

$= -1.91\ \mathrm{V}$ vs. Ag wire

$E_{red}(\mathbf{5a}^{*}) = -1.91\ \mathrm{V}$ vs. Ag wire

$E(\mathbf{5b}^{\bullet+}/\mathbf{5b}^{*}) = E(\mathbf{5b}^{\bullet+}/\mathbf{5b}^{*}) - E(\mathbf{5b}^{*}/\mathbf{5b})$

$E(\mathbf{5b}^{\bullet+}/\mathbf{5b}^{*}) = 1.35\ \mathrm{V} - 2.95\ \mathrm{V}$

$= -1.60\ \mathrm{V}$ vs. Ag wire

$E_{red}(\mathbf{5b}^{*}) = -1.60\ \mathrm{V}$ vs. Ag wire

5.5 NMR Experiments with DHPs

To investigate the interaction between the base and the N–H proton of the Hantzsch ester, we performed series of ^{1}**H NMR** experiments using different bases, including DBU and NaOAc. Significant changes in the chemical shift of the N–H proton were observed, indicating a notable interaction with the base. Due to solubility issues, cesium carbonate was not included in these studies.

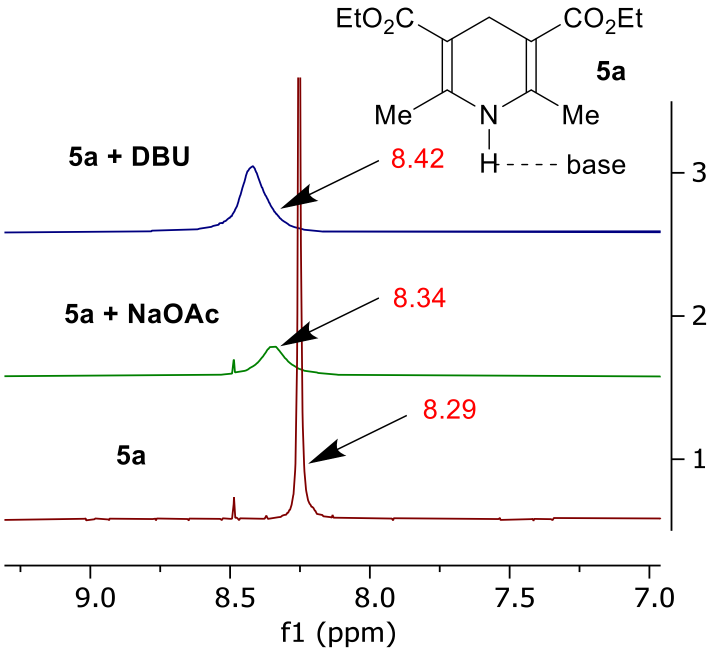

Figure S12: 1H-NMR Spectra (400 MHz, DMSO-d6)

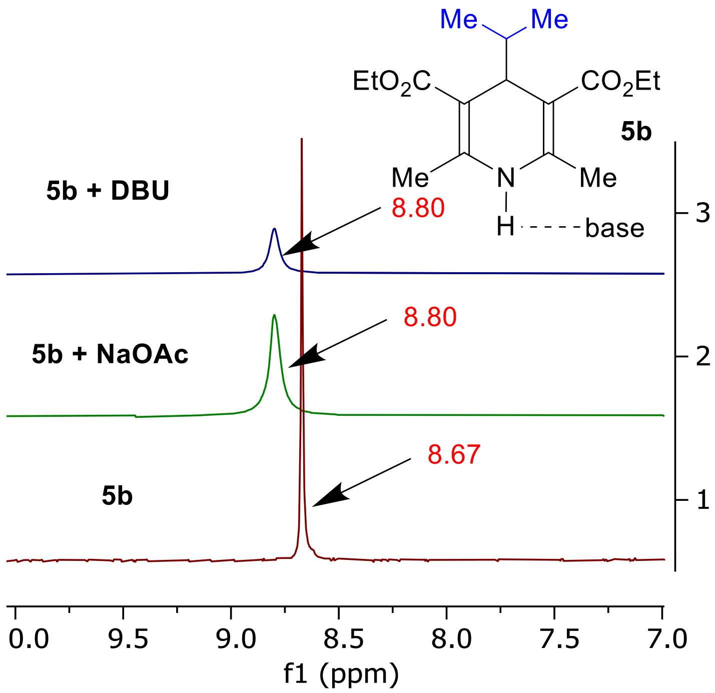

Figure S13: 1H-NMR Spectra (400 MHz, DMSO-d6)

5.6 Fluorescence Quenching Experiments (Stern Volmer Study)

Emission intensities were recorded using a Jasco fluorescence spectrophotometer. In a typical experiment, the samples were prepared separately in vials by taking iminoester (**1a'**) (1.0·10-4 M), DHPs **5a** and **5b** (1.0·10-4 M each) and 10.0 mg of Cs_2CO_3 (0.03 mmol) in acetone solvent. The excitation wavelength was fixed at 370 nm (bandwidth = 3 nm), while the emission spectra was acquired from 400 nm to 600 nm.

We performed a quenching experiment of 1,4-DHPs (**5a** and **5b**) (1.0•10-4 M) using Cs_2CO_3 only (no iminoester). We have observed a small static quenching in the presence of Cs_2CO_3 for both the cases.

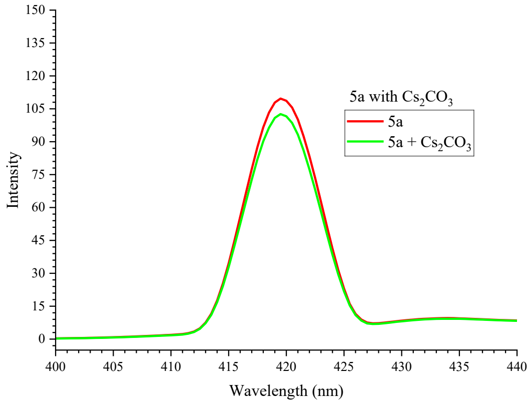

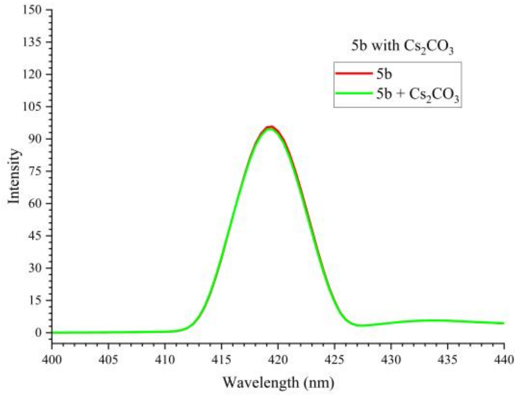

Figure S14: Fluorescence quenching of **5a** using Cs_2CO_3

Figure S15: Fluorescence quenching of **5b** using Cs_2CO_3

The samples were prepared mixing **5a**, **1a'** and Cs_2CO_3 in acetone in a capped 1.0 cm quartz cuvette. The sample was degassed with a stream of Argon for 10 min. For quenching we were added stock solution of iminoester **1a'** (0.0001, 0.0002, and 0.0003 M) in a total volume of 3 mL of acetone in capped 1.0 cm quartz cuvette.

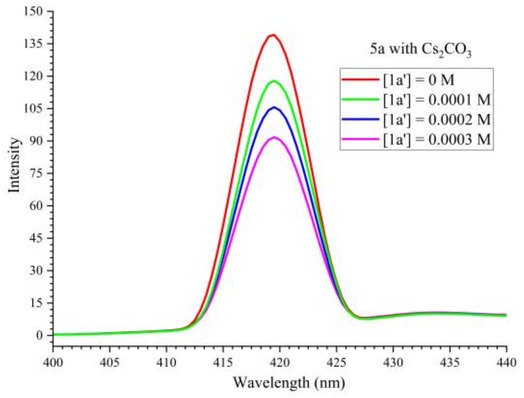

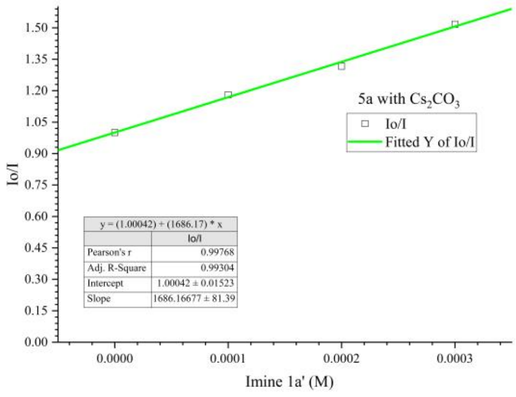

Figure S16:
Fluorescence quenching of **5a** with increasing concentratios of iminoester **1a'**

Figure S17:
Stern-Volmer Plot

The samples were prepared mixing **5a** in acetone in a capped 1.0 cm quartz cuvette. The sample was degassed with a stream of Argon for 10 min. For quenching, we added stock solution of iminoester **1a'** (0.0001, 0.0002, 0.0003M) in a total volume of 3 mL of acetone in capped 1.0 cm quartz cuvette.

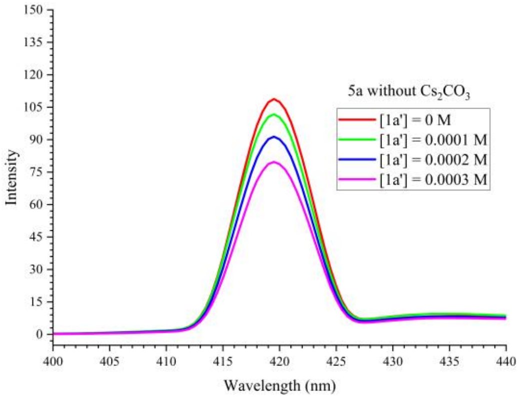

Figure S16:
Fluorescence quenching of **5a** with increasing concentratios of iminoester **1a’**

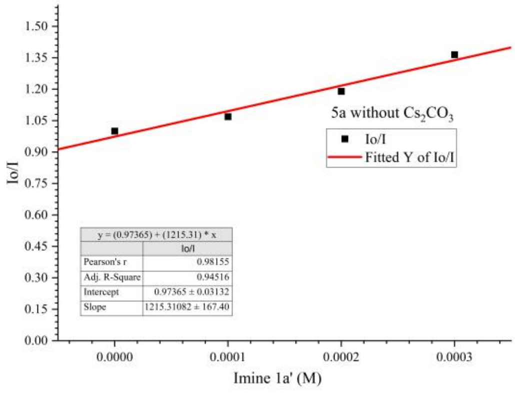

Figure S17:
Stern-Volmer Plot

From quenching experiments for both **5a**/Cs_2CO_3 and **5a**, we observed linear correlations between the amounts of **1a’** and the ratio I_o/I in Stern-Volmer plots. On the basis of the following equation, it is possible to calculate the Stern-Volmer constants K_{SV} (J. R. Lakowicz, Principles of Fluorescence Spectroscopy, chap. 3, pp. 52-93, Plenum Press, New York 1983):

$$I_o/I = 1 + K_{SV}[Q]$$

Where, I_o and I are the fluorescence intensities observed in the absence and presence, respectively, of quencher, [Q] is the quencher concentration and K_{SV} is the Stern-Volmer quenching constant.

K_{SV} for **5a**/Cs_2CO_3 = $1.6 * 10^3$; K_{SV} for **5a** = $1.2*10^3$

Stern-Volmer Plot

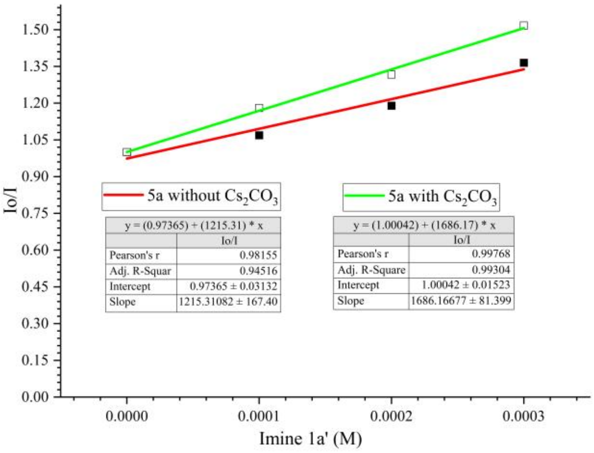

Figure S18: Stern-Volmer Plot

Similarly, the samples were prepared mixing **5b**, **1a’** and Cs_2CO_3 in acetone in a capped 1.0 cm quartz cuvette. The sample was degassed with a stream of Argon for 10 min. For quenching we were added stock solution of iminoester **1a’** (0.0001, 0.0002, and 0.0003 M) in a total volume of 3 mL of acetone in capped 1.0 cm quartz cuvette.

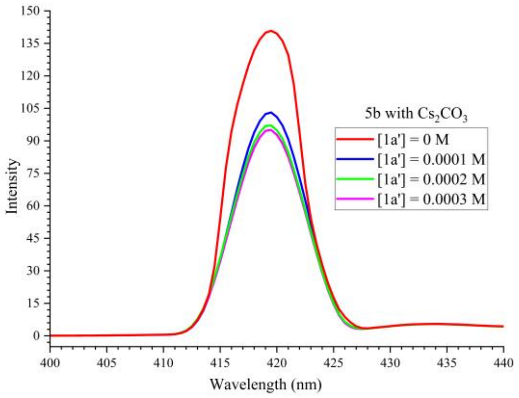

Figure S19:
Fluorescence quenching of **5b** with increasing concentratios of iminoester **1a’**

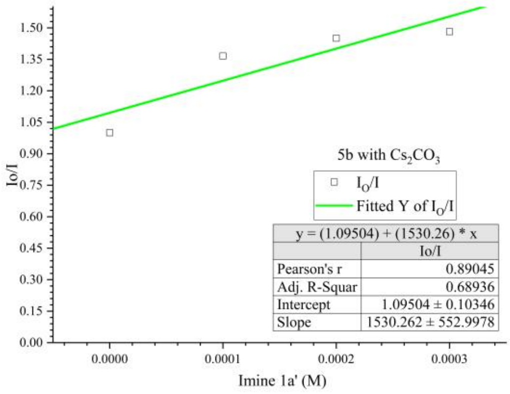

Figure S20:
Stern-Volmer Plot

The samples were prepared mixing **5b** in acetone in a capped 1.0 cm quartz cuvette. The sample was degassed with a stream of Argon for 10 min. For quenching, we added stock solution of iminoester **1a’** (0.0001, 0.0002, 0.0003M) in a total volume of 3 mL of acetone in capped 1.0 cm quartz cuvette.

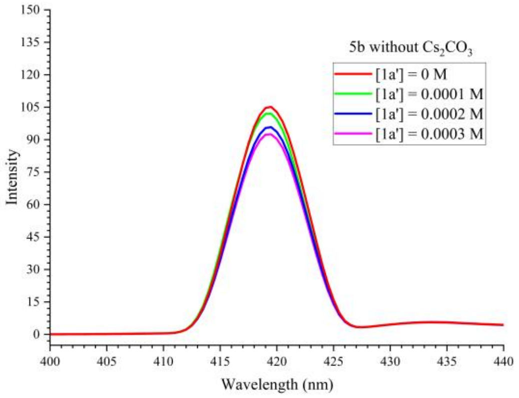

Figure S21:
Fluorescence quenching of **5b** with increasing concentrations of iminoester **1a’**

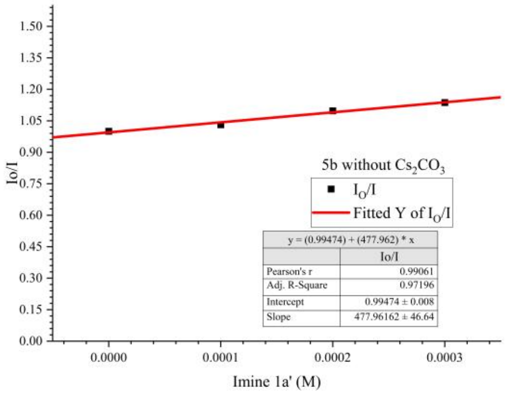

Figure S22:
Stern-Volmer Plot

From quenching experiments for both **5b**/Cs_2CO_3 and **5b**, we observed linear correlations between the amounts of **1a’** and the ratio I_o/I in Stern-Volmer plots. On the basis of the

following equation, it is possible to calculate the Stern-Volmer constants K_{SV} (J. R. Lakowicz, Principles of Fluorescence Spectroscopy, chap. 3, pp. 52-93, Plenum Press, New York 1983):

$$I_o/I = 1 + K_{SV}[Q]$$

Where, I_o and I are the fluorescence intensities observed in the absence and presence, respectively, of quencher, [Q] is the quencher concentration and K_{SV} is the Stern-Volmer quenching constant.

K_{SV} for **5b**/Cs_2CO_3 = 1.5 $*10^3$; K_{SV} for **5b** = 0.5$*10^3$

Stern-Volmer Plot

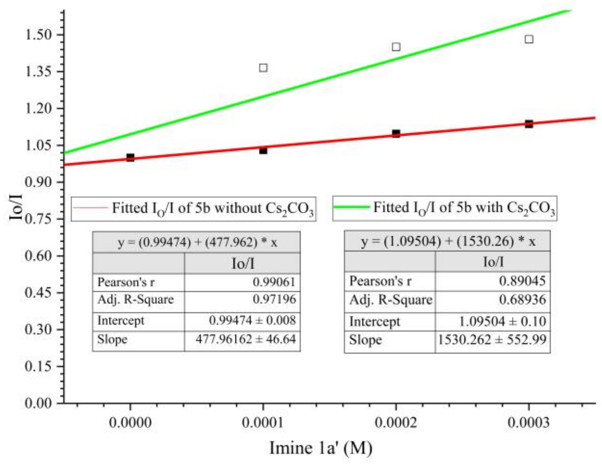

Figure S23: Stern-Volmer Plot

5.7 Measurement of Stokes Shift for DHPs

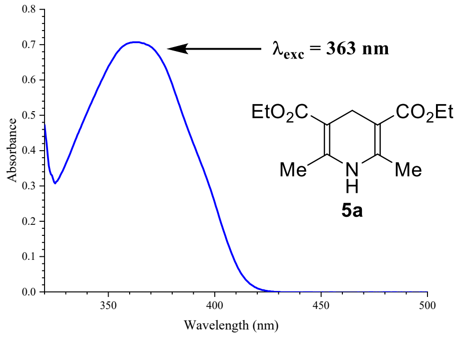

Figure S24: Absorption spectra of **5a**

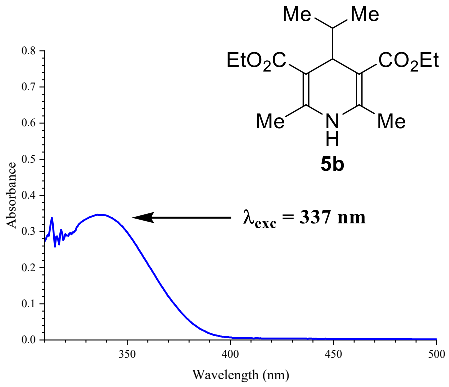

Figure S26: Absorption spectra of **5b**

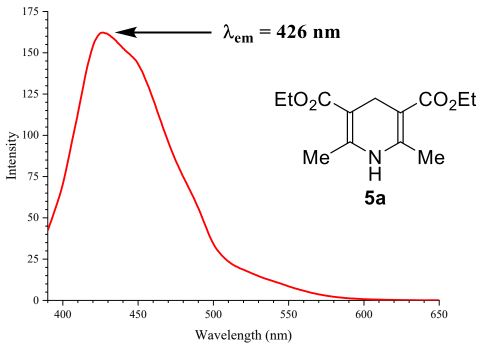

Figure S25: Emmision spectra of **5a**

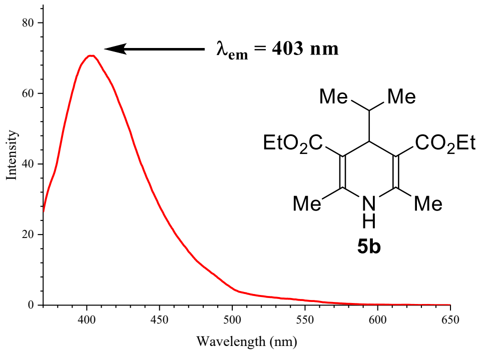

Figure S27: Emmision spectra of **5b**

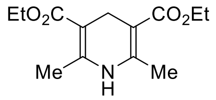

5a

Stokes Shift = $\lambda_{em} - \lambda_{exc}$ = (426 – 363) nm
= 63 nm

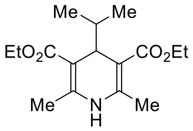

5b

Stokes Shift = $\lambda_{em} - \lambda_{exc}$ = (403 – 337) nm
= 66 nm

6.0 Experimental Details for the Substrate Scope

6.1. Methyl 5,5-difluoro-2,4-diphenyl-2-(phenylamino)pent-4-enoate (4a)

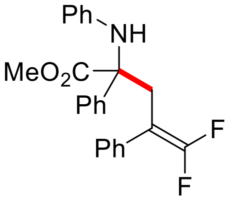

$C_{24}H_{21}F_2NO_2$
M (g/mol): 393.43

Following General Procedure **GP-4** for the title compound **4a** and by using **1a** (32.8 mg, 0.2 mmol, 1.0 equiv), **2a** (22.3 mg, 0.24 mmol, 1.2 equiv), *p*-TSA.H_2O (1.9 mg, 0.01 mmol, 0.05 equiv) and then hantzsch ester **5a** (50.5 mg, 0.2 mmol, 1.0 equiv), Cs_2CO_3 (97.5 mg, 0.3 mmol, 1.5 equiv), and 3a (105 µL, 0.6 mmol, 3.0 equiv) in Acetone for 16 h at 35 ºC. Purification was carried out by column chromatography (Hexane/EtOAc = 97:3) to afford **4a** as colourless oil (70.0 mg, 89%).

HRMS (ESI): m/z $[M+H]^+$ Calculated for $[C_{24}H_{22}F_2NO_2]^+$: 394.1619; Found: 394.1626.

^{1}H NMR (400 MHz, $CDCl_3$) δ 7.60–7.58 (m, 2H), 7.38–7.33 (m, 2H), 7.31–7.28 (m, 1H), 7.27–7.20 (m, 3H), 7.19–7.16 (m, 2H), 6.94–6.88 (m, 2H), 6.57 (t, *J* = 7.3 Hz, 1H), 6.14– 6.12(m, 2H), 5.14 (s, 1H), 3.77–3.67 (m, 2H), 3.26 (s, 3H).

^{13}C NMR (101 MHz, $CDCl_3$) δ 173.2, 154.7 (dd, J = 292.9, 288.8 Hz), 143.9, 140.2, 132.9 (dd, J = 5.0, 4.0 Hz), 128.9, 128.7, 128.2, 127.9, 127.5, 126.9, 117.1, 114.5,88.8 (dd, J = 19.7, 19.1 Hz), 65.7, 52.8, 31.8.

^{19}F NMR (376 MHz, $CDCl_3$) δ -89.02 (d, *J* = 34.7 Hz), -89.42 (d, *J* = 34.7 Hz).

6.2. Ethyl 5,5-difluoro-2,4-diphenyl-2-(phenylamino)pent-4-enoate (4b)

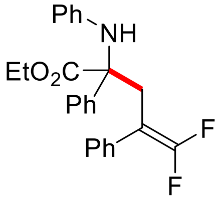

$C_{25}H_{23}F_2NO_2$
M (g/mol): 407.46

Following General Procedure **GP-4** for the title compound **4b** and by using **1b** (35.6 mg, 0.2 mmol, 1.0 equiv), **2a** (22.3 mg, 0.24 mmol, 1.2 equiv), *p*-TSA.H_2O (1.9 mg, 0.01 mmol, 0.05 equiv) and then hantzsch ester **5a** (50.5 mg, 0.2 mmol, 1.0 equiv), Cs_2CO_3 (97.5 mg, 0.3 mmol, 1.5 equiv), and **3a** (105 µL, 0.6 mmol, 3.0 equiv) in Acetone for 16 h at 35 ºC. Purification was carried out by column chromatography (Hexane/EtOAc = 97:3) to afford **4b** as colourless oil (70.0 mg, 86%).

HRMS (ESI): m/z $[M+H]^+$ Calculated for $[C_{25}H_{24}F_2NO_2]^+$: 408.1775; Found: 408.1781.

^{1}H NMR (400 MHz, $CDCl_3$) δ 7.58–7.56 (m, 2H), 7.35–7.31 (m, 2H), 7.28–7.16 (m, 4H), 7.14–7.12 (m, 2H), 6.91–6.86 (m, 2H), 6.55 (t, J = 7.3 Hz, 1H), 6.09 (d, J = 8.0 Hz, 2H), 5.08 (s, 1H), 3.89–3.54 (m, 4H), 0.98 (t, J = 7.1 Hz, 3H).

^{13}C NMR (101 MHz, $CDCl_3$) δ 172.7, 154.7 (dd, J = 292.9, 288.8 Hz), 143.9, 140.3, 132.9 (t, J = 3.0 Hz), 128.9, 128.8, 128.7, 128.2, 127.8, 127.5, 127.0, 117.1, 114.6, 88.9 (dd, J = 19.7, 19.1 Hz), 65.8, 62.2, 31.8, 13.6.

^{19}F NMR (376 MHz, $CDCl_3$) δ -89.11 (d, J = 34.7 Hz), -89.45 (d, J = 34.7 Hz).

6.3. Isopropyl 5,5-difluoro-2,4-diphenyl-2-(phenylamino)pent-4-enoate **(4c)**

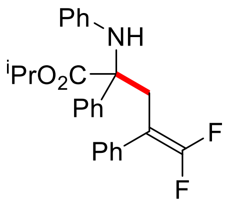

$C_{26}H_{25}F_2NO_2$
M (g/mol): 421.49

Following General Procedure **GP-4** for the title compound **4c** and by using **1c** (38.5 mg, 0.2 mmol, 1.0 equiv), **2a** (22.3 mg, 0.24 mmol, 1.2 equiv), *p*-TSA.H_2O (1.9 mg, 0.01 mmol, 0.05 equiv) and then hantzsch ester **5a** (50.5 mg, 0.2 mmol, 1.0 equiv), Cs_2CO_3 (97.5 mg, 0.3 mmol, 1.5 equiv), and **3a** (105 µL, 0.6 mmol, 3.0 equiv) in Acetone for 16 h at 35 °C. Purification was carried out by column chromatography (Hexane/EtOAc = 97:3) to afford **4c** as colourless oil (62.0 mg, 74%).

HRMS (ESI): m/z $[M+H]^+$ Calculated for $[C_{26}H_{26}F_2NO_2]^+$: 422.1926; Found: 422.1928.

^{1}H NMR (400 MHz, $CDCl_3$) δ 7.55–7.52 (m, 2H), 7.33–7.29 (m, 2H), 7.27–7.22 (m, 1H), 7.21–7.16 (m, 3H), 7.09–7.06 (m, 2H), 6.88–6.84 (m, 2H), 6.54–6.50 (m, 1H), 6.04–6.01 (m, 2H), 4.93 (s, 1H), 4.62 (hept, *J* = 6.1 Hz, 1H), 3.72–3.58 (m, 2H), 1.05 (d, *J* = 6.2 Hz, 3H), 0.85 (d, *J* = 6.3 Hz, 3H).

^{13}C NMR (101 MHz, $CDCl_3$) δ 172.2, 154.5 (dd, J = 292.9, 288.8 Hz), 144.0, 140.5, 133.1 (t, J = 3.0 Hz), 128.7, 128.6, 128.2, 127.6, 127.5, 126.9, 117.0, 114.5, 89.2 (dd, J = 19.7, 19.1 Hz), 70.2, 66.0, 31.6, 21.2, 21.0.

^{19}F NMR (376 MHz, $CDCl_3$) δ -88.74 (d, *J* = 34.7 Hz), -89.16 (d, *J* = 34.7 Hz).

6.4. benzyl 5,5-difluoro-2,4-diphenyl-2-(phenylamino)pent-4-enoate **(4d)**

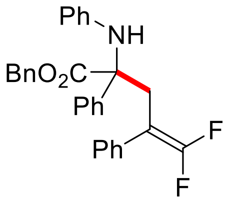

$C_{30}H_{25}F_2NO_2$
M (g/mol): 469.53

Following General Procedure **GP-4** for the title compound **4d** and by using **1d** (48.0 mg, 0.2 mmol, 1.0 equiv), **2a** (22.3 mg, 0.24 mmol, 1.2 equiv), *p*-TSA.H_2O (1.9 mg, 0.01 mmol, 0.05 equiv) and then hantzsch ester **5a** (50.5 mg, 0.2 mmol, 1.0 equiv), Cs_2CO_3 (97.5 mg, 0.3 mmol, 1.5 equiv), and **3a** (105 µL, 0.6 mmol, 3.0 equiv) in Acetone for 16 h at 35 °C. Purification was carried out by column chromatography (Hexane/EtOAc = 97:3) to afford **4d** as colourless oil (79.0 mg, 84%).

HRMS (ESI): m/z $[M+H]^+$ Calculated for $[C_{30}H_{26}F_2NO_2]^+$: 470.1926; Found: 470.1924

^{1}H NMR (400 MHz, CDCl$_3$) δ 7.64–7.61 (m, 2H), 7.39–7.31 (m, 3H), 7.30–7.21 (m, *J* = 3.4 Hz, 6H), 7.20–7.14 (m, 2H), 6.97–6.93 (m, 2H), 6.90–6.87 (m, 2H), 6.61 (t, *J* = 7.3 Hz, 1H), 6.18 (d, *J* = 8.0 Hz, 2H), 5.11 (s, 1H), 4.87 (d, *J* = 12.6 Hz, 1H), 4.47 (d, *J* = 12.6 Hz, 1H), 3.82–3.70 (m, 2H).

^{13}C NMR (101 MHz, CDCl$_3$) δ 172.4, 154.7 (t, J = 291.9 Hz), 143.9, 140.1, 135.1, 132.8 (t, J = 3.0 Hz), 128.9, 128.8, 128.7, 128.4, 128.2, 128.1, 128.0, 127.9, 127.6, 127.3, 127.0, 117.2, 117.1, 114.6, 114.5, 88.7 (dd, J = 19.7, 19.1 Hz), 67.2, 65.8, 32.1.

^{19}F NMR (376 MHz, CDCl$_3$) δ -89.11 (d, *J* = 34.7 Hz), -89.29 (d, *J* = 34.7 Hz).

6.5. Prop-2-yn-1-yl 5,5-difluoro-2,4-diphenyl-2-(phenylamino)pent-4-enoate **(4e)**

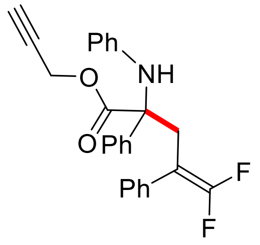

$C_{26}H_{21}F_2NO_2$
M (g/mol): 417.46

Following General Procedure **GP-4** for the title compound **4e** and by using **1e** (37.6 mg, 0.2 mmol, 1.0 equiv), **2a** (22.3 mg, 0.24 mmol, 1.2 equiv), *p*-TSA.H_2O (1.9 mg, 0.01 mmol, 0.05 equiv) and then hantzsch ester **5a** (50.5 mg, 0.2 mmol, 1.0 equiv), Cs_2CO_3 (97.5 mg, 0.3 mmol, 1.5 equiv), and **3a** (105 µL, 0.6 mmol, 3.0 equiv) in Acetone for 16 h at 35 ºC. Purification was carried out by column chromatography (Hexane/EtOAc = 97:3) to afford **4e** as colourless oil (65.0 mg, 78%).

HRMS (ESI): m/z $[M+H]^+$ Calculated for $[C_{26}H_{22}F_2NO_2]^+$: 418.1613; Found: 418.1619.

^{1}H NMR (400 MHz, CDCl$_3$) δ 7.60–7.58 (m, 2H), 7.37–7.32 (m, 2H), 7.30–7.19 (m, 4H), 7.18–7.14 (m, 2H), 6.93–6.88 (m, 2H), 6.57 (t, *J* = 7.3 Hz, 1H), 6.10 (d, *J* = 8.1 Hz, 2H), 4.96 (s, 1H), 4.27–4.08 (m, 2H), 3.77–3.64 (m, 2H), 2.40 (t, *J* = 2.4 Hz, 1H).

^{13}C NMR (101 MHz, CDCl$_3$) δ 172.0, 154.8 (dd, J = 292.9, 288.8 Hz), 143.8, 139.6, 132.7 (t, J = 3.0 Hz), 128.9, 128.7, 128.4, 128.0, 127.6, 127.0, 117.4, 114.7, 88.6 (dd, J = 19.7, 19.1 Hz), 76.7, 75.5, 65.9, 53.4, 32.2.

^{19}F NMR (376 MHz, CDCl$_3$) δ -88.65 (d, *J* = 34.7 Hz), -88.83 (d, *J* = 34.7 Hz).

6.6. But-3-en-1-yl 5,5-difluoro-2,4-diphenyl-2-(phenylamino)pent-4-enoate **(4f)**

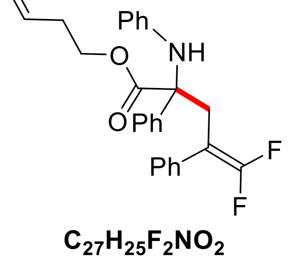

$C_{27}H_{25}F_2NO_2$
M (g/mol): 433.50

Following General Procedure **GP-4** for the title compound **4f** and by using **1f** (40.8 mg, 0.2 mmol, 1.0 equiv), **2a** (22.3 mg, 0.24 mmol, 1.2 equiv), *p*-TSA.H_2O (1.9 mg, 0.01 mmol, 0.05 equiv) and then hantzsch ester **5a** (50.5 mg, 0.2 mmol, 1.0 equiv), Cs_2CO_3 (97.5 mg, 0.3 mmol, 1.5 equiv), and **3a** (105 µL, 0.6 mmol, 3.0 equiv) in Acetone for 16 h at 35 °C. Purification was carried out by column chromatography (Hexane/EtOAc = 97:3) to afford **4f** as colourless oil (60.5 mg, 70%).

HRMS (ESI): m/z $[M+H]^+$ Calculated for $[C_{27}H_{26}F_2NO_2]^+$: 434.1932; Found: 434.1940.

^{1}H NMR (400 MHz, $CDCl_3$) δ 7.58–7.55 (m, 2H), 7.35–7.31 (m, 2H), 7.28–7.19 (m, 4H), 7.15–7.14 (m, 2H), 6.92–6.88 (m, 2H), 6.56 (t, *J* = 7.5 Hz, 1H), 6.12 (d, *J* = 8.0 Hz, 2H), 5.48–5.37 (m, 1H), 5.08 (s, 1H), 4.90–4.80 (m, 2H), 3.86–3.83 (m, 1H), 3.75–3.64 (m, 2H), 3.50–3.44 (m, 1H), 2.12–2.02 (m, 2H).

^{13}C NMR (101 MHz, $CDCl_3$) δ 172.6, 154.7 (dd, J = 292.9, 288.8 Hz), 143.9, 140.2, 133.4, 132.9 (t, J = 3.0 Hz), 128.9, 128.8, 128.7, 128.2, 127.8, 127.5, 127.0, 117.4, 117.1, 114.6, 88.8 (dd, J = 19.7, 19.1 Hz), 65.7, 64.9, 32.6, 31.9.

^{19}F NMR (376 MHz, $CDCl_3$) δ -88.65 δ -89.08 (d, *J* = 34.7 Hz), -89.29 (d, *J* = 34.7 Hz).

6.7. (3S,8S,9S,10R,13R,14S,16S)-10,13-dimethyl-16-((S)-6-methylheptan-2-yl)-2,3,4,7,8,9,10,11,12,13,14,15,16,17-tetradecahydro-1H-cyclopenta[a]phenanthren-3-yl 5,5-difluoro-2-((4-methoxyphenyl)amino)-2,4-diphenylpent-4-enoate **(4g)**

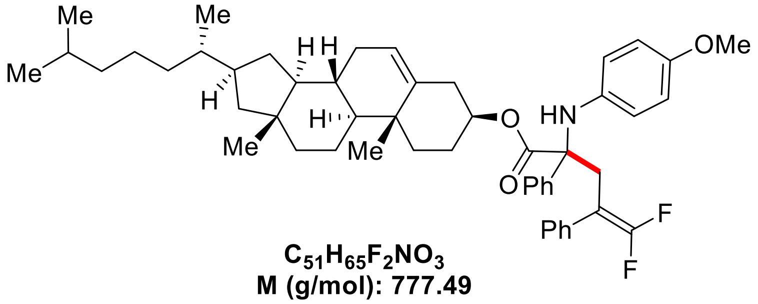

$C_{51}H_{65}F_2NO_3$
M (g/mol): 777.49

Following General Procedure **GP-4** for the title compound **4g** and by using **1h** (103.8 mg, 0.2 mmol, 1.0 equiv), **2a** (22.3 mg, 0.24 mmol, 1.2 equiv), *p*-TSA.H_2O (1.9 mg, 0.01 mmol, 0.05 equiv) and then hantzsch ester **5a** (50.5 mg, 0.2 mmol, 1.0 equiv), Cs_2CO_3 (97.5 mg, 0.3 mmol, 1.5 equiv), and **3a** (105 µL, 0.6 mmol, 3.0 equiv) in Acetone for 16 h at 35 °C. Purification was carried out by column chromatography (Hexane/EtOAc = 97:3) to afford **4g** as colourless oil (118.5 mg, 76%).

MS-MS (ESI): m/z $[M+H]^+$ Calculated for $[C_{51}H_{66}F_2NO_2]^+$: 778.50; Found: 778.5

^{1}H NMR (400 MHz, $CDCl_3$) δ 7.57–7.54 (m, 2H), 7.33–7.28 (m, 2H), 7.26–7.23 (d, 1H), 7.22–7.15 (m, 3H), 7.10–7.06 (m, 2H), 6.49–6.45 (m, 2H), 6.00–5.94 (m, 2H), 5.32–5.21 (m, 1H), 4.66–4.65 (m, 1H), 4.28–4.19 (m, 1H), 3.68–3.53 (m, 5H), 2.13–1.75 (m, 6H), 1.70–1.57 (m, 1H), 1.54–1.47 (m, 2H), 1.44–1.20 (m, 9H), 1.16–0.64 (m, 10H), 0.89 (m, 6H), 0.86 (d, *J* = 2.0 Hz, 3H), 0.85 (d, *J* = 1.9 Hz, 3H), 0.64 (s, 3H).

^{13}C NMR (101 MHz, $CDCl_3$) δ 172.2, 154.5 (dd, J = 292.9, 288.8 Hz), 151.6, 140.8, 139.5, 139.3, 138.2, 138.2, 133.2 (t, J = 3.0 Hz), 128.8, 128.6, 128.2, 128.2, 127.6, 127.5, 127.4, 127.0, 122.9, 115.5, 114.3, 89.1 (dd, J = 19.7, 19.1 Hz), 76.0, 66.3, 56.8, 56.2, 55.7, 50.0, 42.4, 39.8, 39.6, 37.4,

37.3, 36.9, 36.8, 36.6, 36.3, 35.9, 32.2, 32.0, 32.0, 31.9, 28.4, 28.1, 27.1, 27.0, 24.4, 23.9, 23.0, 22.7, 21.1, 19.4, 18.8, 12.0.

^{19}F NMR (376 MHz, CDCl$_3$) -88.78 (t, *J* = 33.8 Hz), -89.78 (t, *J* = 33.8 Hz).

6.8. Methyl 2-(4-bromophenyl)-5,5-difluoro-4-phenyl-2-(phenylamino)pent-4-enoate **(4h)**

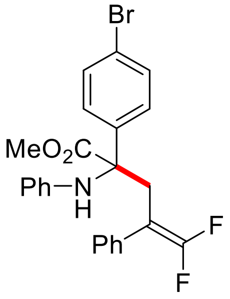

$C_{24}H_{20}BrF_2NO_2$
M (g/mol): 472.33.43

Following General Procedure **GP-4** for the title compound **4h** and by using **1i** (48.6 mg, 0.2 mmol, 1.0 equiv), **2a** (22.3 mg, 0.24 mmol, 1.2 equiv), *p*-TSA.H$_2$O (1.9 mg, 0.01 mmol, 0.05 equiv) and then hantzsch ester **5a** (50.5 mg, 0.2 mmol, 1.0 equiv), Cs$_2$CO$_3$ (97.5 mg, 0.3 mmol, 1.5 equiv), and **3a** (105 μL, 0.6 mmol, 3.0 equiv) in Acetone for 16 h at 35 ºC. Purification was carried out by column chromatography (Hexane/EtOAc = 97:3) to afford **4h** as colourless oil (84.0 mg, 89%).

HRMS (ESI): m/z [M+H]$^+$ Calculated for [$C_{25}H_{21}F_2NBrO_2$]$^+$: 472.0724; Found: 472.0731.

^{1}H NMR (400 MHz, CDCl$_3$) δ 7.47 (s, 4H), 7.27–7.18 (m, 3H), 7.16–7.13 (m, 2H), 6.95–6.91 (m, 2H), 6.62–6.58 (m, 1H), 6.13–6.11 (m, 2H), 5.07 (s, 1H), 3.69–3.62 (m, 2H), 3.28 (s, 3H).

^{13}C NMR (101 MHz, CDCl$_3$) δ 172.6, 154.7 (dd, J = 292.9, 288.8 Hz), 143.6, 139.3, 132.6 (dd, J = 5.0, 4.0 Hz), 131.9, 128.9, 128.8, 128.3, 127.6, 122.1, 117.5, 114.5, 88.5 (dd, J = 19.7, 19.1 Hz), 65.5, 52.9, 32.2.

^{19}F NMR (376 MHz, CDCl$_3$) δ -88.74 (d, *J* = 34.7 Hz), -89.16 (d, *J* = 34.7 Hz).

6.9. Methyl 5,5-difluoro-4-phenyl-2-(phenylamino)-2-(p-tolyl)pent-4-enoate **(4i)**

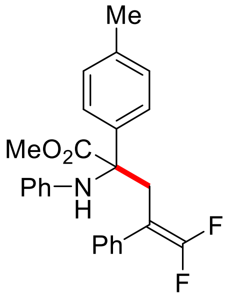

$C_{25}H_{23}F_2NO_2$
M (g/mol): 407.17

Following General Procedure **GP-4** for the title compound **4i** and by using **1j** (35.6 mg, 0.2 mmol, 1.0 equiv), **2a** (22.3 mg, 0.24 mmol, 1.2 equiv), *p*-TSA.H$_2$O (1.9 mg, 0.01 mmol, 0.05 equiv) and then hantzsch ester **5a** (50.5 mg, 0.2 mmol, 1.0 equiv), Cs$_2$CO$_3$ (97.5 mg, 0.3 mmol, 1.5 equiv), and **3a**

(105 μL, 0.6 mmol, 3.0 equiv) in Acetone for 16 h at 35 °C. Purification was carried out by column chromatography (Hexane/EtOAc = 97:3) to afford **4i** as colourless oil (63.5 mg, 78%).

HRMS (ESI): m/z $[M+H]^+$ Calculated for $[C_{26}H_{24}F_2NO_2]^+$: 408.1770; Found: 408.1774

^{1}H NMR (400 MHz, CDCl$_3$) δ 7.44–7.41 (m, 2H), 7.25–7.17 (m, 3H), 7.13–7.11 (m, 4H), 6.91–6.86 (m, 2H), 6.56–6.52 (m, 1H), 6.11–6.08 (m, 2H), 5.08 (s, 1H), 3.71–3.62 (m, 2H), 3.24 (s, 3H), 2.31 (s, 3H).

^{13}C NMR (101 MHz, CDCl$_3$) δ 173.4, 154.7 (dd, J = 292.9, 288.8 Hz), 144.0, 137.6, 137.2, 132.9 (t, J = 3.0 Hz), 129.6, 128.9, 128.7, 128.2, 127.5, 126.8, 117.1, 114.5, 88.8 (dd, J = 19.7, 19.1 Hz), 65.5, 52.8, 31.8, 21.2.

^{19}F NMR (376 MHz, CDCl$_3$) δ -90.13 (d, J = 34.8 Hz), -90.52 (d, J = 34.7 Hz).

6.10. Methyl 5,5-difluoro-2-(4-methoxyphenyl)-4-phenyl-2-(phenylamino)pent-4-enoate **(4j)**

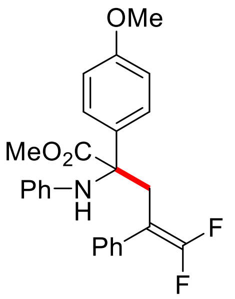

$C_{25}H_{23}F_2NO_2$
M (g/mol): 423.46

Following General Procedure **GP-4** for the title compound **4j** and by using **1k** (38.8 mg, 0.2 mmol, 1.0 equiv), **2a** (22.3 mg, 0.24 mmol, 1.2 equiv), *p*-TSA.H_2O (1.9 mg, 0.01 mmol, 0.05 equiv) and then hantzsch ester **5a** (50.5 mg, 0.2 mmol, 1.0 equiv), Cs_2CO_3 (97.5 mg, 0.3 mmol, 1.5 equiv), and **3a** (105 μL, 0.6 mmol, 3.0 equiv) in Acetone for 16 h at 35 °C. Purification was carried out by column chromatography (Hexane/EtOAc = 97:3) to afford **4j** as colourless oil (76.0 mg, 90%).

HRMS (ESI): m/z $[M+H]^+$ Calculated for $[C_{25}H_{24}F_2NO_3]^+$: 424.1724; Found: 424.1738

^{1}H NMR (400 MHz, CDCl$_3$) δ 7.58–7.56 (m, 2H), 7.35–7.31 (m, 2H), 7.29–7.25 (m, 1H), 7.06–7.03 (m, 2H), 6.90–6.86 (m, 2H), 6.76–6.73 (m, 2H), 6.56–6.52 (m, 1H), 6.09 (d, J = 8.0 Hz, 2H), 5.08 (s, 1H), 3.77 (s, 3H), 3.69–3.60 (m, 2H), 3.31 (s, 3H).

^{13}C NMR (101 MHz, CDCl$_3$) δ 173.3, 158.9, 154.7 (dd, J = 292.9, 288.8 Hz), 144.0, 140.3, 130.0, 128.9, 128.7, 127.9, 127.0, 124.9 (t, J = 3.0 Hz), 117.1, 114.6, 113.7, 88.2 (dd, J = 19.7, 19.1 Hz), 65.7, 55.4, 53.0, 31.9.

^{19}F NMR (376 MHz, CDCl$_3$) δ -89.79 (d, J = 34.7 Hz), -90.22 (d, J = 34.7 Hz).

6.11. Methyl 5,5-difluoro-4-phenyl-2-(phenylamino)-2-(thiophen-2-yl)pent-4-enoate **(4k)**

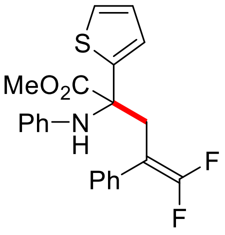

$C_{22}H_{19}F_2NO_2S$
M (g/mol): 399.46

Following General Procedure **GP-4** for the title compound **4k** and by using **1l** (34.0 mg, 0.2 mmol, 1.0 equiv), **2a** (22.3 mg, 0.24 mmol, 1.2 equiv), *p*-TSA.H_2O (1.9 mg, 0.01 mmol, 0.05 equiv) and then hantzsch ester **5a** (50.5 mg, 0.2 mmol, 1.0 equiv), Cs_2CO_3 (97.5 mg, 0.3 mmol, 1.5 equiv), and **3a** (105 µL, 0.6 mmol, 3.0 equiv) in Acetone for 16 h at 35 ºC. Purification was carried out by column chromatography (Hexane/EtOAc = 97:3) to afford **4k** as colourless oil (60.0 mg, 75%).

HRMS (ESI): m/z $[M+H]^+$ Calculated for $[C_{22}H_{20}F_2NO_2S]^+$: 400.1177; Found: 400.1166

^{1}H NMR (400 MHz, $CDCl_3$) δ 7.287–7.19 (m, 5H), 7.17–7.12 (m, 2H), 6.99–6.93 (m, 3H), 6.65–6.61 (m, 1H), 6.22– 6.20 (m, 2H), 5.06 (s, 1H), 3.71–3.66 (m, 1H), 3.55–3.51 (m, 1H), 3.33 (s, 3H).

^{13}C NMR (101 MHz, $CDCl_3$) δ 172.2, 155.0 (dd, J = 292.9, 288.8 Hz), 145.4, 143.8, 132.5 (t, J = 3.0 Hz), 128.8, 128.7, 128.3, 127.6, 127.2, 126.3, 125.7, 117.9, 114.9, 88.4 (dd, J = 19.7, 19.1 Hz), 64.6, 52.9, 34.6.

^{19}F NMR (376 MHz, $CDCl_3$) δ -88.56 (d, *J* = 34.7 Hz), -88.80 (d, *J* = 34.7 Hz).

6.12. Methyl 5,5-difluoro-4-phenyl-2-(phenylamino)-2-(4-(trifluoromethyl)phenyl)pent-4-enoate **(4l)**

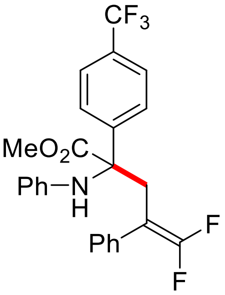

$C_{25}H_{20}F_5NO_2$
M (g/mol): 461.43

Following General Procedure **GP-4** for the title compound **4l** and by using **1m** (46.4 mg, 0.2 mmol, 1.0 equiv), **2a** (22.3 mg, 0.24 mmol, 1.2 equiv), *p*-TSA.H_2O (1.9 mg, 0.01 mmol, 0.05 equiv) and then hantzsch ester **5a** (50.5 mg, 0.2 mmol, 1.0 equiv), Cs_2CO_3 (97.5 mg, 0.3 mmol, 1.5 equiv), and **3a** (105 µL, 0.6 mmol, 3.0 equiv) in Acetone for 16 h at 35 ºC. Purification was carried out by column chromatography (Hexane/EtOAc = 97:3) to afford **4l** as colourless oil (61.0 mg, 85%).

HRMS (ESI): m/z $[M+H]^+$ Calculated for $[C_{25}H_{21}F_5NO_2]^+$: 462.1487; Found: 462.1491.

^{1}H NMR (400 MHz, $CDCl_3$) δ 7.73–7.71 (m, 2H), 7.60–7.58 (m, 2H), 7.27–7.18 (m, 3H), 7.16–7.13 (m, 2H), 6.95–6.91 (m, 2H), 6.62–6.58 (m, 1H), 6.10–6.08 (m, 2H), 5.09 (s, 1H), 3.74–3.65 (m, 2H), 3.29 (s, 3H).

^{13}C NMR (101 MHz, $CDCl_3$) δ 172.4, 154.8 (dd, J = 292.9, 288.8 Hz), 144.2, 143.9, 132.6 (dd, J = 5.0, 4.0 Hz), 130.1 (q, J = 30.3 Hz), 128.9, 128.8, 128.3, 127.7, 127.6, 125.8 (q, J = 4.74 Hz), 124.1 (q, J = 272.7 Hz), 117.7, 114.6, 88.4 ((dd, J = 19.7, 19.1 Hz), 65.7, 53.0, 32.5.

^{19}F NMR (376 MHz, $CDCl_3$) δ -62.40, -88.65 (d, *J* = 34.4 Hz), -89.08 (d, *J* = 34.4 Hz).

6.13. Methyl 2-((2-ethylphenyl)amino)-5,5-difluoro-2,4-diphenylpent-4-enoate **(4m)**

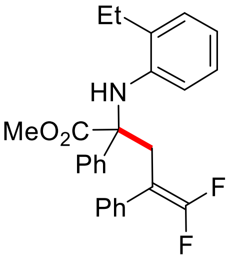

$C_{26}H_{25}F_2NO_2$
M (g/mol): 421.49

Following General Procedure **GP-4** for the title compound **4m** and by using **1a** (32.8 mg, 0.2 mmol, 1.0 equiv), **2b** (29.0 mg, 0.24 mmol, 1.2 equiv), *p*-TSA.H_2O (1.9 mg, 0.01 mmol, 0.05 equiv) and then hantzsch ester **5a** (50.5 mg, 0.2 mmol, 1.0 equiv), Cs_2CO_3 (97.5 mg, 0.3 mmol, 1.5 equiv), and **3a** (105 µL, 0.6 mmol, 3.0 equiv) in Acetone for 16 h at 35 °C. Purification was carried out by column chromatography (Hexane/EtOAc = 97:3) to afford **4m** as colourless oil (67.5 mg, 80%).

HRMS (ESI): m/z $[M+H]^+$ Calculated for $[C_{26}H_{26}F_2NO_2]^+$: 422.1926; Found: 422.1921.

^{1}H NMR (400 MHz, $CDCl_3$) δ 7.56–7.53 (m, 2H), 7.35–7.30 (m, 2H), 7.28–7.26 (m, 1H), 7.22–7.16 (m, 3H), 7.13–7.10 (m, 2H), 6.92 (dd, J = 7.5, 1.6 Hz, 1H), 6.72 (td, J = 7.7, 1.7 Hz, 1H), 6.54 (td, J = 7.3, 1.2 Hz, 1H), 5.90 (dd, J = 8.0, 1.1 Hz, 1H), 5.19 (s, 1H), 3.70–3.69 (m, 2H), 3.26 (s, 3H), 2.32–2.13 (m, 2H), 1.22 (t, J = 7.4 Hz, 3H).

^{13}C NMR (101 MHz, $CDCl_3$) δ 173.6, 154.9 (dd, J = 292.9, 288.8 Hz),141.2, 140.0, 132.7 (dd, J = 5.0, 4.0 Hz), 131.9, 128.8, 128.4, 128.1, 127.8, 127.4, 127.0, 125.9, 116.9, 112.6, 88.8 (dd, J = 19.7, 19.1 Hz), 65.8, 52.8, 31.8, 24.0, 12.7.

^{19}F NMR (376 MHz, $CDCl_3$) δ -88.80 (d, *J* = 34.7 Hz), -89.08 (d, *J* = 34.7 Hz).

6.14. Methyl 2-((3-bromophenyl)amino)-5,5-difluoro-2,4-diphenylpent-4-enoate **(4n)**

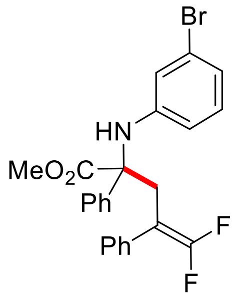

$C_{24}H_{20}BrF_2NO_2$
M (g/mol): 472.33

Following General Procedure **GP-4** for the title compound **4n** and by using **1a** (32.8 mg, 0.2 mmol, 1.0 equiv), **2c** (41.3 mg, 0.24 mmol, 1.2 equiv), *p*-TSA.H_2O (1.9 mg, 0.01 mmol, 0.05 equiv) and then hantzsch ester **5a** (50.5 mg, 0.2 mmol, 1.0 equiv), Cs_2CO_3 (97.5 mg, 0.3 mmol, 1.5 equiv), and **3a** (105 μL, 0.6 mmol, 3.0 equiv) in Acetone for 16 h at 35 ºC. Purification was carried out by column chromatography (Hexane/EtOAc = 97:3) to afford **4n** as colourless oil (79.0 mg, 84%).

HRMS (ESI): m/z $[M+H]^+$ Calculated for $[C_{24}H_{21}F_2NBrO_2]^+$: 472.0718; Found: 472.0706.

^{1}H NMR (400 MHz, $CDCl_3$) δ 7.54 (dd, *J* = 7.4, 2.0 Hz, 2H), 7.38–7.28 (m, 3H), 7.23–7.18 (m, 3H), 7.13–7.08 (m, 2H), 6.70–6.63 (m, 2H), 6.17 (t, *J* = 2.1 Hz, 1H), 5.96–5.91 (m, 1H), 5.20 (s, 1H), 3.76–3.61 (m, 2H), 3.33 (s, 3H).

^{13}C NMR (101 MHz, $CDCl_3$) δ 172.9, 154.7 (dd, J = 292.9, 288.8 Hz), 145.1, 139.6, 132.6 (t, J = 3.0 Hz), 129.8, 129.1, 128.7, 128.2, 128.1, 127.6, 126.7, 122.6, 119.9, 117.4, 112.5, 88.7 (dd, J = 19.7, 19.1 Hz), 65.7, 53.0, 31.2.

^{19}F NMR (376 MHz, $CDCl_3$) δ -88.77 (d, *J* = 34.7 Hz), -89.45 (d, *J* = 34.7 Hz).

6.15. Methyl 5,5-difluoro-2-((3-fluorophenyl)amino)-2,4-diphenylpent-4-enoate **(4o)**

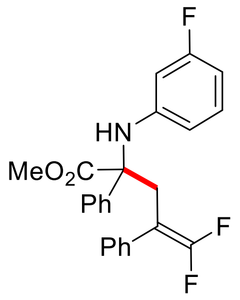

$C_{24}H_{20}F_3NO_2$
M (g/mol): 411.42

Following General Procedure **GP-4** for the title compound **40** and by using **1a** (32.8 mg, 0.2 mmol, 1.0 equiv), **2d** (26.6 mg, 0.24 mmol, 1.2 equiv), *p*-TSA.H_2O (1.9 mg, 0.01 mmol, 0.05 equiv) and then hantzsch ester **5a** (50.5 mg, 0.2 mmol, 1.0 equiv), Cs_2CO_3 (97.5 mg, 0.3 mmol, 1.5 equiv), and **3a** (105 μL, 0.6 mmol, 3.0 equiv) in Acetone for 16 h at 35 ºC. Purification was carried out by column chromatography (Hexane/EtOAc = 97:3) to afford **4o** as colourless oil (70.5 mg, 86%).

HRMS (ESI): m/z $[M+H]^+$ Calculated for $[C_{24}H_{21}F_3NO_2]^+$: 412.1519; Found: 412.1549.

^{1}H NMR (400 MHz, CDCl$_3$) δ 7.55–7.52 (m, 2H), 7.38–7.33 (m, 2H), 7.31–7.27 (m, 1H), 7.25 – 7.18 (m, 3H), 7.14 – 7.12 (m, 2H), 6.83–6.77 (m, 1H), 6.23 (td, *J* = 8.4, 2.5 Hz, 1H), 5.86 (dd, *J* = 8.2, 2.3 Hz, 1H), 5.72 (dt, *J* = 11.9, 2.3 Hz, 1H), 5.26 (s, 1H), 3.76 – 3.63 (m, 2H), 3.29 (s, 3H).

^{13}C NMR (101 MHz, CDCl$_3$) δ 173.0, 163.4 (dd, J = 292.9, 288.8 Hz), 154.7 (t, J = 291.9 Hz), 145.6 (d, J = 11.1 Hz), 139.7, 132.7 (t, J = 3.0 Hz), 129.7 (d, J = 10.1 Hz), 129.1, 128.8, 128.2, 128.1, 127.6, 126.8, 110.3, 103.6 (d, J = 21.2 Hz), 101.3 (d, J = 25.3 Hz), 88.7 (dd, J = 19.7, 19.1 Hz), 65.7, 53.0, 31.4

^{19}F NMR (376 MHz, CDCl$_3$) δ -88.80 (d, *J* = 34.7 Hz), -89.45 (d, *J* = 34.7 Hz), -113.37.

6.16. Methyl 5,5-difluoro-2-((4-methoxyphenyl)amino)-2,4-diphenylpent-4-enoate **(4p)**

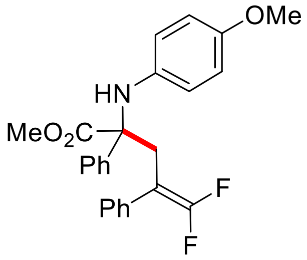

$C_{25}H_{23}F_2NO_3$
M (g/mol): 423.46

Following General Procedure **GP-4** for the title compound **4p** and by using **1a** (32.8 mg, 0.2 mmol, 1.0 equiv), **2e** (29.5 mg, 0.24 mmol, 1.2 equiv), *p*-TSA.H$_2$O (1.9 mg, 0.01 mmol, 0.05 equiv) and then hantzsch ester **5a** (50.5 mg, 0.2 mmol, 1.0 equiv), Cs$_2$CO$_3$ (97.5 mg, 0.3 mmol, 1.5 equiv), and **3a** (105 µL, 0.6 mmol, 3.0 equiv) in Acetone for 16 h at 35 °C. Purification was carried out by column chromatography (Hexane/EtOAc = 97:3) to afford **4p** as colourless oil (65.0 mg, 77%).

HRMS (ESI): m/z [M+H]$^+$ Calculated for [$C_{25}H_{24}F_2NO_3$]$^+$: 424.1719; Found: 424.1748.

^{1}H NMR (400 MHz, CDCl$_3$) δ 7.58–7.56 (m, 2H), 7.36–7.31 (m, 2H), 7.29–7.19 (m, 4H), 7.15–7.12 (m, 2H), 6.51–6.47(m, 2H), 6.05 –6.01 (m, 2H), 4.82 (s, 1H), 3.70– 3.59 (m, 5H), 3.26 (s, 3H).

^{13}C NMR (101 MHz, CDCl$_3$) δ 173.3, 154.7 (dd, J = 292.9, 288.8 Hz), 151.7, 140.5, 138.0, 132.9 (t, J = 3.0 Hz), 128.9, 128.8, 128.2, 127.8, 127.5, 127.0, 115.5, 114.4,88.8 (dd, J = 19.7, 19.1 Hz), 65.9, 55.7, 52.8, 32.1.

^{19}F NMR (376 MHz, CDCl$_3$) δ -89.11 (d, *J* = 34.7 Hz), -89.45 (d, *J* = 34.7 Hz).

6.17. Methyl 5,5-difluoro-2,4-diphenyl-2-((3-(trifluoromethoxy)phenyl)amino)pent-4-enoate **(4q)**

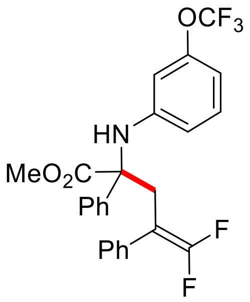

$C_{25}H_{20}F_5NO_3$
M (g/mol): 477.43

Following General Procedure **GP-4** for the title compound **4q** and by using **1a** (32.8 mg, 0.2 mmol, 1.0 equiv), **2f** (42.5 mg, 0.24 mmol, 1.2 equiv), *p*-TSA.H_2O (1.9 mg, 0.01 mmol, 0.05 equiv) and then hantzsch ester **5a** (50.5 mg, 0.2 mmol, 1.0 equiv), Cs_2CO_3 (97.5 mg, 0.3 mmol, 1.5 equiv), and **3a** (105 µL, 0.6 mmol, 3.0 equiv) in Acetone for 16 h at 35 ºC. Purification was carried out by column chromatography (Hexane/EtOAc = 97:3) to afford **4q** as colourless oil (81.0 mg, 85%).

HRMS (ESI): m/z $[M+H]^+$ Calculated for $[C_{26}H_{21}F_5NO_3]^+$: 478.1442; Found: 478.1437.

^{1}H NMR (400 MHz, $CDCl_3$) δ 7.55–7.53 (m, 2H), 7.38–7.2 (m, 3H), 7.23–7.17 (m, 3H), 7.12 (d, *J* = 6.9 Hz, 2H), 6.84 (t, *J* = 8.2 Hz, 1H), 6.37 (d, *J* = 8.2 Hz, 1H), 5.99 (dd, *J* = 8.3, 2.2 Hz, 1H), 5.85 (s, 1H), 5.31 (s, 1H), 3.78–3.62 (m, 2H), 3.34 (s, 3H).

^{13}C NMR (101 MHz, $CDCl_3$) δ 173.0, 154.7 (dd, J = 292.9, 288.8 Hz), 145.2, 139.5, 132.7 (dd, J = 5.0, 4.0 Hz), 129.6, 129.1, 128.7, 128.2, 127.6, 120.5 (q, J = 258.5 Hz), 112.8, 109.0, 106.6, 88.7 (dd, J = 19.7, 19.1 Hz), 65.7, 53.1, 31.2.

^{19}F NMR (376 MHz, $CDCl_3$) δ -57.57, -88.85 (d, *J* = 34.7 Hz), -89.57 (d, *J* = 34.7 Hz).

6.18. Methyl 2-((3,5-dimethoxyphenyl)amino)-5,5-difluoro-2,4-diphenylpent-4-enoate **(4r)**

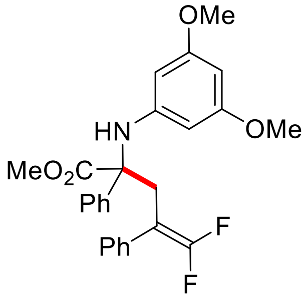

$C_{26}H_{25}F_2NO_4$
M (g/mol): 453.49

Following General Procedure **GP-4** for the title compound **4r** and by using **1a** (32.8 mg, 0.2 mmol, 1.0 equiv), **2g** (36.7 mg, 0.24 mmol, 1.2 equiv), *p*-TSA.H_2O (1.9 mg, 0.01 mmol, 0.05 equiv) and then hantzsch ester **5a** (50.5 mg, 0.2 mmol, 1.0 equiv), Cs_2CO_3 (97.5 mg, 0.3 mmol, 1.5 equiv), and **3a** (105 µL, 0.6 mmol, 3.0 equiv) in Acetone for 16 h at 35 ºC. Purification was carried out by column chromatography (Hexane/EtOAc = 97:3) to afford **4r** as colourless oil (80.0 mg, 88%).

HRMS (ESI): m/z $[M+H]^+$ Calculated for $[C_{26}H_{26}F_2NO_4]^+$: 454.1830; Found: 454.1814.

^{1}H NMR (400 MHz, CDCl$_3$) δ 7.56–7.53(m, 2H), 7.36–7.31 (m, 2H), 7.28–7.24 (m, 1H), 7.24–7.17 (m, 3H), 7.14–7.11 (m, 2H), 5.71 (t, J = 2.2 Hz, 1H), 5.21 (d, J = 2.2 Hz, 2H), 5.07 (s, 1H), 3.71–3.69 (m, 2H), 3.53 (s, 6H), 3.32 (s, 3H).

^{13}C NMR (101 MHz, CDCl$_3$) δ 173.2, 160.9, 154.7 (dd, J = 292.9, 288.8 Hz), 145.6, 140.3, 132.8 (t, J = 3.0 Hz), 129.0, 128.8, 128.2, 128.0, 127.3, 126.8, 93.4, 90.0, 88.8 (dd, J = 19.7, 19.1 Hz), 65.8, 55.0, 53.0, 31.3.

^{19}F NMR (376 MHz, CDCl$_3$) δ -88.99 (d, J = 34.7 Hz), -89.46 (d, J = 34.7 Hz).

6.19. Methyl 5,5-difluoro-2,4-diphenyl-2-(p-tolylamino)pent-4-enoate **(4s)**

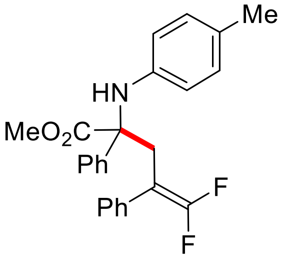

$C_{25}H_{23}F_2NO_2$
M (g/mol): 407.46

Following General Procedure **GP-4** for the title compound **4s** and by using **1a** (32.8 mg, 0.2 mmol, 1.0 equiv), **2h** (25.7 mg, 0.24 mmol, 1.2 equiv), *p*-TSA.H_2O (1.9 mg, 0.01 mmol, 0.05 equiv) and then hantzsch ester **5a** (50.5 mg, 0.2 mmol, 1.0 equiv), Cs_2CO_3 (97.5 mg, 0.3 mmol, 1.5 equiv), and **3a** (105 µL, 0.6 mmol, 3.0 equiv) in Acetone for 16 h at 35 ºC. Purification was carried out by column chromatography (Hexane/EtOAc = 97:3) to afford **4s** as colourless oil (69.0 mg, 85%).

HRMS (ESI): m/z $[M+H]^+$ Calculated for $[C_{25}H_{24}F_2NO_2]^+$: 408.1775; Found: 408.1763.

^{1}H NMR (400 MHz, CDCl$_3$) δ 7.59 (d, J = 7.6 Hz, 2H), 7.36–7.33 (m, 2H), 7.30–7.21 (m, 4H), 7.18–7.16 (m, 2H), 6.73 (d, J = 8.2 Hz, 2H), 6.06–6.04 (m, 2H), 4.99 (s, 1H), 3.74–3.65 (m, 2H), 3.24 (s, 3H), 2.15 (s, 3H).

^{13}C NMR (101 MHz, CDCl$_3$) δ 173.2,154.8 (dd, J = 292.9, 288.8 Hz), 141.5, 140.4, 132.9 (t, J = 3.0 Hz), 129.2, 128.9, 128.8, 128.2, 127.8, 127.5, 127.0, 126.2, 114.6, 88.8 (dd, J = 19.7, 19.1 Hz), 65.7, 52.7, 32.2, 20.4.

^{19}F NMR (376 MHz, CDCl$_3$) δ -89.06 (d, J = 34.7 Hz), -89.32 (d, J = 34.7 Hz).

6.20. Methyl 2-((4-chlorophenyl)amino)-5,5-difluoro-2,4-diphenylpent-4-enoate **(4t)**

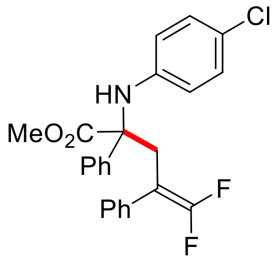

$C_{24}H_{20}F_2NO_2$
M (g/mol): 427.88

Following General Procedure **GP-4** for the title compound **4t** and by using **1a** (32.8 mg, 0.2 mmol, 1.0 equiv), **2i** (30.6 mg, 0.24 mmol, 1.2 equiv), *p*-TSA.H_2O (1.9 mg, 0.01 mmol, 0.05 equiv) and then hantzsch ester **5a** (50.5 mg, 0.2 mmol, 1.0 equiv), Cs_2CO_3 (97.5 mg, 0.3 mmol, 1.5 equiv), and **3a** (105 µL, 0.6 mmol, 3.0 equiv) in Acetone for 16 h at 35 °C. Purification was carried out by column chromatography (Hexane/EtOAc = 97:3) to afford **4t** as colourless oil (75.5 mg, 88%).

HRMS (ESI): m/z $[M+H]^+$ Calculated for $[C_{24}H_{21}F_2NO_2]^+$: 428.1223; Found: 428.1229.

^{1}H NMR (400 MHz, $CDCl_3$) δ 7.52 (d, *J* = 8.7 Hz, 2H), 7.31 (d, *J* = 8.7 Hz, 2H), 7.22 (dd, *J* = 10.8, 7.1 Hz, 3H), 7.14 (d, *J* = 7.3 Hz, 2H), 6.92 (t, *J* = 8.0 Hz, 2H), 6.59 (t, *J* = 7.3 Hz, 1H), 6.10 (d, *J* = 8.2 Hz, 2H), 5.06 (s, 1H), 3.68–3.61 (m, 2H), 3.27 (s, 3H).

^{13}C NMR (101 MHz, $CDCl_3$) δ 172.7, 154.7 (dd, J = 292.9, 288.8 Hz), 143.6, 138.7, 133.8, 132.6 (t, J = 3.0 Hz), 129.0, 128.8, 128.5, 128.3, 127.6, 117.5, 114.5, 88.5 (dd, J = 19.7, 19.1 Hz), 65.4, 52.9, 32.2.

^{19}F NMR (376 MHz, $CDCl_3$) δ -88.77 (d, *J* = 34.7 Hz), -89.20 (d, *J* = 34.7 Hz).

6.21. Methyl 2-((4-bromophenyl)amino)-5,5-difluoro-2,4-diphenylpent-4-enoate **(4u)**

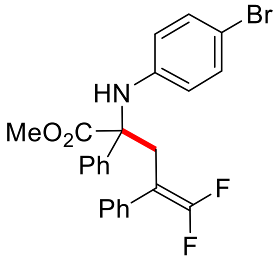

$C_{24}H_{20}F_2NO_2$
M (g/mol): 472.33

Following General Procedure **GP-4** for the title compound **4u** and by using **1a** (32.8 mg, 0.2 mmol, 1.0 equiv), **2j** (41.3 mg, 0.24 mmol, 1.2 equiv), *p*-TSA.H_2O (1.9 mg, 0.01 mmol, 0.05 equiv) and then hantzsch ester **5a** (50.5 mg, 0.2 mmol, 1.0 equiv), Cs_2CO_3 (97.5 mg, 0.3 mmol, 1.5 equiv), and **3a** (105 µL, 0.6 mmol, 3.0 equiv) in Acetone for 16 h at 35 °C. Purification was carried out by column chromatography (Hexane/EtOAc = 97:3) to afford **4u** as colourless oil (85.0 mg, 90%).

HRMS (ESI): m/z $[M+H]^+$ Calculated for $[C_{24}H_{21}F_2NO_2]^+$: .472.0718; Found: 472.0713.

^{1}H NMR (400 MHz, CDCl$_3$) δ 7.53 (d, *J* = 7.5 Hz, 2H), 7.37–7.33 (m, 2H), 7.30 –7.27 (m, 1H), 7.26 –7.18 (m, 3H), 7.13 (d, *J* = 7.6 Hz, 2H), 6.99 – 6.92 (m, 2H), 5.99 – 5.91 (m, 2H), 5.19 (s, 1H),3.65–3.58 (m, 2H), 3.28 (s, 3H).

^{13}C NMR (101 MHz, CDCl$_3$) δ 173.0, 154.8 (dd, J = 292.9, 288.8 Hz), 142.9, 139.6, 132.7 (dd, J = 5.0, 4.0 Hz), 131.4, 129.0, 128.8, 128.3, 128.1, 127.6, 126.8, 116.0, 109.0, 88.7 (dd, J = 19.7, 19.1 Hz), 65.7, 52.9, 31.5.

^{19}F NMR (376 MHz, CDCl$_3$) δ -88.77 (d, *J* = 34.7 Hz), -89.23 (d, *J* = 34.7 Hz).

6.22. Methyl 5,5-difluoro-2-((4-isopropylphenyl)amino)-2,4-diphenylpent-4-enoate **(4v)**

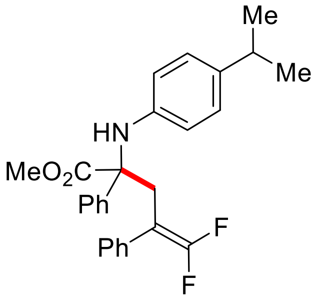

$C_{27}H_{27}F_2NO_2$
M (g/mol): 435.51

Following General Procedure **GP-4** for the title compound **4v** and by using **1a** (32.8 mg, 0.2 mmol, 1.0 equiv), **2k** (32.4 mg, 0.24 mmol, 1.2 equiv), *p*-TSA.H$_2$O (1.9 mg, 0.01 mmol, 0.05 equiv) and then hantzsch ester **5a** (50.5 mg, 0.2 mmol, 1.0 equiv), Cs$_2$CO$_3$ (97.5 mg, 0.3 mmol, 1.5 equiv), and **3a** (105 μL, 0.6 mmol, 3.0 equiv) in Acetone for 16 h at 35 ºC. Purification was carried out by column chromatography (Hexane/EtOAc = 97:3) to afford **4v** as colourless oil (69.5 mg, 80%).

HRMS (ESI): m/z $[M+H]^+$ Calculated for $[C_{27}H_{28}F_2NO_2]^+$: 436.2083; Found: 436.2064.

^{1}H NMR (400 MHz, CDCl$_3$) δ 7.63–7.59 (m, 2H), 7.39–7.28 (m, 3H), 7.24–7.18 (m, 3H), 7.15 – 7.12 (m, 2H), 6.78–6.73 (m, 2H), 6.05–6.01 (m, 2H), 5.01 (s, 1H), 3.75–3.67 (m, 2H), 3.32 (s, 3H), 2.71 (hept, *J* = 6.7 Hz, 1H), 1.16 (d, *J* = 6.4 Hz, 6H).

^{13}C NMR (101 MHz, CDCl$_3$) δ 173.3, 154.7 (dd, J = 292.9, 288.8 Hz), 141.7, 140.6, 137.6, 132.8 (t, J = 3.0 Hz), 128.8, 127.8, 127.4, 127.0, 126.7, 126.5, 126.2, 114.5, 88.8 (dd, J = 19.7, 19.1 Hz), 65.9, 52.8, 33.1, 31.8, 24.4, 24.3.

^{19}F NMR (376 MHz, CDCl$_3$) δ -89.14 (d, *J* = 34.7 Hz), -89.54 (d, *J* = 34.7 Hz).

6.23. Methyl 4-(4-(tert-butyl)phenyl)-5,5-difluoro-2-phenyl-2-(phenylamino)pent-4-enoate **(4w)**

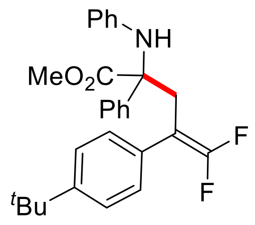

$C_{28}H_{29}F_2NO_2$
M (g/mol): 449.54

Following General Procedure **GP-4** for the title compound **4w** and by using **1a** (32.8 mg, 0.2 mmol, 1.0 equiv), **2a** (22.3 mg, 0.24 mmol, 1.2 equiv), *p*-TSA.H_2O (1.9 mg, 0.01 mmol, 0.05 equiv) and then hantzsch ester **5a** (50.5 mg, 0.2 mmol, 1.0 equiv), Cs_2CO_3 (97.5 mg, 0.3 mmol, 1.5 equiv), and **3g** (137 µL, 0.6 mmol, 3.0 equiv) in Acetone for 16 h at 35 °C. Purification was carried out by column chromatography (Hexane/EtOAc = 97:3) to afford **4w** as colourless oil (82.5 mg, 92%).

HRMS (ESI): m/z $[M+H]^+$ Calculated for $[C_{28}H_{30}F_2NO_2]^+$: 450.2239; Found: 450.2244.

^{1}H NMR (400 MHz, $CDCl_3$) δ 7.59–7.56 (m, 2H), 7.36–7.32 (m, 2H), 7.30–7.26 (m, 1H), 7.24–7.21 (m, 2H), 7.06–7.04 (m, 2H), 6.89–6.85 (m, 2H), 6.53 (t, *J* = 7.3 Hz, 1H), 6.06–6.04 (m, 2H), 5.07 (s, 1H), 3.72–3.62 (m, 2H), 3.27 (s, 3H), 1.29 (s, 9H).

^{13}C NMR (101 MHz, $CDCl_3$) δ 173.2, 154.7 (dd, J = 292.9, 288.8 Hz), 150.2, 143.9, 140.2, 129.8 (t, J = 3.0 Hz), 128.9, 128.6, 128.4, 127.8, 127.0, 125.1, 117.0, 114.5, 88.5 (dd, J = 19.7, 19.1 Hz), 65.8, 52.8, 34.6, 31.6, 31.4.

^{19}F NMR (376 MHz, $CDCl_3$) δ -89.33 (d, *J* = 34.7 Hz), -89.76 (d, *J* = 34.7 Hz).

6.24. Methyl 5,5-difluoro-4-(4-methoxyphenyl)-2-phenyl-2-(phenylamino)pent-4-enoate **(4x)**

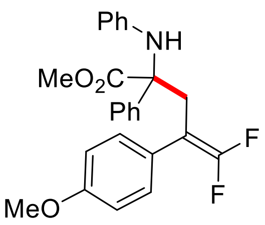

$C_{25}H_{23}F_2NO_3$
M (g/mol): 423.46

Following General Procedure **GP-4** for the title compound **4x** and by using **1a** (32.8 mg, 0.2 mmol, 1.0 equiv), **2a** (22.3 mg, 0.24 mmol, 1.2 equiv), *p*-TSA.H_2O (1.9 mg, 0.01 mmol, 0.05 equiv) and then hantzsch ester **5a** (50.5 mg, 0.2 mmol, 1.0 equiv), Cs_2CO_3 (97.5 mg, 0.3 mmol, 1.5 equiv), and **3a** (121 µL, 0.6 mmol, 3.0 equiv) in Acetone for 16 h at 35 °C. Purification was carried out by column chromatography (Hexane/EtOAc = 97:3) to afford **4x** as colourless oil (70.0 mg, 83%).

HRMS (ESI): m/z $[M+H]^+$ Calculated for $[C_{26}H_{24}F_2NO_3]^+$: 424.1724; Found: 424.1738.

^{1}H NMR (400 MHz, CDCl$_3$) δ 7.58–7.53 (m, 2H), 7.35–7.31 (m, 2H), 7.29–7.25 (m, 1H), 7.06–7.03 (m, 2H), 6.90–6.86 (m, 2H), 6.76–6.73 (m, 2H), 6.56–6.52 (m, 1H), 6.09 (d, *J* = 8.0 Hz, 2H), 5.08 (s, 1H), 3.77 (s, 3H), 3.69–3.60 (m, 2H), 3.31 (s, 3H).

^{13}C NMR (101 MHz, CDCl$_3$) δ 173.3, 157.6 (dd, J = 292.9, 288.8 Hz), 151.8, 144.0, 140.3, 129.9, 128.9, 128.7, 127.9, 127.0, 124.9 (t, J = 3.0 Hz), 117.1, 114.6, 113.7, 88.2 (dd, J = 19.7, 19.1 Hz), 65.7, 55.4, 52.9, 31.8.

^{19}F NMR (376 MHz, CDCl$_3$) δ -89.32 (d, *J* = 34.7 Hz), -89.76 (d, *J* = 34.7 Hz).

6.25. Methyl 4-(4-benzylphenyl)-5,5-difluoro-2-phenyl-2-(phenylamino)pent-4-enoate **(4y)**

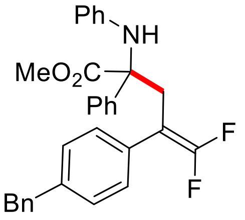

$C_{31}H_{27}F_2NO_2$
M (g/mol): 483.56

Following General Procedure **GP-4** for the title compound **4y** and by using **1a** (32.8 mg, 0.2 mmol, 1.0 equiv), **2a** (22.3 mg, 0.24 mmol, 1.2 equiv), *p*-TSA.H_2O (1.9 mg, 0.01 mmol, 0.05 equiv) and then hantzsch ester **5a** (50.5 mg, 0.2 mmol, 1.0 equiv), Cs_2CO_3 (97.5 mg, 0.3 mmol, 1.5 equiv), and **3c** (157 µL, 0.6 mmol, 3.0 equiv) in Acetone for 16 h at 35 °C. Purification was carried out by column chromatography (Hexane/EtOAc = 97:3) to afford **4y** as colourless oil (73.5 mg, 76%).

HRMS (ESI): m/z $[M+H]^+$ Calculated for $[C_{31}H_{28}F_2NO_2]^+$: 484.2083; Found: 484.2078.

^{1}H NMR (400 MHz, CDCl$_3$) δ 7.58 (d, *J* = 7.6 Hz, 2H), 7.46–7.38 (m, 4H), 7.37–7.32 (m, 3H), 7.30–7.26 (m, 1H), 7.06 (d, *J* = 8.6 Hz, 2H), 6.90 (t, *J* = 7.8 Hz, 2H), 6.85–6.82 (m, 2H), 6.56 (t, *J* = 7.2 Hz, 1H), 6.11 (d, *J* = 8.2 Hz, 2H), 5.11 (s, 1H), 5.05 (s, 2H), 3.70–3.62 (m, 2H), 3.26 (s, 3H).

^{13}C NMR (101 MHz, CDCl$_3$) δ 173.2, 154.7 (dd, J = 292.9, 288.8 Hz), 151.8, 144.0, 140.3, 137.0, 130.0, 128.9, 128.7, 128.2, 127.9, 127.6, 126.9, 125.2 (t, J = 3.0 Hz), 117.1, 114.7, 114.5, 88.2 (dd, J = 19.7, 19.1 Hz), 70.0, 65.7, 52.9, 31.8.

^{19}F NMR (376 MHz, CDCl$_3$) δ -89.66 (d, *J* = 34.7 Hz), -90.09 (d, *J* = 34.7 Hz).

6.26. Methyl 4-(4-bromophenyl)-5,5-difluoro-2-phenyl-2-(phenylamino)pent-4-enoate **(4z)**

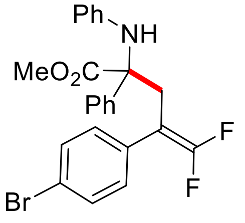

$C_{24}H_{20}F_2NO_2$
M (g/mol): 472.33

Following General Procedure **GP-4** for the title compound **4z** and by using **1a** (32.8 mg, 0.2 mmol, 1.0 equiv), **2a** (22.3 mg, 0.24 mmol, 1.2 equiv), *p*-TSA.H_2O (1.9 mg, 0.01 mmol, 0.05 equiv) and then hantzsch ester **5a** (50.5 mg, 0.2 mmol, 1.0 equiv), Cs_2CO_3 (97.5 mg, 0.3 mmol, 1.5 equiv), and **3d** (151 µL, 0.6 mmol, 3.0 equiv) in Acetone for 16 h at 35 °C. Purification was carried out by column chromatography (Hexane/EtOAc = 97:3) to afford **4z** as colourless oil (80.0 mg, 85%).

HRMS (ESI): m/z $[M+H]^+$ Calculated for $[C_{24}H_{21}F_2NO_2]^+$: 472.0718; Found: 472.0713.

^{1}H NMR (400 MHz, $CDCl_3$) δ 7.56–7.53 (m, 2H), 7.36–7.31 (m, 2H), 7.30–7.24 (m, 3H), 6.94–6.90 (m, 2H), 6.88–6.83 (m, 2H), 6.56–6.52 (m, 1H), 6.00–5.97 (m, 2H), 5.03 (s, 1H), 3.72–3.61 (m, 2H), 3.40 (s, 3H).

^{13}C NMR **(101 MHz, $CDCl_3$)** δ 173.2,154.5 (t, J = 291.9 Hz), 143.6, 140.0, 131.8 (t, J = 3.0 Hz), 131.2, 130.2, 129.0, 128.7, 128.0, 126.8, 121.4, 117.2, 114.3, 88.3 (dd, J = 19.7, 19.1 Hz), 77.5, 77.2, 76.9, 65.8, 53.1, 31.0.

^{19}F NMR (376 MHz, $CDCl_3$) δ -88.34 (d, *J* = 34.7 Hz), -88.89 (d, *J* = 34.7 Hz).

6.27. Methyl 4-(3,5-dichlorophenyl)-5,5-difluoro-2-phenyl-2-(phenylamino)pent-4-enoate **(4aa)**

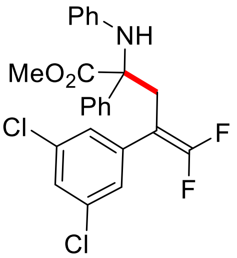

$C_{24}H_{19}Cl_2F_2NO_2$
M (g/mol): 462.32

Following General Procedure **GP-4** for the title compound **4aa** and by using **1a** (32.8 mg, 0.2 mmol, 1.0 equiv), **2a** (22.3 mg, 0.24 mmol, 1.2 equiv), *p*-TSA.H_2O (1.9 mg, 0.01 mmol, 0.05 equiv) and then hantzsch ester **5a** (50.5 mg, 0.2 mmol, 1.0 equiv), Cs_2CO_3 (97.5 mg, 0.3 mmol, 1.5 equiv), and **3f** (145 µL, 0.6 mmol, 3.0 equiv) in Acetone for 16 h at 35 °C. Purification was carried out by column chromatography (Hexane/EtOAc = 97:3) to afford **4aa** as colourless oil (71.0 mg, 77%).

HRMS (ESI): m/z $[M+H]^+$ Calculated for $[C_{24}H_{20}Cl_2F_2NO_2]^+$: 462.0834; Found: 462.0829.

^{1}H NMR (400 MHz, $CDCl_3$) δ 7.55–7.52 (m, 2H), 7.37–7.32 (m, 2H), 7.31–7.26 (m, 1H), 7.10 (t, *J* = 2.0 Hz, 1H), 6.95–6.94 (m, 2H), 6.90–6.85 (m, 2H), 6.55–6.52 (m, 1H), 6.04–6.01 (m, 2H), 5.10 (s, 1H), 3.76–3.60 (m, 2H), 3.51 (s, 3H).

^{13}C NMR (101 MHz, $CDCl_3$) δ 173.3, 154.8 (dd, J = 292.9, 288.8 Hz), 151.8, 143.3, 139.8, 135.9 (dd, J = 5.0, 4.0 Hz), 134.7, 129.0, 128.8, 128.1, 127.4, 127.2, 126.8, 117.7, 114.2, 87.8 (dd, J = 19.7, 19.1 Hz), 65.7, 53.3, 31.1.

^{19}F NMR (376 MHz, $CDCl_3$) δ -86.74 (d, *J* = 34.7 Hz), -87.62 (d, *J* = 34.7 Hz).

6.28. Methyl 3-methyl-2-phenyl-2-(phenylamino)butanoate (**6a**)

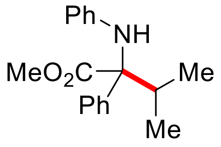

$C_{18}H_{21}NO_2$
M (g/mol): 283.37

Following General Procedure **GP-5** for the title compound **6a** and by using **1a** (32.8 mg, 0.2 mmol, 1.0 equiv), **2a** (22.3 mg, 0.24 mmol, 1.2 equiv), *p*-TSA.H_2O (1.9 mg, 0.01 mmol, 0.05 equiv) and then DHP **5b** (118.2 mg, 0.4 mmol, 2.0 equiv) and Cs_2CO_3 (130.0 mg, 0.4 mmol, 2.0 equiv), in Acetone for 16 h at 35 ºC. Purification was carried out by column chromatography (Hexane/EtOAc = 99:1) to afford **6a** as colourless oil (49.0 mg, 86%).

HRMS (ESI): m/z $[M+H]^+$ Calculated for $[C_{18}H_{22}NO_2]^+$: 284.1651; Found: 284.1648.

^{1}H NMR (400 MHz, $CDCl_3$) δ 7.67–7.64 (m, 2H), 7.36–7.27 (m, 3H), 7.04–6.99 (m, 2H), 6.66 (t, *J* = 7.3 Hz, 1H), 6.39 (d, *J* = 8.0 Hz, 2H), 4.50 (br s, 1H) 3.62 (s, 3H), 2.56 (hept, *J* = 6.8 Hz, 1H), 0.93 (d, *J* = 6.7 Hz, 3H), 0.85 (d, *J* = 6.8 Hz, 3H).

^{13}C NMR (101 MHz, $CDCl_3$) δ 174.0, 145.8, 136.3, 129.2, 128.7, 127.6, 127.3, 118.2, 115.5, 70.8, 52.4, 38.5, 18.4, 17.6.

6.29. Benzyl 3-methyl-2-phenyl-2-(phenylamino)butanoate (**6b**)

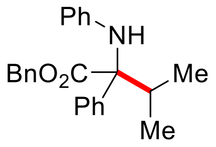

$C_{24}H_{25}NO_2$
M (g/mol): 359.47

Following General Procedure **GP-5** for the title compound **6b** and by using **1d** (48.0 mg, 0.2 mmol, 1.0 equiv), **2a** (22.3 mg, 0.24 mmol, 1.2 equiv), *p*-TSA.H_2O (1.9 mg, 0.01 mmol, 0.05 equiv) and then DHP **5b** (118.2 mg, 0.4 mmol, 2.0 equiv) and Cs_2CO_3 (130.0 mg, 0.4 mmol, 2.0 equiv), in Acetone for 16 h at 35 ºC. Purification was carried out by column chromatography (Hexane/EtOAc = 99:1) to afford **6b** as colourless oil (65.0 mg, 90%).

HRMS (ESI): m/z $[M+H]^+$ Calculated for $[C_{24}H_{26}NO_2]^+$ 360.1964; Found: 360.1927.
^{1}H NMR (400 MHz, $CDCl_3$) δ 7.77–7.73 (m, 2H), 7.39–7.30 (m, 3H), 7.29–7.22 (m, 3H), 7.07–7.02 (m, 4H), 6.71 (t, *J* = 7.3 Hz, 1H), 6.43 (d, *J* = 8.0 Hz, 2H), 5.11–5.02 (m, 2H), 4.46 (s, 1H), 2.57 (hept, *J* = 6.8 Hz, 1H), 0.95 (d, *J* = 6.8 Hz, 3H), 0.85 (d, *J* = 6.8 Hz, 3H).

^{13}C NMR (101 MHz, $CDCl_3$) δ 173.4, 146.3, 136.2, 135.6, 129.3, 128.8, 128.5, 128.2, 128.1, 127.6, 127.3, 118.2, 115.4, 70.6, 67.1, 38.8, 18.3, 17.5.

6.30. Isopropyl 3-methyl-2-phenyl-2-(phenylamino)butanoate (**6c**)

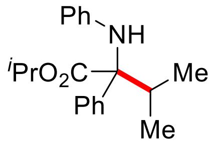

$C_{20}H_{25}NO_2$
M (g/mol): 311.43

Following General Procedure **GP-5** for the title compound **6c** and by using **1c** (38.5 mg, 0.2 mmol, 1.0 equiv), **2a** (22.3 mg, 0.24 mmol, 1.2 equiv), *p*-TSA.H_2O (1.9 mg, 0.01 mmol, 0.05 equiv) and then DHP **5b** (118.2 mg, 0.4 mmol, 2.0 equiv) and Cs_2CO_3 (130.0 mg, 0.4 mmol, 2.0 equiv), in Acetone for 16 h at 35 °C. Purification was carried out by column chromatography (Hexane/EtOAc = 99:1) to afford **6c** as colourless oil (47.0 mg, 75%).

HRMS (ESI): m/z $[M+H]^+$ Calculated for $[C_{20}H_{26}NO_2]^+$: 312.1964; Found: 312.1956.

^{1}H NMR (400 MHz, $CDCl_3$) δ 7.71–7.68 (m, 2H), 7.34–7.27 (m, 3H), 7.02–6.96 (m, 2H), 6.66–6.61 (m, 1H), 6.41 – 6.37 (m, 2H), 4.93 (hept, *J* = 6.2 Hz, 1H), 4.35 (s, 1H), 2.51 (hept, *J* = 7.0 Hz, 1H), 1.14 (d, *J* = 6.1 Hz, 3H), 0.93 (d, *J* = 6.8 Hz, 3H), 0.85–0.82 (m, 6H).

^{13}C NMR (101 MHz, $CDCl_3$) δ 172.1, 145.9, 135.9, 128.7, 128.0, 126.8, 126.5, 117.4, 114.9, 70.0, 68.0, 38.0, 21.1, 20.7, 17.7, 16.8.

6.31. Prop-2-yn-1-yl 3-methyl-2-phenyl-2-(phenylamino)butanoate (**6d**)

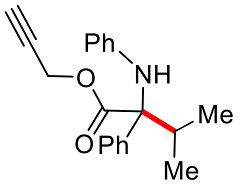

$C_{20}H_{21}NO_2$
M (g/mol): 307.39

Following General Procedure **GP-5** for the title compound **6d** and by using **1e** (37.6 mg, 0.2 mmol, 1.0 equiv), **2a** (22.3 mg, 0.24 mmol, 1.2 equiv), *p*-TSA.H_2O (1.9 mg, 0.01 mmol, 0.05 equiv) and then DHP **5b** (118.2 mg, 0.4 mmol, 2.0 equiv) and Cs_2CO_3 (130.0 mg, 0.4 mmol, 2.0 equiv), in Acetone for 16 h at 35 °C. Purification was carried out by column chromatography (Hexane/EtOAc = 99:2) to afford **6d** as colourless oil (37.0 mg, 60%).
HRMS (ESI): m/z $[M+H]^+$ Calculated for $[C_{20}H_{22}NO_2]^+$:308.1651; Found:. 308.1647.

^{1}H NMR (400 MHz, $CDCl_3$) δ 7.68–7.63 (m, 2H), 7.35–7.27 (m, 3H), 7.03–6.96 (m, 2H), 6.68–6.64 (m, 1H), 6.40 (d, *J* = 8.1 Hz, 2H), 4.70–4.65 (m, 1H), 4.55–4.50 (m, 1H), 2.58 (heptet, *J* = 6.8 Hz, 1H), 2.38–2.35 (m, 1H), 0.96 (d, *J* = 6.8 Hz, 3H), 0.84 (d, *J* = 6.8 Hz, 3H).

^{13}C NMR (101 MHz, $CDCl_3$) δ 172.5, 145.3, 135.6, 130.2, 129.3, 129.0, 128.7, 128.7, 127.7, 127.6, 127.5, 118.8, 116.0, 75.0, 52.6, 38.6, 18.3, 17.5.

6.32. But-3-en-1-yl 3-methyl-2-phenyl-2-(phenylamino)butanoate (**6e**)

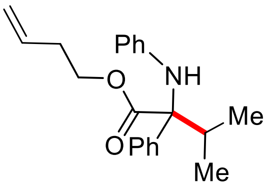

$C_{21}H_{25}NO_2$
M (g/mol): 323.44

Following General Procedure **GP-5** for the title compound **6e** and by using **1f** (40.8 mg, 0.2 mmol, 1.0 equiv), **2a** (22.3 mg, 0.24 mmol, 1.2 equiv), *p*-TSA.H_2O (1.9 mg, 0.01 mmol, 0.05 equiv) and then DHP **5b** (118.2 mg, 0.4 mmol, 2.0 equiv) and Cs_2CO_3 (130.0 mg, 0.4 mmol, 2.0 equiv), in Acetone for 16 h at 35 ºC. Purification was carried out by column chromatography (Hexane/EtOAc = 99:1) to afford **6e** as colourless oil (58.0 mg, 90%).
HRMS (ESI): m/z $[M+H]^+$ Calculated for $[C_{21}H_{26}NO_2]^+$:324.1964; Found:. 324.1968.

^{1}H NMR (400 MHz, $CDCl_3$) δ 7.70–7.66 (m, 2H), 7.03–6.98 (m, 3H), 7.06–6.96 (m, 2H), 6.67–6.63 (m, 1H), 6.39– 6.37 (m, 2H), 5.59–5.49 (m, 1H), 4.98–4.92 (m, 2H), 4.39 (s, 1H), 4.12–3.98 (m, 2H), 2.53 (hept, *J* = 6.8 Hz, 1H), 2.20–2.12 (m, 1H), 0.93 (d, *J* = 6.7 Hz, 3H), 0.84 (d, *J* = 6.9 Hz, 3H).

^{13}C NMR (101 MHz, $CDCl_3$) δ 173.5, 146.3, 136.3, 134.0, 129.3, 128.7, 127.5, 127.2, 118.1, 117.2, 115.4, 70.7, 64.4, 38.7, 32.9, 18.4, 17.5.

6.33. (1*S*,2*R*,5*S*)-2-Isopropyl-5-methylcyclohexyl 3-methyl-2-phenyl-2(phenylamino)butanoate **(6f)**

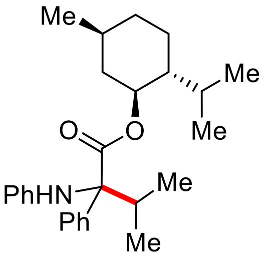

$C_{27}H_{37}NO_2$
M (g/mol): 407.60

Following General Procedure **GP-5** for the title compound **6f** and by using **1f** (57.6 mg, 0.2 mmol, 1.0 equiv), **2a** (22.3 mg, 0.24 mmol, 1.2 equiv), *p*-TSA.H_2O (1.9 mg, 0.01 mmol, 0.05 equiv) and then DHP **5b** (118.2 mg, 0.4 mmol, 2.0 equiv) and Cs_2CO_3 (130.0 mg, 0.4 mmol, 2.0 equiv), in Acetone for 16 h at 35 ºC. Purification was carried out by column chromatography (Hexane/EtOAc = 99:1) to afford **6f** as colourless oil (66.0 mg, 81%). Isolated as 70:30 diastereomeric mixture.

HRMS (ESI): m/z $[M+H]^+$ Calculated for $[C_{27}H_{38}NO_2]^+$: 408.2903; Found:. 408.2913.

^{1}H NMR (400 MHz, $CDCl_3$) δ 7.72–7.65 (m, 2.56H), 7.33–7.26 (m, 3.87H), 7.00–6.94 (m, 2.58H), 6.63–6.59 (m, 1.27H), 6.36 (d, *J* = 8.1 Hz, 2.58H), 4.63– 4.53(m, 1.70H), 4.36 (s, 0.97H), 2.62 (hept, *J* = 6.8 Hz, 0.42H), 2.46 (hept, *J* = 6.8 Hz, 1H), 2.03–1.98 (m, 1.05H), 1.65–1.58 (m, 1.55H), 1.55–1.52 (m, 0.87H), 1.51–1.40 (m, 1.80H), 1.23–1.16 (m, 1.66H), 1.14–1.03 (m, 1.44H), 0.98–0.92 (m, 5.48H), 0.90–0.82 (m, 6.54H), 0.81–0.74 (m, 5.28H), 0.73–0.69 (m, 1.54H), 0.64–0.67 (m, 4.24H), 0.23 (d, *J* = 6.9 Hz, 2.88H).

^{13}C NMR (101 MHz, CDCl$_3$) δ 173.2, 172.9, 146.5, 146.2, 136.5, 135.6, 129.6, 129.3, 128.8, 128.6, 127.6, 127.3, 127.1, 127.1, 118.1, 117.9, 115.6, 115.2, 75.5, 75.1, 70.8, 70.4, 46.9, 46.8, 40.7, 39.9, 38.8, 37.6, 34.3, 34.2, 31.5, 31.2, 25.6, 25.0, 22.8, 22.7, 22.2, 22.0, 21.1, 21.0, 18.7, 18.3, 17.5, 17.3, 15.7, 15.1.

6.34. (3*S*,8*S*,9*S*,10*R*,13*R*,14*S*,16*S*)-10,13-Dimethyl-16-((*S*)-6-methylheptan-2-yl)-2,3,4,7,8,9,10,11,12,13,14,15,16,17-tetradecahydro-1H-cyclopenta[a]phenanthren-3-yl 3-methyl-2-phenyl-2-(phenylamino)butanoate (**6g**)

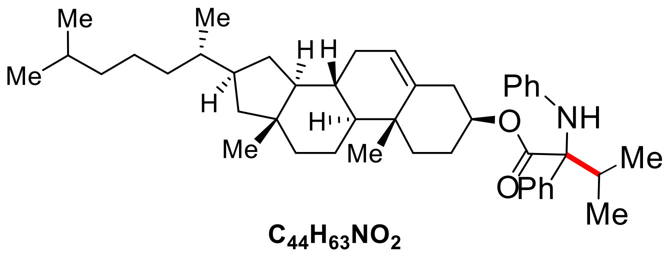

$C_{44}H_{63}NO_2$
M (g/mol): 637.99

Following General Procedure **GP-5** for the title compound **6g** and by using **1h** (103.8 mg, 0.2 mmol, 1.0 equiv), **2a** (22.3 mg, 0.24 mmol, 1.2 equiv), *p*-TSA.H_2O (1.9 mg, 0.01 mmol, 0.05 equiv) and then DHP **5b** (118.2 mg, 0.4 mmol, 2.0 equiv) and Cs_2CO_3 (130.0 mg, 0.4 mmol, 2.0 equiv), in Acetone for 16 h at 35 ºC. Purification was carried out by column chromatography (Hexane/EtOAc = 99:1) to afford **6g** as colourless oil (89.5 mg, 70%).

HRMS (ESI): m/z [M+H]$^+$ Calculated for [$C_{44}H_{64}NO_2$]$^+$: 638.4937; Found:638.4893.

^{1}H NMR (400 MHz, CDCl$_3$) δ 7.71–7.68 (m, 2H), 7.34–7.26 (m, 3H), 7.02–6.97 (m, 2H), 6.66–6.62 (m, 1H), 6.40–6.38 (m, 2H), 5.34–5.18 (m, 1H), 4.58–4.50 (m, 1H), 4.35 (s, 1H), 2.55–2.48 (m, 1H), 2.24–2.15 (m, 1H), 2.00–1.67 (m, 5H), 1.56–1.20 (m, 13H), 1.17–0.96 (m, 10H), 0.94–0.91 (m, 8H), 0.87 (d, *J* = 1.9 Hz, 3H), 0.85–0.84 (m, 4H), 0.83 (s, 2H), 0.65 (s, 3H).

^{13}C NMR (101 MHz, CDCl$_3$). δ 172.7, 172.7, 146.6, 139.7, 139.6, 136.7, 136.6, 129.3, 128.6, 127.5, 127.1, 122.8, 122.7, 118.2, 118.1, 115.6, 74.9, 74.8, 70.7, 70.7, 56.8, 56.2, 50.0, 42.4, 39.8, 39.6, 38.8, 38.1, 37.6, 37.0, 36.9, 36.7, 36.3, 35.9, 32.00, 31.9, 31.7, 28.4, 28.2, 27.8, 27.3, 27.0, 25.4, 24.4, 23.9, 23.00, 22.8, 22.7, 21.1, 20.8, 19.4, 18.8, 18.4, 17.5, 14.3, 12.0.

6.35. Methyl 2-(4-bromophenyl)-3-methyl-2-(phenylamino)butanoate (**6h**)

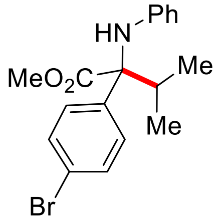

$C_{18}H_{20}BrNO_2$
M (g/mol): 362.27

Following General Procedure **GP-5** for the title compound **6h** and by using **1i** (48.6 mg, 0.2 mmol, 1.0 equiv), **2a** (22.3 mg, 0.24 mmol, 1.2 equiv), *p*-TSA.H_2O (1.9 mg, 0.01 mmol, 0.05 equiv) and then DHP **5b** (118.2 mg, 0.4 mmol, 2.0 equiv) and Cs_2CO_3 (130.0 mg, 0.4 mmol, 2.0 equiv), in Acetone

for 16 h at 35 °C. Purification was carried out by column chromatography (Hexane/EtOAc = 99:1) to afford **6h** as colourless oil (53.0 mg, 73%).

HRMS (ESI): m/z $[M+H]^+$ Calculated for $[C_{18}H_{21}BrNO_2]^+$: 362.0750; Found:.362.0787.

^{1}H NMR (400 MHz, CDCl$_3$) δ 7.59–7.55 (m, 2H), 7.46–7.42 (m, 2H), 7.05–7.00 (m, 2H), 6.69– 6.65 (m, 1H), 6.36– 6.33 (m, 2H), 4.35 (s, 1H), 3.60 (s, 3H), 2.47 (hept, *J* = 6.8 Hz, 1H), 0.90 (d, *J* = 6.8 Hz, 3H), 0.82 (d, *J* = 6.8 Hz, 3H).

^{13}C NMR (101 MHz, CDCl$_3$) δ 173.7, 145.8, 135.3, 131.2, 130.7, 128.8, 121.6, 118.4, 115.2, 70.3, 52.5, 38.9, 18.3, 17.4.

6.36. Methyl 3-methyl-2-(phenylamino)-2-(4-(trifluoromethyl)phenyl)butanoate **(6i)**

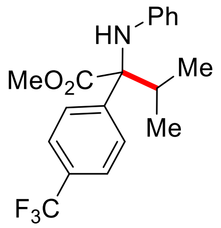

$C_{19}H_{20}F_3NO_2$
M (g/mol): 351.37

Following General Procedure **GP-5** for the title compound **6i** and by using **1m** (46.4 mg, 0.2 mmol, 1.0 equiv), **2a** (22.3 mg, 0.24 mmol, 1.2 equiv), *p*-TSA.H_2O (1.9 mg, 0.01 mmol, 0.05 equiv) and then DHP **5b** (118.2 mg, 0.4 mmol, 2.0 equiv) and Cs_2CO_3 (130.0 mg, 0.4 mmol, 2.0 equiv), in Acetone for 16 h at 35 °C. Purification was carried out by column chromatography (Hexane/EtOAc = 99:1) to afford **6i** as colourless oil (59.0 mg, 84%).

HRMS (ESI): m/z $[M+H]^+$ Calculated for $[C_{19}H_{21}NO_2F_3]^+$: 352.1524; Found:.352.1514.

^{1}H NMR (400 MHz, CDCl$_3$) δ 7.84 (d, *J* = 8.3 Hz, 2H), 7.58 (d, *J* = 8.5 Hz, 2H), 7.06–7.01 (m, 2H), 6.71–6.67 (m, 1H), 6.36–6.32 (m, 2H), 4.38 (s, 1H), 3.62 (s, 3H), 2.51 (heptet, *J* = 6.8 Hz, 1H), 0.92 (d, *J* = 6.7 Hz, 3H), 0.82 (d, *J* = 6.8 Hz, 3H).
^{19}F NMR (376 MHz, CDCl$_3$) δ -62.33.
^{13}C NMR (101 MHz, CDCl$_3$) δ 173.6, 145.6, 140.4, 129.5 (q, *J* = 30.3 Hz), 124.4 (q, *J* = 272.7 Hz), 124.5 (q, *J* = 3.2 Hz), 118.6, 115.2, 70.5, 52.6, 39.2, 18.3, 17.4.

6.37. Methyl 3-methyl-2-(phenylamino)-2-(thiophen-2-yl)butanoate **(6j)**

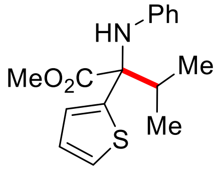

$C_{16}H_{19}NO_2S$
M (g/mol): 289.39

Following General Procedure **GP-5** for the title compound **6j** and by using **1l** (34.0 mg, 0.2 mmol, 1.0 equiv), **2a** (22.3 mg, 0.24 mmol, 1.2 equiv), *p*-TSA.H_2O (1.9 mg, 0.01 mmol, 0.05 equiv) and then

DHP **5b** (118.2 mg, 0.4 mmol, 2.0 equiv) and Cs_2CO_3 (130.0 mg, 0.4 mmol, 2.0 equiv), in Acetone for 16 h at 35 ºC. Purification was carried out by column chromatography (Hexane/EtOAc = 99:2) to afford **6j** as colourless oil (37.6 mg, 65%).

HRMS (ESI): m/z $[M+H]^+$ Calculated for $[C_{16}H_{20}NO_2S]^+$: 290.1215; Found:. 290.1215.

^{1}H NMR (400 MHz, CDCl$_3$) δ 7.26–7.24 (m, 2H), 7.07–7.01 (m, 2H), 7.00–6.94 (m, 1H), 6.70–6.66 (m, 1H), 6.44–6.40 (m, 2H), 4.49 (s, 1H), 3.64 (s, 3H), 2.35 (hept, J = 6.9 Hz, 1H), 0.96–0.92 (m, 6H).

^{13}C NMR (101 MHz, CDCl$_3$) δ 173.8, 145.9, 140.9, 128.8, 127.5, 126.3, 125.6, 118.5, 114.9, 69.5, 52.6, 40.0, 17.9, 17.6.

6.38. Methyl 2-((4-methoxyphenyl)amino)-3-methyl-2-phenylbutanoate **(6k)**

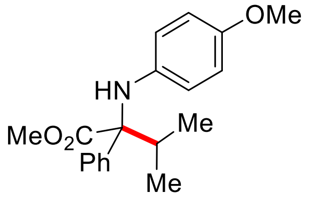

$C_{19}H_{23}NO_3$
M (g/mol): 313.40

Following General Procedure **GP-5** for the title compound **6k** and by using **1a** (32.8 mg, 0.2 mmol, 1.0 equiv), **2e** (29.5 mg, 0.24 mmol, 1.2 equiv), *p*-TSA.H_2O (1.9 mg, 0.01 mmol, 0.05 equiv) and then DHP **5b** (118.2 mg, 0.4 mmol, 2.0 equiv) and Cs_2CO_3 (130.0 mg, 0.4 mmol, 2.0 equiv), in Acetone for 16 h at 35 ºC. Purification was carried out by column chromatography (Hexane/EtOAc = 99:2) to afford **6k** as colourless oil (59.0 mg, 94%).

HRMS (ESI): m/z $[M+H]^+$ Calculated for $[C_{19}H_{24}NO_3]^+$: 314.1756; Found: 314.1758.

^{1}H NMR (400 MHz, CDCl$_3$) δ 7.67–7.64 (m, 2H), 7.34–7.27 (m, 3H), 6.63–6.59 (m, 2H), 6.38–6.34 (m, 2H), 4.15 (br s, 1H), 3.67 (s, 3H), 3.60 (s, 3H), 2.51 (hept, J = 6.8 Hz, 1H), 0.91 (d, J = 6.8 Hz, 3H), 0.83 (d, J = 6.8 Hz, 3H).

^{13}C NMR (101 MHz, CDCl$_3$) δ 174.2, 152.4, 139.9, 136.5, 129.3, 127.5, 127.2, 116.8, 114.2, 71.2, 55.6, 52.2, 38.4, 18.5, 17.5.

6.39. Methyl 2-((4-chlorophenyl)amino)-3-methyl-2-phenylbutanoate (**6l**)

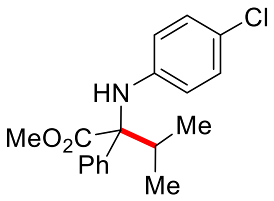

$C_{18}H_{20}ClNO_2$
M (g/mol): 317.81

Following General Procedure **GP-5** for the title compound **6l** and by using **1a** (32.8 mg, 0.2 mmol, 1.0 equiv), **2i** (30.6 mg, 0.24 mmol, 1.2 equiv), *p*-TSA.H_2O (1.9 mg, 0.01 mmol, 0.05 equiv) and then DHP **5b** (118.2 mg, 0.4 mmol, 2.0 equiv) and Cs_2CO_3 (130.0 mg, 0.4 mmol, 2.0 equiv), in Acetone for 16 h at 35 ºC. Purification was carried out by column chromatography (Hexane/EtOAc = 99:1) to afford **6l** as colourless oil (54.5 mg, 86%).

HRMS (ESI): m/z $[M+H]^+$ Calculated for $[C_{18}H_{21}ClNO_2]^+$: 318.1261; Found:. 318.1293.
^{1}H NMR (400 MHz, CDCl$_3$) δ 7.63–7.60 (m, 2H), 7.35–7.29 (m, 3H), 6.97–6.93 (m, 2H), 6.31–6.28 (m, 2H), 4.45 (s, 1H), 3.63 (s, 3H), 2.54 (hept, *J* = 6.8 Hz, 1H), 0.92 (d, *J* = 6.8 Hz, 3H), 0.84 (d, *J* = 6.8 Hz, 3H).
^{13}C NMR (101 MHz, CDCl$_3$) δ 173.8, 144.7, 136.0, 129.0, 128.6, 127.78, 127.5, 122.7, 116.5, 70.7, 52.5, 38.6, 18.3, 17.6.

6.40. Methyl 2-((4-bromophenyl)amino)-3-methyl-2-phenylbutanoate (6m)

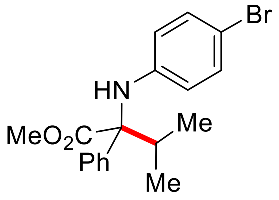

$C_{18}H_{20}BrNO_2$
M (g/mol): 362.27

Following General Procedure **GP-5** for the title compound **6m** and by using **1a** (32.8 mg, 0.2 mmol, 1.0 equiv), **2j** (41.3 mg, 0.24 mmol, 1.2 equiv), *p*-TSA.H_2O (1.9 mg, 0.01 mmol, 0.05 equiv) and then DHP **5b** (118.2 mg, 0.4 mmol, 2.0 equiv) and Cs_2CO_3 (130.0 mg, 0.4 mmol, 2.0 equiv), in Acetone for 16 h at 35 °C. Purification was carried out by column chromatography (Hexane/EtOAc = 99:1) to afford **6m** as colourless oil (58.0 mg, 80%).
HRMS (ESI): m/z $[M+H]^+$ Calculated for $[C_{18}H_{21}NO_2Br]^+$: 362.0756; Found:. 362.0787.
^{1}H NMR (400 MHz, CDCl$_3$) δ 7.63–7.60 (m, 2H), 7.35–7.27 (m, 3H), 7.10–7.06 (m, 2H), 6.27–6.23 (m, 2H), 4.48 (s, 1H), 3.63 (s, 3H), 2.54 (hept, *J* = 6.8 Hz, 1H), 0.92 (d, *J* = 6.9 Hz, 3H), 0.84 (d, *J* = 6.8 Hz, 3H).
^{13}C NMR (101 MHz, CDCl$_3$) δ 173.8, 145.1, 135.9, 131.4, 129.0, 127.8, 127.5, 116.9, 109.9, 70.6, 52.5, 38.6, 18.3, 17.6.

6.41. Methyl-2-((2,6-dimethylphenyl)amino)-3-methyl-2-phenylbutanoate (6n)

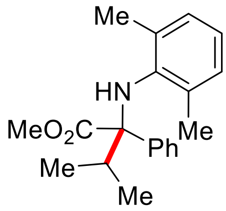

$C_{20}H_{25}NO_2$
M (g/mol): 311.43

Following General Procedure **GP-5** for the title compound **6n** and by using **1a** (32.8 mg, 0.2 mmol, 1.0 equiv), **2m** (29.0 mg, 0.24 mmol, 1.2 equiv), *p*-TSA.H_2O (1.9 mg, 0.01 mmol, 0.05 equiv) and then DHP **5b** (118.2 mg, 0.4 mmol, 2.0 equiv) and Cs_2CO_3 (130.0 mg, 0.4 mmol, 2.0 equiv), in Acetone for 16 h at 35 °C. Purification was carried out by column chromatography (Hexane/EtOAc = 99:1) to afford **6n** as white solid (60.0 mg, 96%).
HRMS (ESI): m/z $[M+H]^+$ Calculated for $[C_{20}H_{26}NO_2]^+$: 312.1964; Found:. 312.1971.
^{1}H NMR (400 MHz, CDCl$_3$) δ 7.54–7.49 (m, 2H), 7.28–7.20 (m, 3H), 6.84 (d, *J* = 7.4 Hz, 2H), 6.72 (dd, *J* = 8.0, 6.9 Hz, 1H), 4.06 (s, 1H), 3.38 (s, 3H), 2.90 (hept, *J* = 6.8 Hz, 1H), 2.07 (s, 6H), 0.93 (d, *J* = 6.7 Hz, 3H), 0.89 (d, *J* = 6.9 Hz, 3H).

^{13}C NMR (101 MHz, CDCl$_3$) δ 173.3, 144.4, 138.3, 132.2, 129.4, 128.8, 127.0, 126.6, 122.5, 74.6, 51.6, 38.5, 20.6, 19.3, 17.5.

6.42. Methyl 2-((2-ethylphenyl)amino)-3-methyl-2-phenylbutanoate **(6o)**

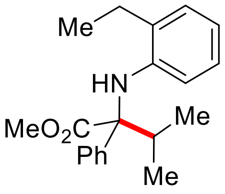

$C_{20}H_{25}NO_2$
M (g/mol): 311.43

Following General Procedure **GP-5** for the title compound **6o** and by using **1a** (32.8 mg, 0.2 mmol, 1.0 equiv), **2b** (29.0 mg, 0.24 mmol, 1.2 equiv), *p*-TSA.H_2O (1.9 mg, 0.01 mmol, 0.05 equiv) and then DHP **5b** (118.2 mg, 0.4 mmol, 2.0 equiv) and Cs_2CO_3 (130.0 mg, 0.4 mmol, 2.0 equiv), in Acetone for 16 h at 35 °C. Purification was carried out by column chromatography (Hexane/EtOAc = 99:1) to afford **6o** as yellow oil (50.0 mg, 80%).
HRMS (ESI): m/z $[M+H]^+$ Calculated for $[C_{20}H_{26}NO_2]^+$: 312.1964; Found:. 312.1969.

.

^{1}H NMR (400 MHz, CDCl$_3$) δ 7.68–7.65 (m, 2H), 7.34–7.25 (m, 3H), 7.09–7.06 (m, 1H), 6.79–6.75 (m, 1H), 6.65–6.61 (m, 1H), 6.02–6.00 (m, 1H), 4.43 (s, 1H), 3.61 (s, 3H), 2.67 (q, *J* = 7.5 Hz, 2H), 2.57 (hept, *J* = 6.8 Hz, 1H), 1.36 (t, *J* = 7.5 Hz, 3H), 0.94 (d, *J* = 6.8 Hz, 3H), 0.88 (d, *J* = 6.8 Hz, 3H).
^{13}C NMR (101 MHz, CDCl$_3$) δ 174.5, 143.3, 136.6, 129.2, 128.5, 128.1, 127.6, 127.2, 126.3, 117.7, 114.0, 70.3, 52.4, 39.2, 24.7, 18.4, 17.8, 13.3.

6.43. Methyl 2-((3,5-bis(trifluoromethyl)phenyl)amino)-3-methyl-2-phenylbutanoate (**6p**)

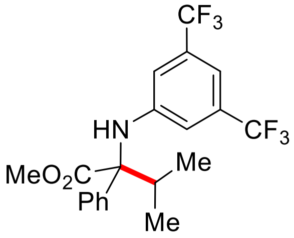

$C_{20}H_{19}F_6NO_2$
M (g/mol): 419.37

Following General Procedure **GP-5** for the title compound **6p** and by using **1a** (32.8 mg, 0.2 mmol, 1.0 equiv), **2l** (55.0 mg, 0.24 mmol, 1.2 equiv), *p*-TSA.H_2O (1.9 mg, 0.01 mmol, 0.05 equiv) and then DHP **5b** (118.2 mg, 0.4 mmol, 2.0 equiv) and Cs_2CO_3 (130.0 mg, 0.4 mmol, 2.0 equiv), in Acetone for 16 h at 35 °C. Purification was carried out by column chromatography (Hexane/EtOAc = 99:1) to afford **6p** as yellow oil (71.0 mg, 85%).
HRMS (ESI): m/z $[M-H]^+$ Calculated for $[C_{20}H_{20}NO_2F_6]^+$: 420.1398; Found:.420.1432.
^{1}H NMR (400 MHz, CDCl$_3$) δ 7.54–7.51 (m, 2H), 7.36–7.30 (m, 3H), 7.08 (s, 1H), 6.70 (s, 2H), 5.08 (s, 1H), 3.68 (s, 3H), 2.71 (hept, *J* = 6.8 Hz, 1H), 0.98 (d, *J* = 6.8 Hz, 3H), 0.94 (d, *J* = 6.6 Hz, 3H).

^{19}F NMR (376 MHz, CDCl$_3$) δ -63.28.

^{13}C NMR (101 MHz, CDCl$_3$) δ 173.3, 146.9, 135.5, 131.7 (q, *J* = 40.4 Hz), 128.4, 128.2, 128.0, 123.6 (q, *J* = 272.7 Hz), 114.7, 110.7, 70.7, 52.8, 37.7, 18.3, 17.9.

6.44. Methyl 2-cyclohexyl-2-phenyl-2-(phenylamino)acetate **(6q)**

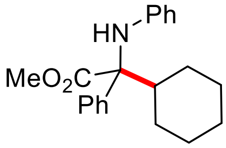

$C_{21}H_{25}NO_2$
M (g/mol): 323.44

Following General Procedure **GP-5** for the title compound **6q** and by using **1a** (32.8 mg, 0.2 mmol, 1.0 equiv), **2a** (22.3 mg, 0.24 mmol, 1.2 equiv), *p*-TSA.H_2O (1.9 mg, 0.01 mmol, 0.05 equiv) and then DHP **5c** (134.5 mg, 0.4 mmol, 2.0 equiv) and Cs_2CO_3 (130.0 mg, 0.4 mmol, 2.0 equiv), in Acetone for 16 h at 35 °C. Purification was carried out by column chromatography (Hexane/EtOAc = 99:1) to afford **6q** as colourless oil (46.5 mg, 72%).
HRMS (ESI): m/z $[M+H]^+$ Calculated for $[C_{21}H_{26}NO_2]^+$: 324.1964; Found:.324.1950.
^{1}H NMR (400 MHz, $CDCl_3$) δ 7.62 (d, *J* = 7.6 Hz, 2H), 7.32–7.24 (m, 3H), 7.01–6.96 (m, 2H), 6.64 (t, *J* = 7.3 Hz, 1H), 6.36 (d, *J* = 8.0 Hz, 2H), 3.60 (s, 3H), 2.21–2.15 (m, 1H), 1.80–1.68 (m, 4H), 1.65–1.58 (m, 1H), 1.28–1.19 (m, 2H), 1.13–0.94 (m, 2H), 0.70 (qd, *J* = 12.8, 3.4 Hz, 1H).

^{13}C NMR (101 MHz, $CDCl_3$) δ 174.1, 146.2, 136.8, 129.1, 128.7, 127.6, 127.2, 117.9, 115.2, 70.8, 52.4, 49.3, 28.7, 28.0, 26.8, 26.6, 26.3.

6.45. Methyl 2-cyclobutyl-2-phenyl-2-(phenylamino)acetate **(6r)**

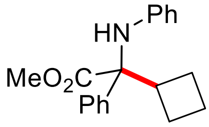

$C_{19}H_{21}NO_2$
M (g/mol): 295.38

Following General Procedure **GP-5** for the title compound **6r** and by using **1a** (32.8 mg, 0.2 mmol, 1.0 equiv), **2a** (22.3 mg, 0.24 mmol, 1.2 equiv), *p*-TSA.H_2O (1.9 mg, 0.01 mmol, 0.05 equiv) and then DHP **5d** (123.0 mg, 0.4 mmol, 2.0 equiv) and Cs_2CO_3 (130.0 mg, 0.4 mmol, 2.0 equiv), in Acetone for 16 h at 35 °C. Purification was carried out by column chromatography (Hexane/EtOAc = 99:1) to afford **6r** as colourless oil (29.5 mg, 50%).

HRMS (ESI): m/z $[M+H]^+$ Calculated for $[C_{19}H_{22}NO_2]^+$: 296.1651; Found:. 296.1631.

^{1}H NMR (400 MHz, $CDCl_3$) δ 7.57–7.51 (m, 2H), 7.35–7.25 (m, 3H), 7.01– 6.96 (m, 2H), 6.65–6.60 (m, 1H), 6.37–6.33 (m, 2H), 5.00 (s, 1H), 3.66 (s, 3H), 3.40–3.32 (m, 1H), 2.15–1.92 (m, 4H), 1.85–1.73 (m, 2H).

^{13}C NMR (101 MHz, $CDCl_3$) δ 173.9, 145.6, 139.6, 128.7, 128.4, 127.8, 127.4, 117.7, 115.4, 68.4, 52.7, 43.3, 25.2, 24.4, 17.3.

6.46. Methyl (3*R*)-3,7-dimethyl-2-phenyl-2-(phenylamino)oct-6-enoate **(6s)**

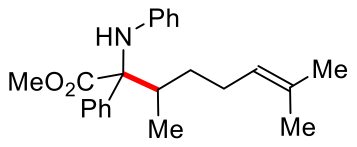

$C_{23}H_{29}NO_2$
M (g/mol): 351.49

Following General Procedure **GP-5** for the title compound **6s** and by using **1a** (32.8 mg, 0.2 mmol, 1.0 equiv), **2a** (22.3 mg, 0.24 mmol, 1.2 equiv), *p*-TSA.H_2O (1.9 mg, 0.01 mmol, 0.05 equiv) and then DHP **5e** (145.5 mg, 0.4 mmol, 2.0 equiv) and Cs_2CO_3 (130.0 mg, 0.4 mmol, 2.0 equiv), in Acetone for 16 h at 35 °C. Purification was carried out by column chromatography (Hexane/EtOAc = 99:1) to afford **6s** as colourless oil (53.0 mg, 75%).Isolated as a mixture of diastereomers (dr = 3:2).

HRMS (ESI): m/z $[M-H]^+$ Calculated for $[C_{23}H_{30}NO_2]^+$: 352.2277; Found:. 352.2269.

^{1}H NMR (400 MHz, $CDCl_3$) δ 7.67–7.63 (m, 3.64H), 7.35 – 7.27 (m, 5.24H), 7.05–6.99 (m, 3.57H), 6.66 (t, *J* = 7.3 Hz, 1.76H), 6.41–6.37 (m, 3.57H), 5.11–5.06 (m, 0.87H), 5.06–5.01 (m, 0.95H), 4.39 (s, 1.81H), 3.62 (s, 2.09H), 3.62 (s, 2.95H), 2.37–2.28 (m, 1.93H), 2.09–2.01 (m, 1.98H), 1.97–1.84 (m, 2.10H), 1.72 (s, 2.64H), 1.70 (s, 3.09H), 1.60 (s, 2.48H), 1.58 (s, 3.23H), 1.54–1.42 (m, 1.32H), 1.13–1.02 (m, 1.23H), 0.97 (d, *J* = 6.8 Hz, 2.70H), 0.86 (d, *J* = 6.6 Hz, 3H), 0.77–0.67 (m, 0.93H).

^{13}C NMR (101 MHz, $CDCl_3$) δ 174.0, 174.0, 146.1, 136.6, 136.5, 132.3, 132.3, 129.2, 129.1, 129.0, 128.7, 127.6, 127.5, 127.2, 124.1, 124.0, 118.1, 115.4, 71.1, 71.0, 52.3, 52.3, 43.5, 43.3, 32.5, 31.6, 26.0, 25.9, 25.9, 17.9, 17.8, 15.4, 14.3.

6.47. Methyl (3*R*)-4-(4-(tert-butyl)phenyl)-3-methyl-2-phenyl-2-(phenylamino)butanoate **(6t)**

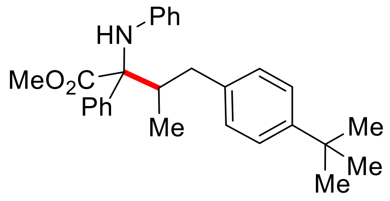

$C_{28}H_{33}NO_2$
M (g/mol): 415.58

Following General Procedure **GP-5** for the title compound **6t** and by using **1a** (32.8 mg, 0.2 mmol, 1.0 equiv), **2a** (22.3 mg, 0.24 mmol, 1.2 equiv), *p*-TSA.H_2O (1.9 mg, 0.01 mmol, 0.05 equiv) and then DHP **5f** (171.0 mg, 0.4 mmol, 2.0 equiv) and Cs_2CO_3 (130.0 mg, 0.4 mmol, 2.0 equiv), in Acetone for 16 h at 35 °C. Purification was carried out by column chromatography (Hexane/EtOAc = 99:1) to afford **6t** as colourless oil (58.0 mg, 70%). Isolated as a mixture of diastereomers (dr = 3:2).

HRMS (ESI): m/z $[M+Na]^+$ Calculated for $[C_{28}H_{33}NO_2Na]^+$: 438.2409; Found:. 438.2464.

^{1}H NMR (400 MHz, $CDCl_3$) δ 7.73-7.68 (m, 3.80H), 7.40-7.28 (m, 9.58H), 7.08–7.00 (m, 7.64H), 6.69 (t, *J* = 7.3 Hz, 1.80H), 6.45–6.42 (m, 3.64H), 3.68 (s, 3H), 3.65 (s, 2.08H), 3.01–2.90 (m, 1.86H), 2.70 (br s, 1.84H), 2.10 (dd, *J* = 13.3, 11.0 Hz, 1.17H), 1.86 (dd, *J* = 13.7, 10.6 Hz, 0.73H), 1.32 (s, 5.84H), 1.31 (s, 9.31H), 0.90 (d, *J* = 6.4 Hz, 2.20H), 0.81 (d, *J* = 6.9 Hz, 3.27H).

^{13}C NMR (101 MHz, CDCl$_3$) δ 173.8, 173.7, 149.1, 145.7, 137.4, 137.3, 136.5, 136.2, 135.1, 130.2, 130.2, 129.2, 129.0, 128.9, 128.9, 128.7, 127.8, 127.5, 125.4, 125.3, 118.4, 115.8, 71.3, 62.5, 60.6, 53.6, 52.5, 45.8, 38.5, 37.7, 34.5, 31.5, 21.2, 15.4, 14.2.

6.48. Methyl 3-ethyl-2-phenyl-2-(phenylamino)pentanoate **(6u)**

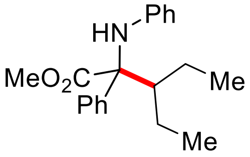

$C_{20}H_{25}NO_2$
M (g/mol): 311.42

Following General Procedure **GP-5** for the title compound **6u** and by using **1a** (32.8 mg, 0.2 mmol, 1.0 equiv), **2a** (22.3 mg, 0.24 mmol, 1.2 equiv), *p*-TSA.H_2O (1.9 mg, 0.01 mmol, 0.05 equiv) and then DHP **5g** (129.0 mg, 0.4 mmol, 2.0 equiv) and Cs_2CO_3 (130.0 mg, 0.4 mmol, 2.0 equiv), in Acetone for 16 h at 35 ºC. Purification was carried out by column chromatography (Hexane/EtOAc = 99:1) to afford **6u** as colourless oil (51.0 mg, 82%).

MS-MS (ESI): m/z $[M+H]^+$ Calculated for $[C_{20}H_{26}NO_2]^+$: 312.19; Found:. 312.19.

^{1}H NMR (400 MHz, CDCl$_3$) δ 7.68–7.65 (m, 2H), 7.34–7.25 (m, 3H), 7.04–6.99 (m, 2H), 6.68–6.63 (m, 1H), 6.38–6.35 (m, 2H), 4.38 (s, 1H), 3.61 (s, 3H), 2.02–1.96 (m, 1H), 1.73–1.61 (m, 1H), 1.60–1.54 (m, 1H), 1.24–1.14 (m, 1H), 1.07–0.95 (m, 1H), 0.92 (d, *J* = 7.4 Hz, 3H), 0.88 (d, *J* = 7.4 Hz, 3H).

^{13}C NMR (101 MHz, CDCl$_3$) δ 174.2, 146.1, 137.2, 129.1, 128.7, 127.6, 127.2, 118.0, 115.4, 71.4, 53.1, 52.3, 24.2, 23.5, 13.8.

6.49. Methyl 4-methyl-2-phenyl-2-(phenylamino)pentanoate **(6v)**

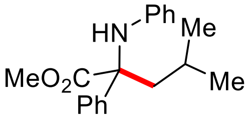

$C_{19}H_{23}NO_2$
M (g/mol): 297.40

Following General Procedure **GP-5** for the title compound **6v** and by using **1a** (32.8 mg, 0.2 mmol, 1.0 equiv), **2a** (22.3 mg, 0.24 mmol, 1.2 equiv), *p*-TSA.H_2O (1.9 mg, 0.01 mmol, 0.05 equiv) and then DHP **5h** (124.0 mg, 0.4 mmol, 2.0 equiv) and Cs_2CO_3 (130.0 mg, 0.4 mmol, 2.0 equiv), in Acetone for 16 h at 35 ºC. Purification was carried out by column chromatography (Hexane/EtOAc = 99:1) to afford **6v** as colourless oil (27.0 mg, 45%).

MS-MS (ESI): m/z $[M+H]^+$ Calculated for $[C_{19}H_{24}NO_2]^+$: 298.1807; Found:. 298.1798.

^{1}H NMR (400 MHz, CDCl$_3$) δ 7.58–7.55 (m, 2H), 7.34–7.29 (m, 2H), 7.25–7.23 (m, 1H), 7.02–6.97 (m, 2H), 6.58 (t, *J* = 7.3 Hz, 1H), 6.35–6.32 (m, 2H), 3.65 (s, 3H), 2.60–2.48 (m, 2H), 1.76–1.67 (m, 1H), 0.85 (d, *J* = 6.6 Hz, 3H), 0.81 (d, *J* = 6.8 Hz, 3H).

^{13}C NMR (101 MHz, CDCl$_3$) δ 175.2, 144.6, 141.4, 128.9, 128.7, 127.6, 127.1, 117.0, 114.6, 65.4, 53.1, 41.9, 24.6, 24.4, 22.3.

6.50. Methyl 2,4-diphenyl-2-(phenylamino)butanoate (6w)

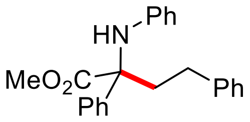

$C_{23}H_{23}NO_2$
M (g/mol): 345.44

Following General Procedure **GP-5** for the title compound **6w** and by using **1a** (32.8 mg, 0.2 mmol, 1.0 equiv), **2a** (22.3 mg, 0.24 mmol, 1.2 equiv), *p*-TSA.H_2O (1.9 mg, 0.01 mmol, 0.05 equiv) and then DHP **5i** (143.0 mg, 0.4 mmol, 2.0 equiv) and Cs_2CO_3 (130.0 mg, 0.4 mmol, 2.0 equiv), in Acetone for 16 h at 35 °C. Purification was carried out by column chromatography (Hexane/EtOAc = 99:1) to afford **6w** as colourless oil (45.0 mg, 65%).

HRMS (ESI): m/z $[M+H]^+$ Calculated for $[C_{23}H_{24}NO_2]^+$: 346.1807; Found:. 346.1804.

^{1}H NMR (400 MHz, CDCl$_3$) δ 7.60–7.57 (m, 2H), 7.35–7.20 (m, 6H), 7.17–7.13 (m, 1H), 7.03–6.99 (m, 3H), 6.65 (t, *J* = 7.3 Hz, 1H), 6.43–6.40 (m, 2H), 5.48 (s, 1H), 3.64 (s, 3H), 2.93–2.78 (m, 2H), 2.71–2.64 (m, 1H), 2.40–2.32 (m, 1H).

^{13}C NMR (101 MHz, CDCl$_3$) δ 174.3, 144.2, 141.5, 140.7, 129.1, 128.8, 128.6, 128.5, 127.8, 127.1, 126.1, 117.7, 115.2, 66.2, 53.4, 35.1, 30.7.

6.51. Methyl 2-phenyl-2-(phenylamino)pentanoate (6x)

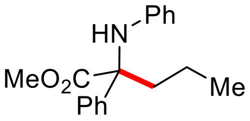

$C_{18}H_{21}NO_2$
M (g/mol): 283.37

Following General Procedure **GP-5** for the title compound **6x** and by using **1a** (32.8 mg, 0.2 mmol, 1.0 equiv), **2a** (22.3 mg, 0.24 mmol, 1.2 equiv), *p*-TSA.H_2O (1.9 mg, 0.01 mmol, 0.05 equiv) and then DHP **5l** (118.0 mg, 0.4 mmol, 2.0 equiv) and Cs_2CO_3 (130.0 mg, 0.4 mmol, 2.0 equiv), in Acetone for 16 h at 35 °C. Purification was carried out by column chromatography (Hexane/EtOAc = 99:1) to afford **6x** as colourless oil (39.5 mg, 70%).

HRMS (ESI): m/z $[M+H]^+$ Calculated for $[C_{18}H_{22}NO_2]^+$: 284.1651; Found: 284.1646.

^{1}H NMR (400 MHz, CDCl$_3$) δ 7.59–7.57 (m, 2H), 7.36–7.27 (m, 3H), 7.03–6.98 (m, 2H), 6.64 (t, *J* = 7.4 Hz, 1H), 6.39 (d, *J* = 7.9 Hz, 2H), 3.66 (s, 3H), 2.53–2.45 (m, 2H), 1.45–1.36 (m, 1H), 1.16–1.02 (m, 1H), 0.88 (t, *J* = 7.3 Hz, 3H).

^{13}C NMR (101 MHz, CDCl$_3$) δ 174.5, 144.3, 140.8, 128.9, 128.7, 127.7, 127.2, 117.6, 115.2, 66.6, 53.2, 35.4, 17.6, 14.3.

6.52. Methyl 2-phenyl-2-(phenylamino)hexanoate (6y)

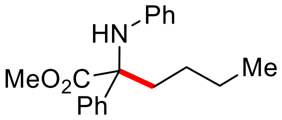

$C_{19}H_{23}NO_2$
M (g/mol): 297.40

Following General Procedure **GP-5** for the title compound **6y** and by using **1a** (32.8 mg, 0.2 mmol, 1.0 equiv), **2a** (22.3 mg, 0.24 mmol, 1.2 equiv), *p*-TSA.H_2O (1.9 mg, 0.01 mmol, 0.05 equiv) and then DHP **5k** (124.0 mg, 0.4 mmol, 2.0 equiv) and Cs_2CO_3 (130.0 mg, 0.4 mmol, 2.0 equiv), in Acetone for 16 h at 35 °C. Purification was carried out by column chromatography (Hexane/EtOAc = 99:1) to afford **6y** as colourless oil (35.5 mg, 60%).

MS-MS (ESI): m/z $[M+H]^+$ Calculated for $[C_{19}H_{24}NO_2]^+$: 298.18; Found:. 298.18.

^{1}H NMR (400 MHz, $CDCl_3$) δ 7.62–7.59 (m, 2H), 7.37–7.27 (m, 3H), 7.02– 6.98 (m, 2H), 6.62 (t, *J* = 7.3 Hz, 1H), 6.37 (d, *J* = 8.1 Hz, 2H), 3.67 (s, 3H), 2.57–2.45 (m, 2H), 1.37–1.22 (m, 3H), 1.09–0.98 (m, 1H), 0.82 (t, *J* = 7.2 Hz, 3H).

^{13}C NMR (101 MHz, $CDCl_3$) δ 174.6, 144.3, 140.9, 128.9, 128.7, 127.7, 127.1, 117.4, 115.1, 66.4, 53.3, 32.8, 32.7, 26.3, 26.3, 22.7, 14.1.

6.53. Methyl 3-(benzyloxy)-2-phenyl-2-(phenylamino)propanoate (6z)

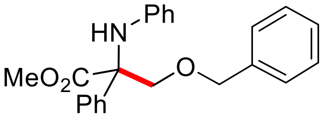

$C_{23}H_{23}NO_3$
M (g/mol): 361.44

Following General Procedure **GP-5** for the title compound **6z** and by using **1a** (32.8 mg, 0.2 mmol, 1.0 equiv), **2a** (22.3 mg, 0.24 mmol, 1.2 equiv), *p*-TSA.H_2O (1.9 mg, 0.01 mmol, 0.05 equiv) and then DHP **5m** (150.0 mg, 0.4 mmol, 2.0 equiv) and Cs_2CO_3 (130.0 mg, 0.4 mmol, 2.0 equiv), in Acetone for 16 h at 35 °C. Purification was carried out by column chromatography (Hexane/EtOAc = 99:1) to afford **6z** as colourless oil (68.0 mg, 94%).
HRMS (ESI): m/z $[M+H]^+$ Calculated for $[C_{23}H_{24}NO_3]^+$: 362.1756; Found: 362.1759.

^{1}H NMR (400 MHz, $CDCl_3$) δ 7.55–7.52 (m, 2H), 7.35–7.30 (m, 2H), 7.29–7.21 (m, 4H), 7.16–7.11 (m, 2H), 7.02– 6.97 (m, 2H), 6.68–6.64 (m, 1H), 6.38–6.34 (m, 2H), 5.32 (s, 1H), 4.52–4.44 (m, 2H), 4.33 (d, *J* = 9.1 Hz, 1H), 4.10 (d, *J* = 9.1 Hz, 1H), 3.69 (s, 3H).

^{13}C NMR (101 MHz, $CDCl_3$) δ 173.0, 145.0, 138.0, 137.8, 128.9, 128.8, 128.4, 128.1, 127.7, 127.6, 127.1, 118.1, 116.0, 73.4, 70.7, 67.6, 53.2.

6.54. Methyl 2,3-diphenyl-2-(phenylamino)propanoate **(6aa)**

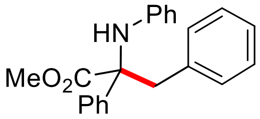

$C_{22}H_{21}NO_2$
M (g/mol): 331.42

Following General Procedure **GP-5** for the title compound **6aa** and by using **1a** (32.8 mg, 0.2 mmol, 1.0 equiv), **2a** (22.3 mg, 0.24 mmol, 1.2 equiv), *p*-TSA.H_2O (1.9 mg, 0.01 mmol, 0.05 equiv) and then DHP **5j** (137.5 mg, 0.4 mmol, 2.0 equiv) and Cs_2CO_3 (130.0 mg, 0.4 mmol, 2.0 equiv), in Acetone for 16 h at 35 °C. Purification was carried out by column chromatography (Hexane/EtOAc = 99:1) to afford **6aa** as colourless oil (46.5 mg, 70%).

HRMS (ESI): m/z $[M+H]^+$ Calculated for $[C_{22}H_{22}NO_2]^+$: 332.1651; Found:. 332.1642.

^{1}H NMR (400 MHz, CDCl$_3$) δ 7.69–7.66 (m, 2H), 7.42–7.37 (m, 2H), 7.35–7.30 (m, 1H), 7.25–7.17 (m, 3H), 7.09– 7.03 (m, 2H), 6.95–6.90 (m, 2H), 6.66 (t, *J* = 7.3 Hz, 1H), 6.36 (d, *J* = 8.1 Hz, 2H), 5.23 (br s, 1H) 3.90–3.82 (m, 2H), 3.66 (s, 3H).

^{13}C NMR (101 MHz, CDCl$_3$) δ 173.4, 144.4, 140.3, 136.1, 130.3, 129.1, 129.0, 128.2, 127.9, 127.2, 127.1, 117.3, 115.0, 67.3, 53.0, 37.8.

6.55. Methyl 3,3-dimethyl-2-phenyl-2-(phenylamino)butanoate **(6ab)**

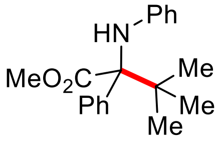

$C_{19}H_{23}NO_2$
M (g/mol): 297.40

Following General Procedure **GP-5** for the title compound **6ab** and by using **1a** (32.8 mg, 0.2 mmol, 1.0 equiv), **2a** (22.3 mg, 0.24 mmol, 1.2 equiv), *p*-TSA.H_2O (1.9 mg, 0.01 mmol, 0.05 equiv) and then DHP **5n** (86.2 mg, 0.4 mmol, 2.0 equiv) and Cs_2CO_3 (130.0 mg, 0.4 mmol, 2.0 equiv), in Acetone for 16 h at 35 °C. Purification was carried out by column chromatography (Hexane/EtOAc = 99:1) to afford **6ab** as colourless oil (44.0 mg, 74%).

HRMS (ESI): m/z $[M+H]^+$ Calculated for $[C_{19}H_{24}NO_2]^+$: 298.1807; Found: 298.1803.

^{1}H NMR (400 MHz, CDCl$_3$) δ 7.96 (s, 1H), 7.38–7.15 (m, 4H), 7.01–6.95 (m, 2H), 6.64–6.55 (m, 1H), 6.21 (d, *J* = 7.8 Hz, 2H), 4.52 (s, 1H), 3.55 (s, 3H), 1.02 (s, 9H).

^{13}C NMR (101 MHz, CDCl$_3$) δ 173.8, 146.6, 136.8, 130.1, 128.6, 127.3, 127.1, 117.6, 115.0, 71.7, 51.8, 39.4, 26.5.

6.56. Methyl 3,3-dimethyl-2,4-diphenyl-2-(phenylamino)butanoate (6ac)

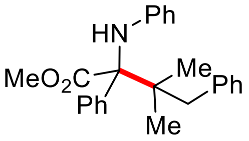

$C_{25}H_{27}NO_2$
M (g/mol): 373.50

Following General Procedure **GP-5** for the title compound **6ac** and by using **1a** (32.8 mg, 0.2 mmol, 1.0 equiv), **2a** (22.3 mg, 0.24 mmol, 1.2 equiv), *p*-TSA.H_2O (1.9 mg, 0.01 mmol, 0.05 equiv) and then DHP **5o** (117.0 mg, 0.4 mmol, 2.0 equiv) and Cs_2CO_3 (130.0 mg, 0.4 mmol, 2.0 equiv), in Acetone for 16 h at 35 °C. Purification was carried out by column chromatography (Hexane/EtOAc = 99:1) to afford **6ac** as colourless oil (51.0 mg, 68%).

HRMS (ESI): m/z $[M+H]^+$ Calculated for $[C_{25}H_{28}NO_2]^+$: 374.2120; Found: 374.2112.

^{1}H NMR (400 MHz, $CDCl_3$) δ 8.08–8.00 (m, 2H), 7.30–7.26 (m, 3H), 7.24–7.18 (m, 3H), 7.08–7.04 (m, 2H), 7.00– 6.95 (m, 2H), 6.64–6.59 (m, 1H), 6.21–6.18 (m, 2H), 3.60 (s, 3H), 2.67 (s, 2H), 0.96 (s, 3H), 0.94 (s, 3H).

^{13}C NMR (101 MHz, $CDCl_3$) δ 173.7, 146.5, 138.2, 136.5, 131.4, 130.4, 128.6, 127.9, 127.5, 127.3, 126.4, 117.7, 115.2, 72.7, 51.9, 43.3, 43.0, 23.0, 22.7.

6.57. Methyl 4-cyano-2-phenyl-2-(phenylamino)butanoate (8a)

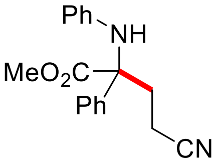

$C_{18}H_{18}N_2O_2$
M (g/mol): 294.35

Following General Procedure **GP-4** for the title compound **8a** and by using **1a** (32.8 mg, 0.2 mmol, 1.0 equiv), **2a** (22.3 mg, 0.24 mmol, 1.2 equiv), *p*-TSA.H_2O (1.9 mg, 0.01 mmol, 0.05 equiv) and then hantzsch ester **5a** (50.5 mg, 0.2 mmol, 1.0 equiv), Cs_2CO_3 (97.5 mg, 0.3 mmol, 1.5 equiv), and **3k** (40 µL, 0.6 mmol, 3.0 equiv) in Acetone for 16 h at 35 °C. Purification was carried out by column chromatography (Hexane/EtOAc = 97:3) to afford **8a** as colourless oil (46.0 mg, 78%).

MS-MS (ESI): m/z $[M+H]^+$ Calculated for $[C_{18}H_{19}N_2O_2]^+$: 295.14; Found: 295.15.

^{1}H NMR (400 MHz, $CDCl_3$) δ 7.53–7.50 (m, 2H), 7.39–7.30 (m, 3H), 7.07–7.02 (m, 2H), 6.70–6.66 (m, 1H), 6.40–6.37 (m, 2H), 5.45 (s, 1H), 3.74 (s, 3H), 3.14–3.06 (m, 1H), 2.94–2.87 (m, 1H), 2.46–2.38 (m, 1H), 2.29–2.21(m, 1H).

^{13}C NMR (101 MHz, $CDCl_3$) δ 173.1, 143.1, 139.0, 129.3, 129.1, 128.3, 126.6, 119.1, 118.4, 115.0, 65.1, 53.8, 28.9, 12.6.

6.58. 5-(tert-butyl) 1-methyl 2-phenyl-2-(phenylamino)pentanedioate **(8b)**

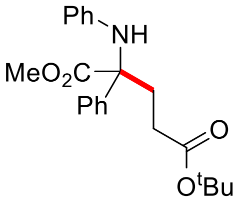

$C_{22}H_{27}NO_4$
M (g/mol): 369.46

Following General Procedure **GP-4** for the title compound **8b** and by using **1a** (32.8 mg, 0.2 mmol, 1.0 equiv), **2a** (22.3 mg, 0.24 mmol, 1.2 equiv), *p*-TSA.H_2O (1.9 mg, 0.01 mmol, 0.05 equiv) and then hantzsch ester **5a** (50.5 mg, 0.2 mmol, 1.0 equiv), Cs_2CO_3 (97.5 mg, 0.3 mmol, 1.5 equiv), and **3i** (88 µL, 0.6 mmol, 3.0 equiv) in Acetone for 16 h at 35 °C. Purification was carried out by column chromatography (Hexane/EtOAc = 97:3) to afford **8b** as colourless oil (56.0 mg, 76%).

MS-MS (ESI): m/z $[M+H]^+$ Calculated for $[C_{22}H_{28}NO_4]^+$: 370.20; Found: 370.21.

^{1}H NMR (400 MHz, $CDCl_3$) δ 7.58–7.57 (m, 2H), 7.35–7.25 (m, 3H), 7.02–6.98 (m, 2H), 6.62 (t, J = 7.2 Hz, 1H), 6.38 (d, J = 8.0 Hz, 2H), 5.43 (s, 1H), 3.66 (s, 3H), 2.94–2.87 (m, 1H), 2.80–2.73 (m, 1H), 2.35–2.27 (m, 1H), 2.11–2.02 (m, 1H), 1.39 (s, 9H).

^{13}C NMR (101 MHz, $CDCl_3$) δ 174.1, 172.4, 143.9, 139.9, 129.0, 128.8, 127.8, 127.1, 117.7, 115.1, 80.7, 65.7, 53.5, 30.5, 28.8, 28.2.

6.59. 5-benzyl 1-methyl 2-phenyl-2-(phenylamino)pentanedioate **(8c)**

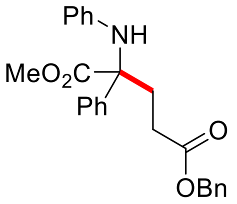

$C_{25}H_{25}NO_4$
M (g/mol): 403.08

Following General Procedure **GP-4** for the title compound **8c** and by using **1a** (32.8 mg, 0.2 mmol, 1.0 equiv), **2a** (22.3 mg, 0.24 mmol, 1.2 equiv), *p*-TSA.H_2O (1.9 mg, 0.01 mmol, 0.05 equiv) and then hantzsch ester **5a** (50.5 mg, 0.2 mmol, 1.0 equiv), Cs_2CO_3 (97.5 mg, 0.3 mmol, 1.5 equiv), and **3h** (97 µL, 0.6 mmol, 3.0 equiv) in Acetone for 16 h at 35 °C. Purification was carried out by column chromatography (Hexane/EtOAc = 97:3) to afford **8c** as colourless oil (66.0 mg, 82%).

HRMS (ESI): m/z $[M+H]^+$ Calculated for $[C_{25}H_{26}NO_4]^+$: 404.1862; Found: 404.1880

^{1}H NMR (400 MHz, $CDCl_3$) δ 7.56–7.54 (m, 2H), 7.36–7.30 (m, 5H),7.29–7.27(m, 3H), 7.01–6.97 (m, 2H), 6.61 (t, J = 7.3 Hz, 1H), 6.37– 6.34 (m, 2H), 5.36 (s, 1H), 5.03–4.96 (m, 2H), 3.65 (s, 3H), 3.01–293 (m, 1H), 2.90–2.83 (m, 1H), 2.46–2.38 (m, 1H), 2.25–2.17 (m, 1H).

^{13}C NMR (101 MHz, $CDCl_3$) δ 174.0, 172.9, 143.9, 139.9, 135.9, 129.1, 128.9, 128.7, 128.4, 127.9, 127.0, 117.8, 115.0, 66.5, 65.6, 53.6, 29.5, 28.6.

6.60. Methyl 5-(dimethylamino)-5-oxo-2-phenyl-2-(phenylamino)pentanoate **(8d)**

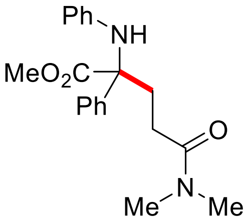

$C_{20}H_{24}N_2O_3$
M (g/mol): 340.42

Following General Procedure **GP-4** for the title compound **8d** and by using **1a** (32.8 mg, 0.2 mmol, 1.0 equiv), **2a** (22.3 mg, 0.24 mmol, 1.2 equiv), *p*-TSA.H_2O (1.9 mg, 0.01 mmol, 0.05 equiv) and then hantzsch ester **5a** (50.5 mg, 0.2 mmol, 1.0 equiv), Cs_2CO_3 (97.5 mg, 0.3 mmol, 1.5 equiv), and **3l** (62 µL, 0.6 mmol, 3.0 equiv) in Acetone for 16 h at 35 °C. Purification was carried out by column chromatography (Hexane/EtOAc = 97:3) to afford **8d** as colourless oil (47.5 mg, 70%).

HRMS (ESI): m/z $[M+H]^+$ Calculated for $[C_{20}H_{25}N_2O_3]^+$: 341.1860; Found: 341.1863.

^{1}H NMR (400 MHz, $CDCl_3$) δ 7.60–7.57 (m, 2H), 7.35–7.30 (m, J = 8.3, 2H), 7.28–7.24 (m, 1H), 7.00–6.95 (m, 2H), 6.60 (t, J = 7.3 Hz, 1H), 6.39–6.35 (m, 2H), 3.65 (s, 3H), 3.04–2.97 (m, 1H), 2.86 (s, 3H), 2.81–2.76 (m, 1H), 2.74 (s, 3H), 2.45–2.37 (m, 1H), 2.07–1.99(m, 1H).

^{13}C NMR (101 MHz, $CDCl_3$) δ 174.4, 172.1, 144.1, 140.1, 129.0, 128.7, 127.8, 127.2, 117.7, 115.0, 65.8, 53.5, 37.1, 35.5, 29.4, 28.4.

6.61. N-(4,4-difluoro-1,1,3-triphenylbut-3-en-1-yl)aniline **(8e)**

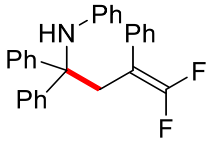

$C_{28}H_{23}F_2N$
M (g/mol): 411.49

Following General Procedure **GP-5** for the title compound **9e** and by using **1n** (36.4 mg, 0.2 mmol, 1.0 equiv), **2n** (22.3 mg, 0.24 mmol, 1.2 equiv), *p*-TSA.H_2O (1.9 mg, 0.01 mmol, 0.05 equiv) and then hantzsch ester **5a** (50.5 mg, 0.2 mmol, 1.0 equiv), Cs_2CO_3 (97.5 mg, 0.3 mmol, 1.5 equiv), and **3a** (105 µL, 0.6 mmol, 3.0 equiv) in Acetone for 16 h at 35 °C. Purification was carried out by column chromatography (Hexane/EtOAc = 97:3) to afford **8e** as colourless oil (43.0 mg, 52%).

MS-MS (ESI): m/z $[M+H]^+$ Calculated for $[C_{16}H_{15}F_3NO_2]^+$: 412.19; Found: 412.18.

^{1}H NMR (400 MHz, $CDCl_3$)δ 7.45–7.42 (m, 4H), 7.29–7.25 (m, 5H), 7.22–7.19 (m, 4H), 6.99–6.95 (m, 2H), 6.80– 6.74 (m, 2H), 6.46 (t, J = 7.3 Hz, 1H), 5.85–5.82 (d, 2H), 4.30 (s, 1H), 3.50 (t, J = 2.3 Hz, 2H).

^{13}C NMR (101 MHz, $CDCl_3$)δ 154.6 (dd, J = 292.9, 288.8 Hz), 145.4, 143.5, 134.0 (d, J = 3.0 Hz), 128.7, 128.6, 128.4, 128.2, 127.8, 127.5, 127.0, 117.0, 115.2, 89.6 (dd, J = 19.7, 19.1 Hz), 66.3, 40.3.

^{19}F NMR (376 MHz, $CDCl_3$) δ -89.26 (d, *J* = 34.7 Hz), -90.40 (d, *J* = 34.7 Hz).

6.62. Diethyl 2-(3,3-difluoro-2-phenylallyl)-2-(phenylamino)malonate **(8f)**

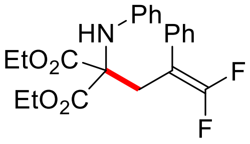

$C_{22}H_{23}F_2NO_4$
M (g/mol): 403.43

Following General Procedure **GP-4** for the title compound **8f** and by using **1r** (34.8 mg, 0.2 mmol, 1.0 equiv), **2a** (22.3 mg, 0.24 mmol, 1.2 equiv), *p*-TSA.H_2O (1.9 mg, 0.01 mmol, 0.05 equiv) and then hantzsch ester **5a** (50.5 mg, 0.2 mmol, 1.0 equiv), Cs_2CO_3 (97.5 mg, 0.3 mmol, 1.5 equiv), and **3a** (105 µL, 0.6 mmol, 3.0 equiv) in Acetone for 16 h at 35 ºC. Purification was carried out by column chromatography (Hexane/EtOAc = 97:3) to afford **8f** as colourless oil (72.5 mg, 90%).

HRMS (ESI): m/z $[M+H]^+$ Calculated for $[C_{22}H_{24}F_2NO_4]^+$: 404.1673; Found: 404.1652.

^{1}H NMR (400 MHz, $CDCl_3$) δ 7.24–7.17 (m, 3H), 7.15–7.12 (m, 2H), 7.08–7.04 (m, 2H), 6.69 (t, *J* = 7.3 Hz, 1H), 6.42 (d, *J* = 7.9 Hz, 2H), 4.96 (s, 1H), 4.03 (dq, *J* = 10.6, 7.1 Hz, 2H), 3.83 (dq, *J* = 10.9, 7.1 Hz, 2H), 3.51 (t, *J* = 2.2 Hz, 2H), 1.08 (t, *J* = 7.1 Hz, 6H).

^{13}C NMR (101 MHz, $CDCl_3$) δ 169.3, 155.0 (t, J = 292.9 Hz), 143.9, 132.8, 129.1, 128.6, 128.1, 127.4, 118.1, 113.8, 87.8 (dd, J = 19.7, 19.1 Hz), 67.3, 62.5, 30.1, 13.8.

^{19}F NMR (376 MHz, $CDCl_3$) δ -88.59 (d, *J* = 34.7 Hz), -88.80 (d, *J* = 34.7 Hz).

6.63. (*S*)-N-(2-methyl-1-phenylpropyl)aniline (**9a**)

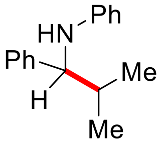

$C_{16}H_{19}N$
M (g/mol): 225.34

Following General Procedure **GP-5** for the title compound **9a** and by using **1p** (21.2 mg, 0.2 mmol, 1.0 equiv), **2a** (22.3 mg, 0.24 mmol, 1.2 equiv), *p*-TSA.H_2O (1.9 mg, 0.01 mmol, 0.05 equiv) and then DHP **5b** (118.2 mg, 0.4 mmol, 2.0 equiv) and Cs_2CO_3 (130.0 mg, 0.4 mmol, 2.0 equiv), in Acetone for 16 h at 35 ºC. Purification was carried out by column chromatography (Hexane) to afford **9a** as colourless oil (34.0 mg, 75%).

HRMS (ESI): m/z $[M+H]^+$ Calculated for $[C_{16}H_{20}N]^+$: 226.1596; Found:226.1589.

^{1}H NMR (400 MHz, $CDCl_3$) δ 7.32–7.37 (m, 4H), 7.25–7.19 (m, 1H), 7.10–7.05 (m, 2H), 6.64–6.59 (m, 1H), 6.53–6.49 (m, 2H), 4.13 (d, *J* = 5.8 Hz, 1H), 2.10–1.99 (m, 1H), 1.00 (d, *J* = 6.7 Hz, 3H), 0.93 (d, *J* = 6.8 Hz, 3H).

^{13}C NMR (101 MHz, $CDCl_3$) δ 147.8, 142.7, 129.2, 128.3, 127.3, 126.9, 117.1, 113.3, 63.9, 35.0, 19.9, 18.7.

6.64. (*S*)-4-methoxy-N-(2-methyl-1-phenylpropyl)aniline **(9b)**

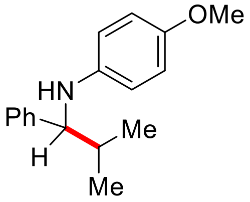

$C_{17}H_{21}N$
M (g/mol): 255.36

Following General Procedure **GP-5** for the title compound **9a** and by using **1p** (21.2 mg, 0.2 mmol, 1.0 equiv), **2e** (29.5 mg, 0.24 mmol, 1.2 equiv), *p*-TSA.H_2O (1.9 mg, 0.01 mmol, 0.05 equiv) and then DHP **5b** (118.2 mg, 0.4 mmol, 2.0 equiv) and Cs_2CO_3 (130.0 mg, 0.4 mmol, 2.0 equiv), in Acetone for 16 h at 35 ºC. Purification was carried out by column chromatography (Hexane) to afford **9b** as colourless oil (32.0 mg, 62%).

HRMS (ESI): m/z $[M+H]^+$ Calculated for $[C_{17}H_{22}NO]^+$: 256.1701; Found:256.1696.

^{1}H NMR (400 MHz, CDCl$_3$) δ 7.32–7.26 (m, 4H), 7.24–7.18 (m, 1H), 6.75 (dd, *J* = 7.8, 1.4 Hz, 1H), 6.67 (td, *J* = 7.5, 1.5 Hz, 1H), 6.57 (td, *J* = 7.8, 1.6 Hz, 1H), 6.31 (dd, *J* = 7.8, 1.7 Hz, 1H), 4.78 (s, 1H), 4.11 (t, *J* = 5.4 Hz, 1H), 3.90 (s, 3H), 2.07 (hept, *J* = 6.7 Hz, 1H), 1.01 (d, *J* = 6.8 Hz, 3H), 0.94 (d, *J* = 6.9 Hz, 3H).

^{13}C NMR (101 MHz, CDCl$_3$) δ 146.8, 143.0, 137.8, 128.3, 127.3, 126.8, 121.3, 116.0, 110.8, 109.3, 63.8, 55.7, 35.1, 20.0, 18.8.

6.65. Diethyl 2-isopropyl-2-(phenylamino)malonate **(9c)**

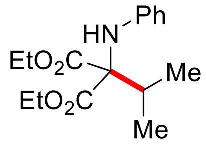

$C_{16}H_{23}NO_4$
M (g/mol): 293.36

Following General Procedure **GP-5** for the title compound **9b** and by using **1r** (34.8 mg, 0.2 mmol, 1.0 equiv), **2a** (22.3 mg, 0.24 mmol, 1.2 equiv), *p*-TSA.H_2O (1.9 mg, 0.01 mmol, 0.05 equiv) and then DHP **5b** (118.2 mg, 0.4 mmol, 2.0 equiv) and Cs_2CO_3 (130.0 mg, 0.4 mmol, 2.0 equiv), in Acetone for 16 h at 35 ºC. Purification was carried out by column chromatography (Hexane/EtOAc = 98:2) to afford **9b** as colourless oil (47.0 mg, 80%).

MS-MS (ESI): m/z $[M+H]^+$ Calculated for $[C_{16}H_{24}NO_4]^+$: 294.1705; Found:294.1718.

^{1}H NMR (400 MHz, CDCl$_3$) δ 7.14–7.09 (m, 2H), 6.74–6.69 (m, 1H), 6.65–6.62 (m, 2H), 4.96 (s, 1H), 4.28–4.16 (m, 4H), 2.87 (hept, *J* = 8 Hz, 1H), 1.20 (t, *J* = 7.1 Hz, 6H), 1.02 (d, *J* = 6.9 Hz, 6H).

^{13}C NMR (101 MHz, CDCl$_3$). δ 169.6, 145.1, 129.1, 118.4, 114.9, 71.5, 62.0, 32.0, 18.3, 14.1.

6.66. *N*-(3-methyl-2-phenylbutan-2-yl)aniline **(9d)**

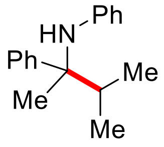

$C_{17}H_{21}N$
M (g/mol): 239.36

Following General Procedure **GP-5** for the title compound **9d** and by using **1o** (24.0 mg, 0.2 mmol, 1.0 equiv), **2a** (22.3 mg, 0.24 mmol, 1.2 equiv), *p*-TSA.H_2O (1.9 mg, 0.01 mmol, 0.05 equiv) and then DHP **5b** (118.2 mg, 0.4 mmol, 2.0 equiv) and Cs_2CO_3 (130.0 mg, 0.4 mmol, 2.0 equiv), in Acetone for 16 h at 35 °C. Purification was carried out by column chromatography (Hexane) to afford **9d** as colourless oil (24.0 mg, 50%).

HRMS (ESI): m/z $[M+H]^+$ Calculated for $[C_{17}H_{22}N]^+$: 240.1752; Found:240.1753.

^{1}H NMR (400 MHz, $CDCl_3$) δ 7.44–7.41 (m, 2H), 7.34–7.29 (m, 2H), 7.25–7.21 (m, 1H), 7.02–6.96 (m, 2H), 6.59 (t, *J* = 7.3 Hz, 1H), 6.34–6.30 (m, 2H), 4.08 (br s, 1H), 1.91 (hept, *J* = 6.9 Hz, 1H), 1.67 (s, 3H), 0.94 (d, *J* = 6.7 Hz, 3H), 0.82 (d, *J* = 6.9 Hz, 3H).

^{13}C NMR (101 MHz, $CDCl_3$) δ 146.3, 144.8, 128.8, 128.1, 127.4, 126.3, 116.9, 115.5, 61.1, 41.3, 20.4, 17.6, 17.6.

6.67. 2-methoxy-*N*-(2-methyl-1,1-diphenylpropyl)aniline **(9e)**

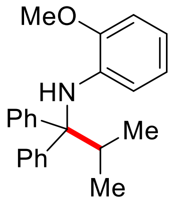

$C_{23}H_{25}NO$
M (g/mol): 331.46

Following General Procedure **GP-5** for the title compound **9e** and by using **1n** (36.4 mg, 0.2 mmol, 1.0 equiv), **2n** (22.3 mg, 0.24 mmol, 1.2 equiv), *p*-TSA.H_2O (1.9 mg, 0.01 mmol, 0.05 equiv) and then DHP **5b** (118.2 mg, 0.4 mmol, 2.0 equiv) and Cs_2CO_3 (130.0 mg, 0.4 mmol, 2.0 equiv), in Acetone for 16 h at 35 °C. Purification was carried out by column chromatography (Hexane) to afford **9e** as colourless oil (60.0 mg, 77%).

HRMS (ESI): m/z $[M+H]^+$ Calculated for $[C_{23}H_{26}NO]^+$: 332.2014; Found:332.2017.

^{1}H NMR (400 MHz, $CDCl_3$) δ 7.54–7.51 (m, 4H), 7.32–7.27 (m, 4H), 7.25–7.20 (m, 2H), 6.70 (dd, *J* = 7.8, 1.4 Hz, 1H), 6.49 (td, *J* = 7.6, 1.6 Hz, 1H), 6.41 (td, *J* = 7.7, 1.6 Hz, 1H), 6.06 (dd, *J* = 7.8, 1.8 Hz, 1H), 5.27 (s, 1H), 3.88 (s, 3H), 2.87 (heptet, *J* = 6.7 Hz, 1H), 0.87 (d, *J* = 6.8 Hz, 6H).

^{13}C NMR (101 MHz, $CDCl_3$) δ 147.2, 141.6, 136.0, 129.4, 127.5, 126.6, 120.2, 116.1, 114.5, 109.2, 68.8, 55.8, 39.6, 18.7.

6.68. *N*-(1,1,1-trifluoro-3-methyl-2-phenylbutan-2-yl)aniline **(9f)**

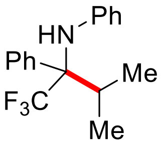

$C_{17}H_{18}F_3N$
M (g/mol): 293.33

Following General Procedure **GP-5** for the title compound **9f** and by using **1q** (35.0 mg, 0.2 mmol, 1.0 equiv), **2a** (29.5 mg, 0.24 mmol, 1.2 equiv), *p*-TSA.H_2O (1.9 mg, 0.01 mmol, 0.05 equiv) and then DHP **5b** (118.2 mg, 0.4 mmol, 2.0 equiv) and Cs_2CO_3 (130.0 mg, 0.4 mmol, 2.0 equiv), in Acetone for 16 h at 35 ºC. Purification was carried out by column chromatography (Hexane) to afford **9f** as colourless oil (51.0 mg, 91%).

HRMS (ESI): m/z $[M+H]^+$ Calculated for $[C_{17}H_{19}NF_3]^+$: 294.1470; Found:294.1460.

^{1}H NMR (400 MHz, $CDCl_3$) δ 7.55–7.52 (m, 2H), 7.40–7.32 (m, 3H), 7.07–7.01 (m, 2H), 6.74–6.70 (m, 1H), 6.49 (d, *J* = 8.1 Hz, 2H), 4.05 (s, 1H), 2.34 (hept, *J* = 7.0 Hz, 1H), 1.13 (d, *J* = 6.8 Hz, 3H), 0.86 (d, *J* = 7.0 Hz, 3H).

^{19}F NMR (376 MHz, $CDCl_3$) δ -61.29.

^{13}C NMR (101 MHz, $CDCl_3$) δ 144.9, 136.6, 128.6, 128.2, 127.9, 127.8, 127.7 (q, J =294.9 Hz), 118.9, 116.5, 69.7 (q, J = 24.2 Hz), 38.2, 17.9.

6.69 *N*-benzyl-2-methyl-1,1-diphenylpropan-1-amine **(9g)**

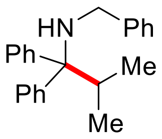

$C_{23}H_{25}N$
M (g/mol): 315.46

Following General Procedure **GP-5** for the title compound **9g** and by using **1n** (36.4 mg, 0.2 mmol, 1.0 equiv), **2o** (25.7 mg, 0.24 mmol, 1.2 equiv), *p*-TSA.H_2O (1.9 mg, 0.01 mmol, 0.05 equiv) and then DHP **5b** (118.2 mg, 0.4 mmol, 2.0 equiv) and Cs_2CO_3 (130.0 mg, 0.4 mmol, 2.0 equiv), in Acetone for 16 h at 35 ºC. Purification was carried out by column chromatography (Hexane) to afford **9g** as colourless oil (30.0 mg, 48%).

HRMS (ESI): m/z $[M+Na]^+$ Calculated for $[C_{23}H_{25}NNa]^+$: 338.1885; Found:338.1830.

^{1}H NMR (400 MHz, $CDCl_3$) δ 7.42–7.39 (m, 3H), 7.33–7.27 (m, 4H), 7.26–7.24 (m, 5H), 7.23–7.13 (m, 3H), 3.25 (s, 2H), 2.80 (hept, *J* = 6.7 Hz, 1H), 0.88 (d, *J* = 6.5 Hz, 6H).

^{13}C NMR (101 MHz, $CDCl_3$) δ 143.5, 141.4, 129.6, 128.4, 128.2, 127.3, 126.8, 126.4, 69.6, 47.1, 32.1, 18.4.

7. NMR Spectra:

7.1. Methyl 5,5-difluoro-2,4-diphenyl-2-(phenylamino)pent-4-enoate (4a)

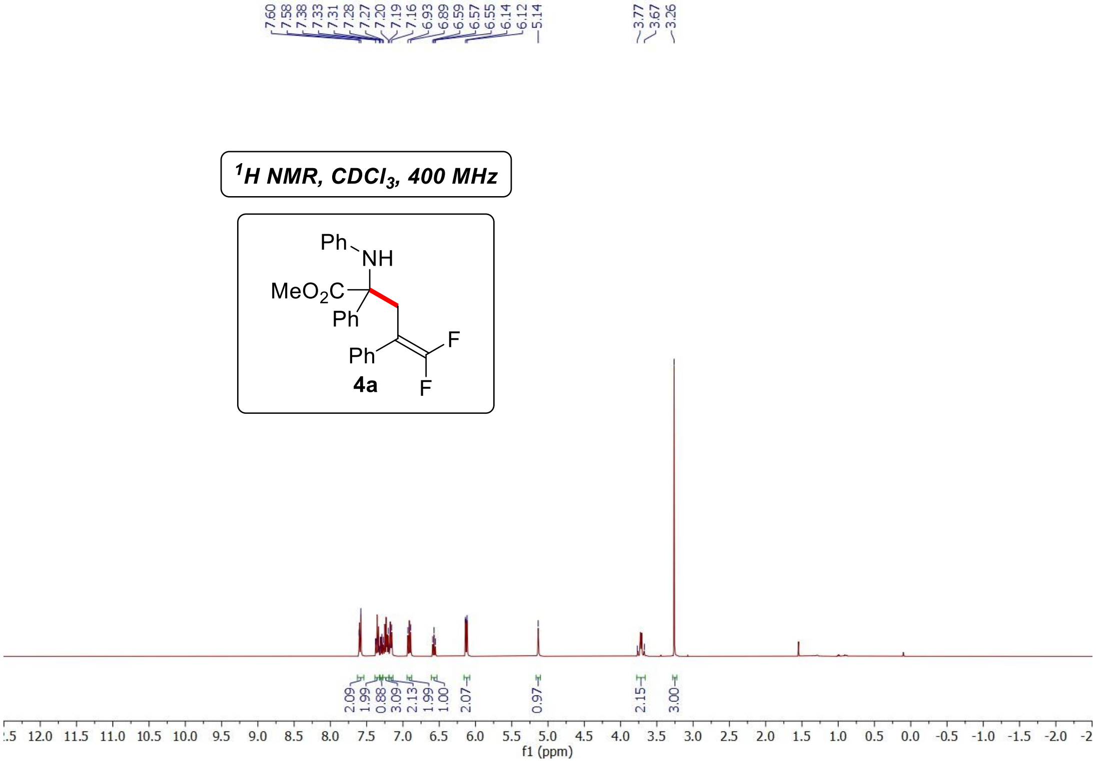

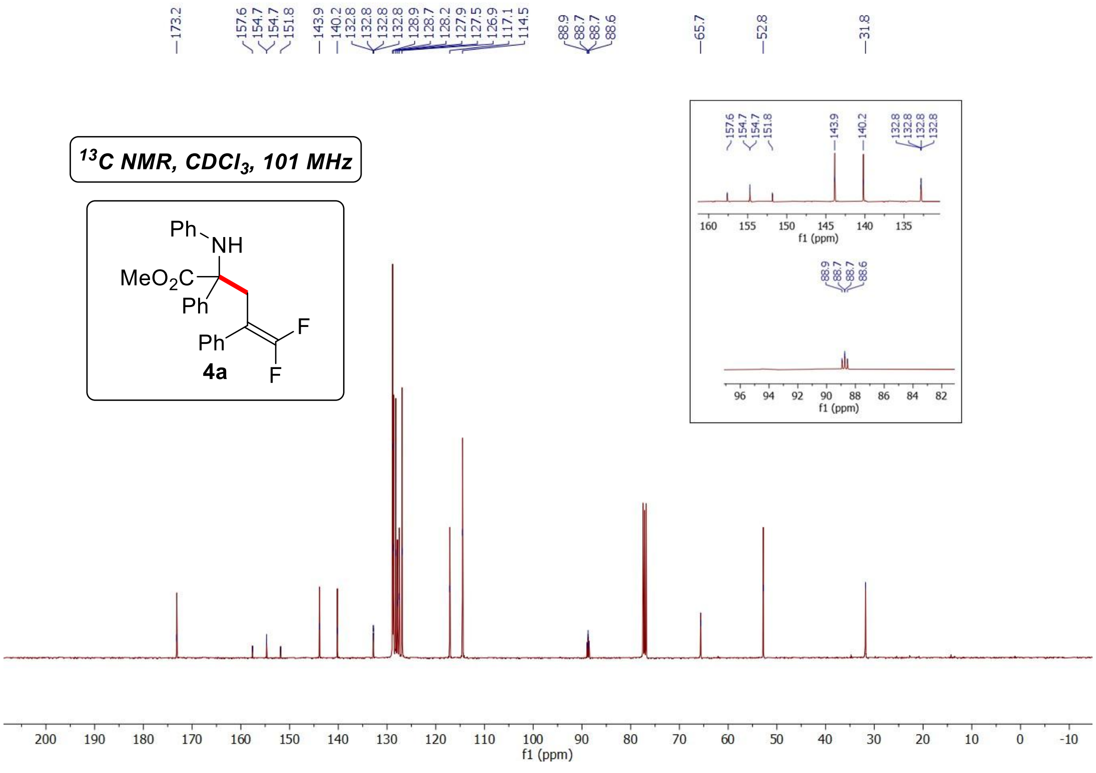

^{19}F NMR, $CDCl_3$, 376 MHz

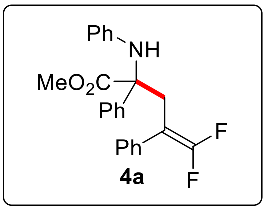

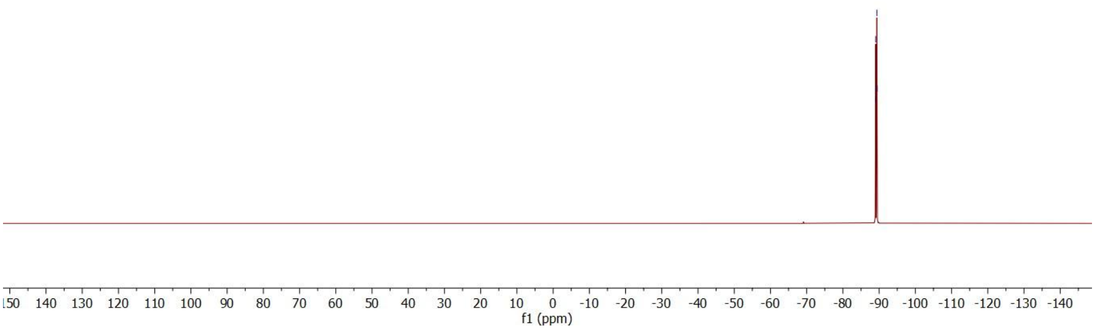

7.2. Ethyl 5,5-difluoro-2,4-diphenyl-2-(phenylamino)pent-4-enoate (4b)

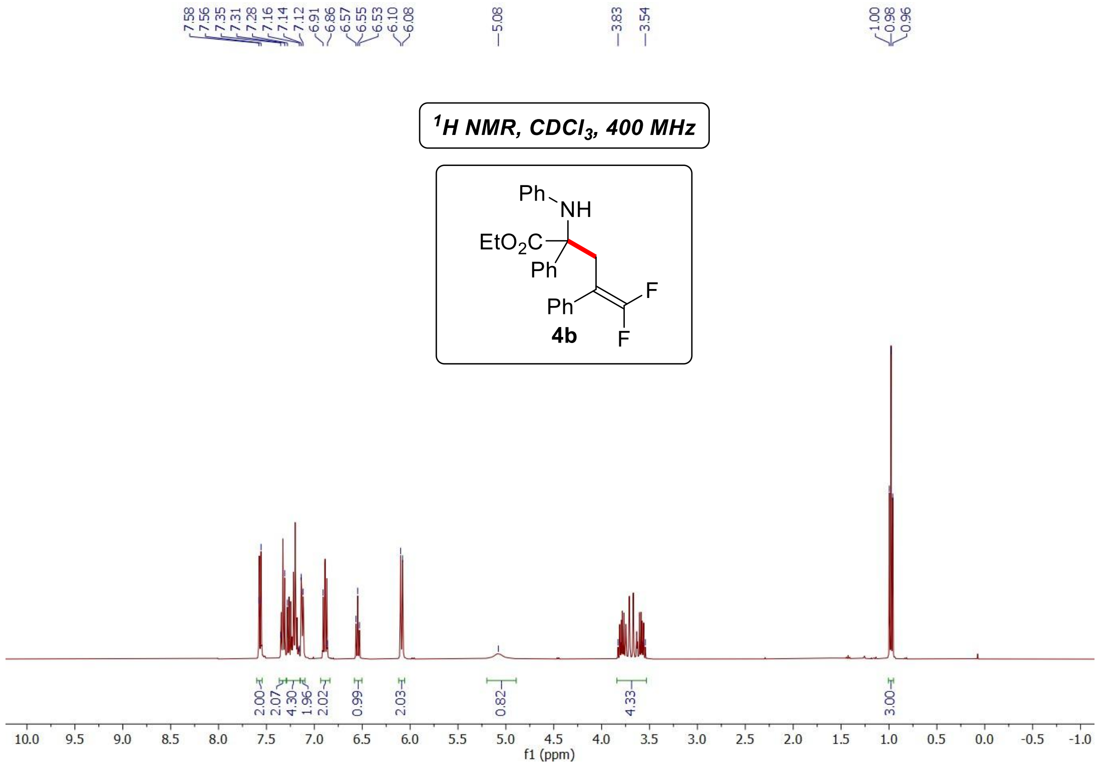

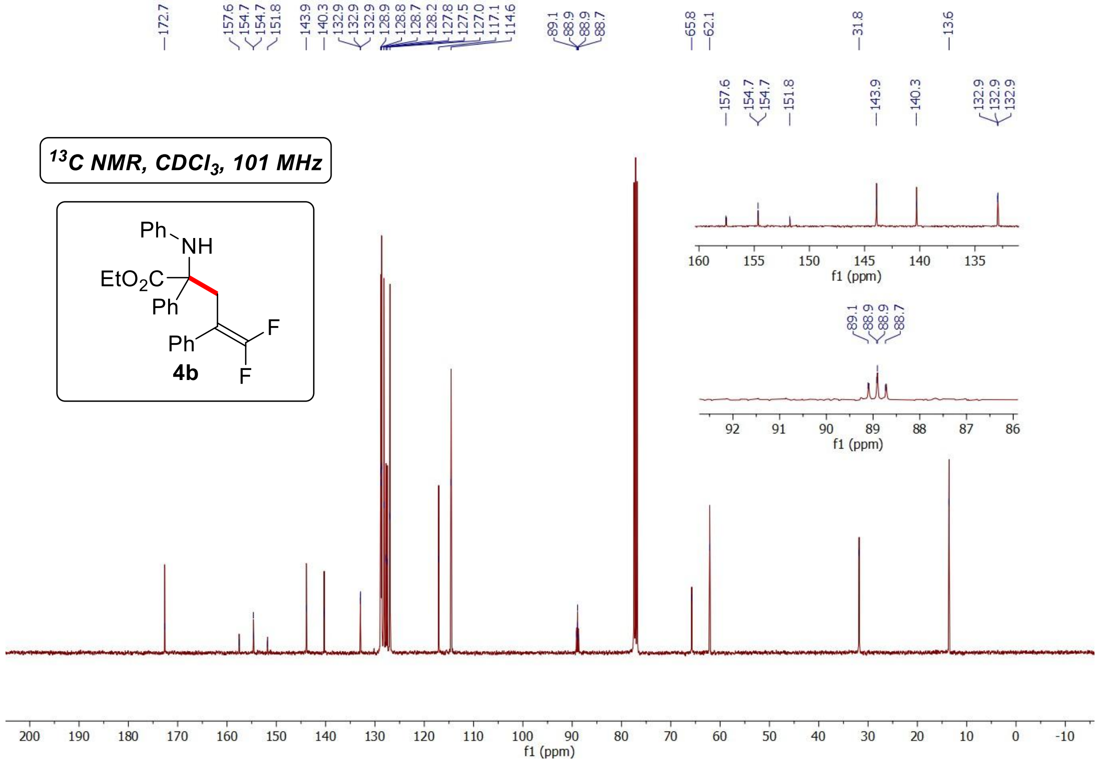

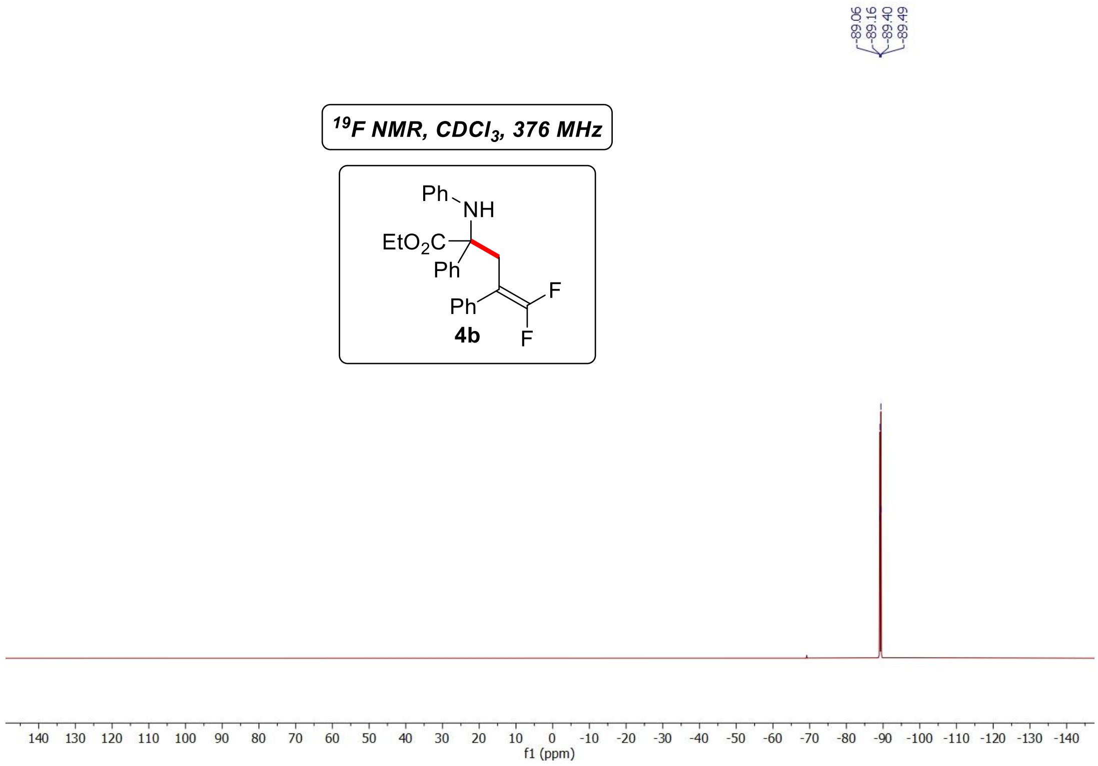
-89.06
-89.16
-89.40
-89.49
19F NMR, CDCl3, 376 MHz
Ph
NH
EtO2C
Ph
Ph
F
F
4b
140 130 120 110 100 90 80 70 60 50 40 30 20 10 0 -10 -20 -30 -40 -50 -60 -70 -80 -90 -100 -110 -120 -130 -140
f1 (ppm)

7.3. Isopropyl 5,5-difluoro-2,4-diphenyl-2-(phenylamino)pent-4-enoate (4c)

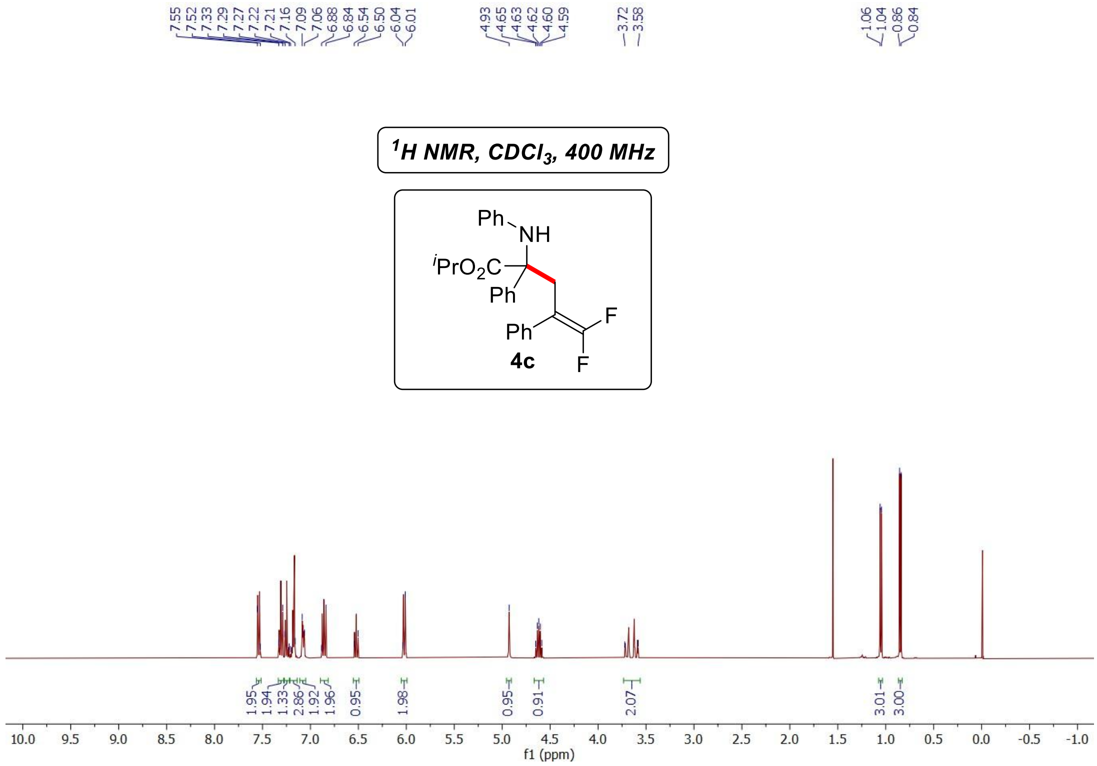

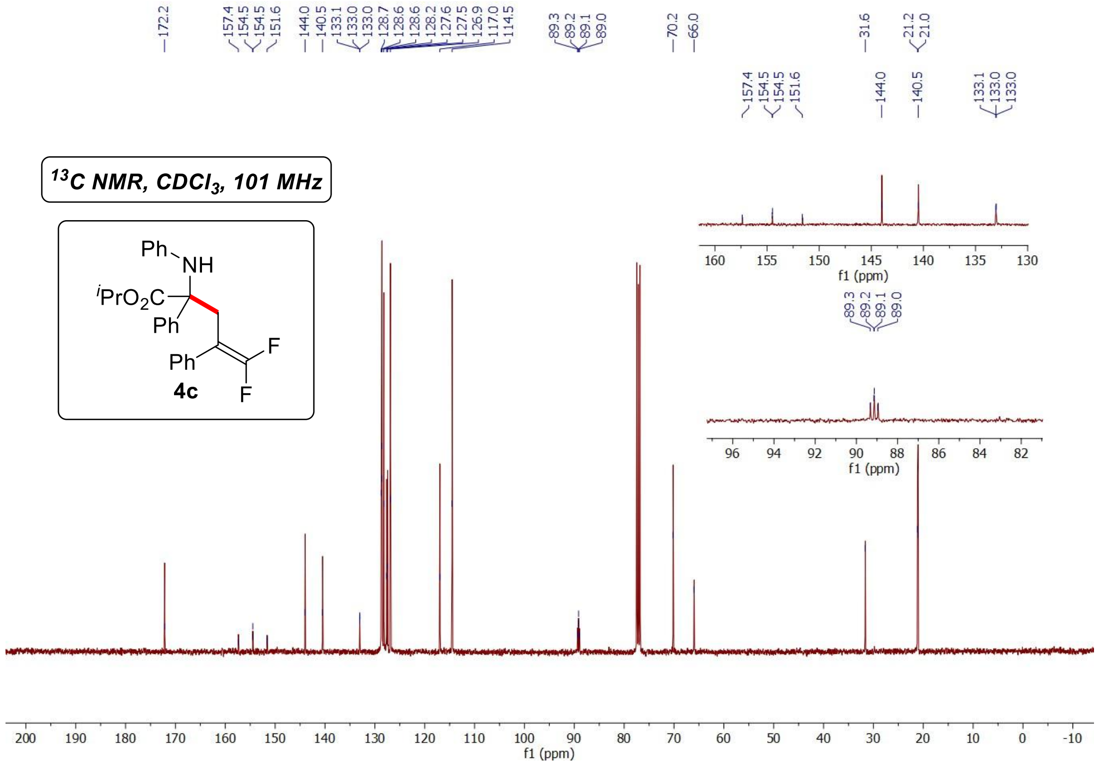

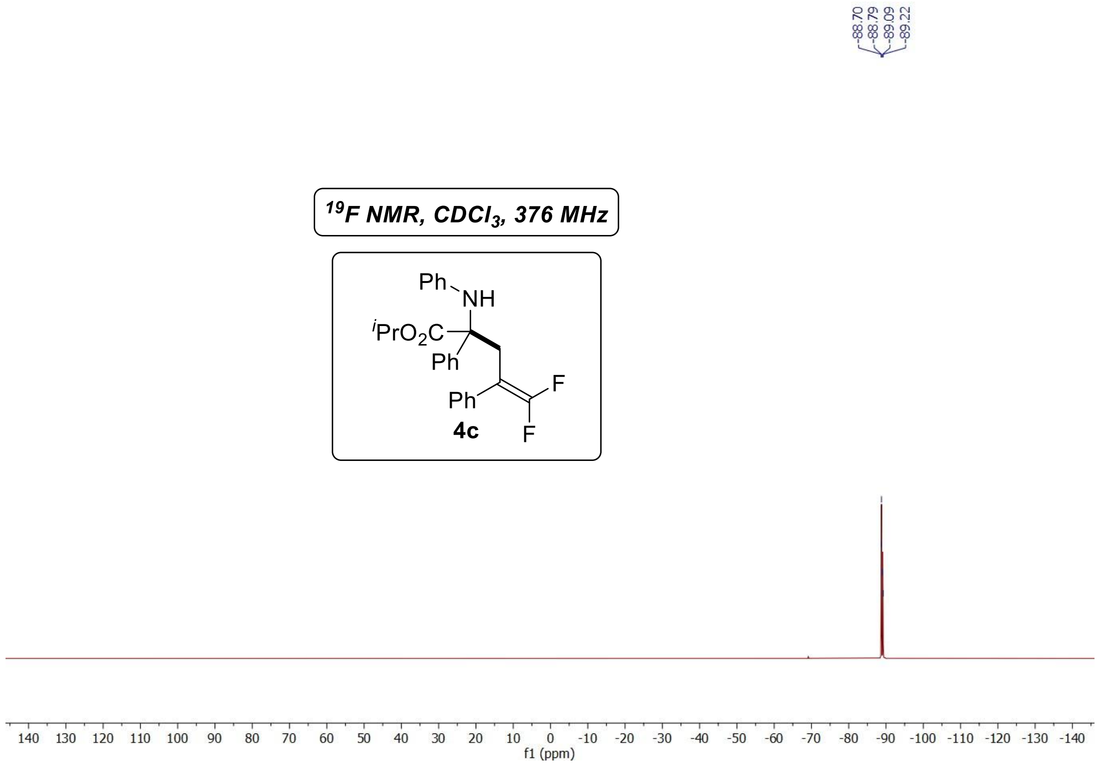
-88.70
-88.79
-89.09
-89.22
19F NMR, CDCl3, 376 MHz
Ph
NH
iPrO2C
Ph
Ph
F
F
4c
140 130 120 110 100 90 80 70 60 50 40 30 20 10 0 -10 -20 -30 -40 -50 -60 -70 -80 -90 -100 -110 -120 -130 -140
f1 (ppm)

7.4. Benzyl 5,5-difluoro-2,4-diphenyl-2-(phenylamino)pent-4-enoate (4d)

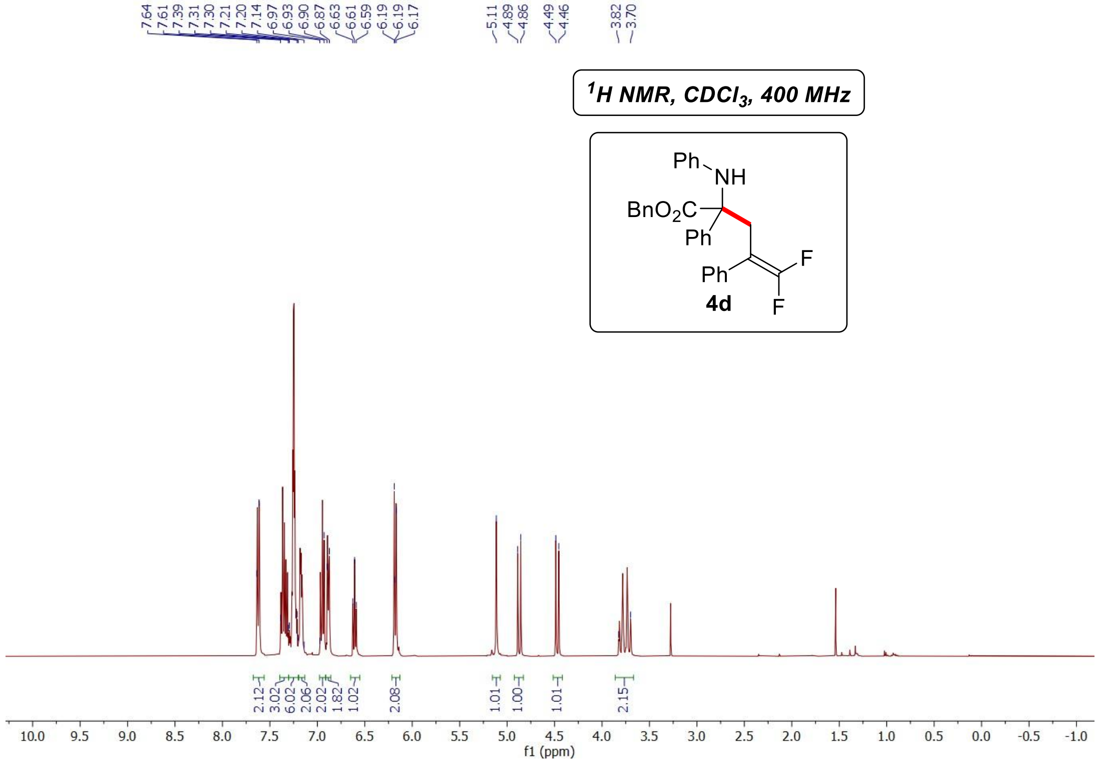

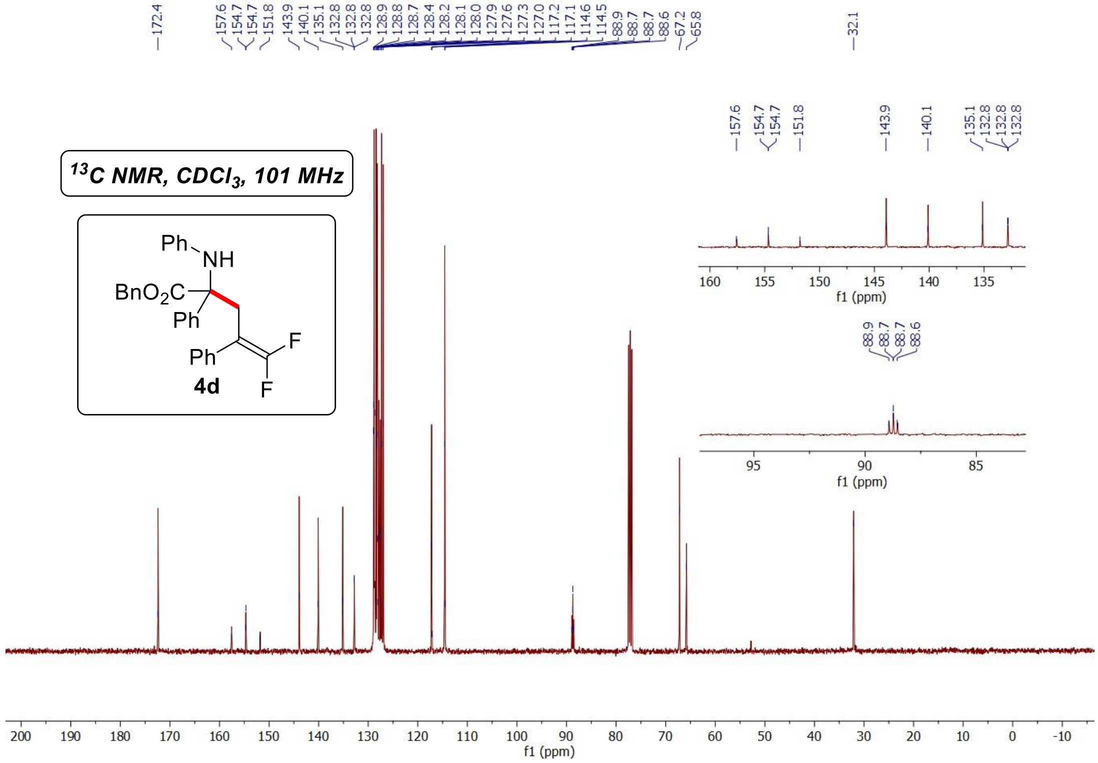

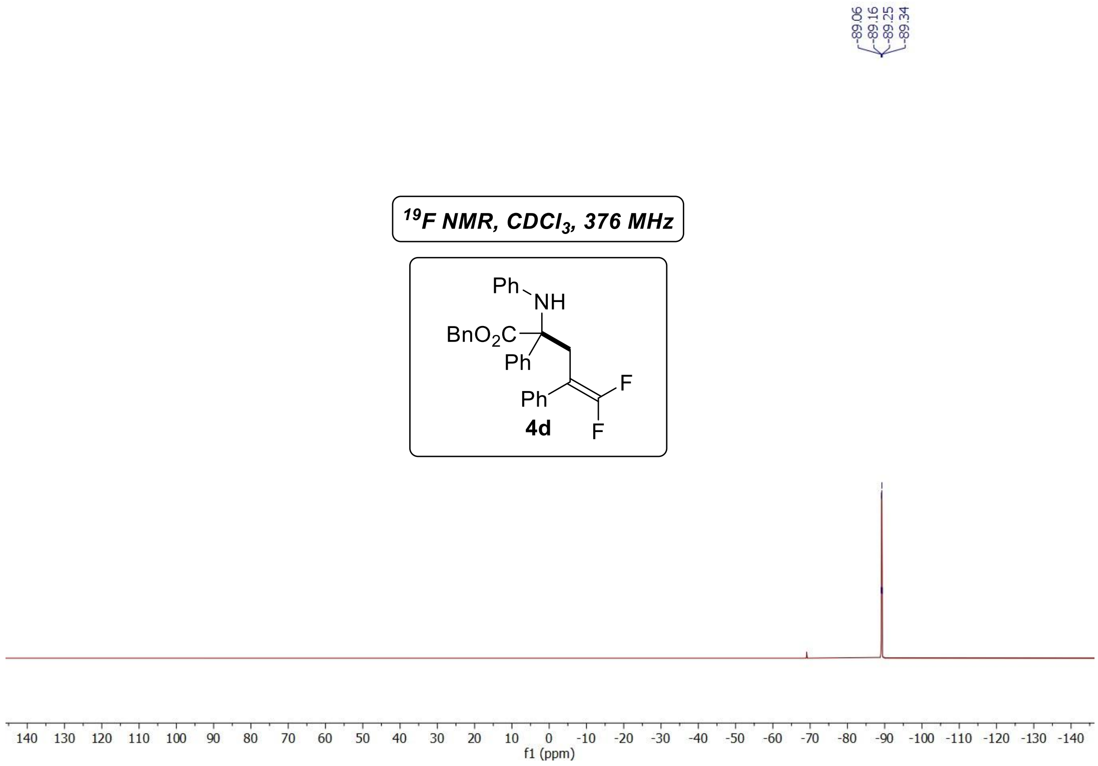

-89.06
-89.16
-89.25
-89.34
19F NMR, CDCl3, 376 MHz
Ph
NH
BnO2C
Ph
Ph
F
F
4d
140 130 120 110 100 90 80 70 60 50 40 30 20 10 0 -10 -20 -30 -40 -50 -60 -70 -80 -90 -100 -110 -120 -130 -140
f1 (ppm)

7.5. Prop-2-yn-1-yl 5,5-difluoro-2,4-diphenyl-2-(phenylamino)pent-4-enoate (4e)

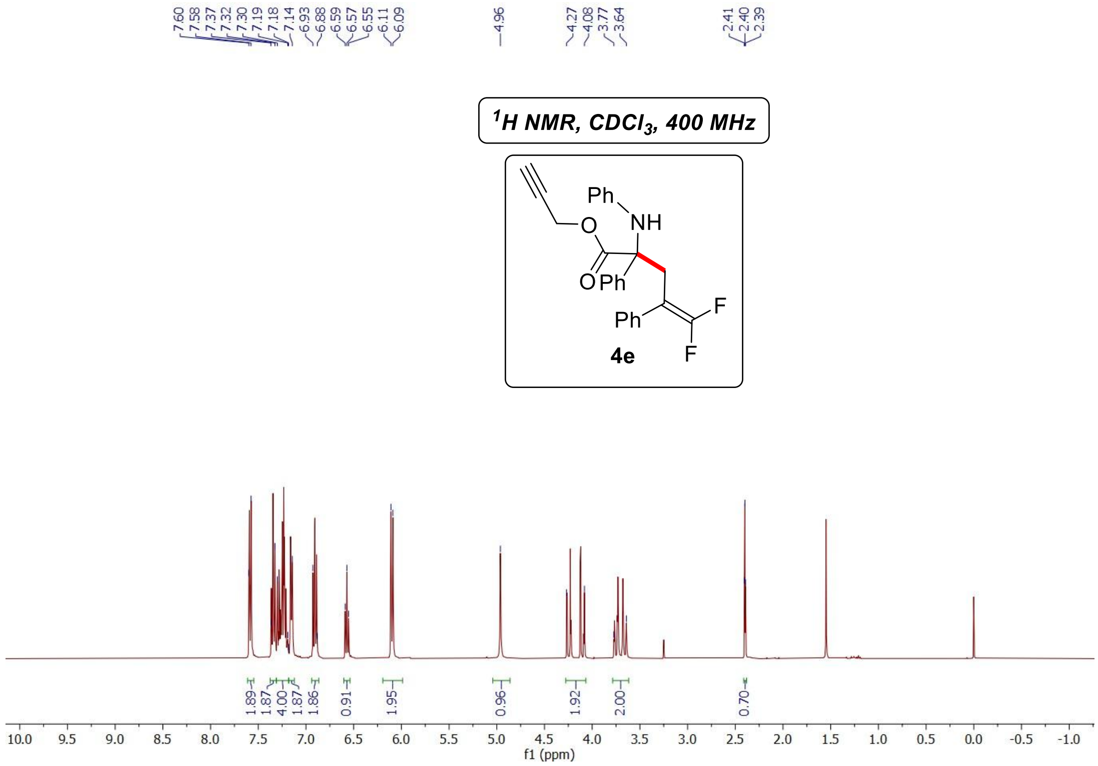

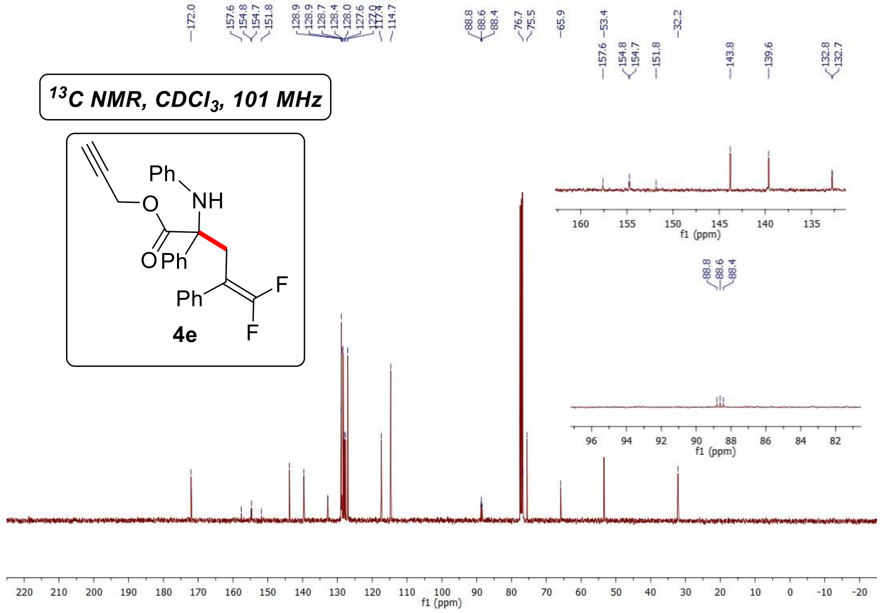

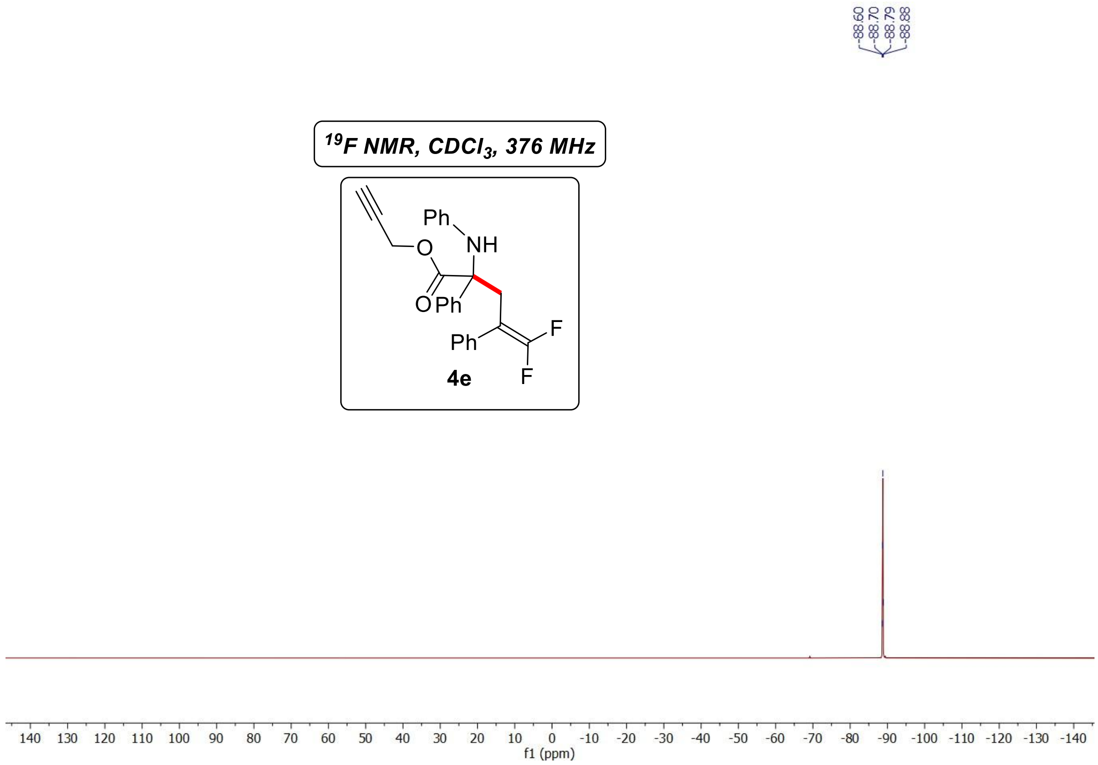
-88.60
-88.70
-88.79
-88.88
19F NMR, CDCl3, 376 MHz
Ph
NH
O
O
Ph
Ph
F
F
4e
140 130 120 110 100 90 80 70 60 50 40 30 20 10 0 -10 -20 -30 -40 -50 -60 -70 -80 -90 -100 -110 -120 -130 -140
f1 (ppm)

7.6. But-3-en-1-yl 5,5-difluoro-2,4-diphenyl-2-(phenylamino)pent-4-enoate (4f)

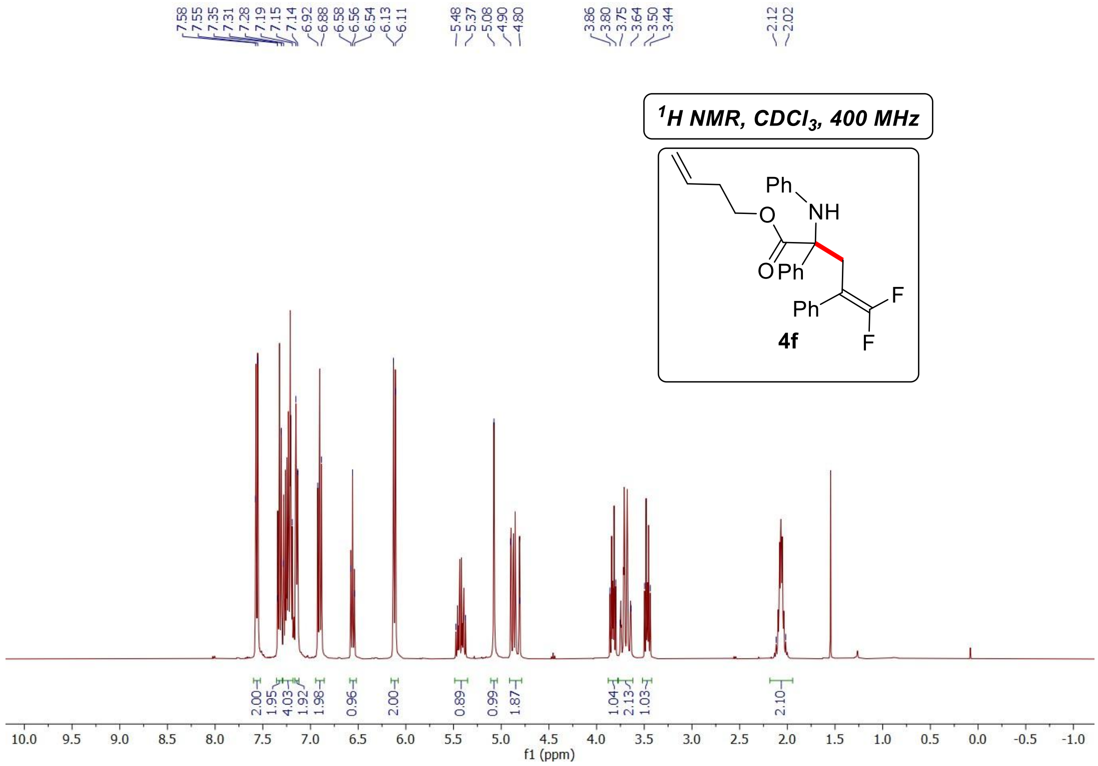

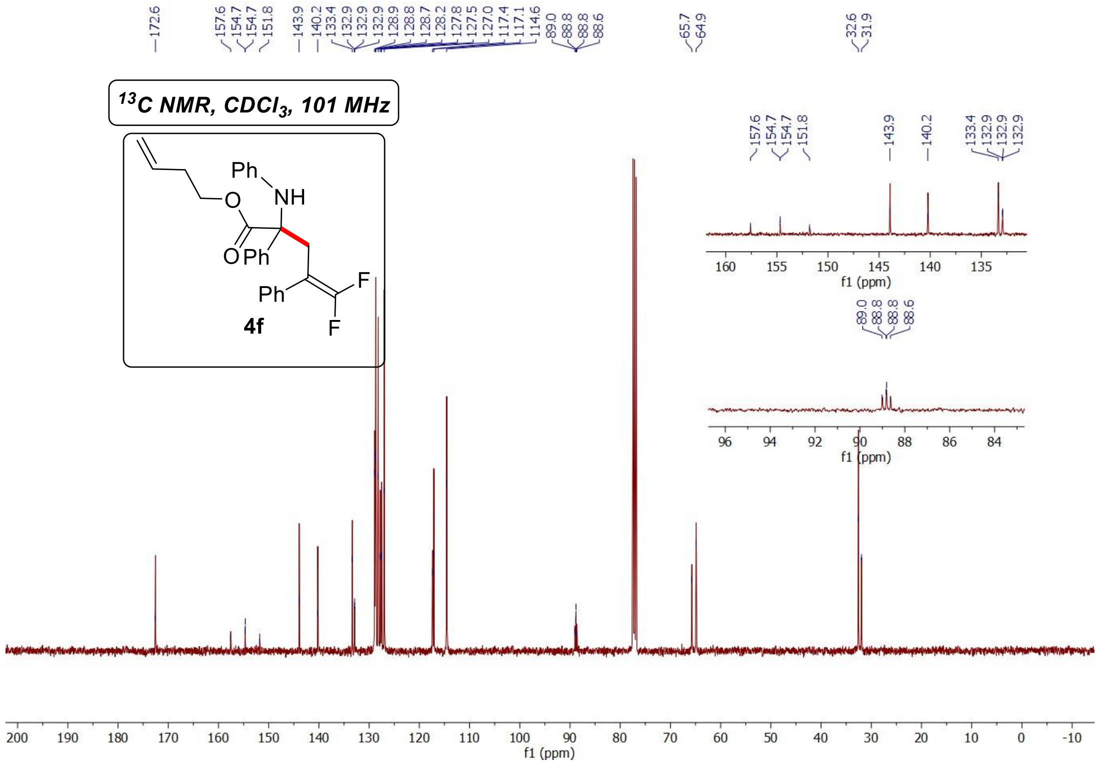

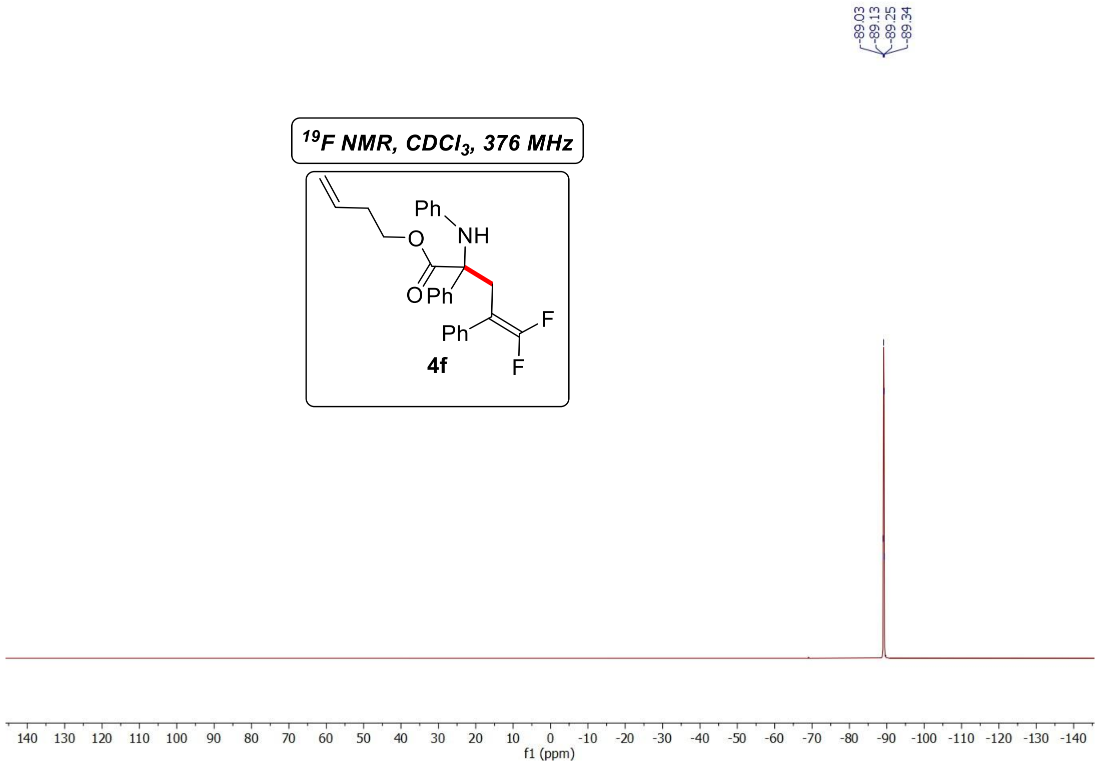

-89.03
-89.13
-89.25
-89.34
^{19}F NMR, CDCl$_3$, 376 MHz
Ph
NH
O
O
Ph
Ph
F
F
4f
140 130 120 110 100 90 80 70 60 50 40 30 20 10 0 -10 -20 -30 -40 -50 -60 -70 -80 -90 -100 -110 -120 -130 -140
f1 (ppm)

7.7. (3S,8S,9S,10R,13R,14S,16S)-10,13-dimethyl-16-((S)-6-methylheptan-2-yl)-2,3,4,7,8,9,10,11,12,13,14,15,16,17-tetradecahydro-1H-cyclopenta[a]phenanthren-3-yl 5,5-difluoro-2-((4-methoxyphenyl)amino)-2,4-diphenylpent-4-enoate (4g)

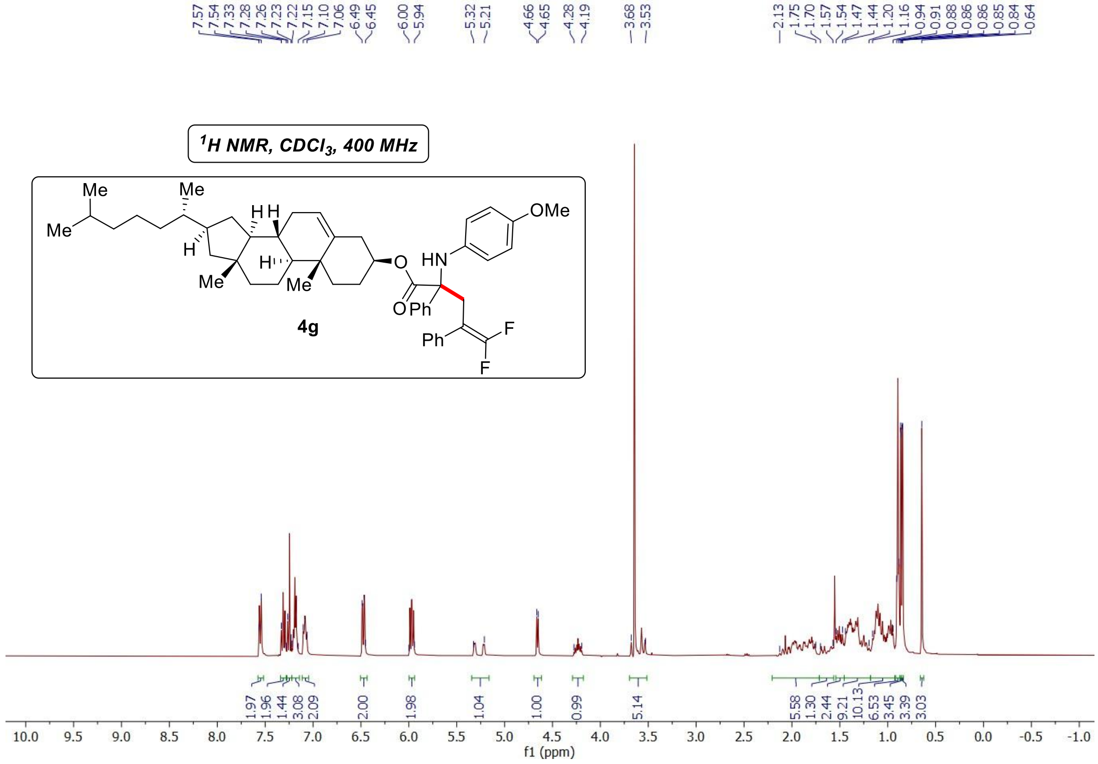

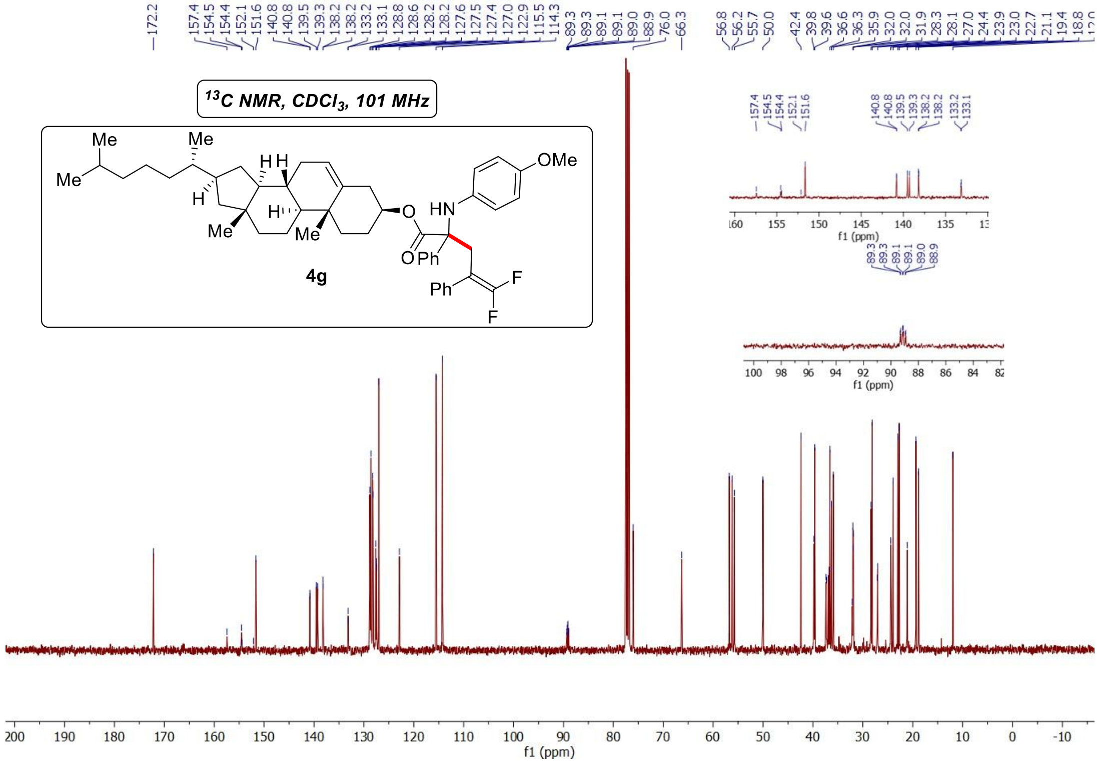

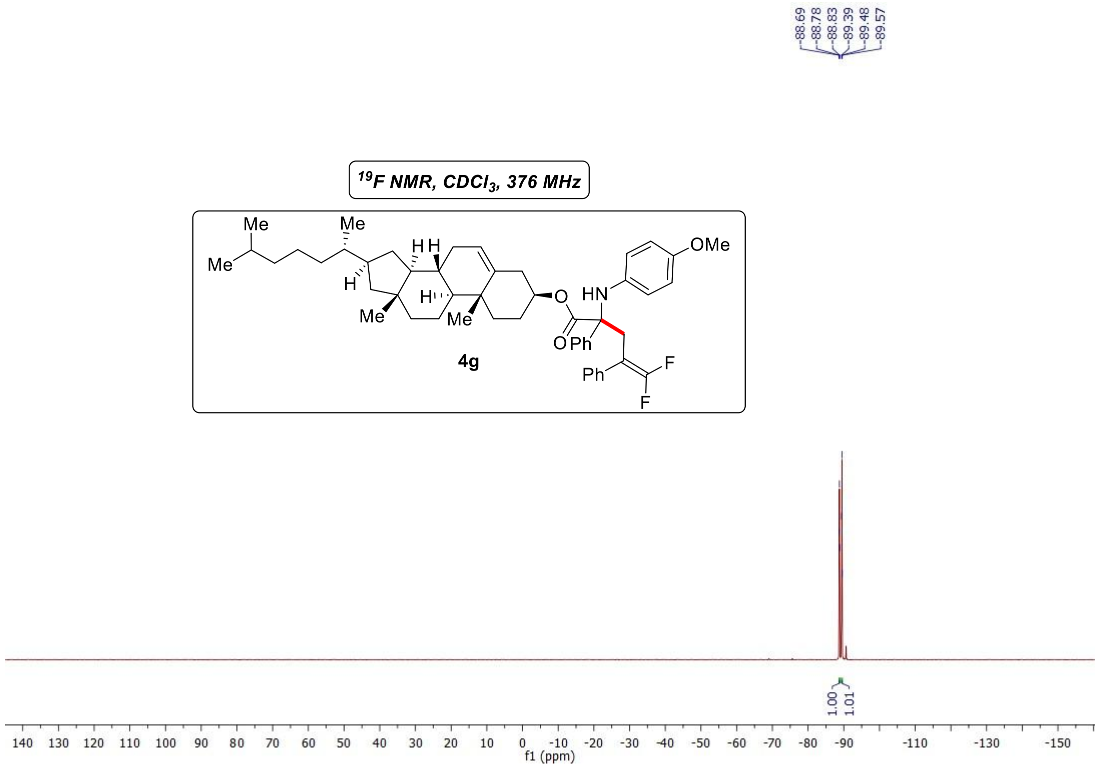
-88.69
-88.78
-88.83
-89.39
-89.48
-89.57
19F NMR, CDCl3, 376 MHz
Me
Me
Me
H
H
H
H
Me
Me
O
HN
OMe
O
Ph
Ph
F
F
4g
1.00
1.01
140 130 120 110 100 90 80 70 60 50 40 30 20 10 0 -10 -20 -30 -40 -50 -60 -70 -80 -90 -110 -130 -150
f1 (ppm)

7.8. Methyl 2-(4-bromophenyl)-5,5-difluoro-4-phenyl-2-(phenylamino)pent-4-enoate (4h)

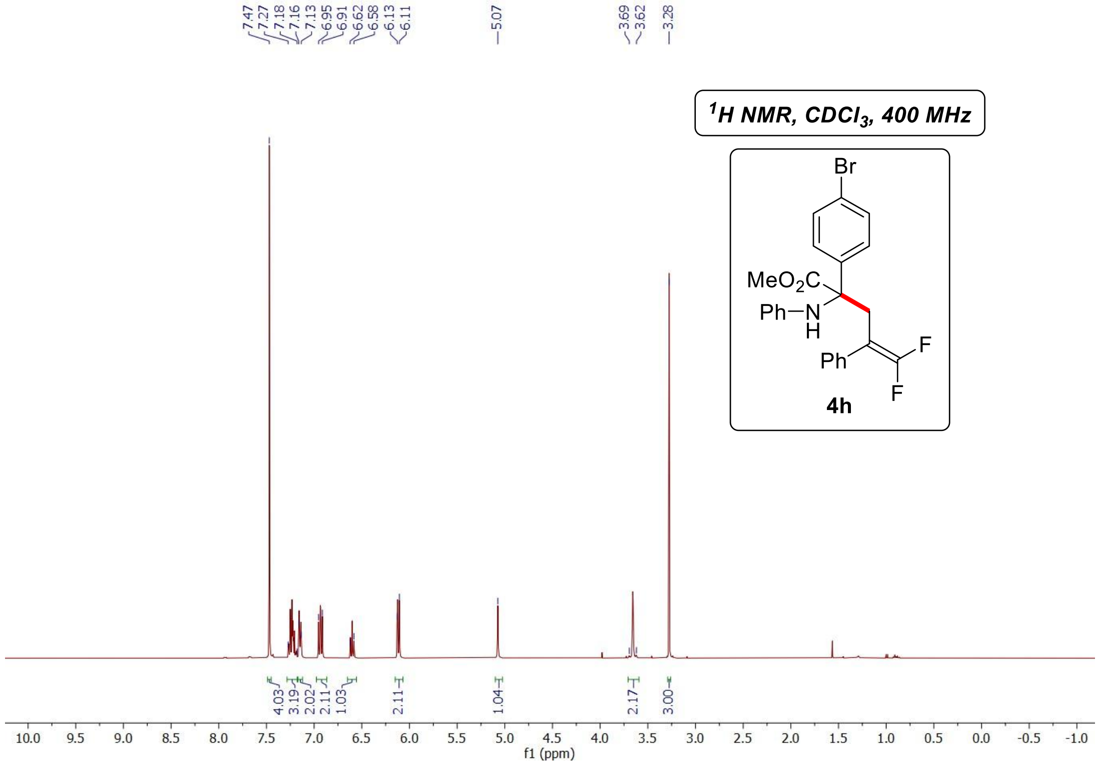

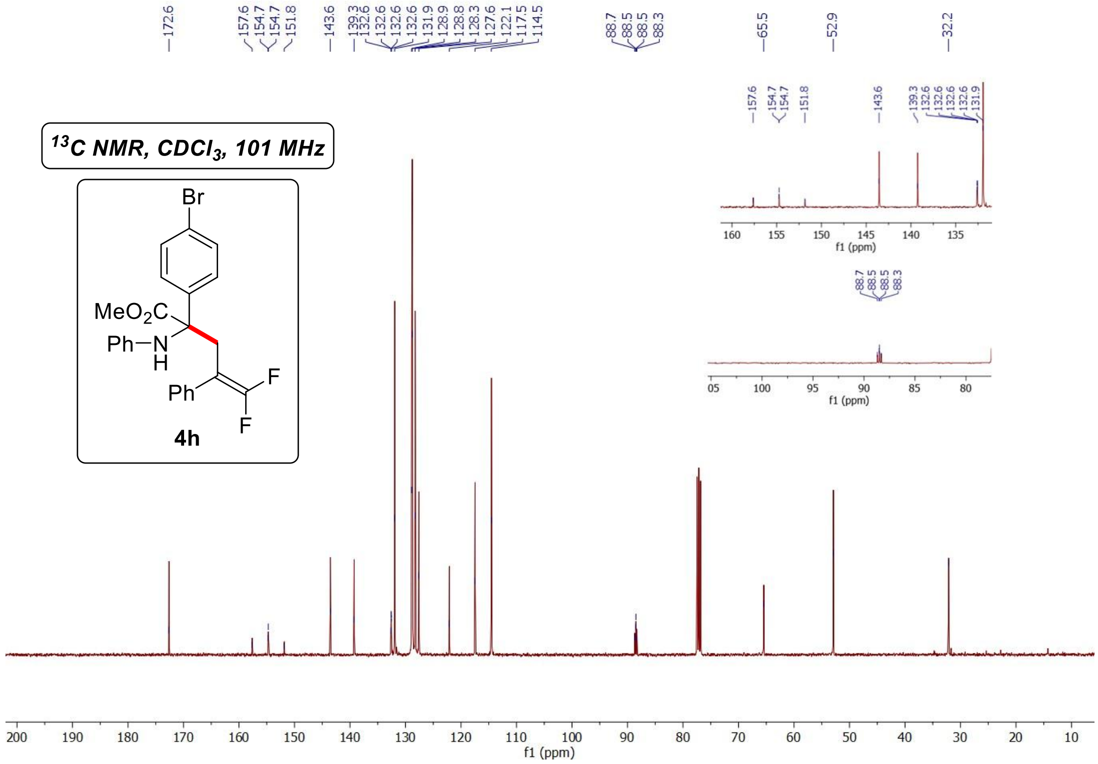

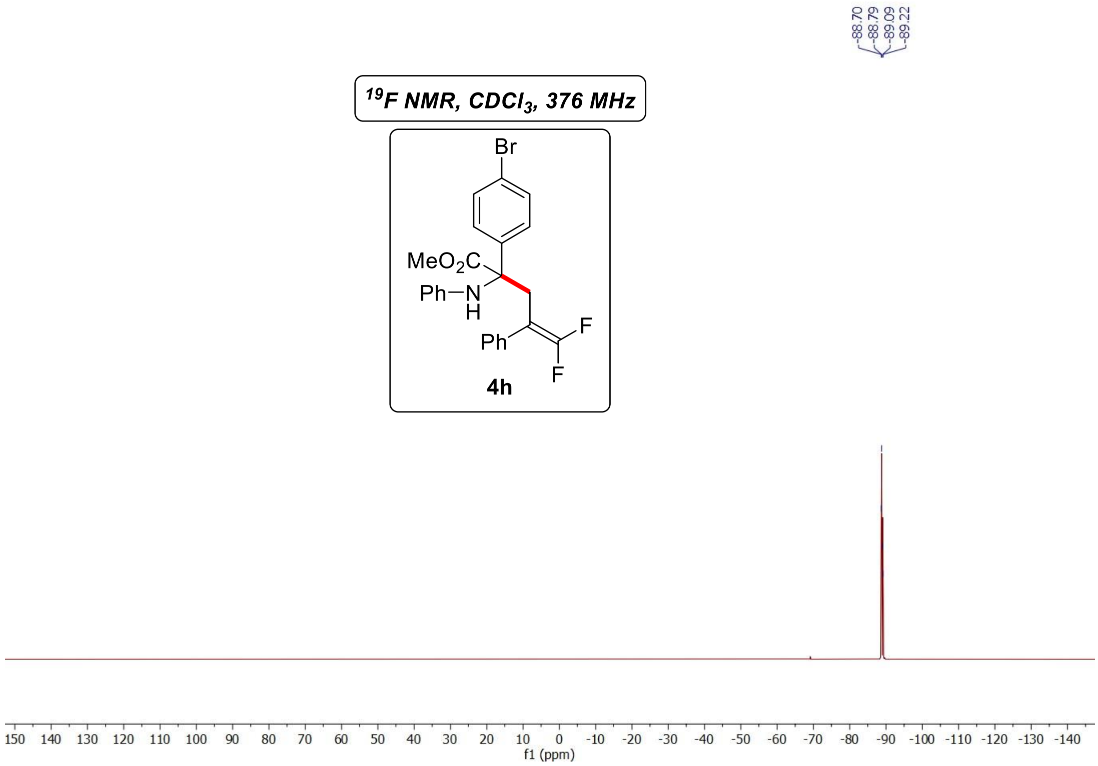
-88.70
-88.79
-89.09
-89.22
19F NMR, CDCl3, 376 MHz
Br
MeO2C
Ph—N
H
Ph
F
F
4h
150 140 130 120 110 100 90 80 70 60 50 40 30 20 10 0 -10 -20 -30 -40 -50 -60 -70 -80 -90 -100 -110 -120 -130 -140
f1 (ppm)

7.9. Methyl 5,5-difluoro-4-phenyl-2-(phenylamino)-2-(p-tolyl)pent-4-enoate (4i)

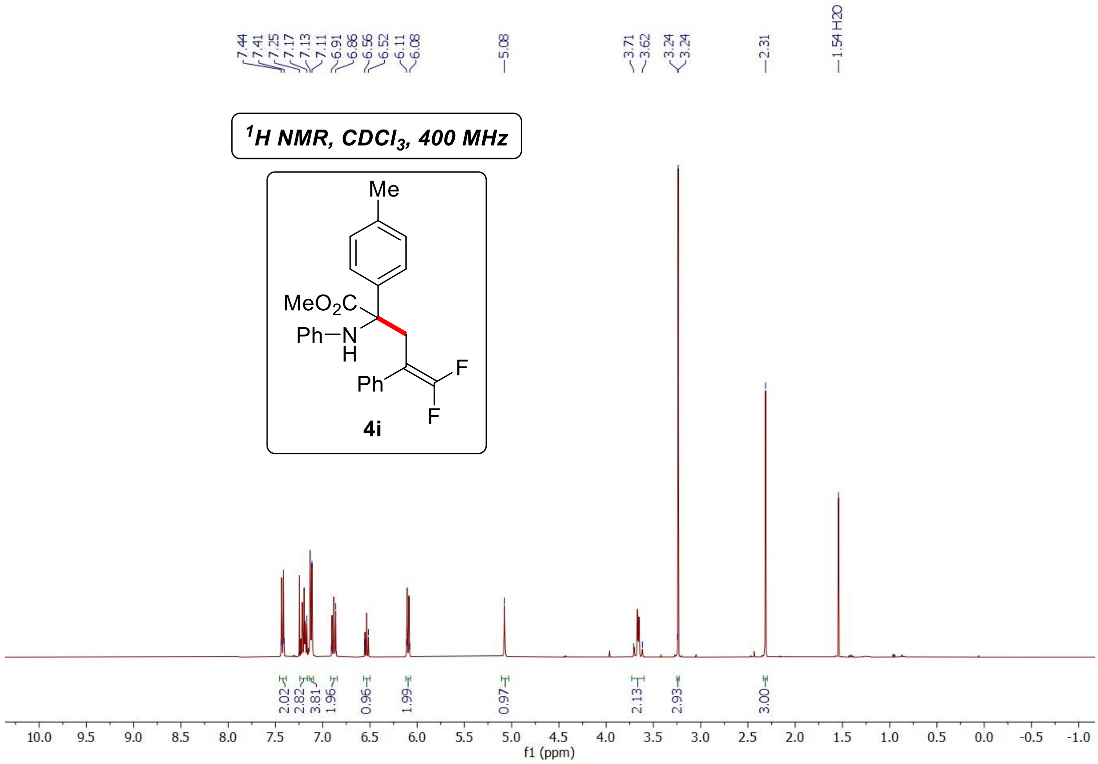

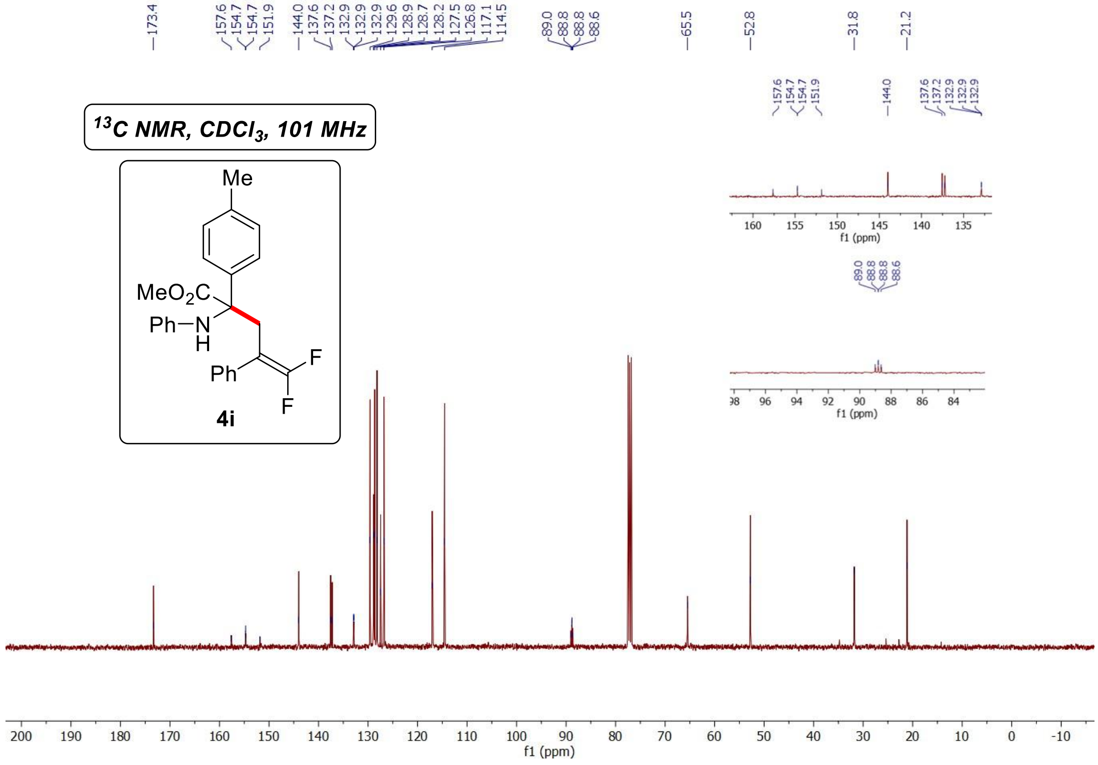

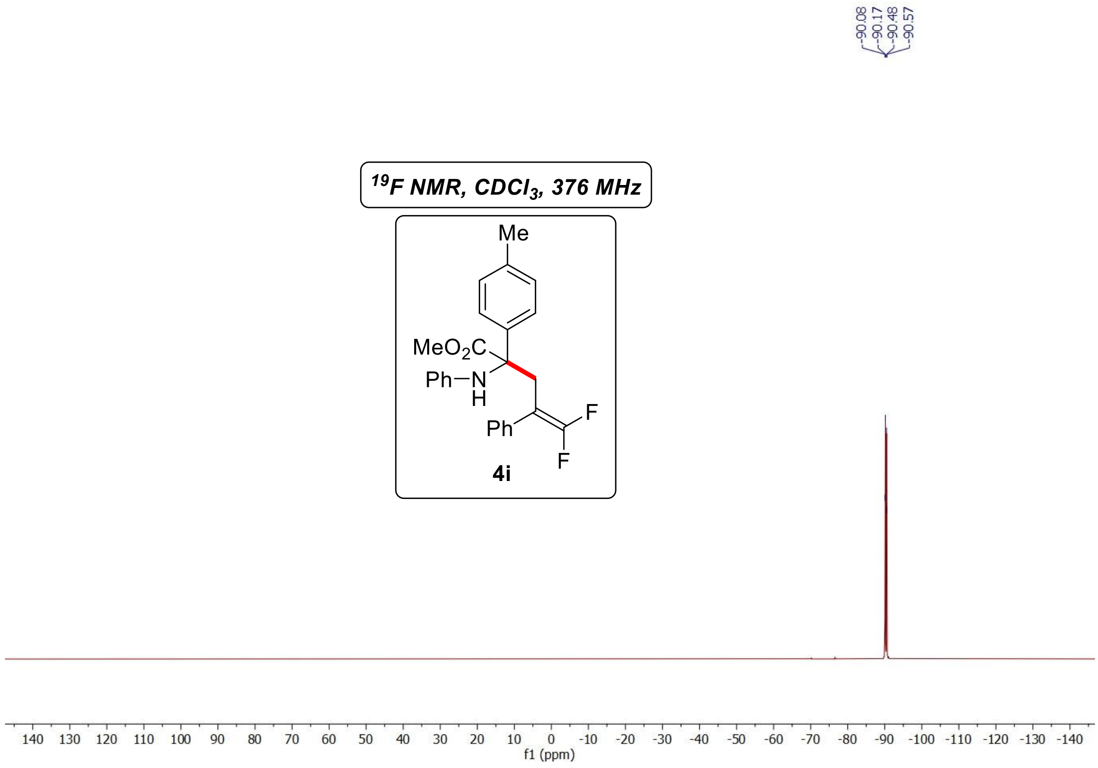

-90.08
-90.17
-90.48
-90.57
19F NMR, CDCl3, 376 MHz
Me
MeO2C
Ph−N
H
Ph
F
F
4i
140 130 120 110 100 90 80 70 60 50 40 30 20 10 0 -10 -20 -30 -40 -50 -60 -70 -80 -90 -100 -110 -120 -130 -140
f1 (ppm)

7.10. Methyl 5,5-difluoro-2-(4-methoxyphenyl)-4-phenyl-2-(phenylamino)pent-4-enoate (4j)

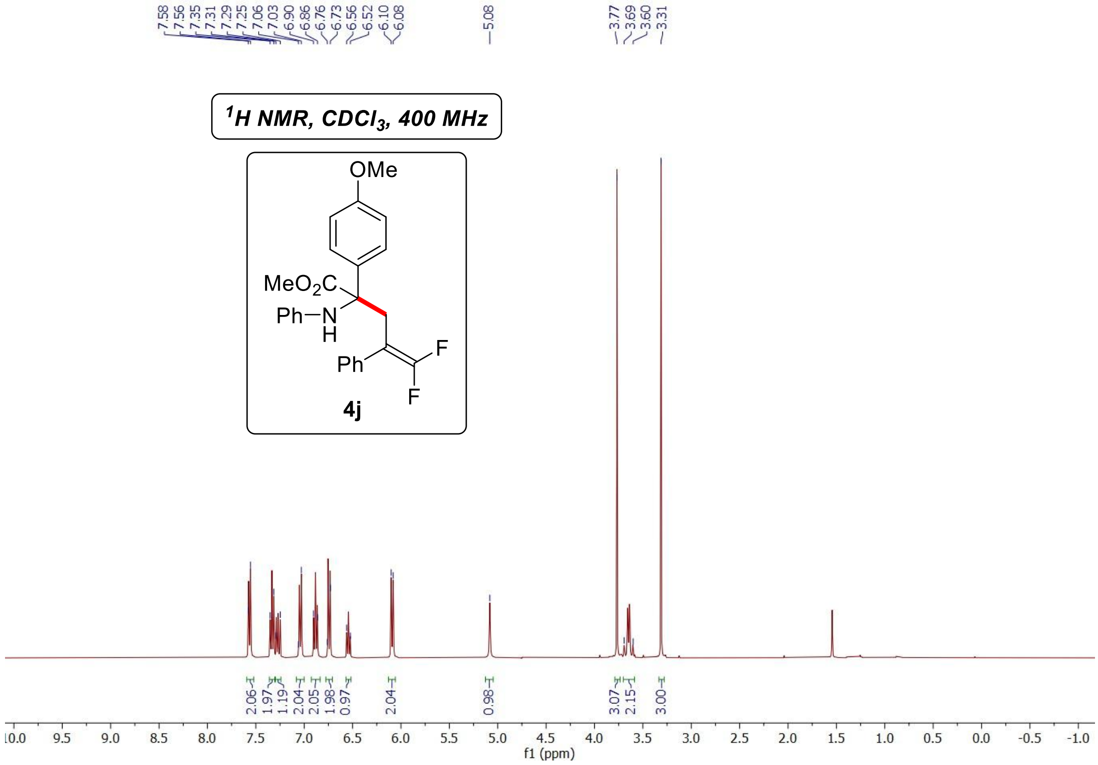

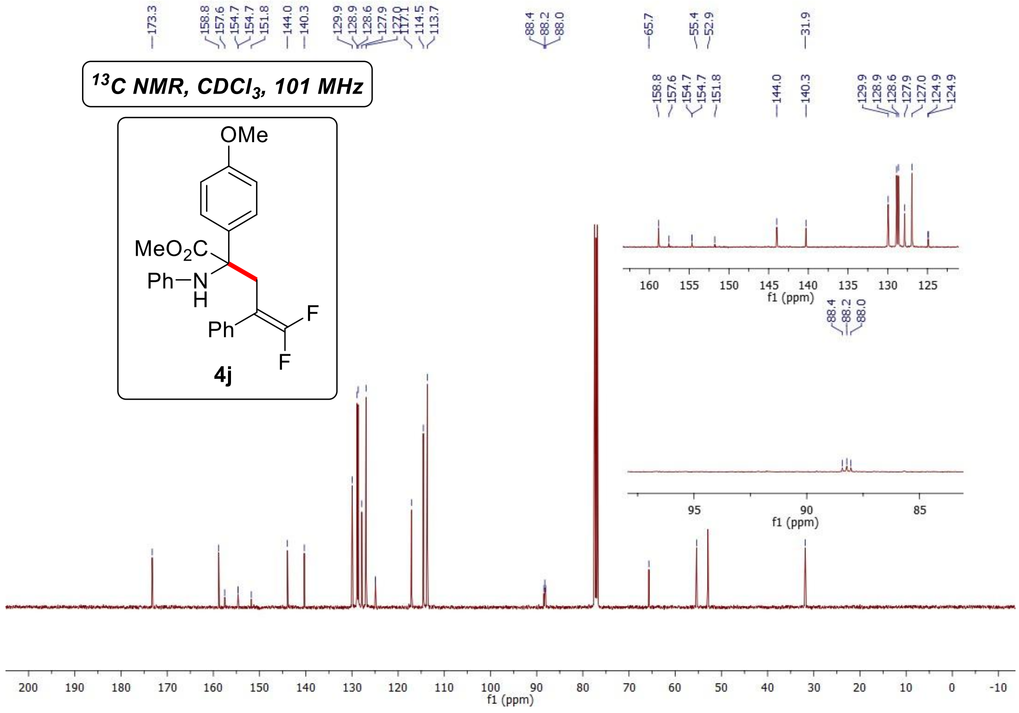

^{19}F NMR, CDCl$_3$, 376 MHz

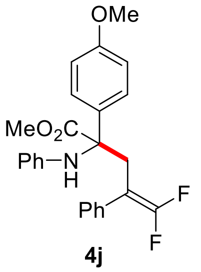

4j

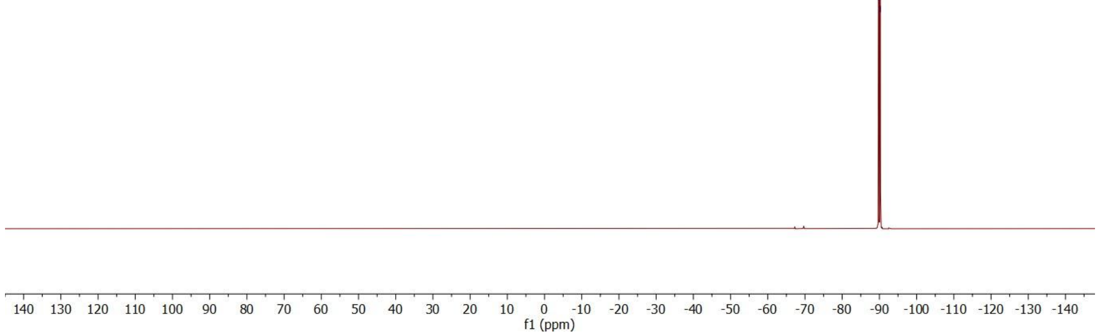

7.11. Methyl 5,5-difluoro-4-phenyl-2-(phenylamino)-2-(thiophen-2-yl)pent-4-enoate(4k)

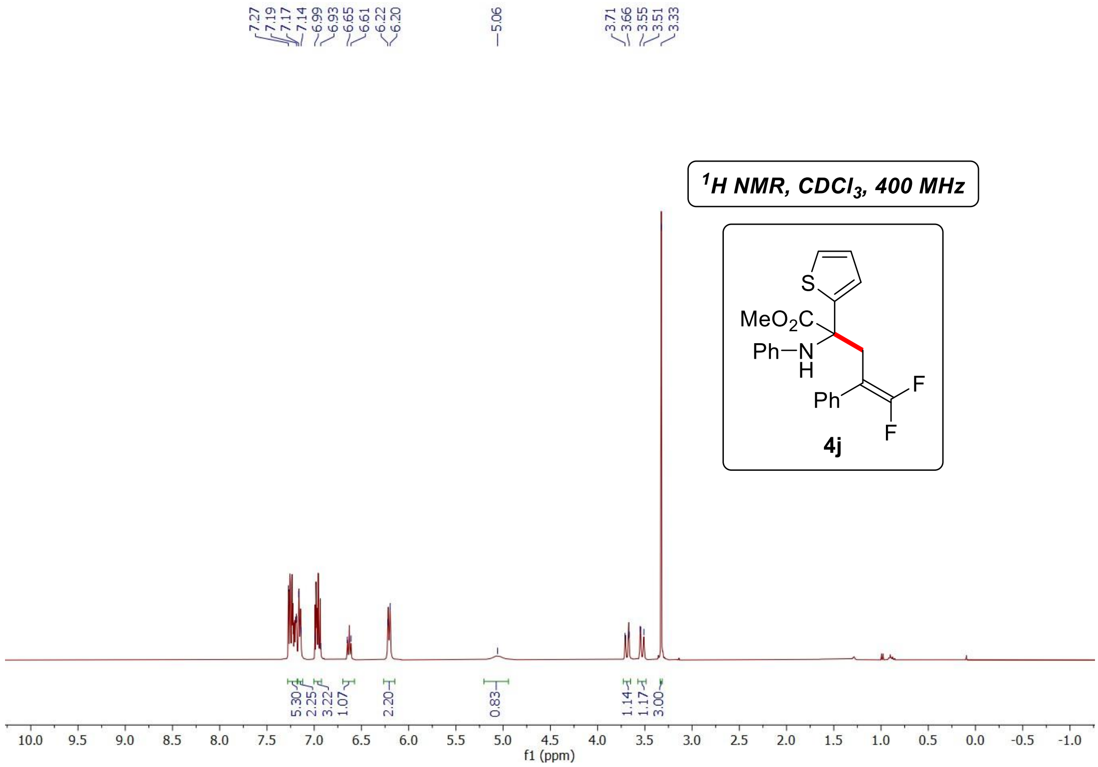

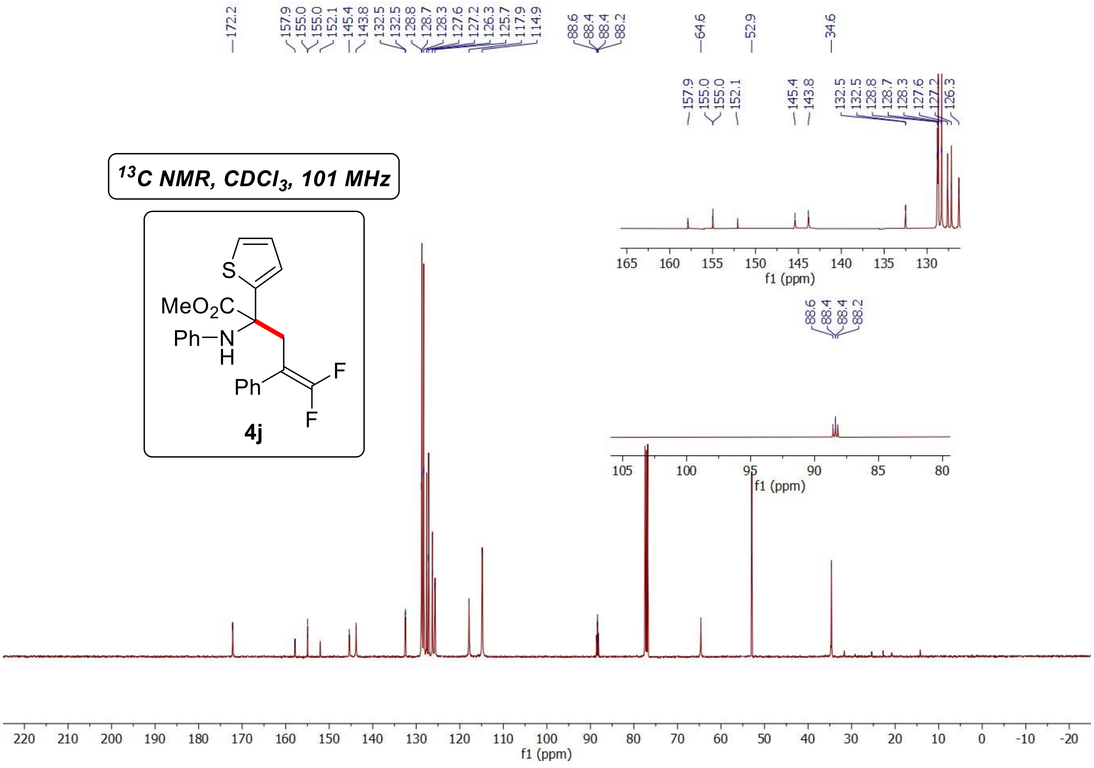

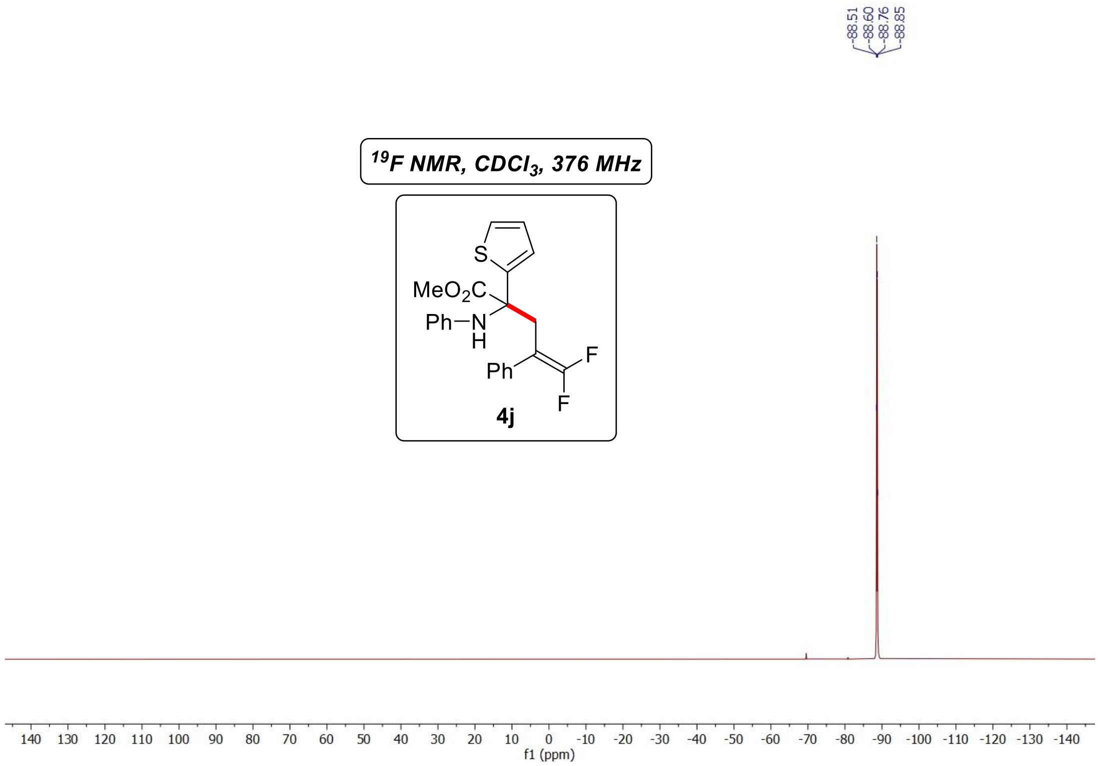
-88.51
-88.60
-88.76
-88.85
19F NMR, CDCl3, 376 MHz
MeO2C
Ph−N
H
S
Ph
F
F
4j
140 130 120 110 100 90 80 70 60 50 40 30 20 10 0 -10 -20 -30 -40 -50 -60 -70 -80 -90 -100 -110 -120 -130 -140
f1 (ppm)

7.12. Methyl 5,5-difluoro-4-phenyl-2-(phenylamino)-2-(4-(trifluoromethyl)phenyl)pent-4-enoate (4l)

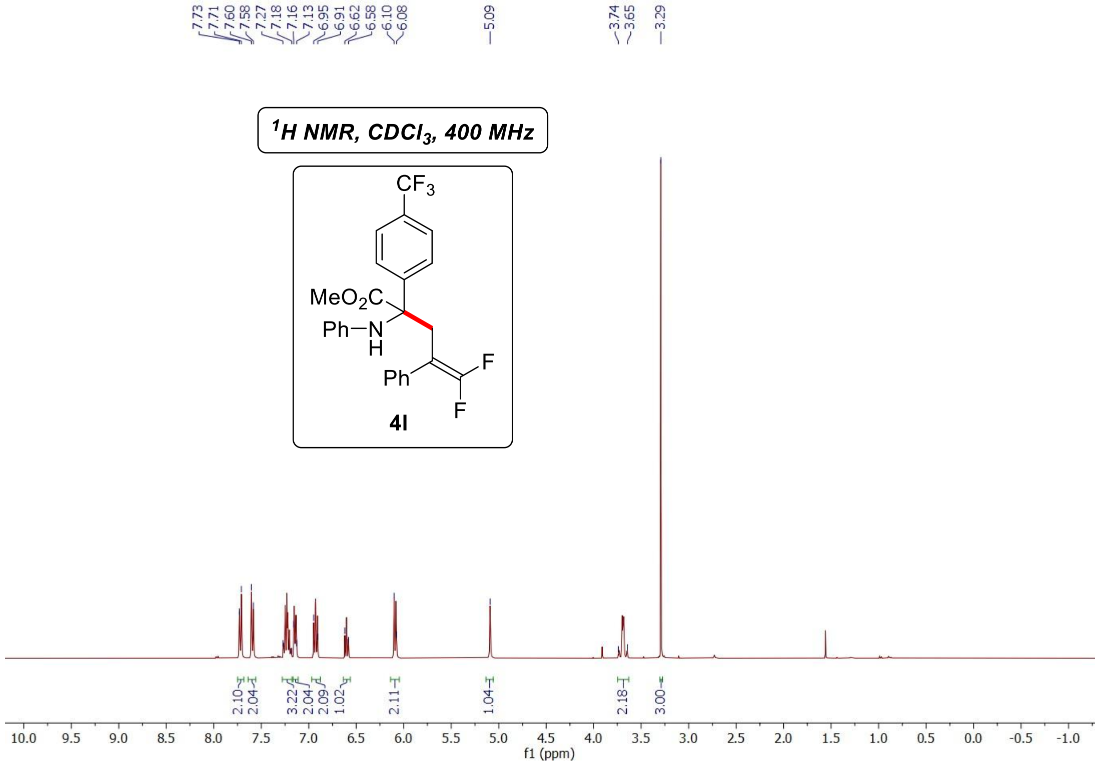

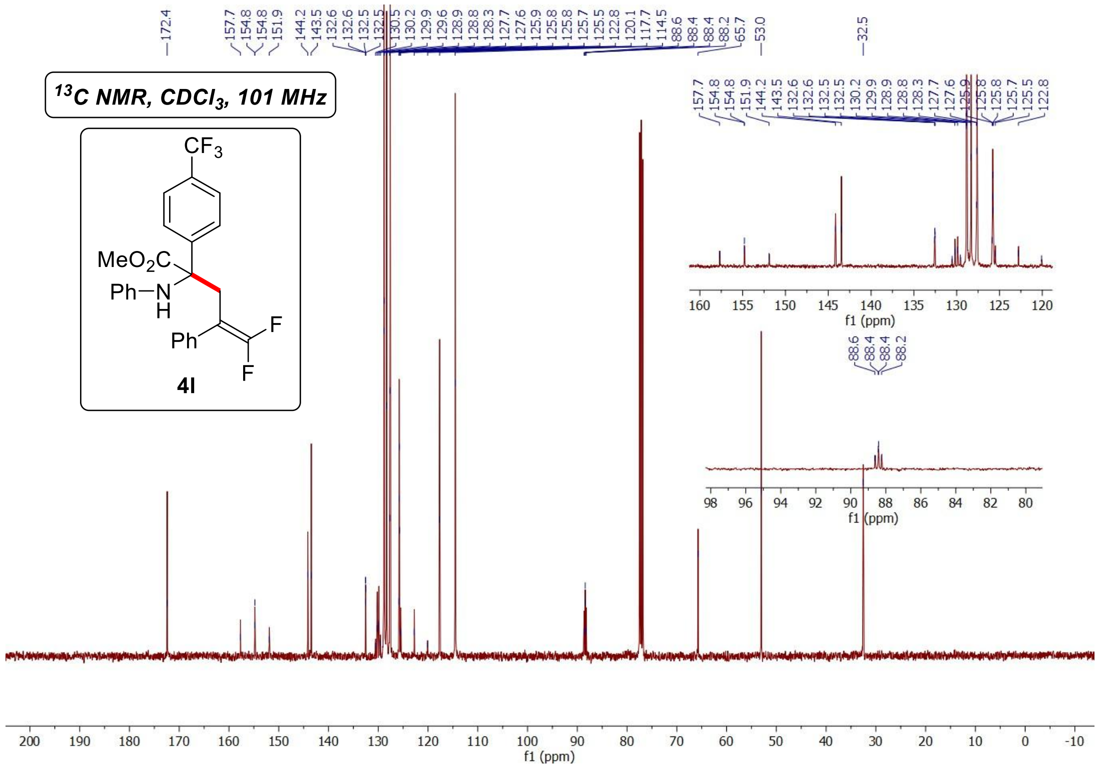

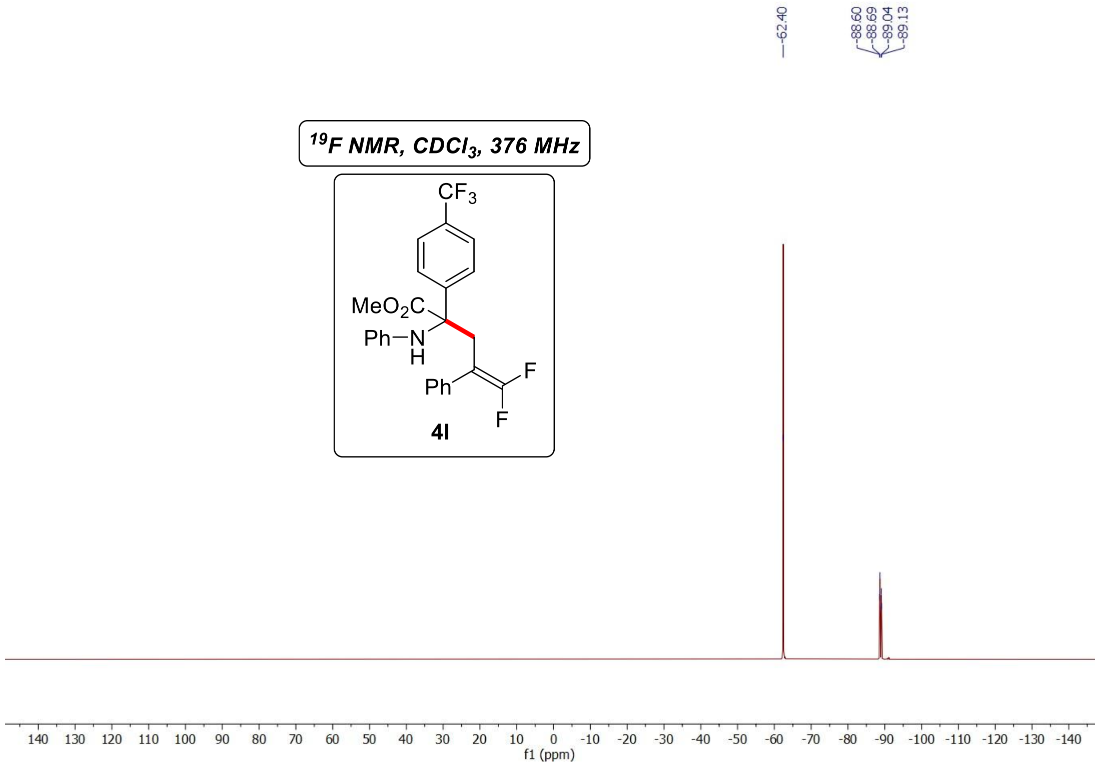
-62.40
-88.60
-88.69
-89.04
-89.13
19F NMR, CDCl3, 376 MHz
CF3
MeO2C
Ph–N
H
Ph
F
F
4l
140 130 120 110 100 90 80 70 60 50 40 30 20 10 0 -10 -20 -30 -40 -50 -60 -70 -80 -90 -100 -110 -120 -130 -140
f1 (ppm)

7.13. Methyl 2-((2-ethylphenyl)amino)-5,5-difluoro-2,4-diphenylpent-4-enoate (4m)

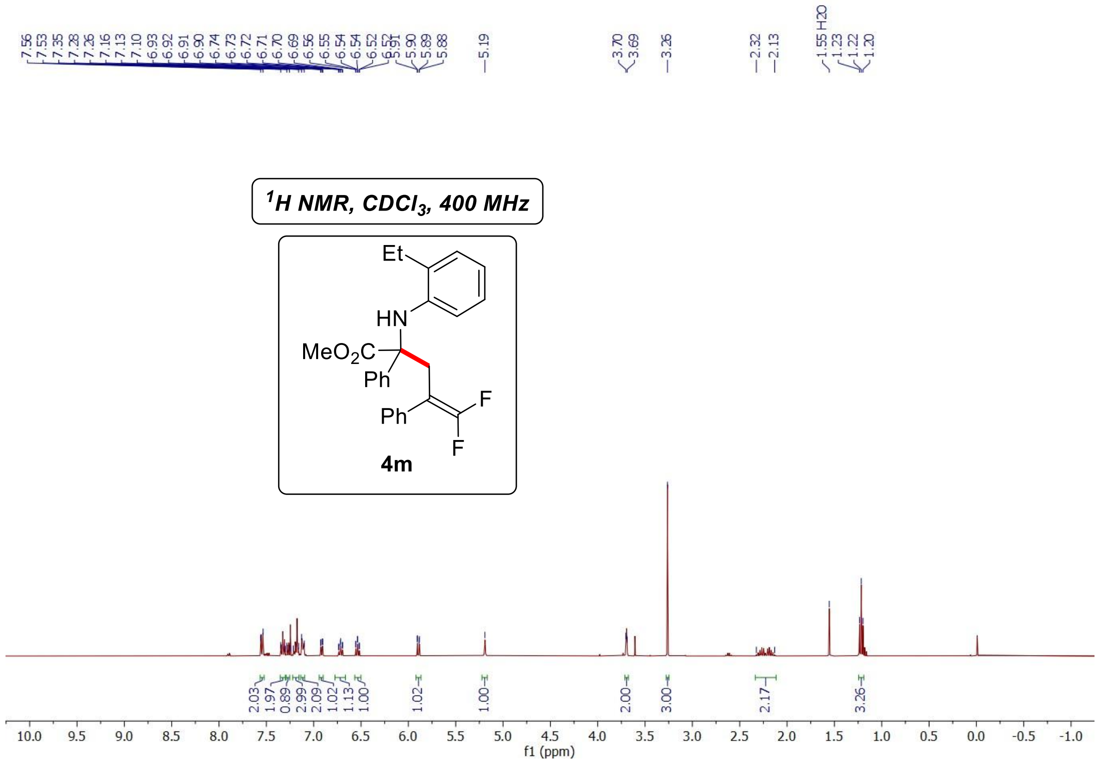

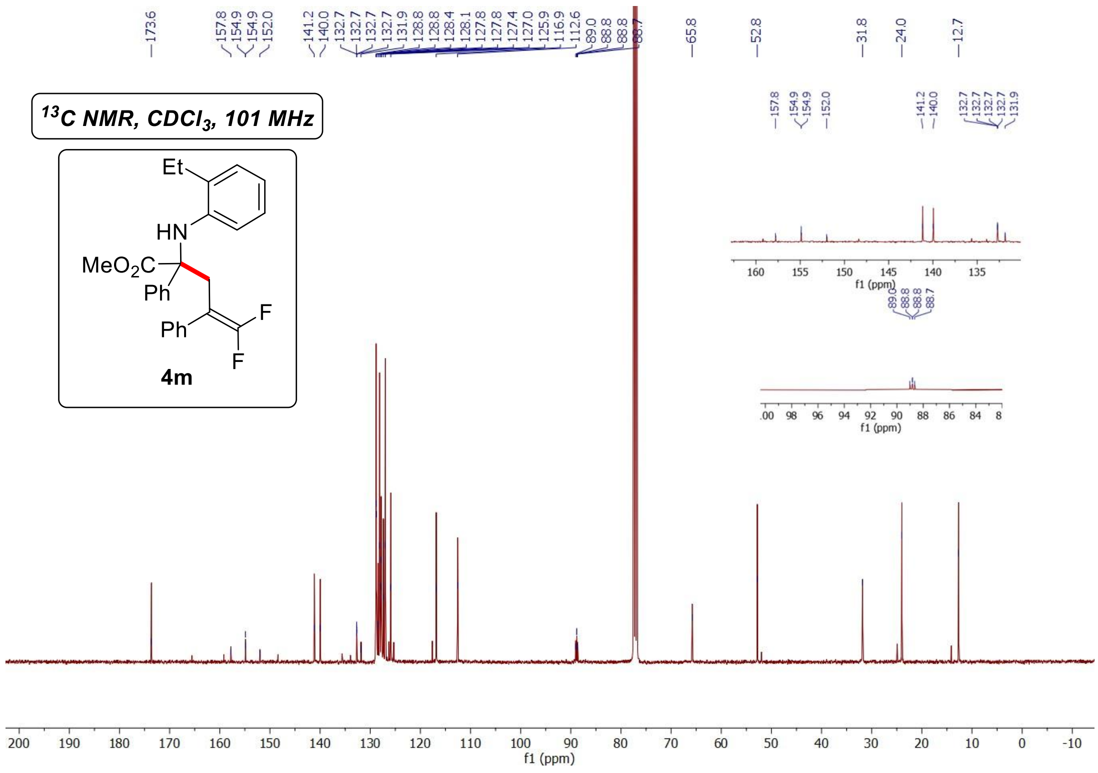

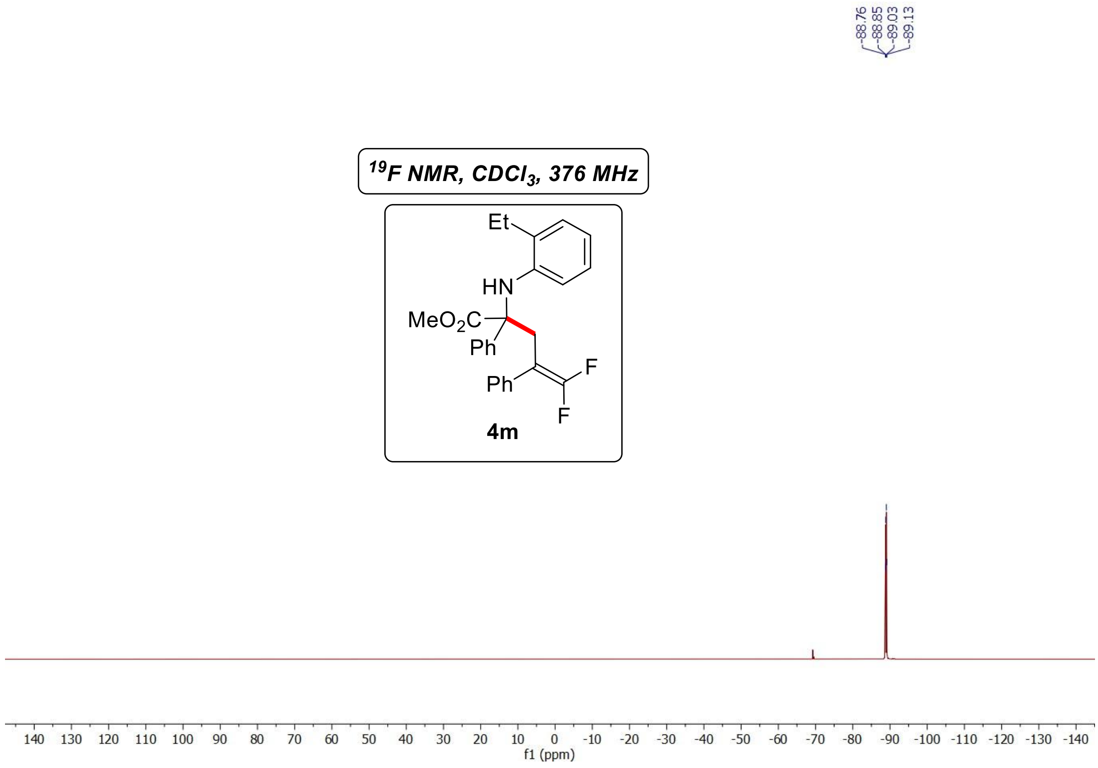

-88.76
-88.85
-89.03
-89.13
^{19}F NMR, CDCl$_3$, 376 MHz
Et
HN
MeO$_2$C
Ph
Ph
F
F
4m
140 130 120 110 100 90 80 70 60 50 40 30 20 10 0 -10 -20 -30 -40 -50 -60 -70 -80 -90 -100 -110 -120 -130 -140
f1 (ppm)

7.14. Methyl 2-((3-bromophenyl)amino)-5,5-difluoro-2,4-diphenylpent-4-enoate (4n)

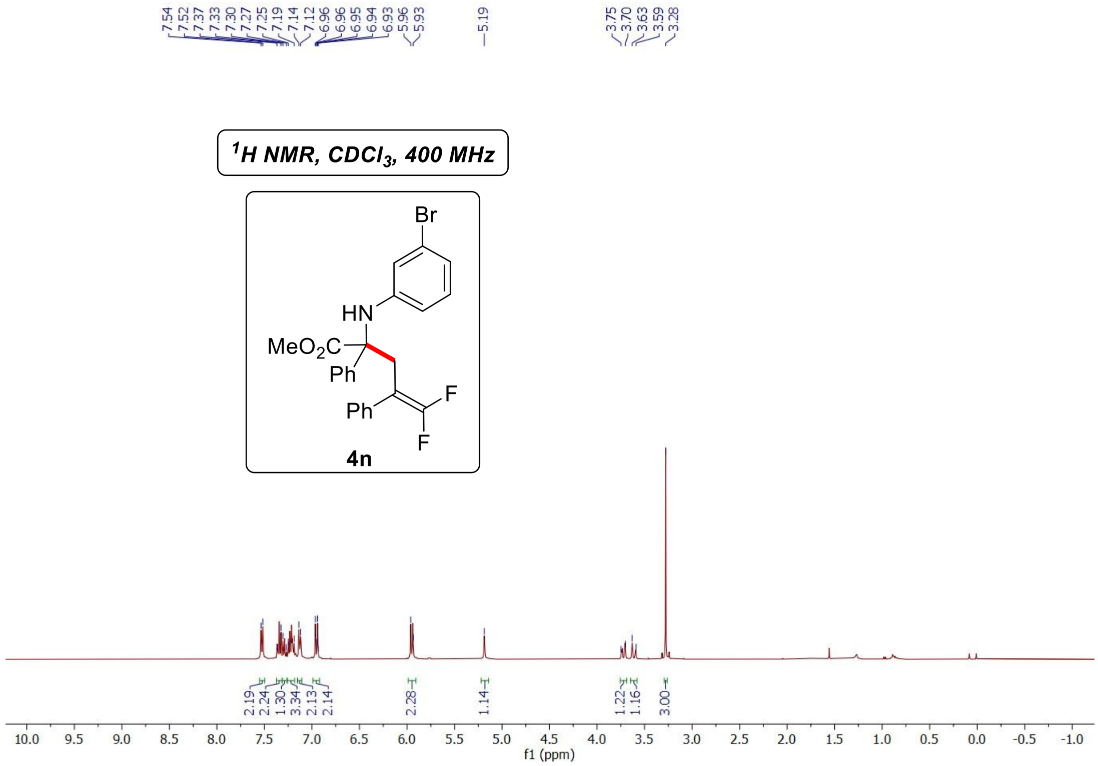

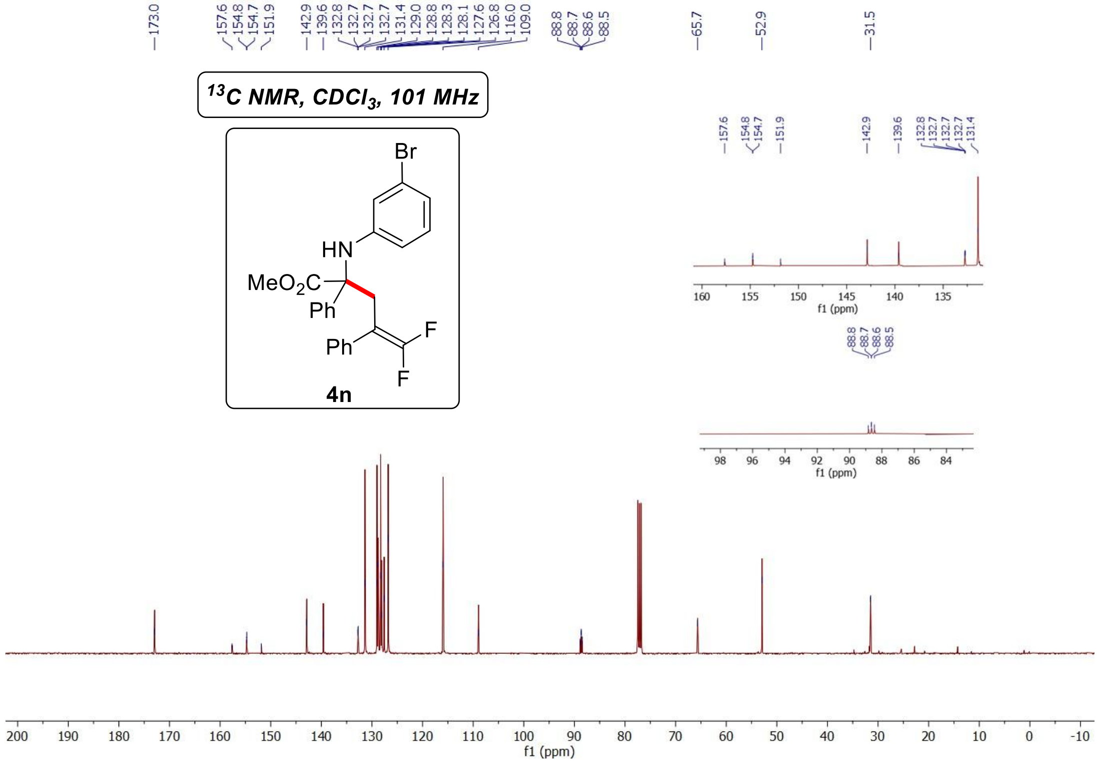

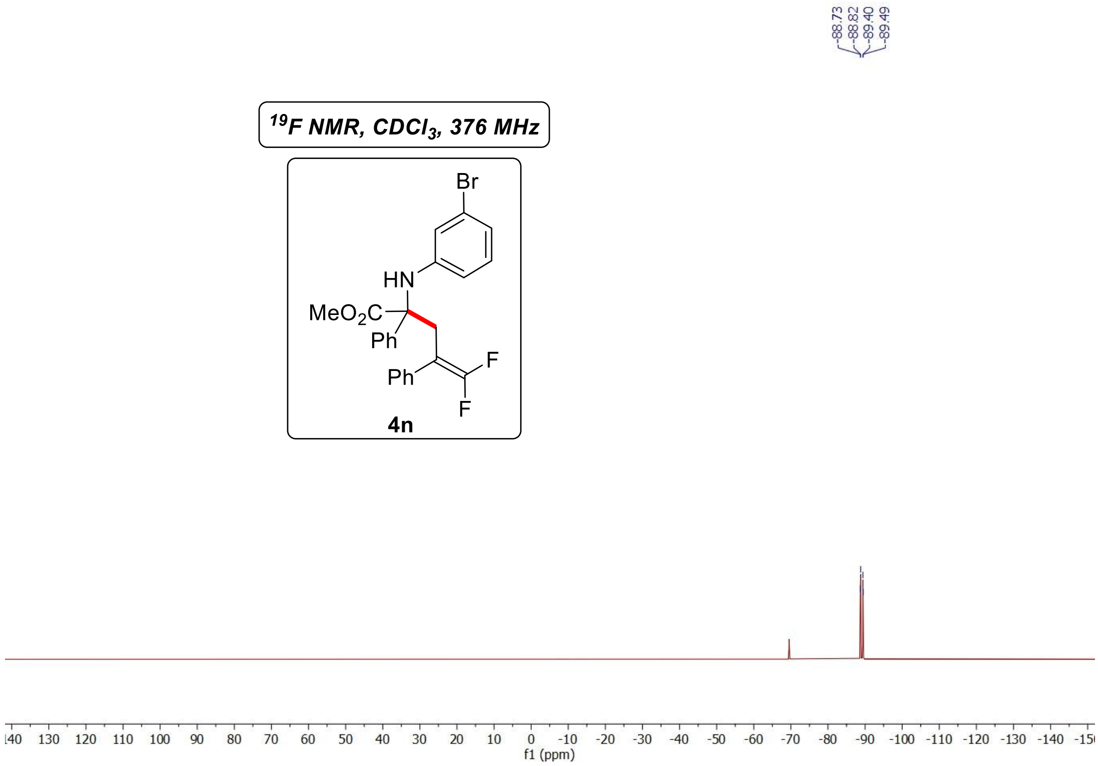
-88.73
-88.82
-89.40
-89.49
19F NMR, CDCl3, 376 MHz
Br
HN
MeO2C
Ph
Ph
F
F
4n
140 130 120 110 100 90 80 70 60 50 40 30 20 10 0 -10 -20 -30 -40 -50 -60 -70 -80 -90 -100 -110 -120 -130 -140 -150
f1 (ppm)

7.15. Methyl 5,5-difluoro-2-((3-fluorophenyl)amino)-2,4-diphenylpent-4-enoate (4o)

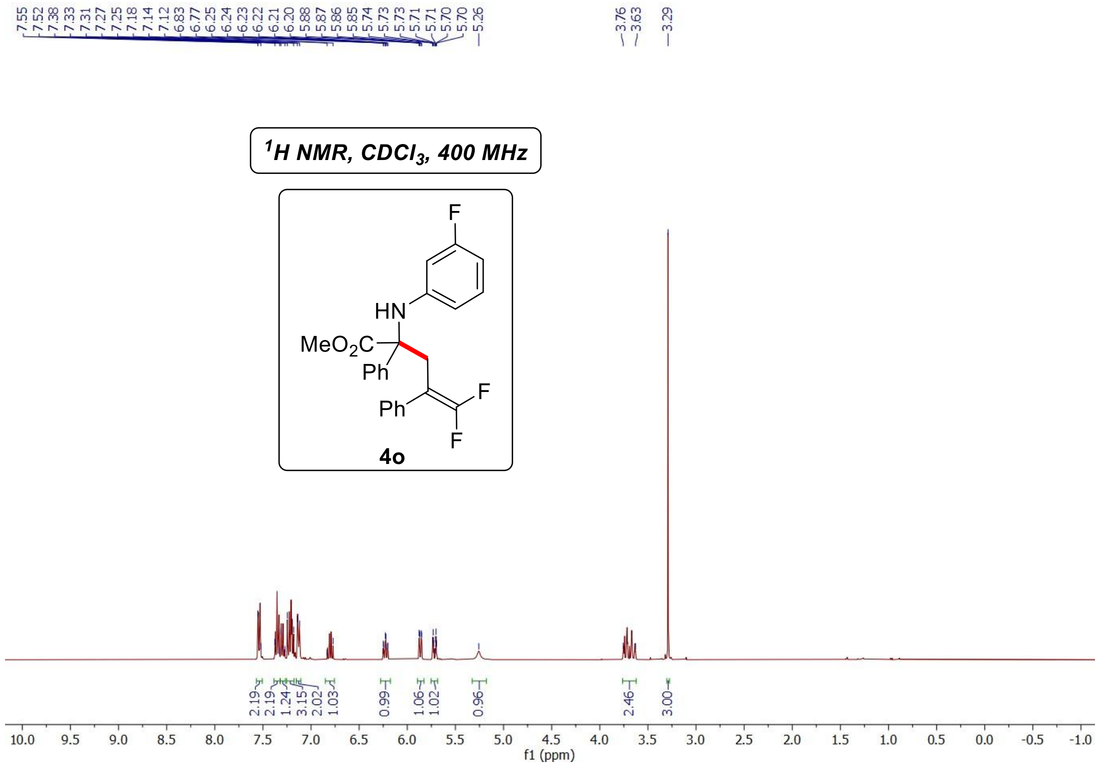

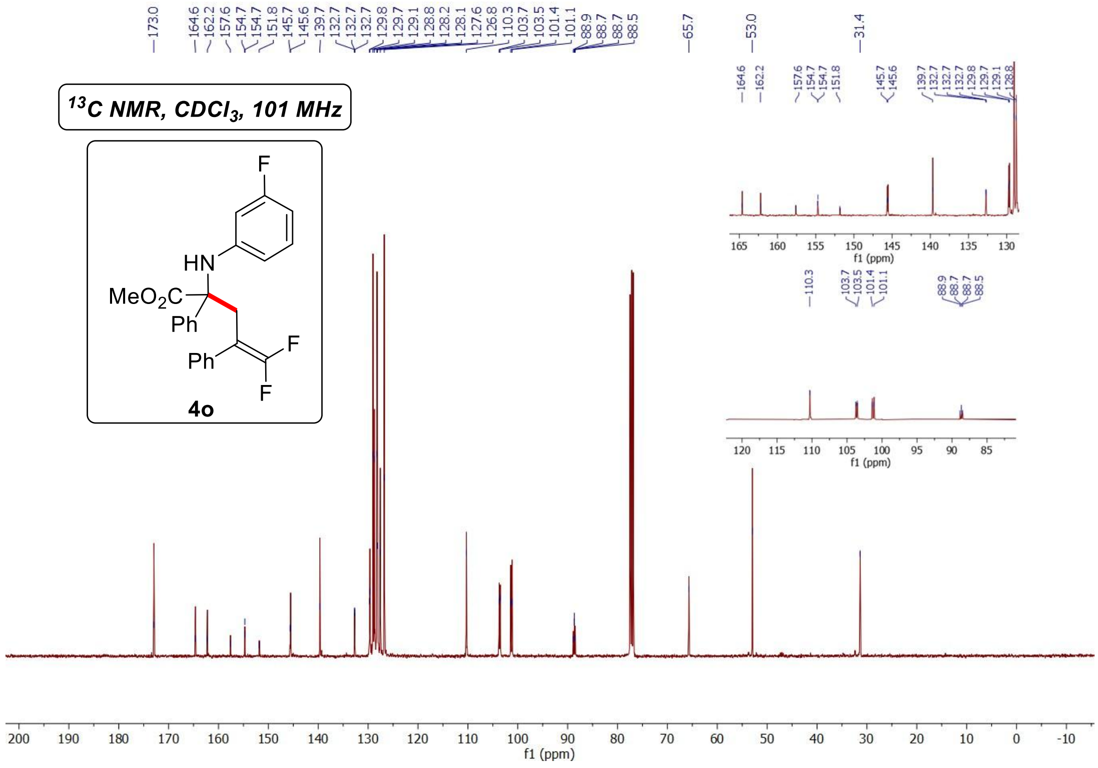

^{19}F NMR, $CDCl_3$, 376 MHz

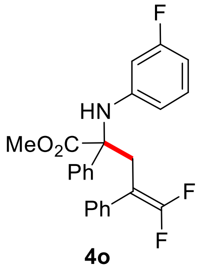

4o

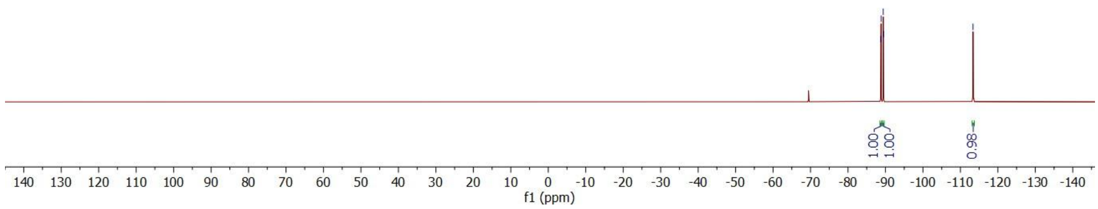

7.16. Methyl 5,5-difluoro-2-((4-methoxyphenyl)amino)-2,4-diphenylpent-4-enoate (4p)

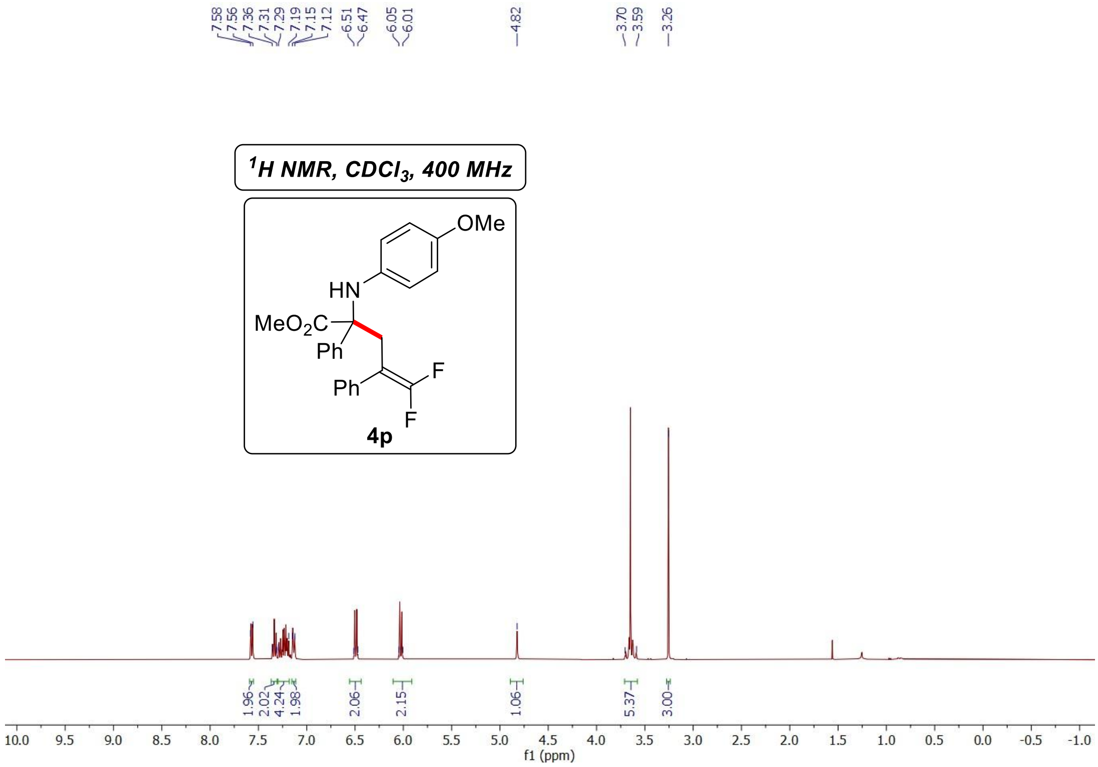

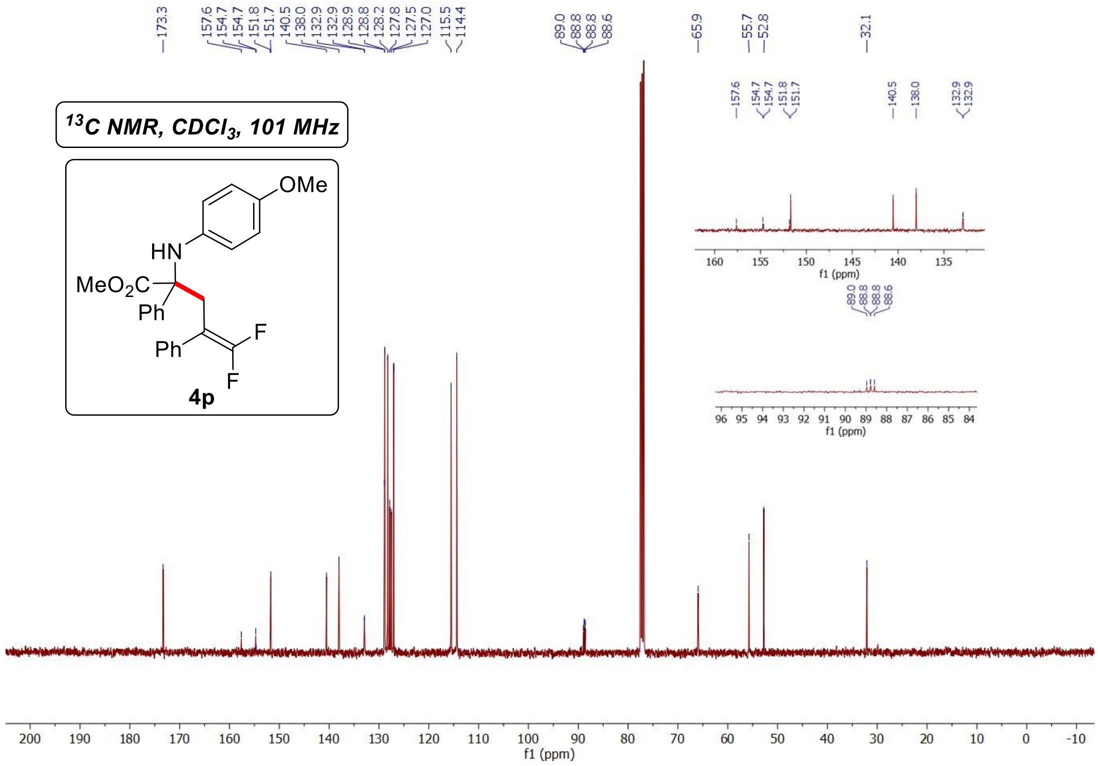

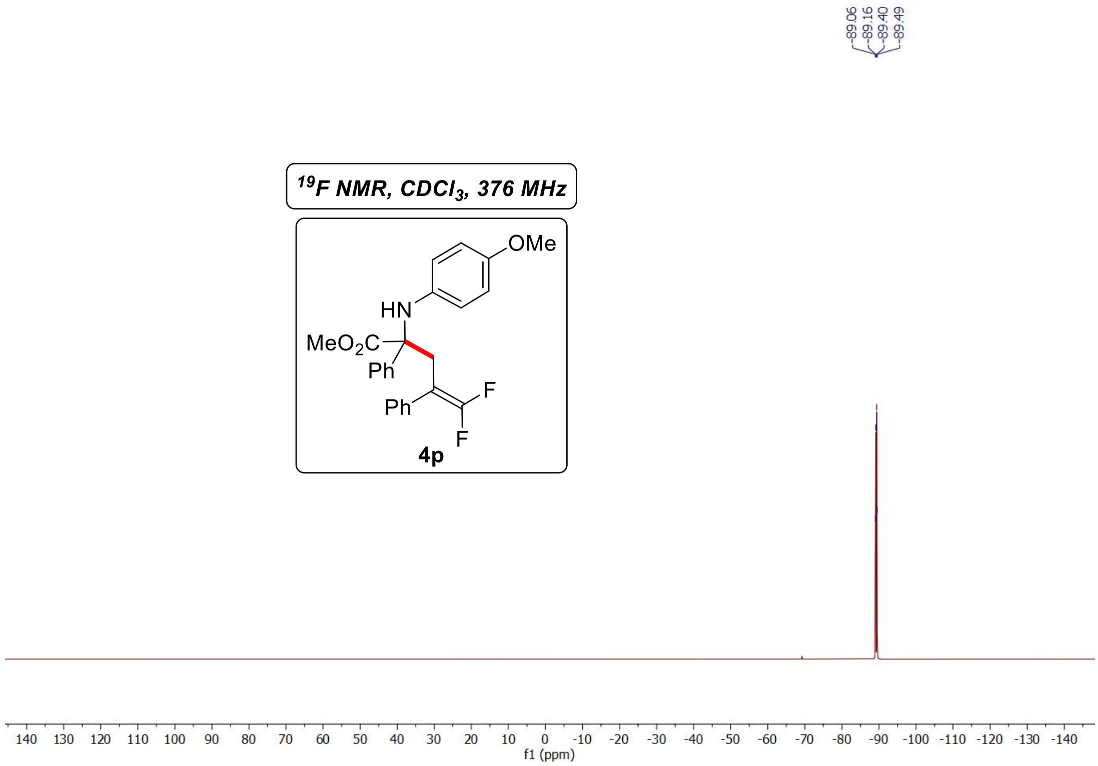
-89.06
-89.16
-89.40
-89.49
19F NMR, CDCl3, 376 MHz
OMe
HN
MeO2C
Ph
Ph
F
F
4p
140 130 120 110 100 90 80 70 60 50 40 30 20 10 0 -10 -20 -30 -40 -50 -60 -70 -80 -90 -100 -110 -120 -130 -140
f1 (ppm)

7.17. Methyl 5,5-difluoro-2,4-diphenyl-2-((3-(trifluoromethoxy)phenyl)amino)pent-4-enoate (4q)

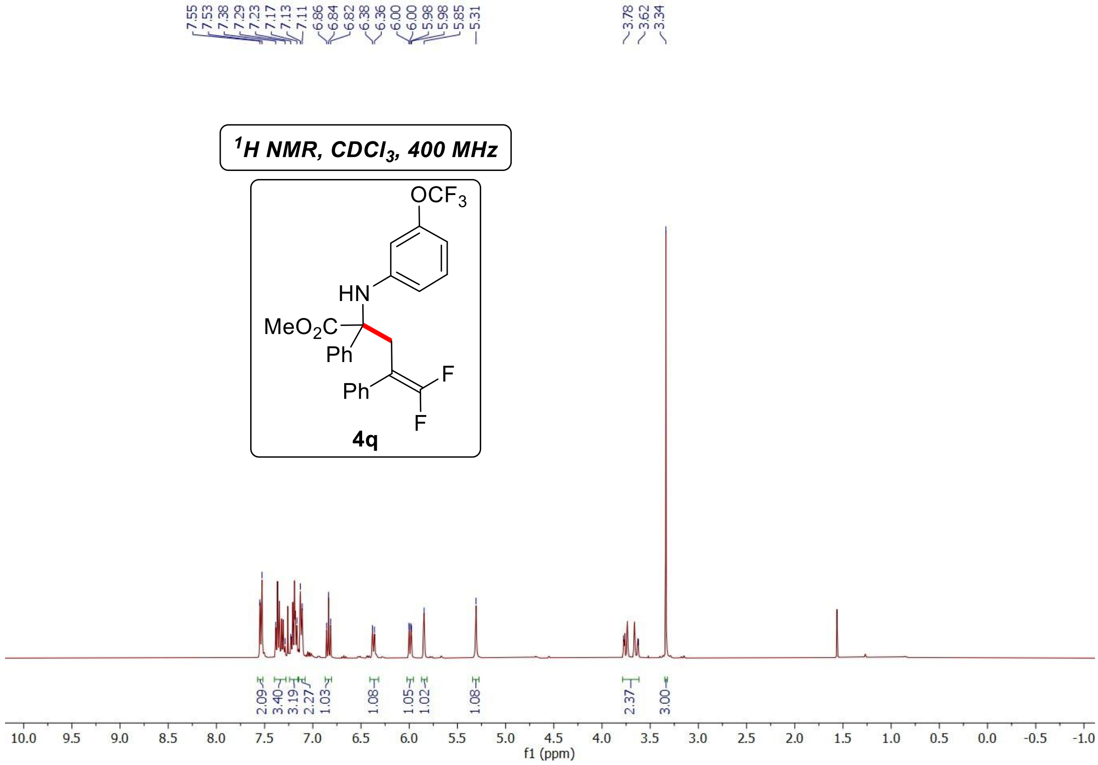

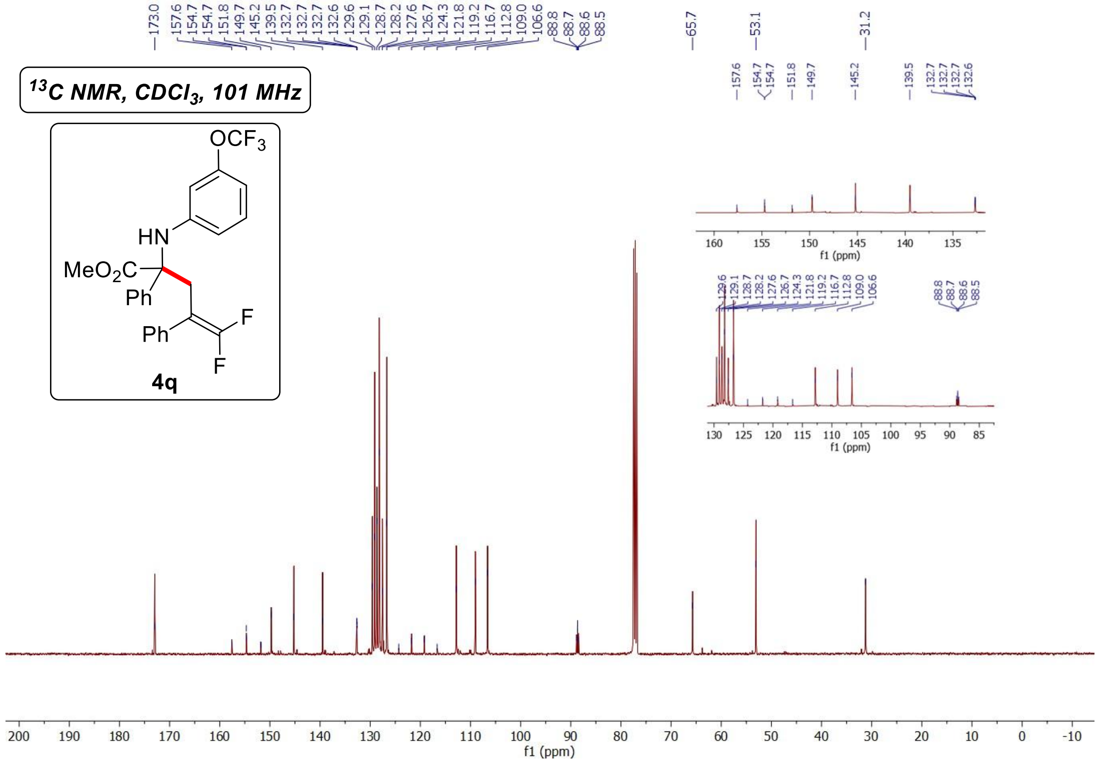

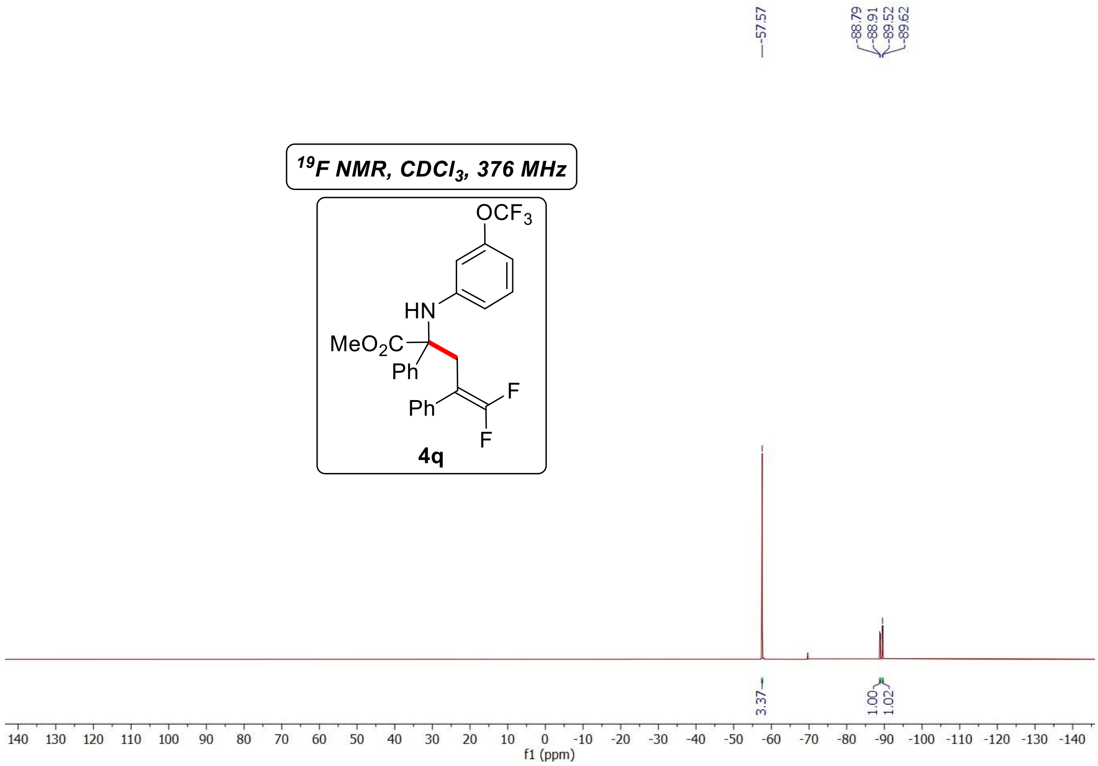

-57.57
-88.79
-88.91
-89.52
-89.62
19F NMR, CDCl3, 376 MHz
OCF3
HN
MeO2C
Ph
Ph
F
F
4q
3.37
1.00
1.02
140 130 120 110 100 90 80 70 60 50 40 30 20 10 0 -10 -20 -30 -40 -50 -60 -70 -80 -90 -100 -110 -120 -130 -140
f1 (ppm)

7.18. Methyl 2-((3,5-dimethoxyphenyl)amino)-5,5-difluoro-2,4-diphenylpent-4-enoate (4r)

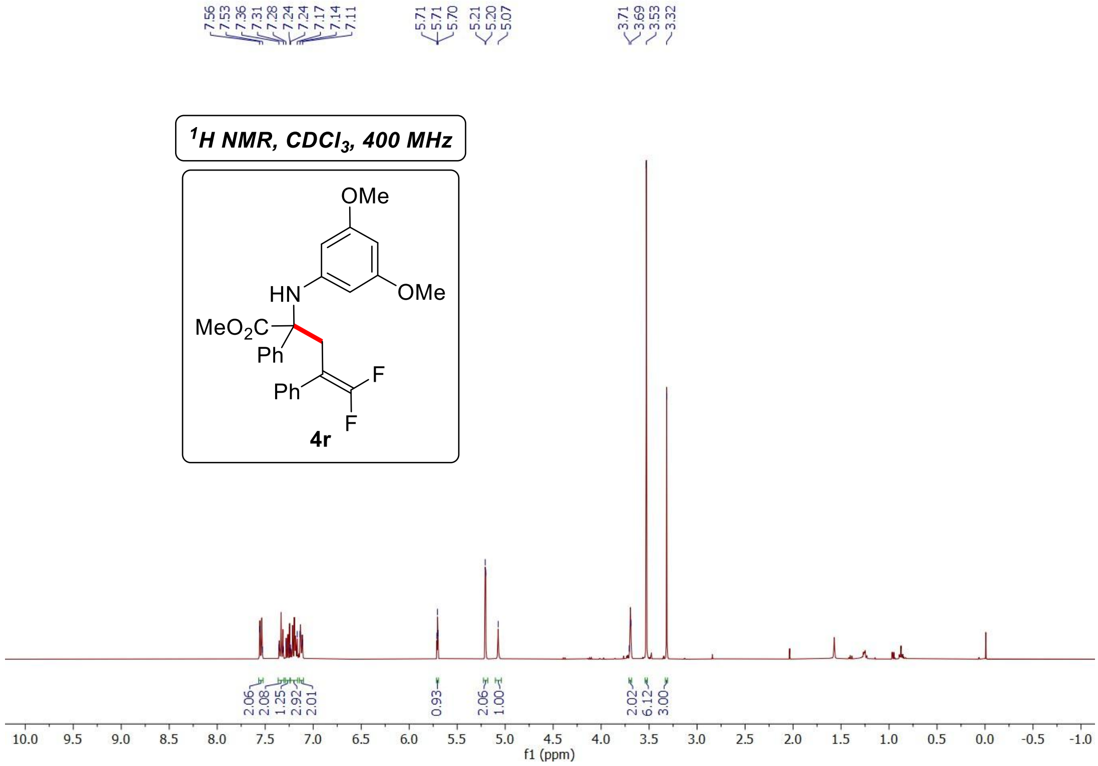

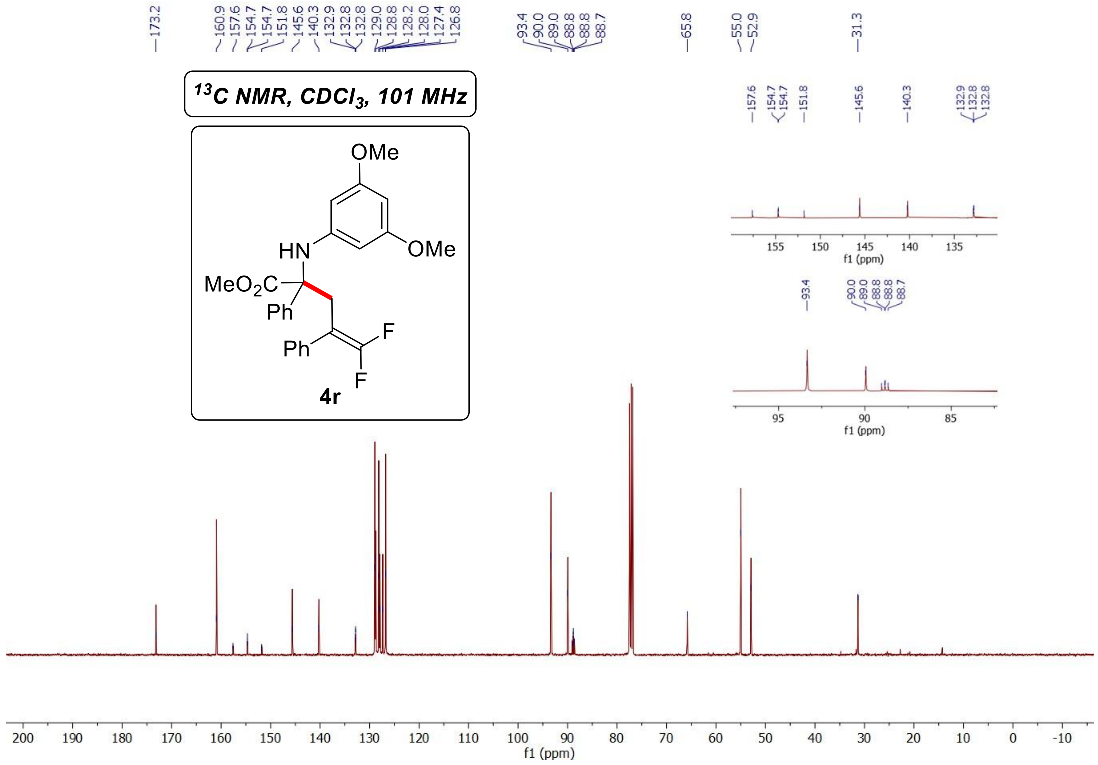

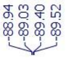

^{19}F NMR, $CDCl_3$, 376 MHz

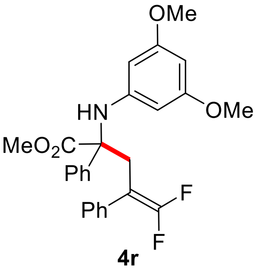

4r

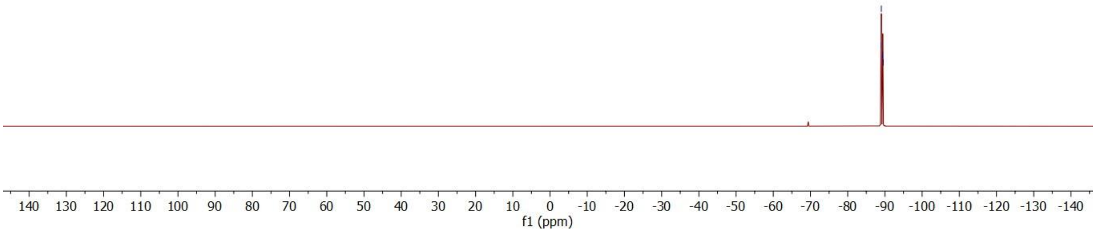

7.19. Methyl 5,5-difluoro-2,4-diphenyl-2-(p-tolylamino)pent-4-enoate (4s)

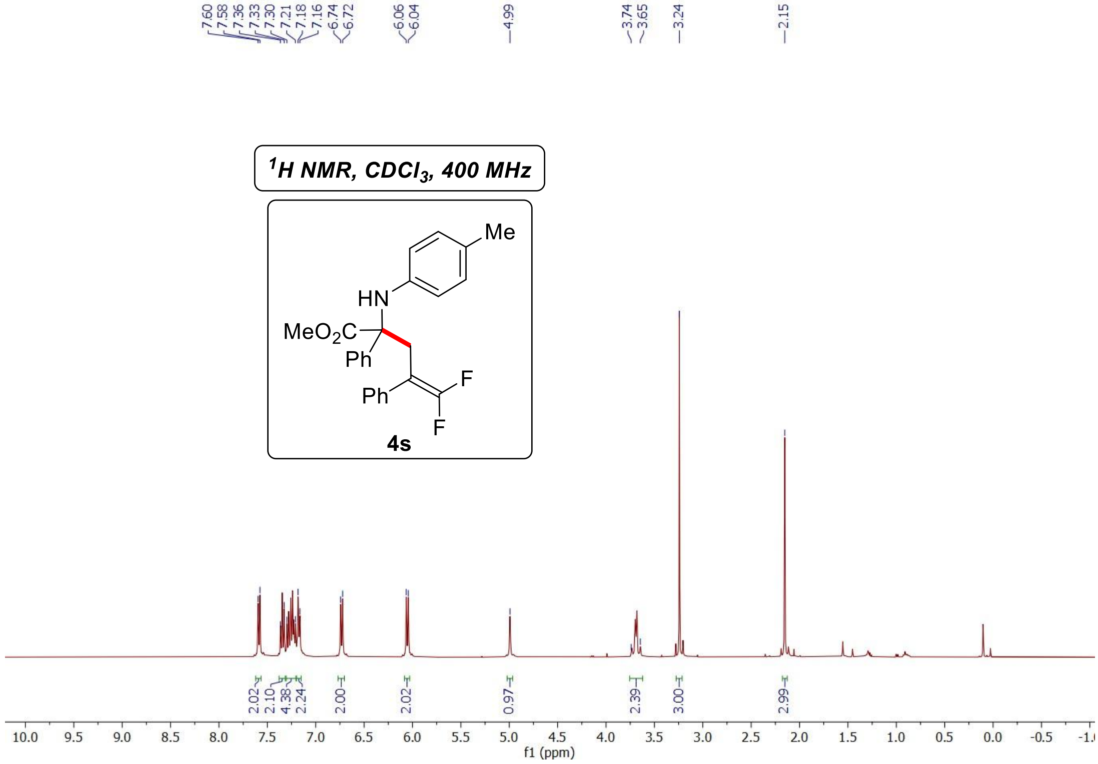

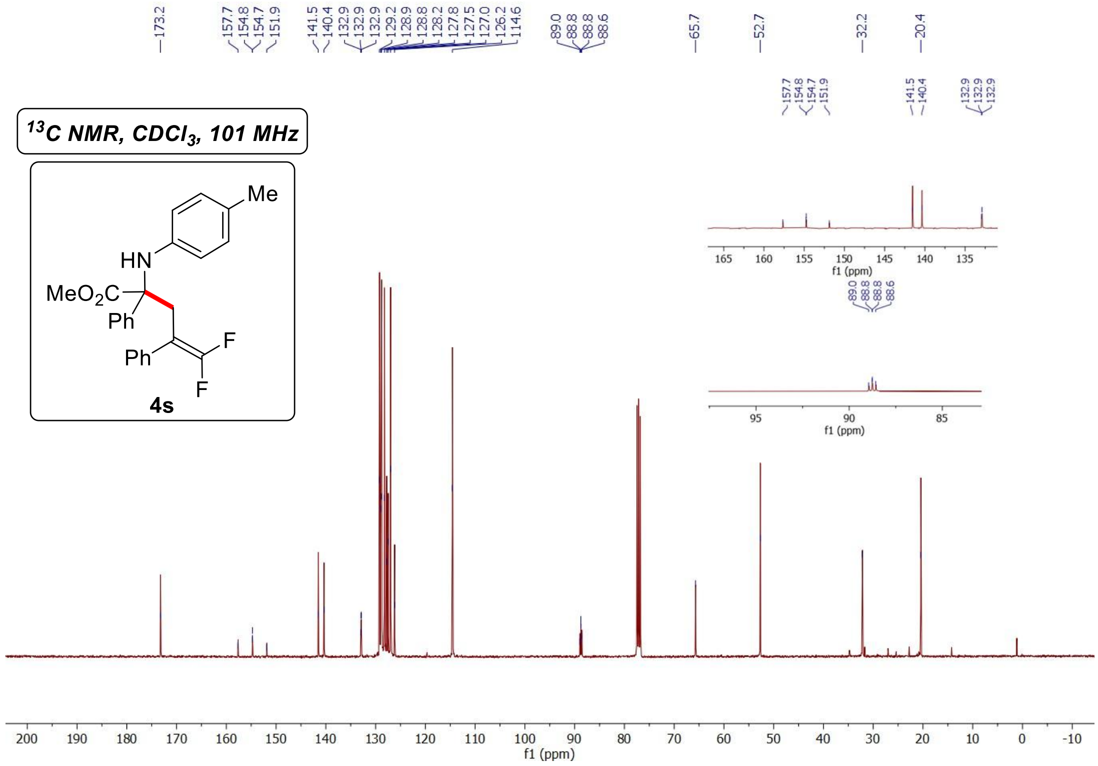

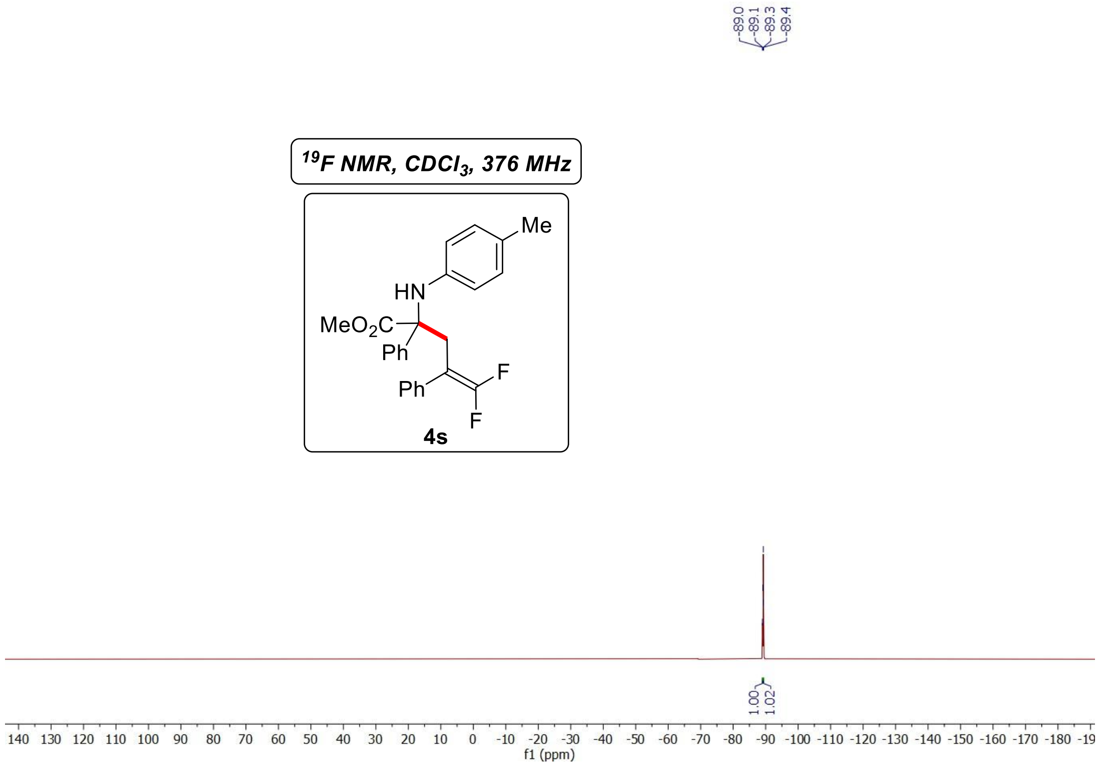
-89.0
-89.1
-89.3
-89.4
19F NMR, CDCl3, 376 MHz
Me
HN
MeO2C
Ph
Ph
F
F
4s
1.00
1.02
140 130 120 110 100 90 80 70 60 50 40 30 20 10 0 -10 -20 -30 -40 -50 -60 -70 -80 -90 -100 -110 -120 -130 -140 -150 -160 -170 -180 -19
f1 (ppm)

7.20. Methyl 2-((4-chlorophenyl)amino)-5,5-difluoro-2,4-diphenylpent-4-enoate (4t)

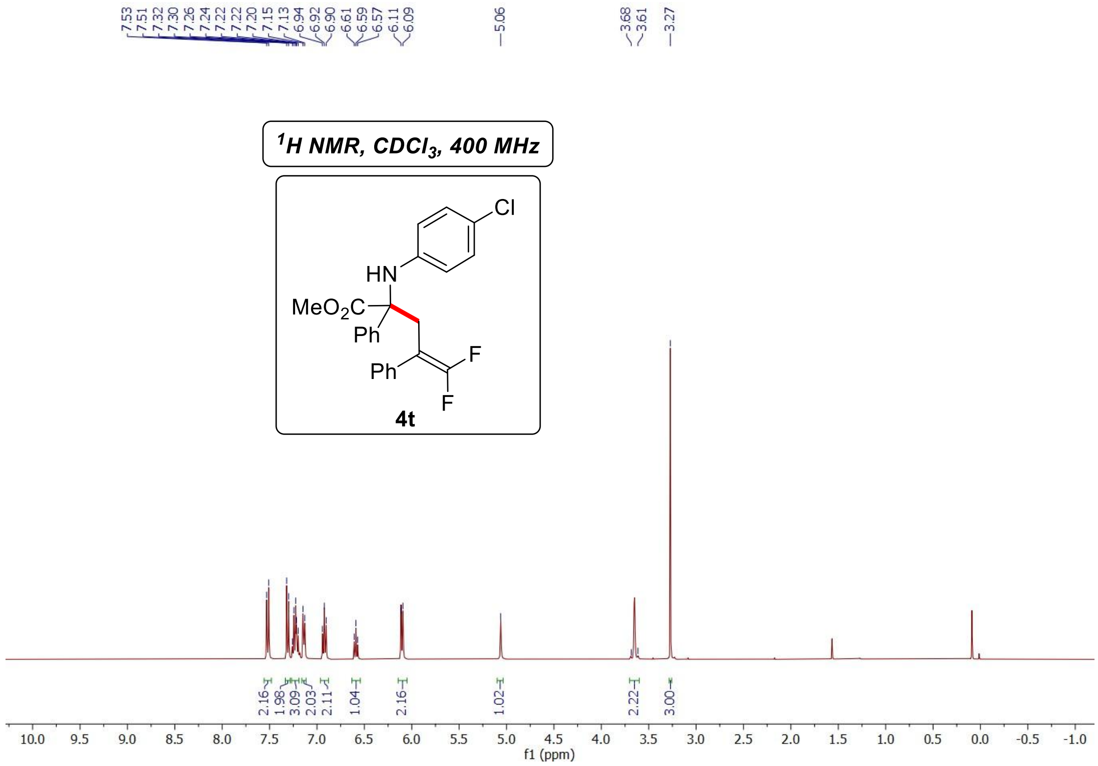

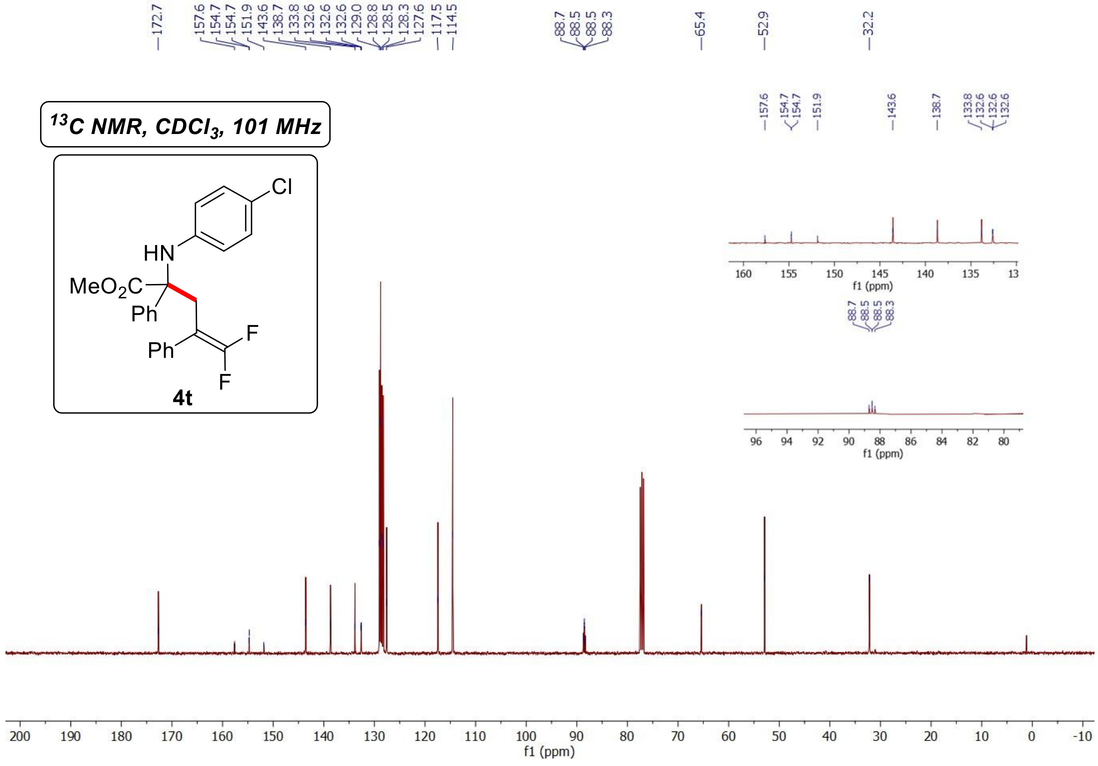

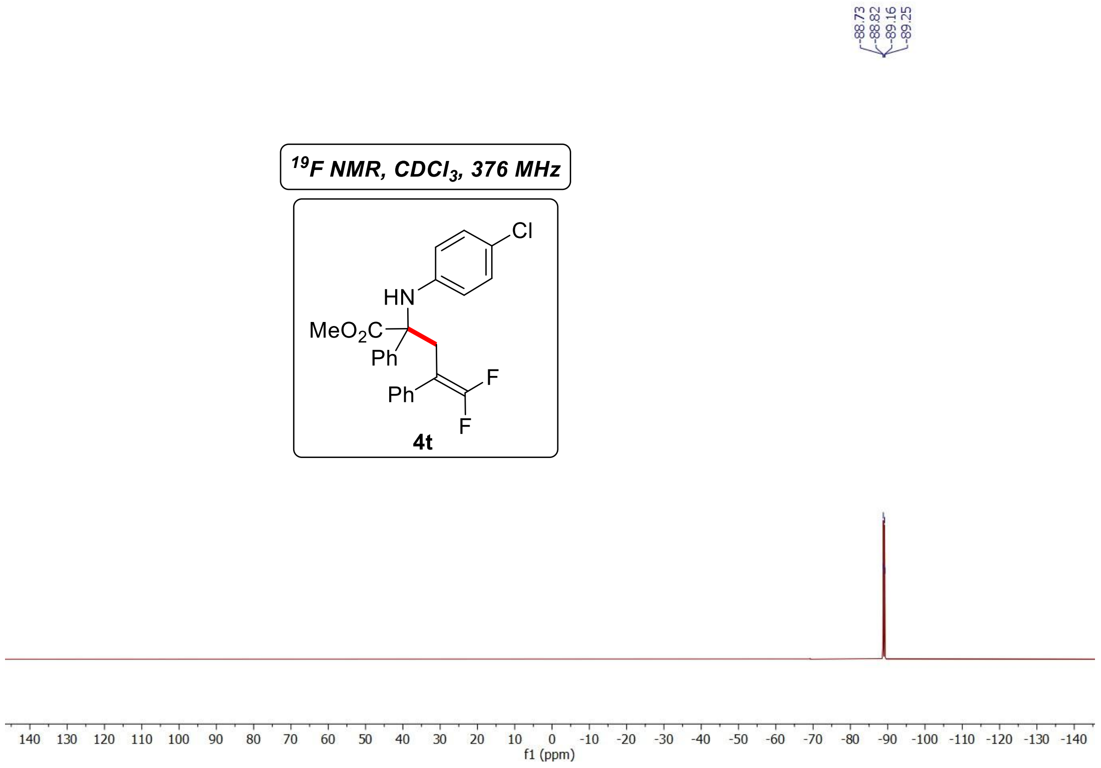
-88.73
-88.82
-89.16
-89.25
^{19}F NMR, $CDCl_3$, 376 MHz
Cl
HN
MeO_2C
Ph
Ph
F
F
4t
140 130 120 110 100 90 80 70 60 50 40 30 20 10 0 -10 -20 -30 -40 -50 -60 -70 -80 -90 -100 -110 -120 -130 -140
f1 (ppm)

7.21. Methyl 2-((4-bromophenyl)amino)-5,5-difluoro-2,4-diphenylpent-4-enoate (4u)

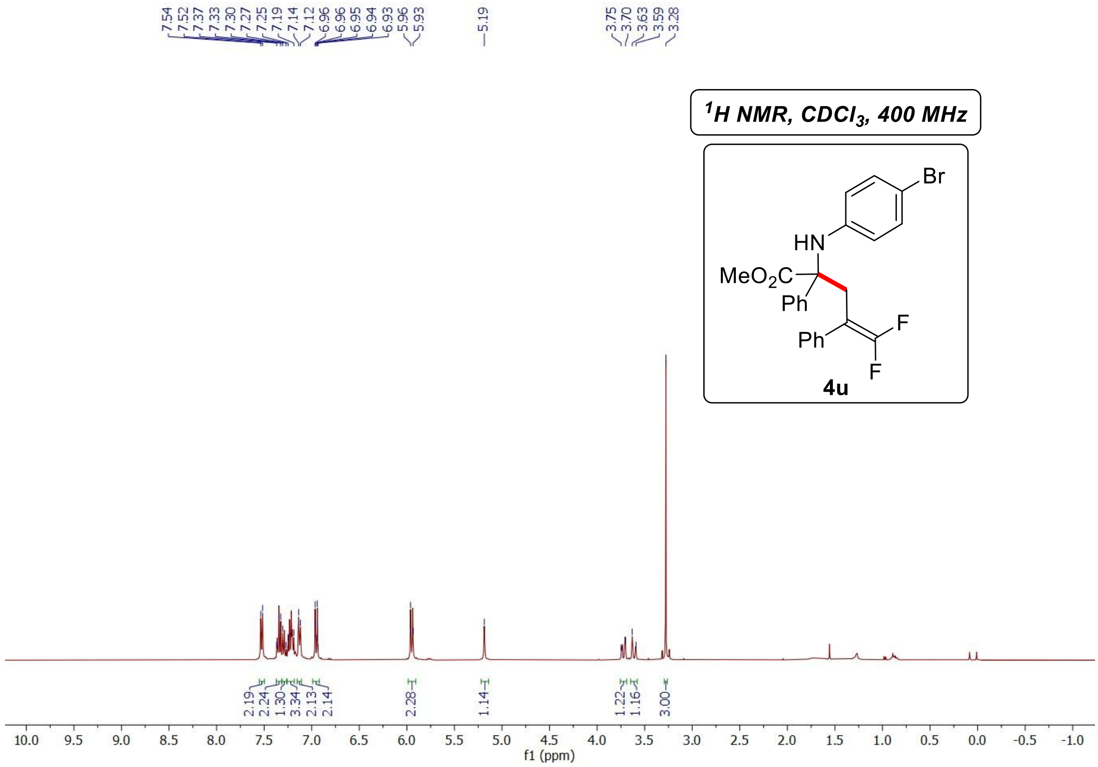

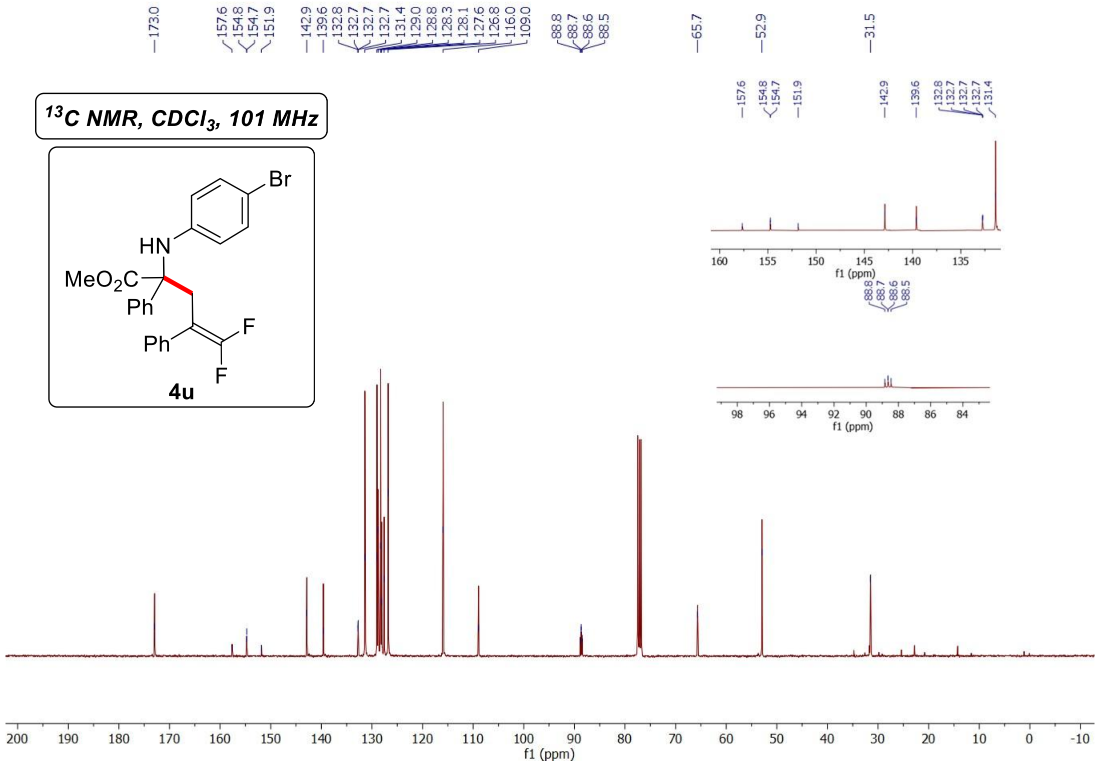

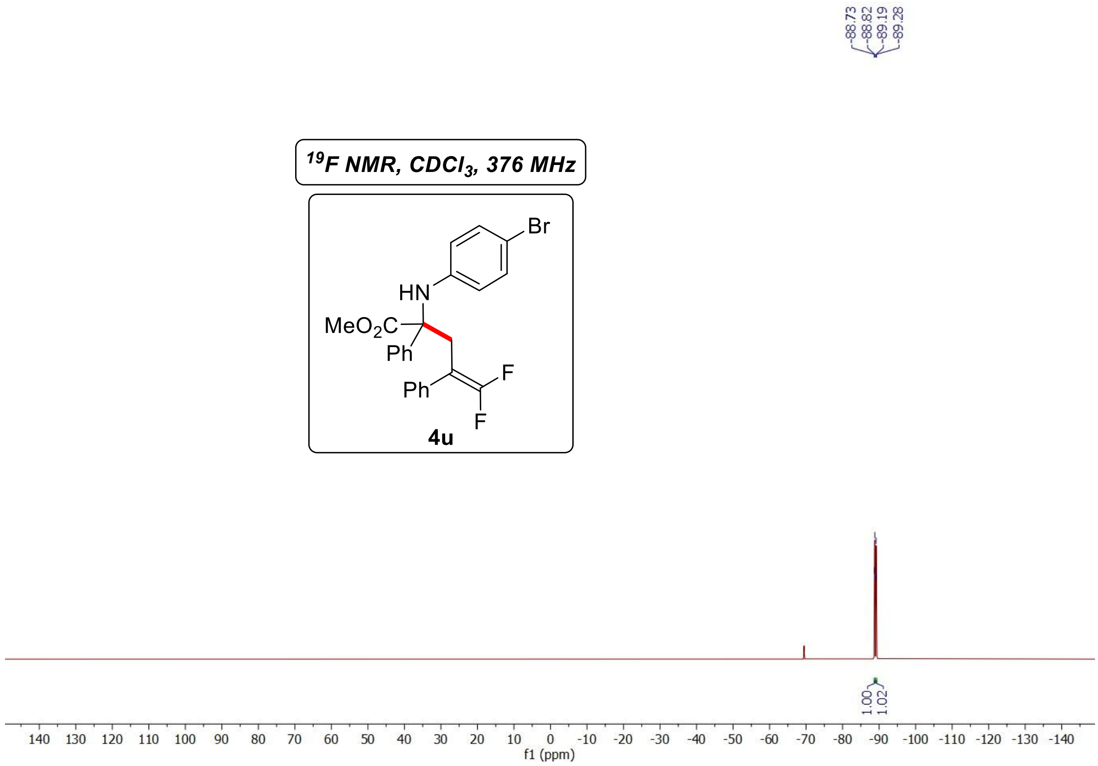

-88.73
-88.82
-89.19
-89.28
19F NMR, CDCl3, 376 MHz
Br
HN
MeO2C
Ph
Ph
F
F
4u
1.00
1.02
140 130 120 110 100 90 80 70 60 50 40 30 20 10 0 -10 -20 -30 -40 -50 -60 -70 -80 -90 -100 -110 -120 -130 -140
f1 (ppm)

7.22. Methyl 5,5-difluoro-2-((4-isopropylphenyl)amino)-2,4-diphenylpent-4-enoate (4v)

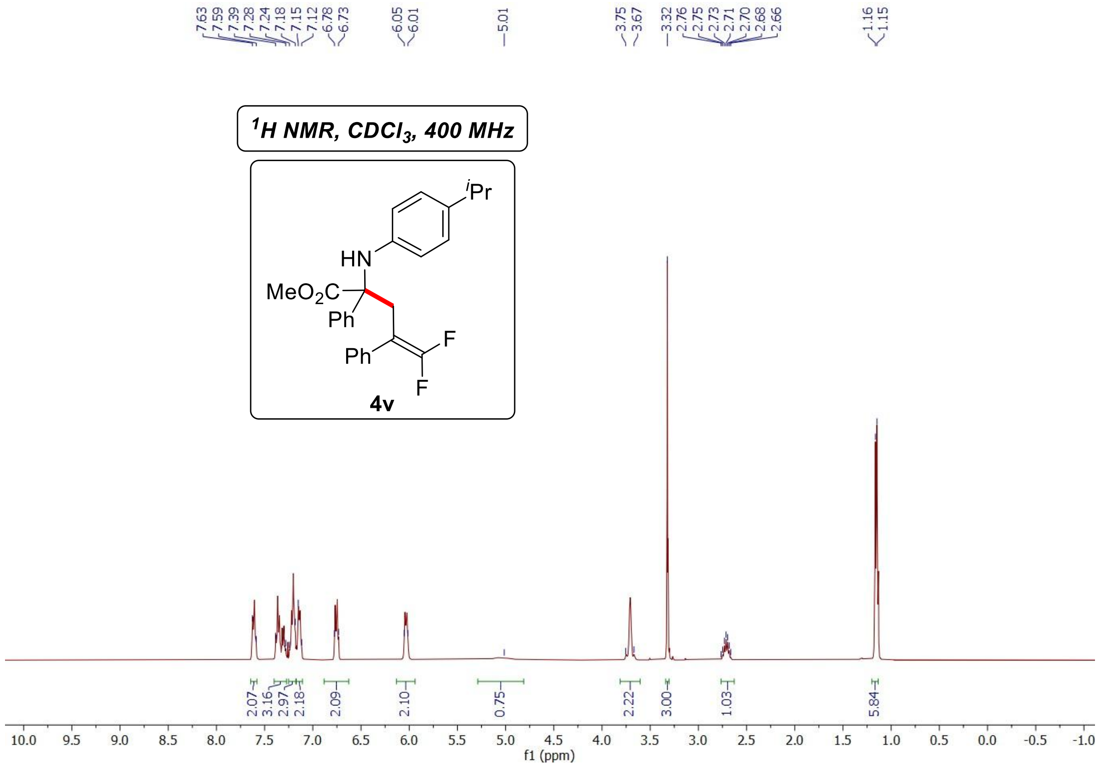

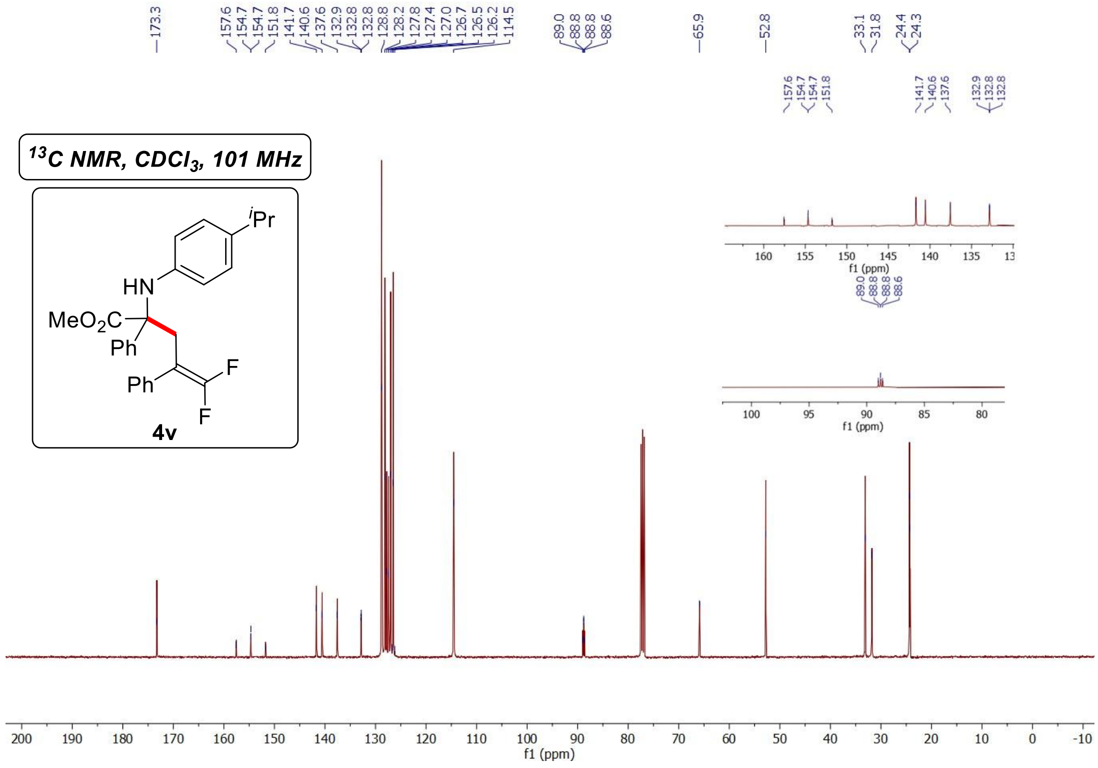

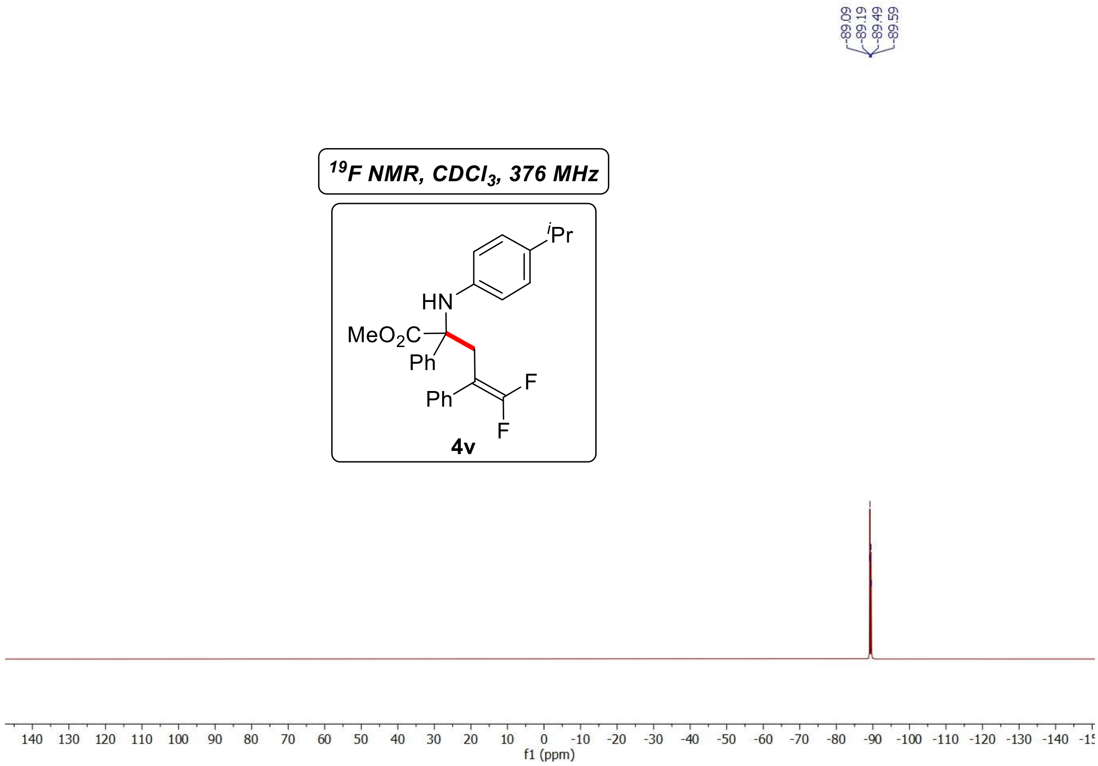
-89.09
-89.19
-89.49
-89.59
19F NMR, CDCl3, 376 MHz
iPr
HN
MeO2C
Ph
Ph
F
F
4v
140 130 120 110 100 90 80 70 60 50 40 30 20 10 0 -10 -20 -30 -40 -50 -60 -70 -80 -90 -100 -110 -120 -130 -140
f1 (ppm)

7.23. Methyl 4-(4-(tert-butyl)phenyl)-5,5-difluoro-2-phenyl-2-(phenylamino)pent-4-enoate (4w)

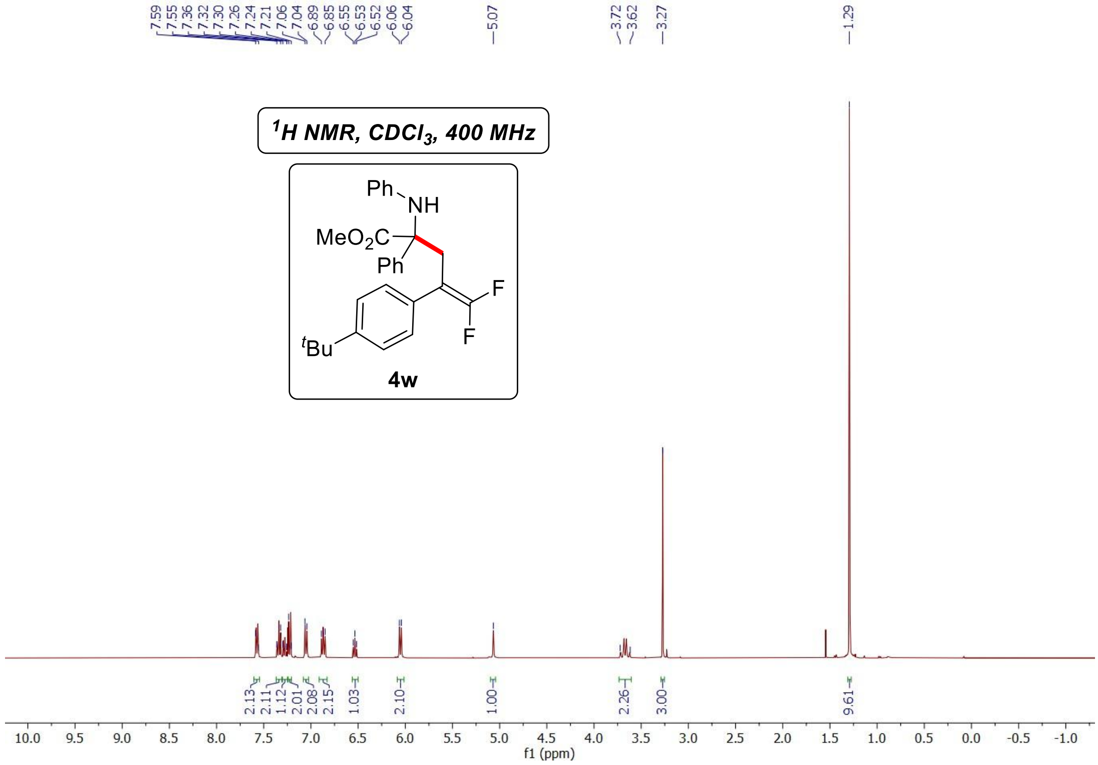

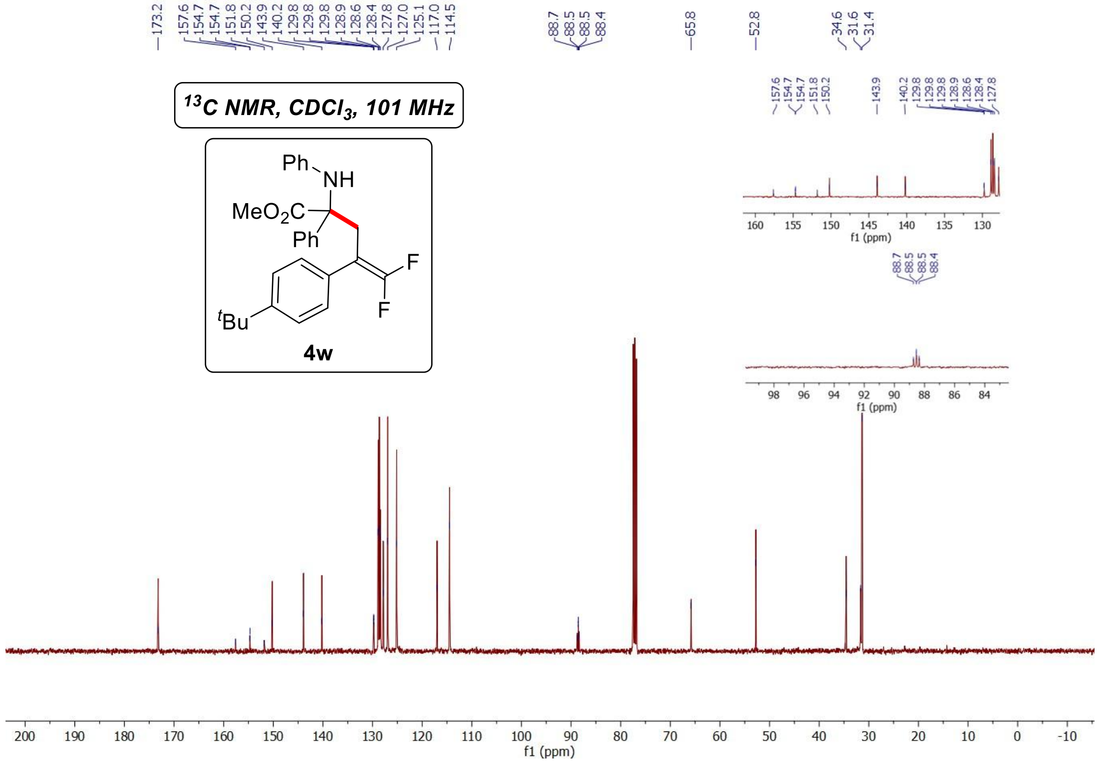

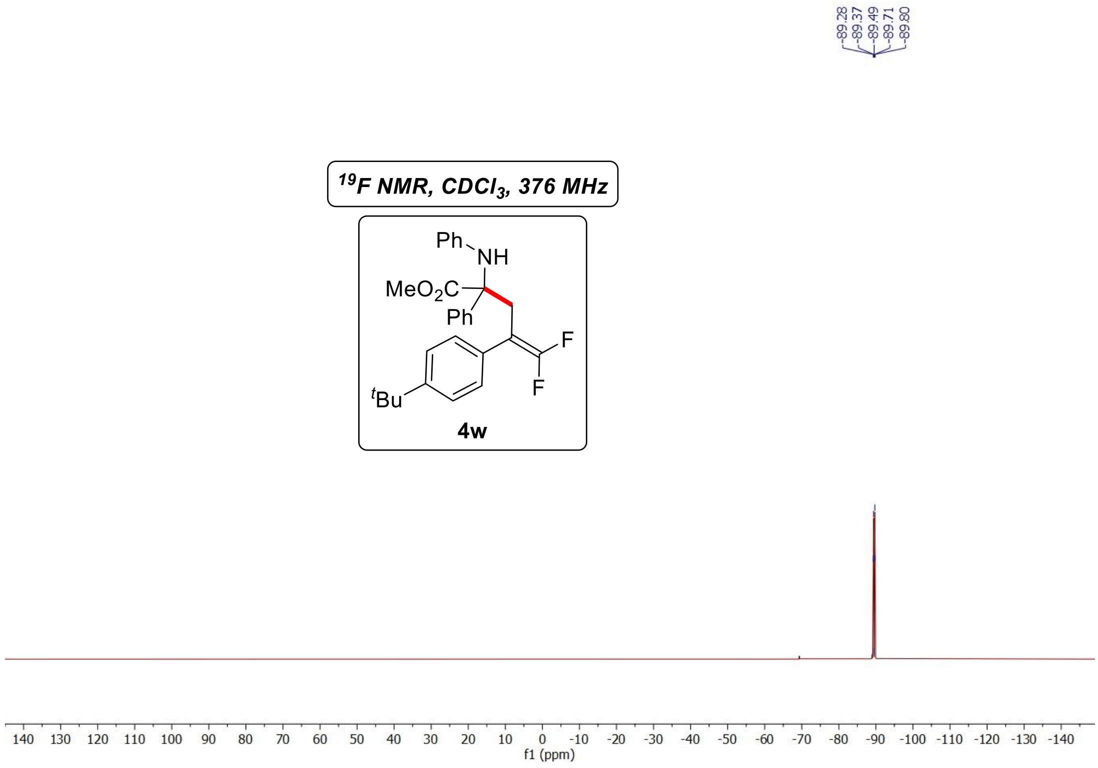
-89.28
-89.37
-89.49
-89.71
-89.80
19F NMR, CDCl3, 376 MHz
Ph
NH
MeO2C
Ph
F
F
tBu
4w
140 130 120 110 100 90 80 70 60 50 40 30 20 10 0 -10 -20 -30 -40 -50 -60 -70 -80 -90 -100 -110 -120 -130 -140
f1 (ppm)

7.24. Methyl 5,5-difluoro-4-(4-methoxyphenyl)-2-phenyl-2-(phenylamino)pent-4-enoate (4x)

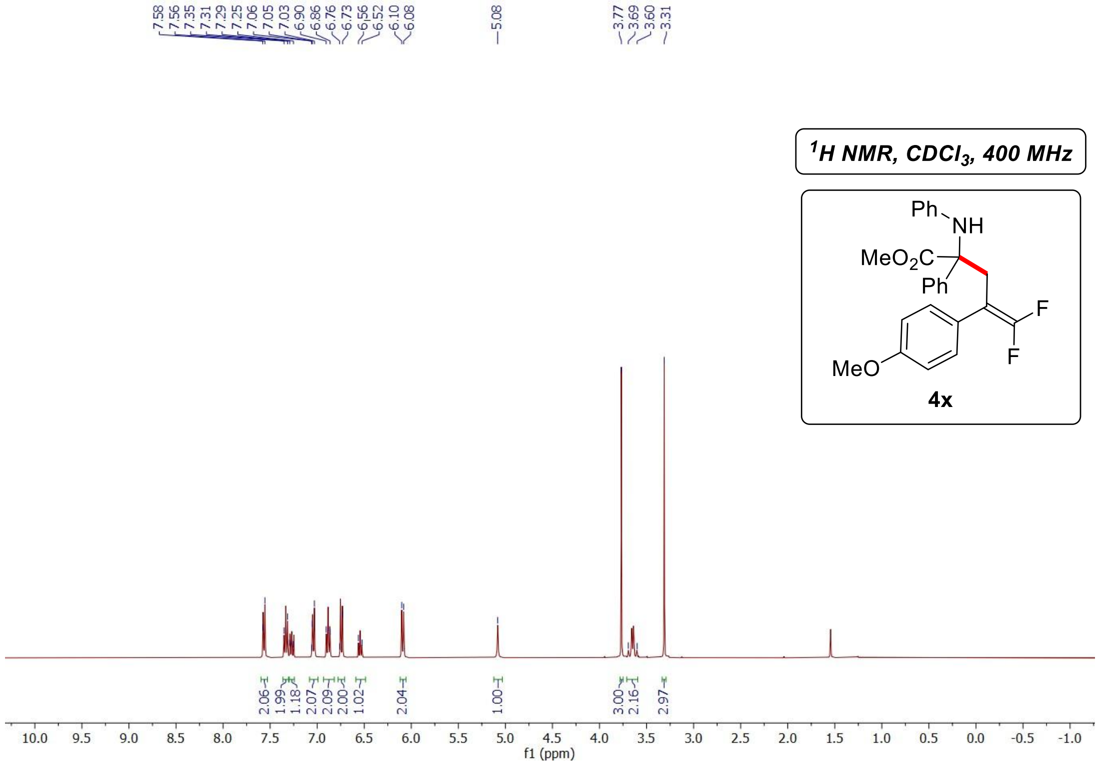

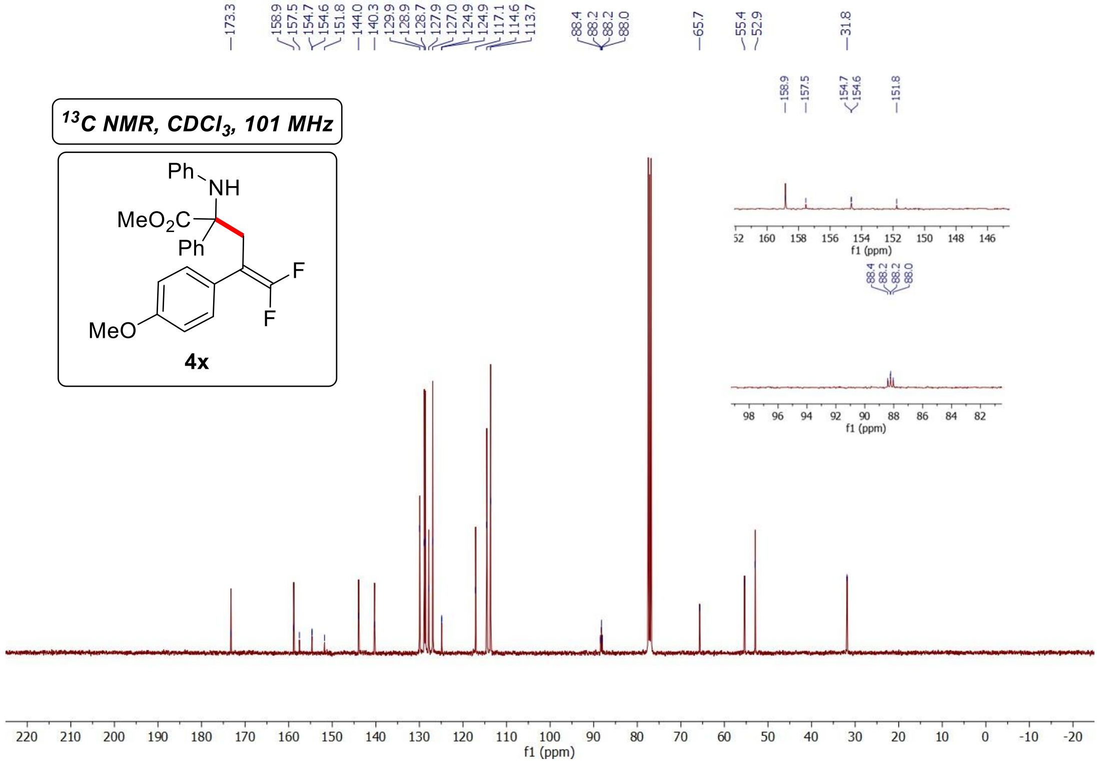

19F NMR, CDCl3, 376 MHz

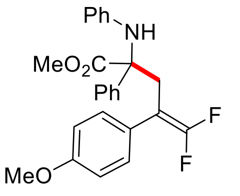

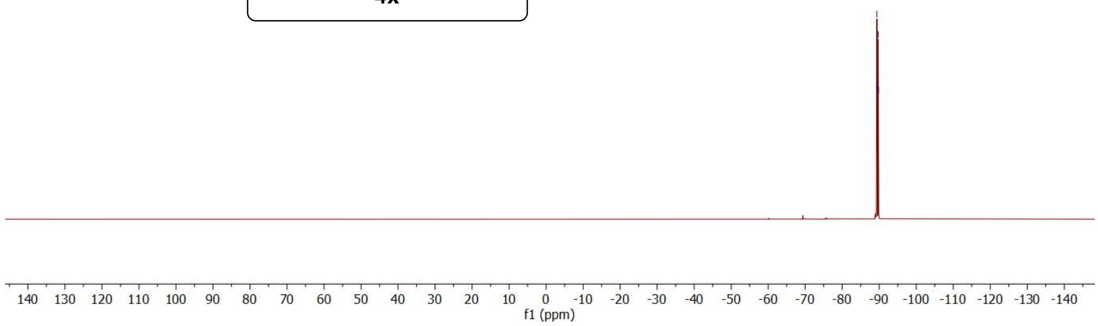

7.25. Methyl 4-(4-benzylphenyl)-5,5-difluoro-2-phenyl-2-(phenylamino)pent-4-enoate (4y)

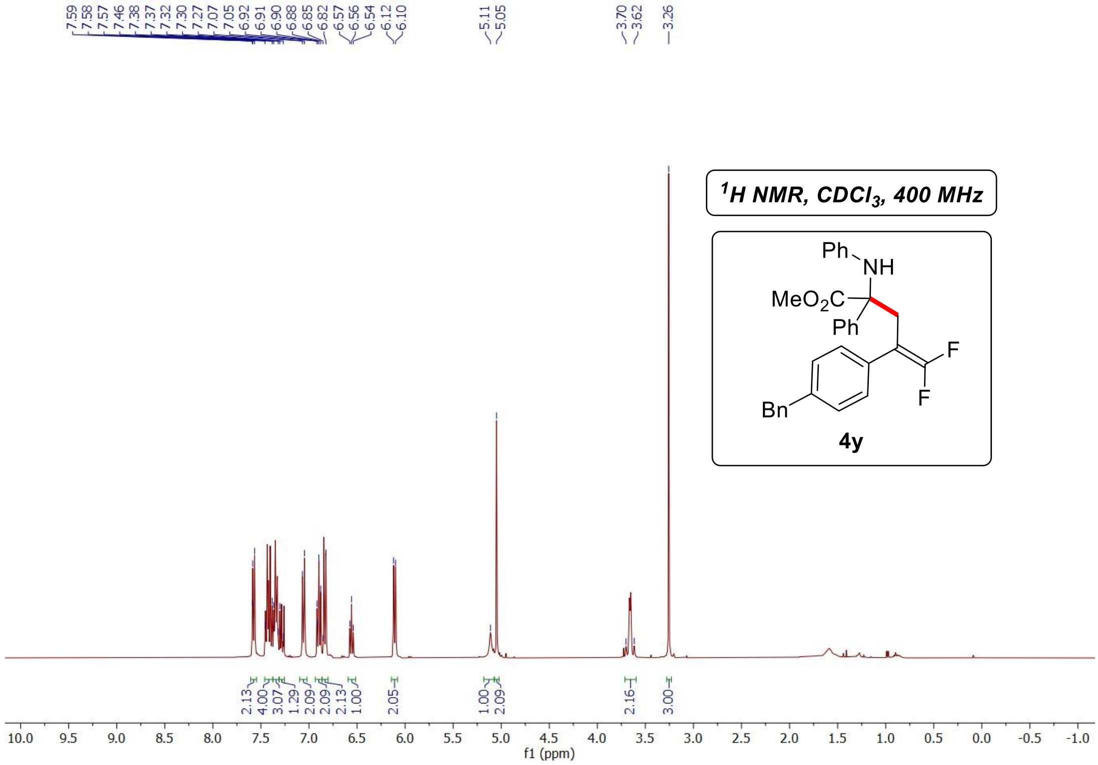

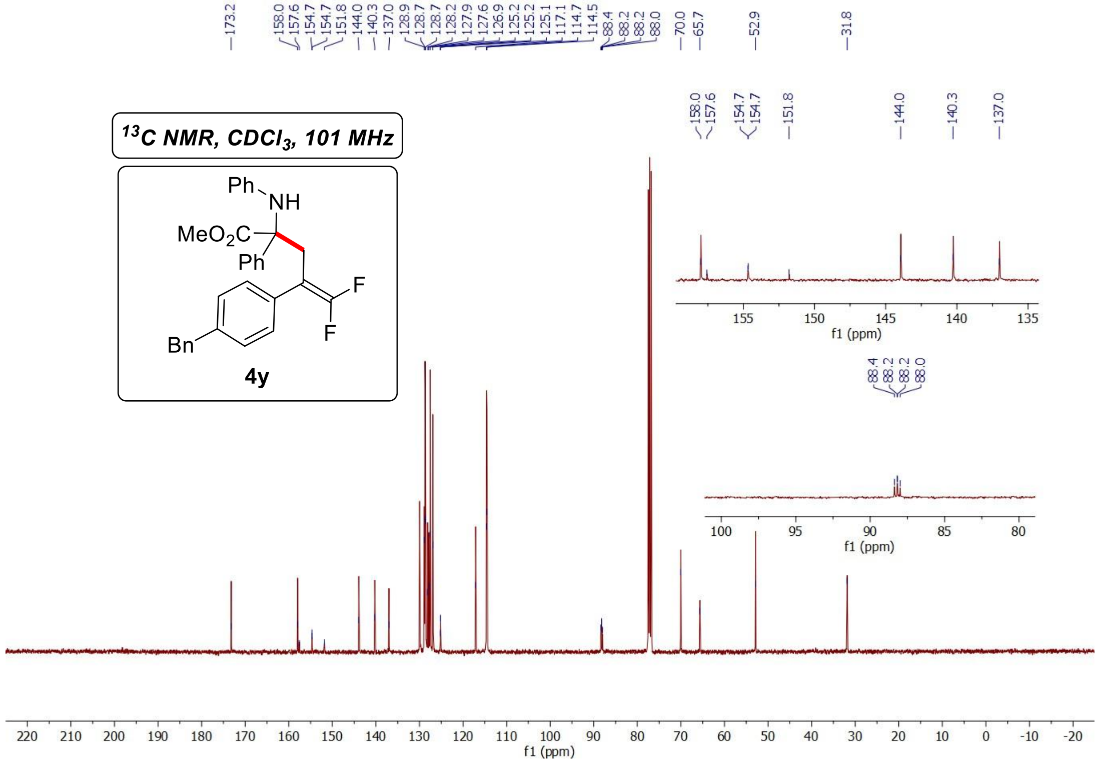

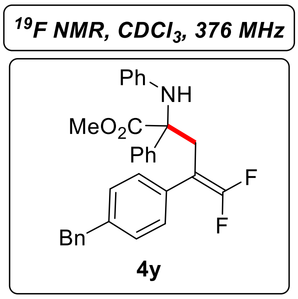
19F NMR, CDCl3, 376 MHz
Ph
NH
MeO2C
Ph
F
F
Bn
4y

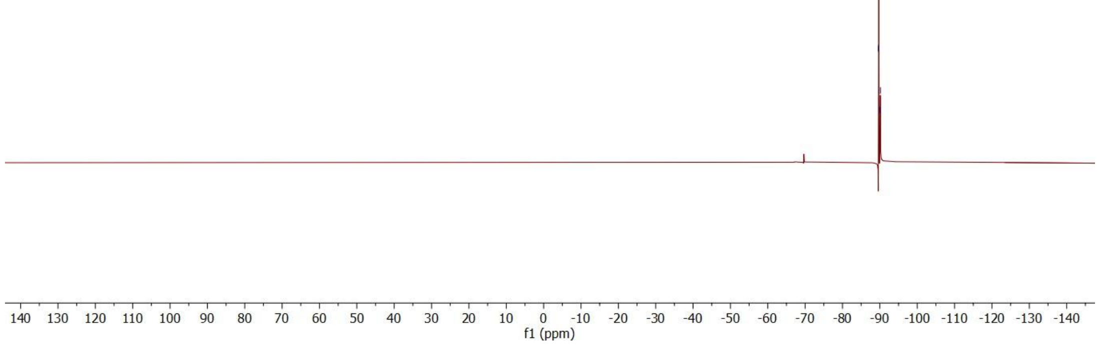
-89.62
-89.71
-90.05
-90.14
140 130 120 110 100 90 80 70 60 50 40 30 20 10 0 -10 -20 -30 -40 -50 -60 -70 -80 -90 -100 -110 -120 -130 -140
f1 (ppm)

7.26. Methyl 4-(4-bromophenyl)-5,5-difluoro-2-phenyl-2-(phenylamino)pent-4-enoate (4z)

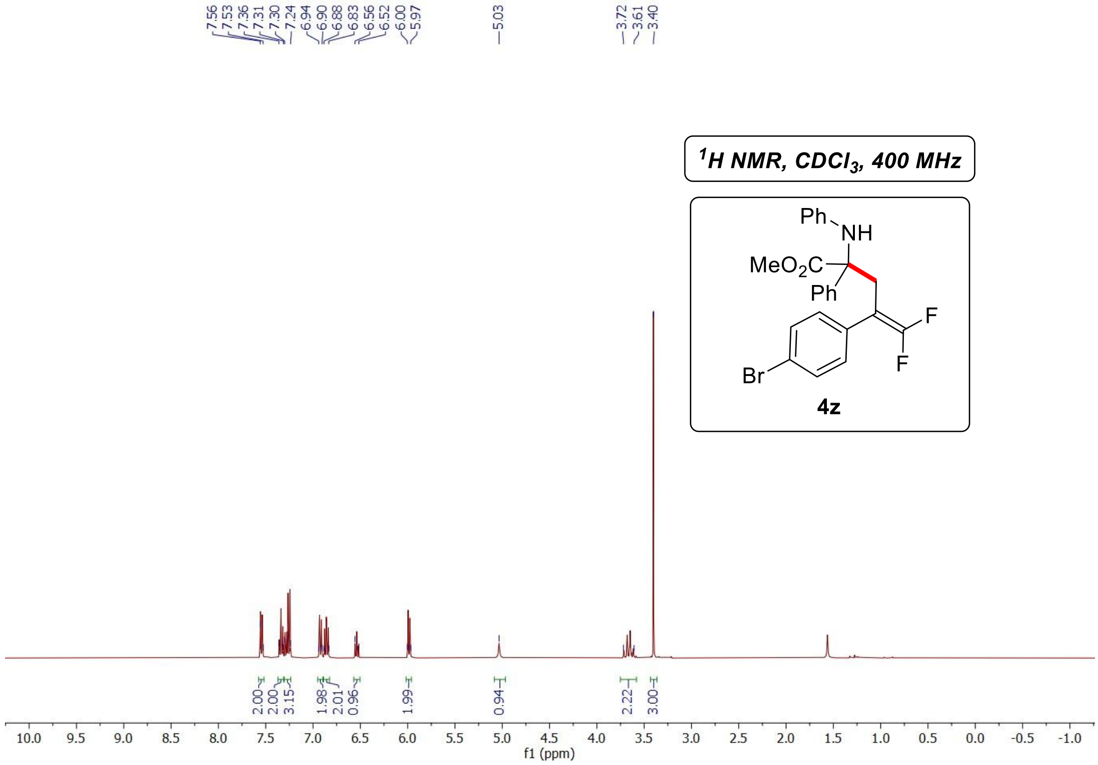

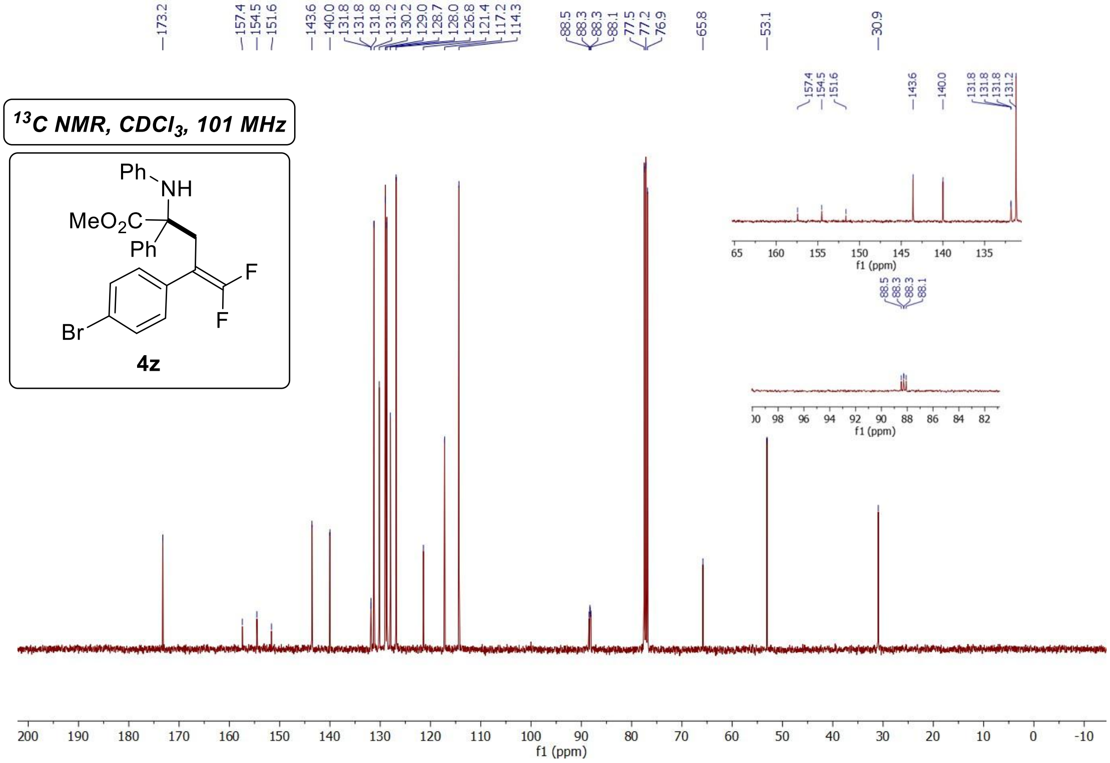

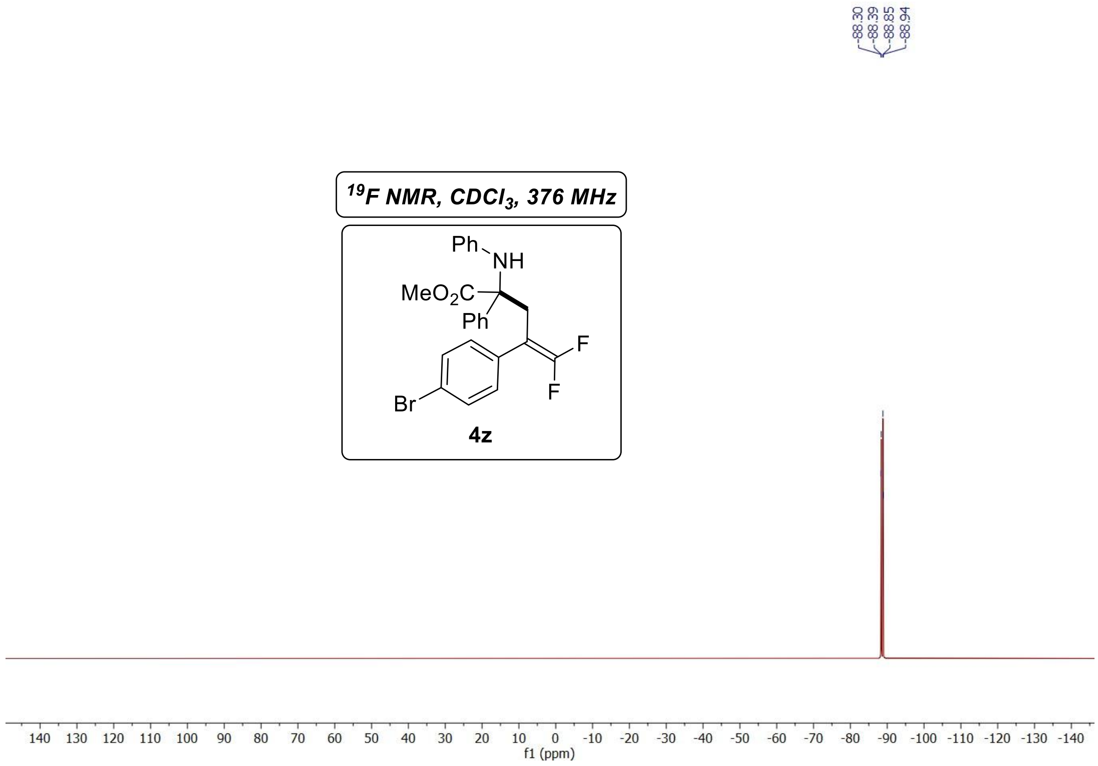
-88.30
-88.39
-88.85
-88.94
19F NMR, CDCl3, 376 MHz
Ph
NH
MeO2C
Ph
F
F
Br
4z
140 130 120 110 100 90 80 70 60 50 40 30 20 10 0 -10 -20 -30 -40 -50 -60 -70 -80 -90 -100 -110 -120 -130 -140
f1 (ppm)

7.27. Methyl 4-(3,5-dichlorophenyl)-5,5-difluoro-2-phenyl-2-(phenylamino)pent-4-enoate (4aa)

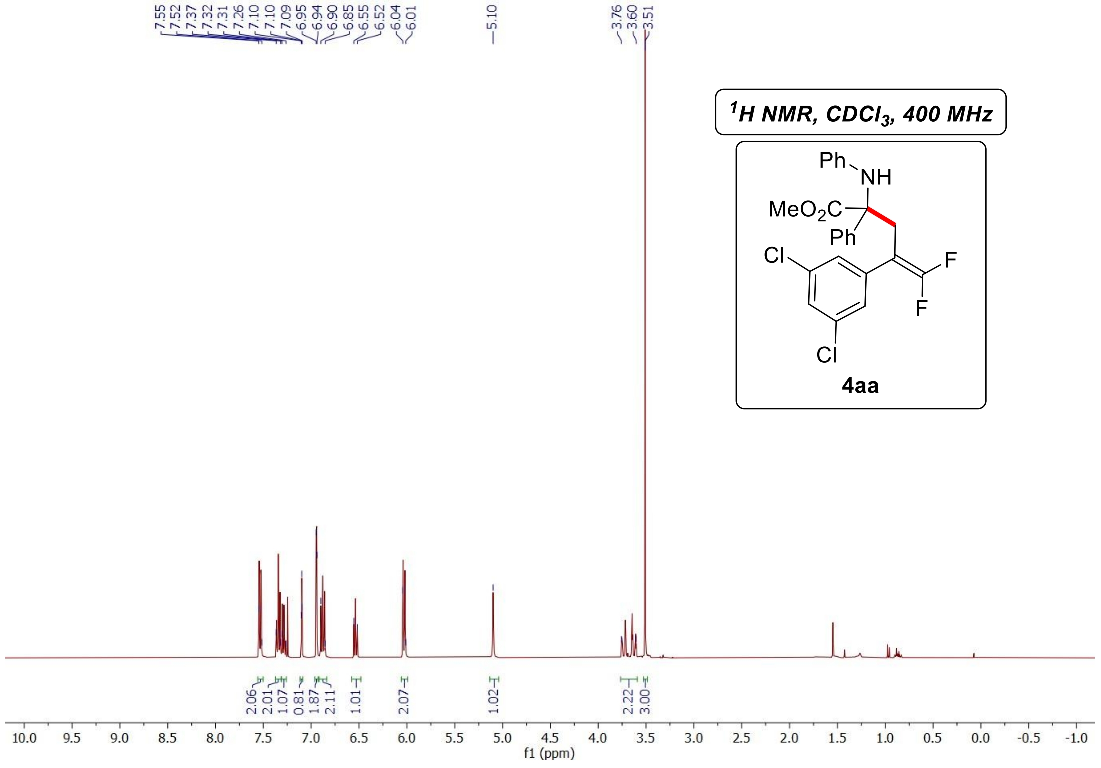

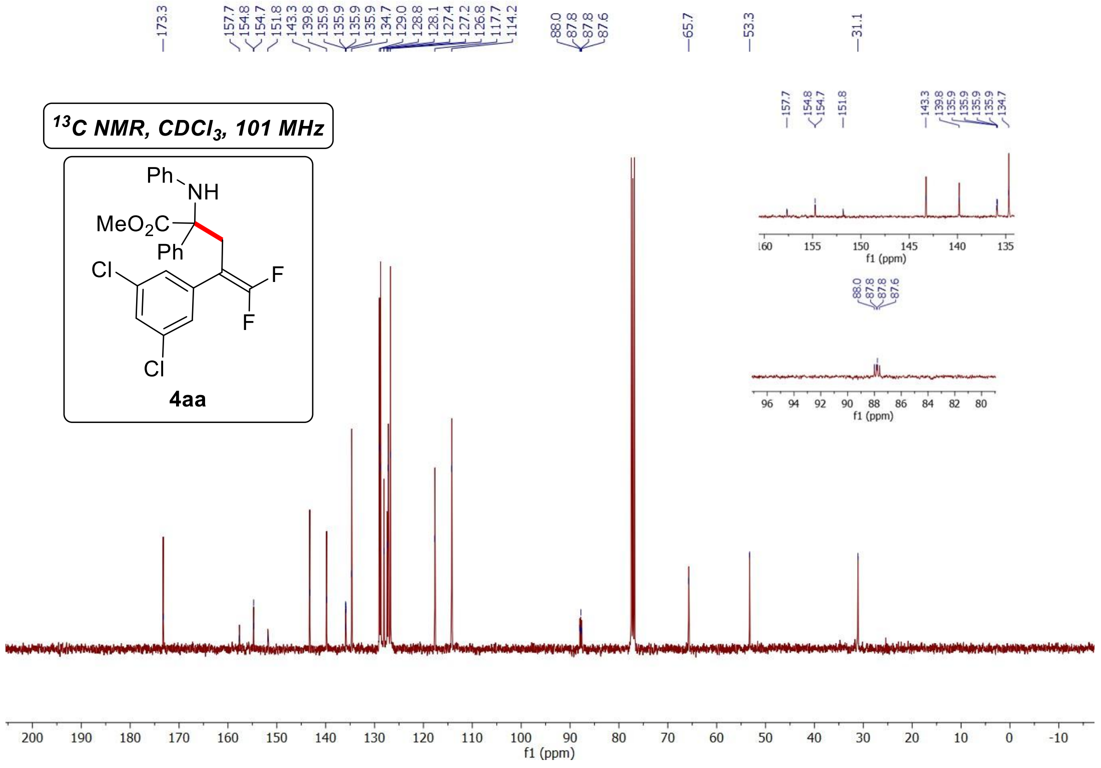

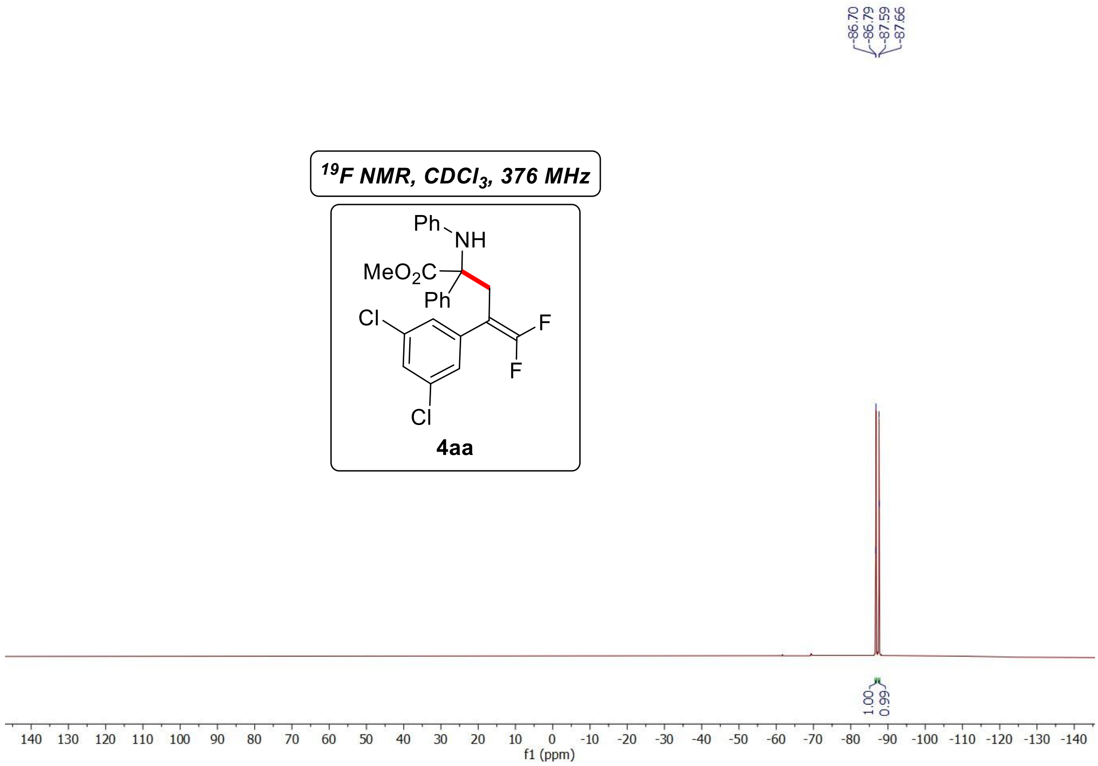
-86.70
-86.79
-87.59
-87.66
19F NMR, CDCl3, 376 MHz
Ph
NH
MeO2C
Ph
Cl
F
F
Cl
4aa
1.00
0.99
140 130 120 110 100 90 80 70 60 50 40 30 20 10 0 -10 -20 -30 -40 -50 -60 -70 -80 -90 -100 -110 -120 -130 -140
f1 (ppm)

7.28. Methyl 3-methyl-2-phenyl-2-(phenylamino)butanoate (6a)

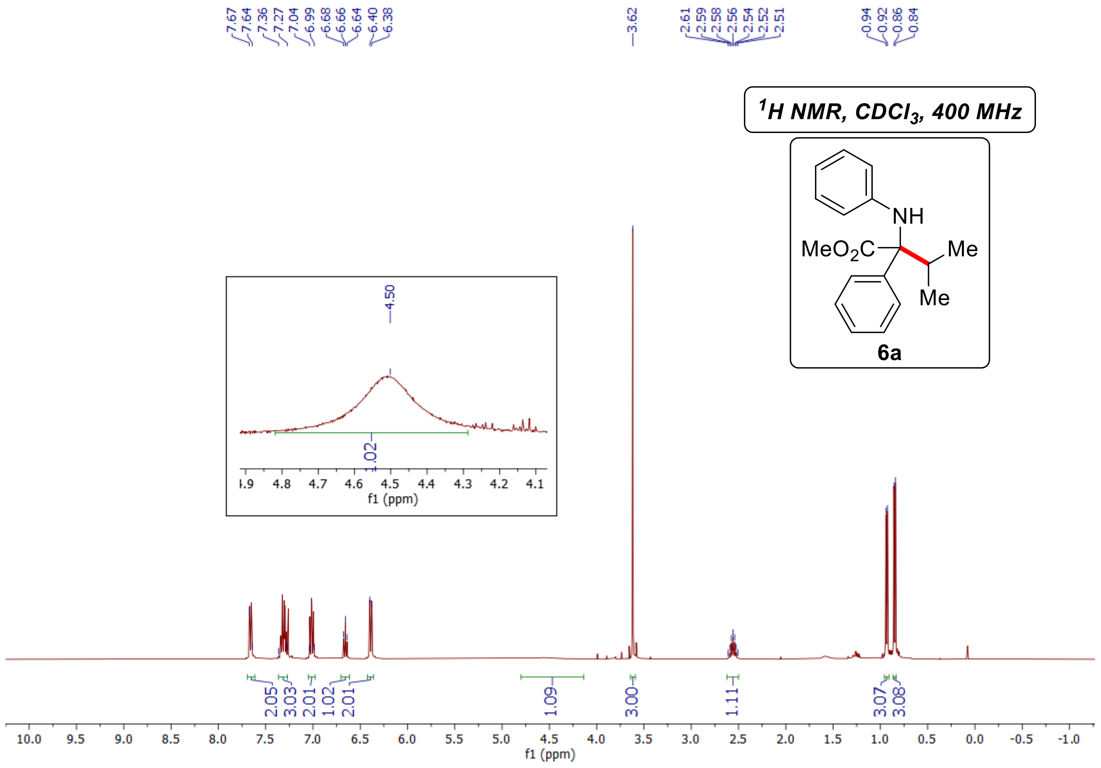

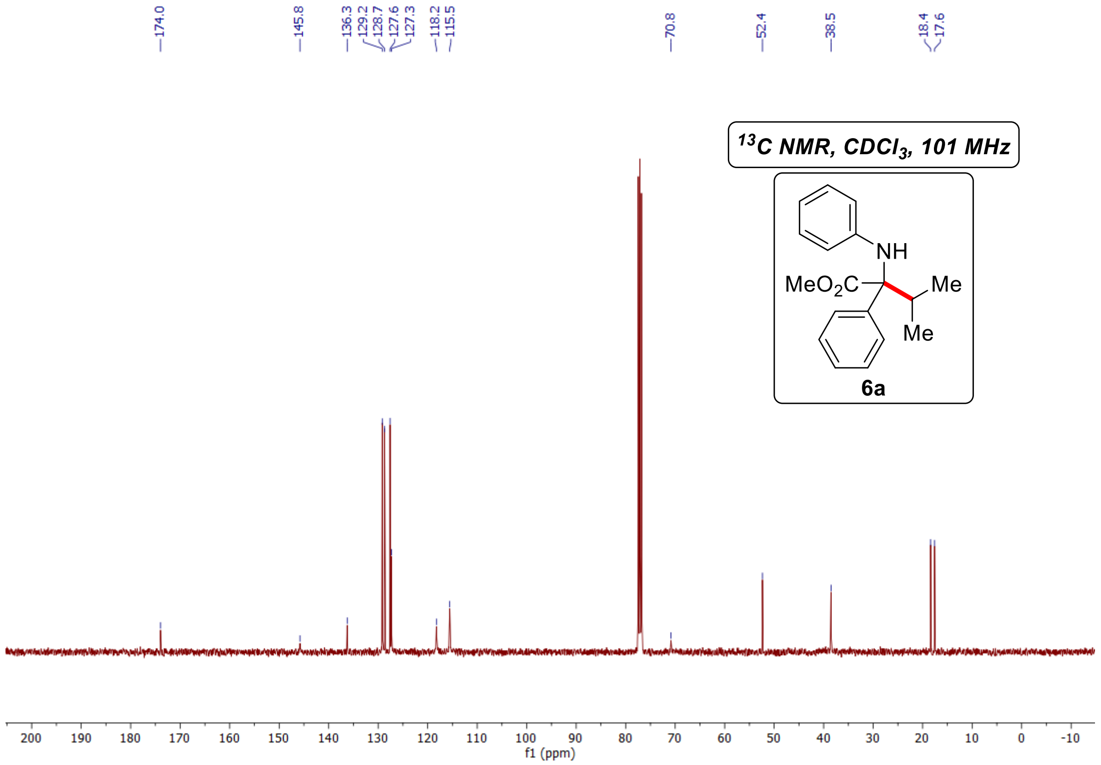

7.29 Benzyl 3-methyl-2-phenyl-2-(phenylamino)butanoate (6b)

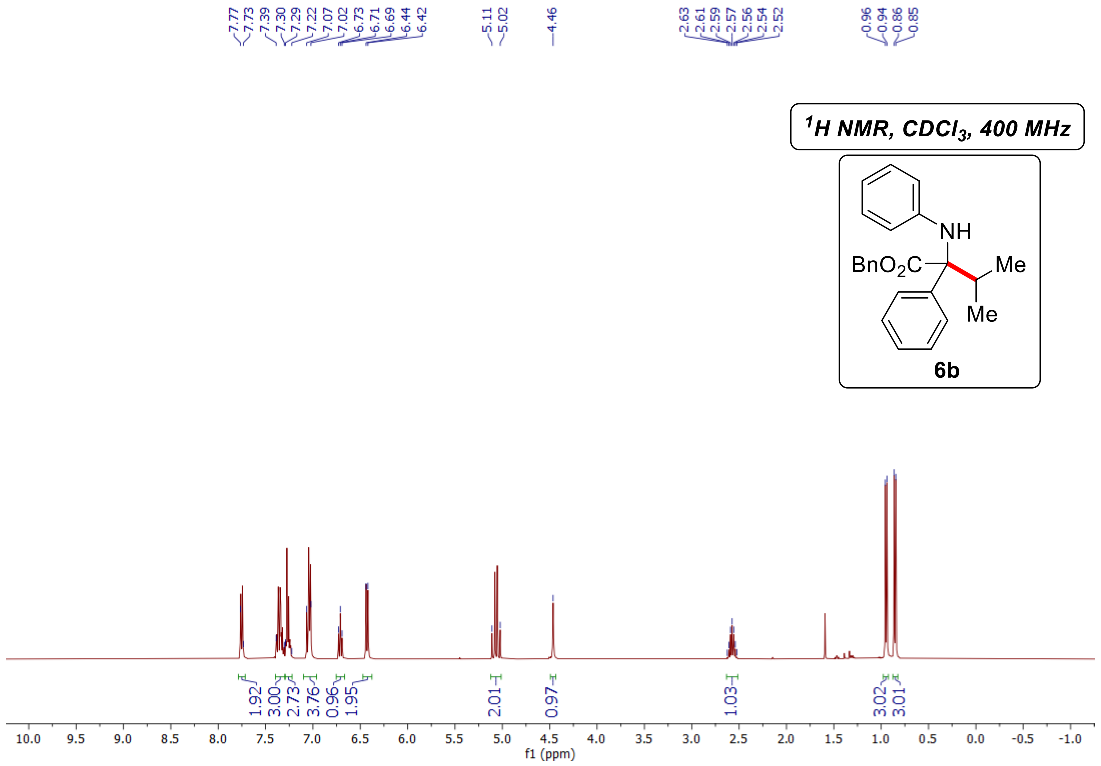

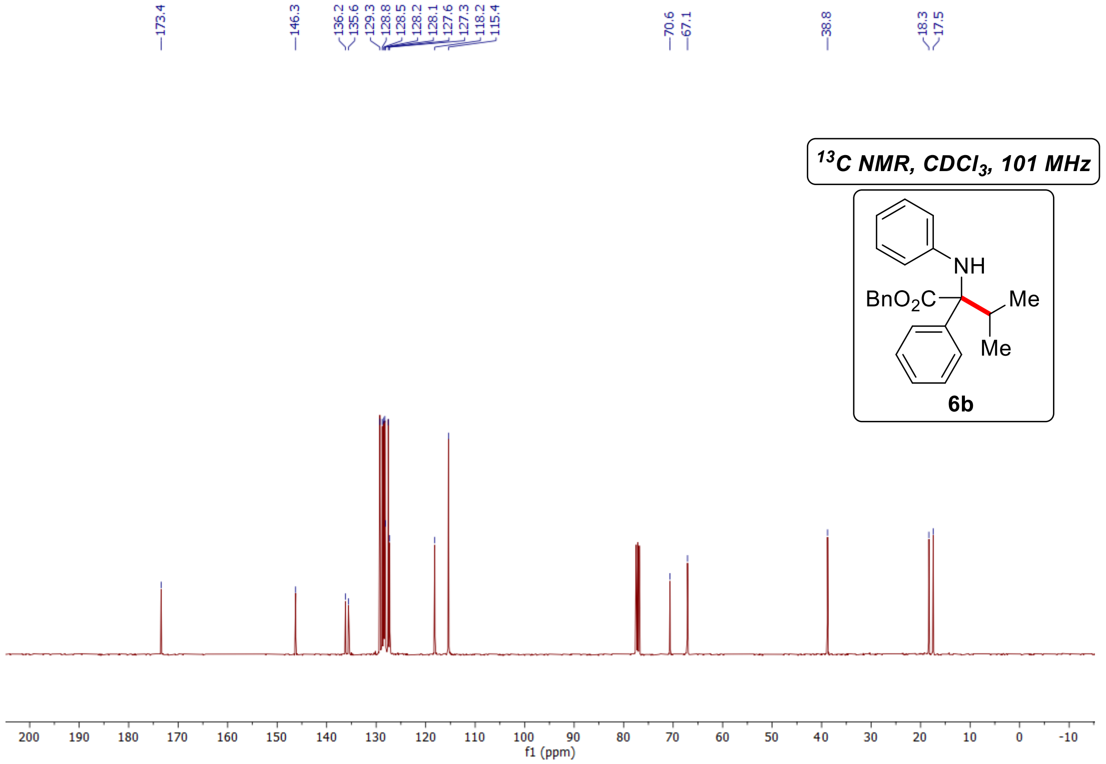

7.30 Isopropyl 3-methyl-2-phenyl-2-(phenylamino)butanoate (6c)

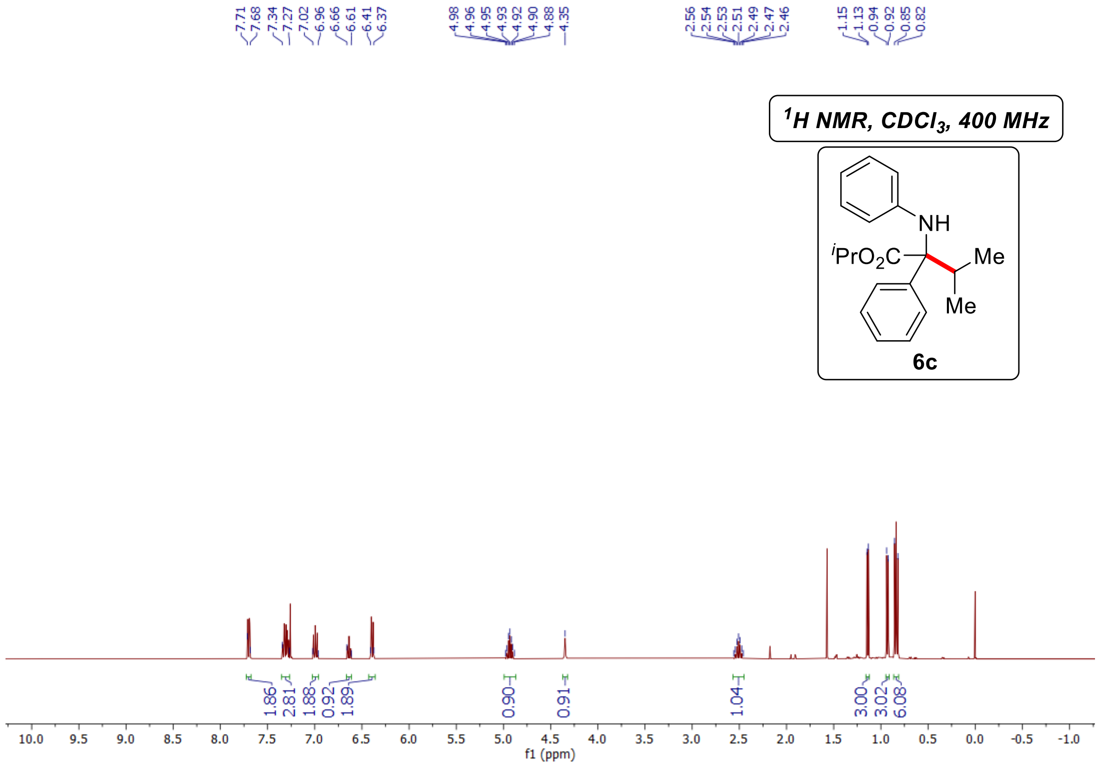

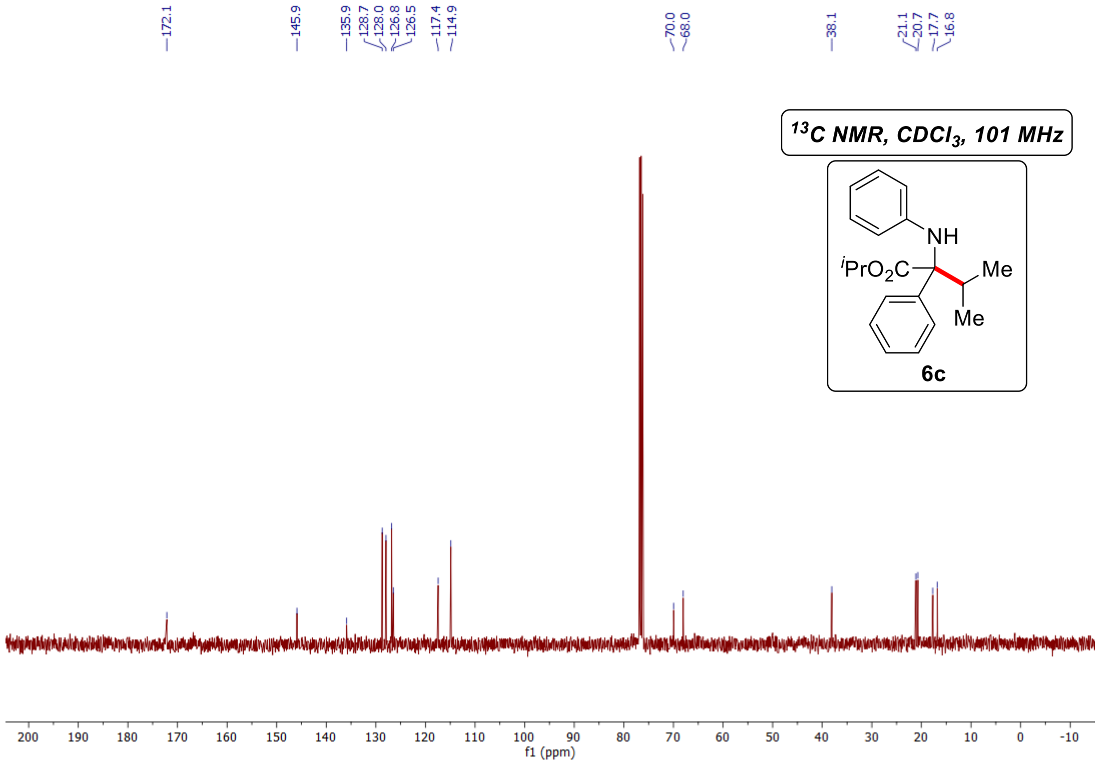

7.31 Prop-2-yn-1-yl 3-methyl-2-phenyl-2-(phenylamino)butanoate (6d)

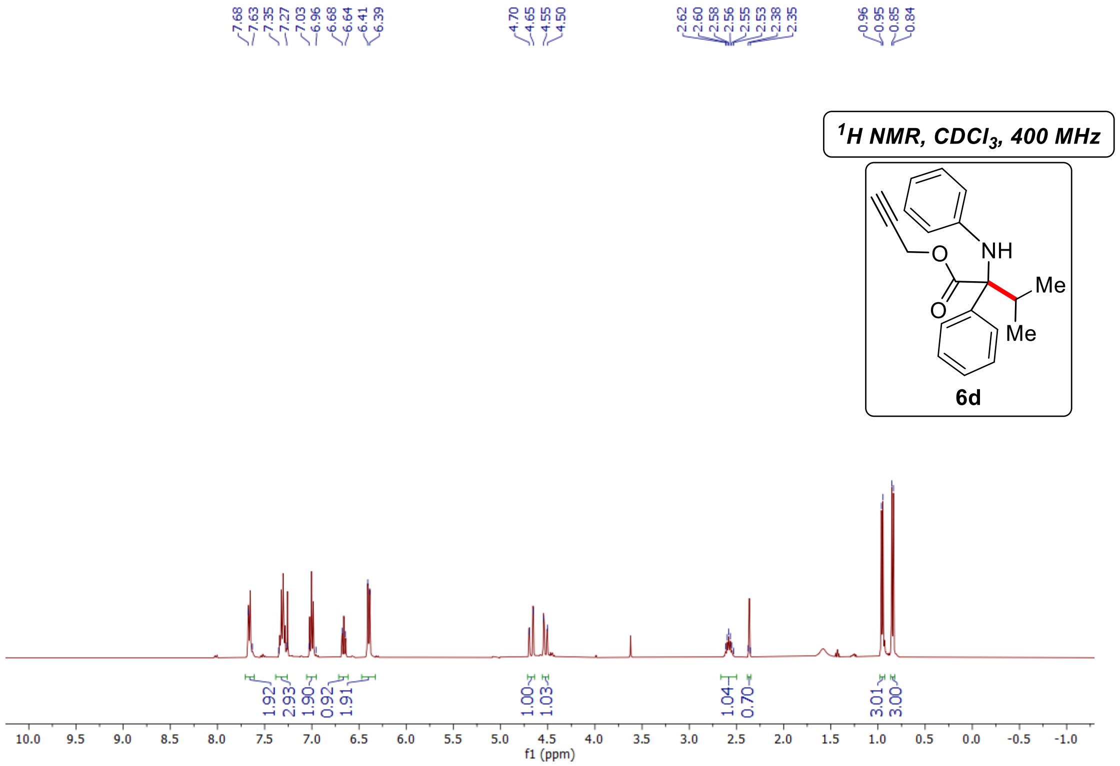

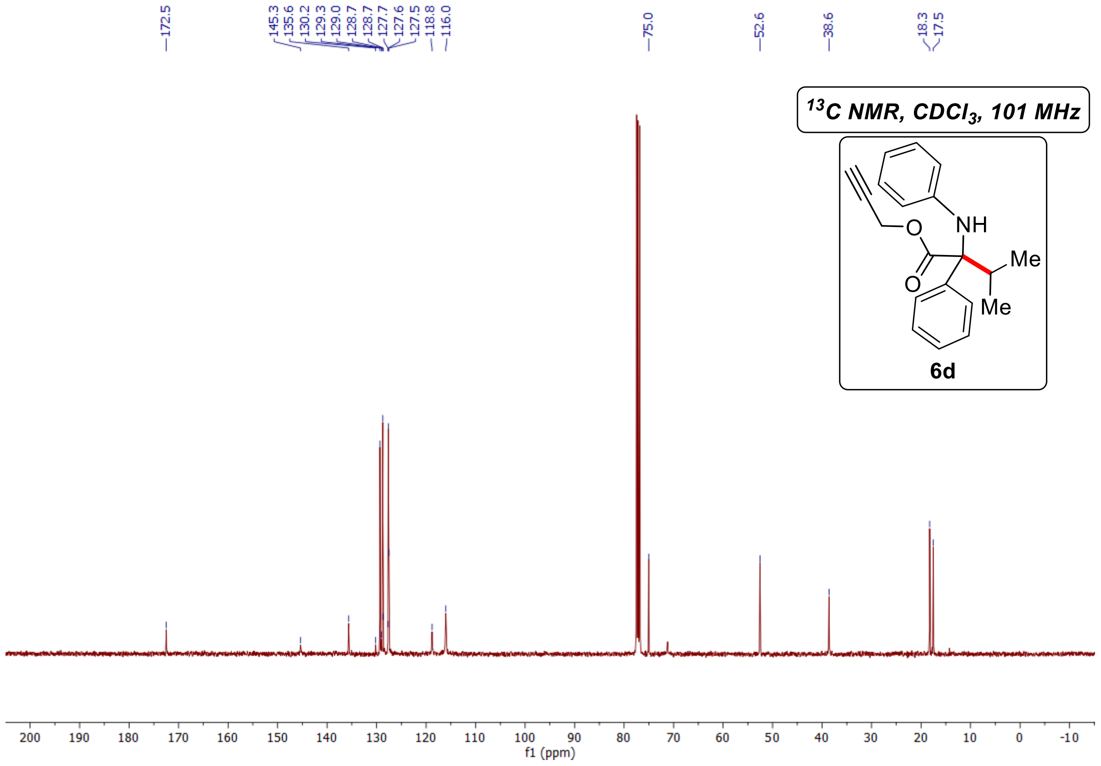

7.32 But-3-en-1-yl 3-methyl-2-phenyl-2-(phenylamino)butanoate (6e)

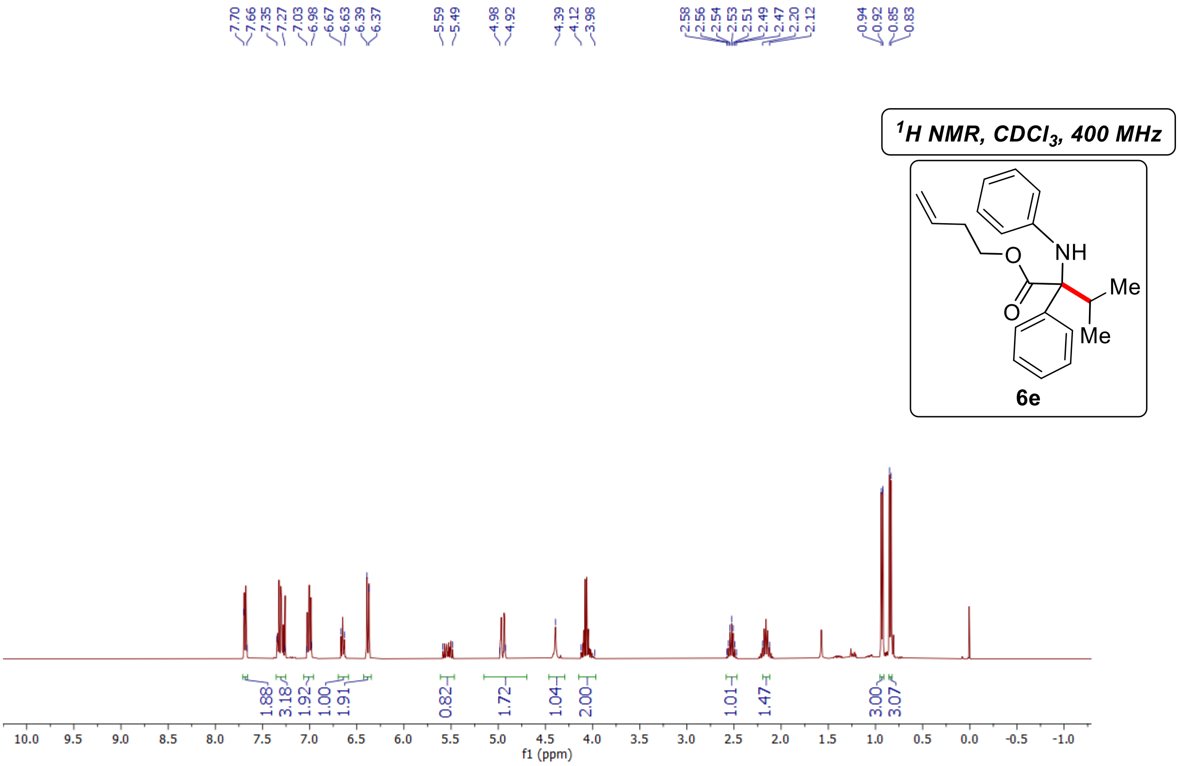

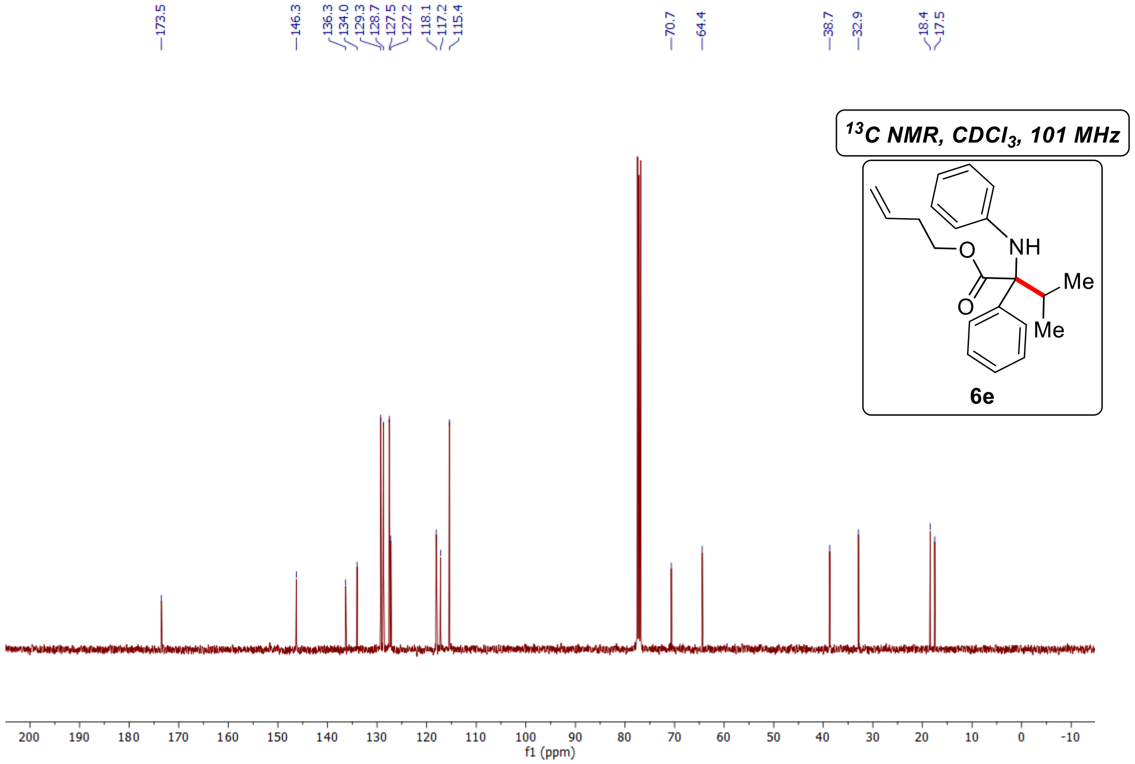

7.33 (1*S*,2*R*,5*S*)-2-Isopropyl-5-methylcyclohexyl 3-methyl-2-phenyl-2-(phenylamino)butanoate (6f)

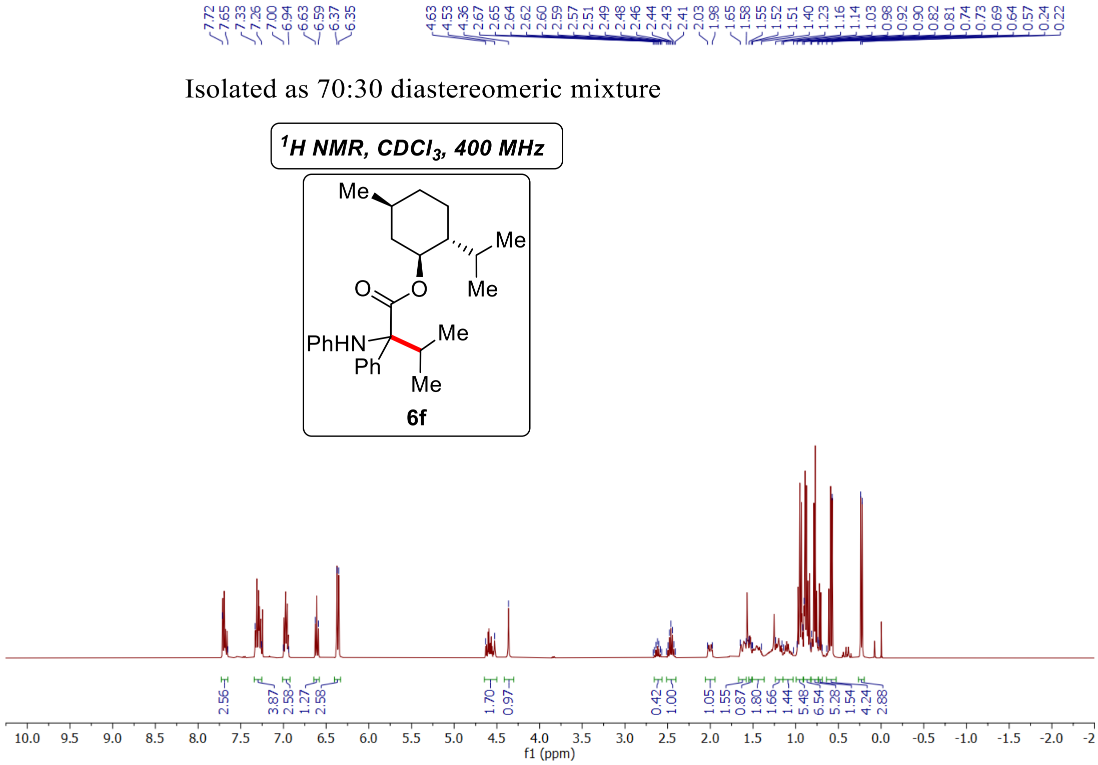

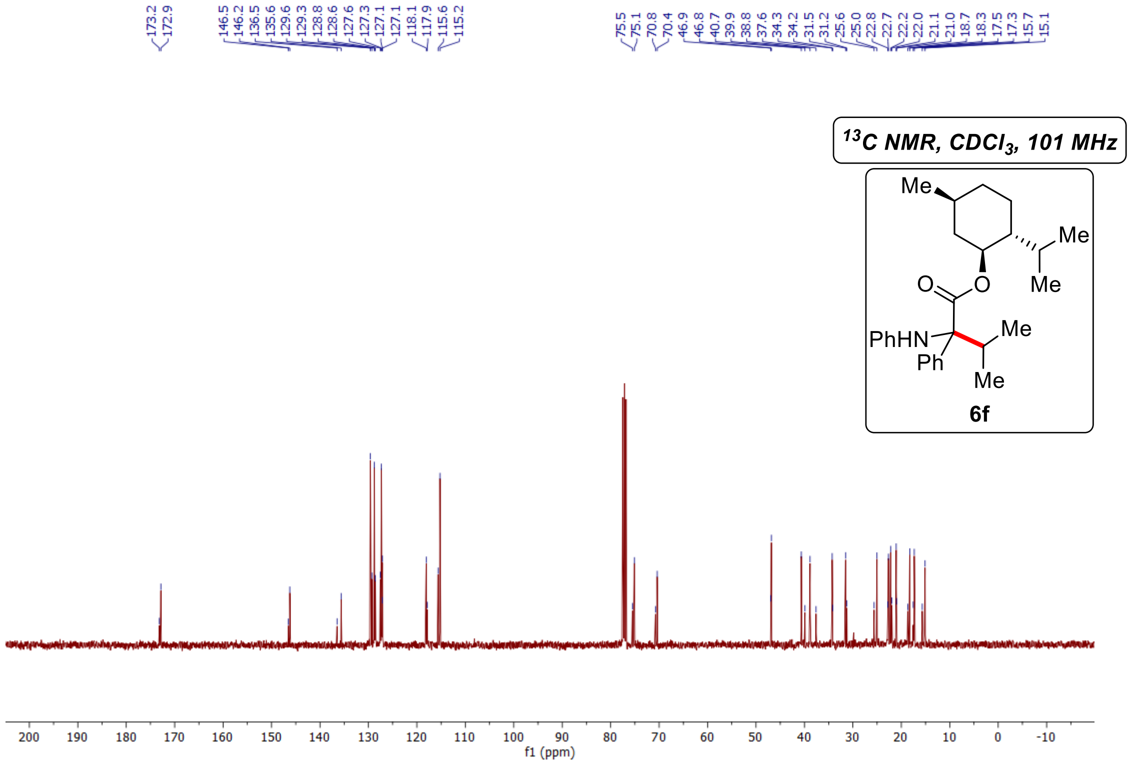

7.34 (3*S*,8*S*,9*S*,10*R*,13*R*,14*S*,16*S*)-10,13-Dimethyl-16-((*S*)-6-methylheptan-2-yl)-2,3,4,7,8,9,10,11,12,13,14,15,16,17-tetradecahydro-1H-cyclopenta[a]phenanthren-3-yl 3-methyl-2-phenyl-2-(phenylamino)butanoate (6g)

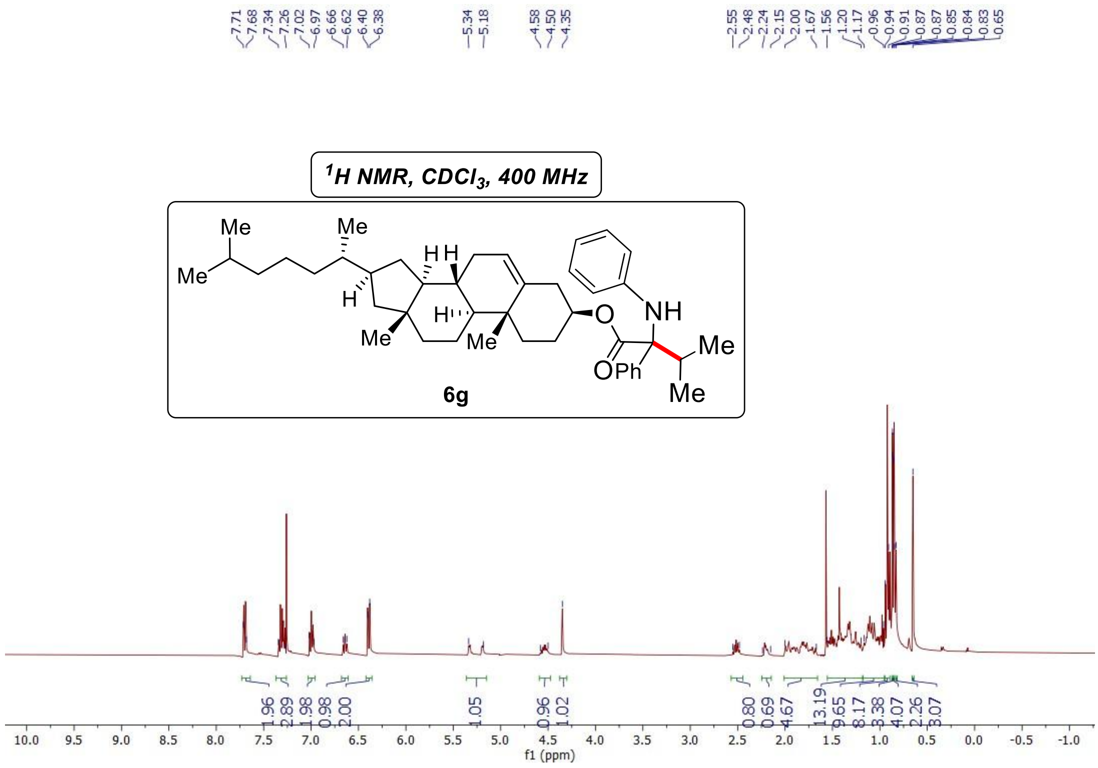

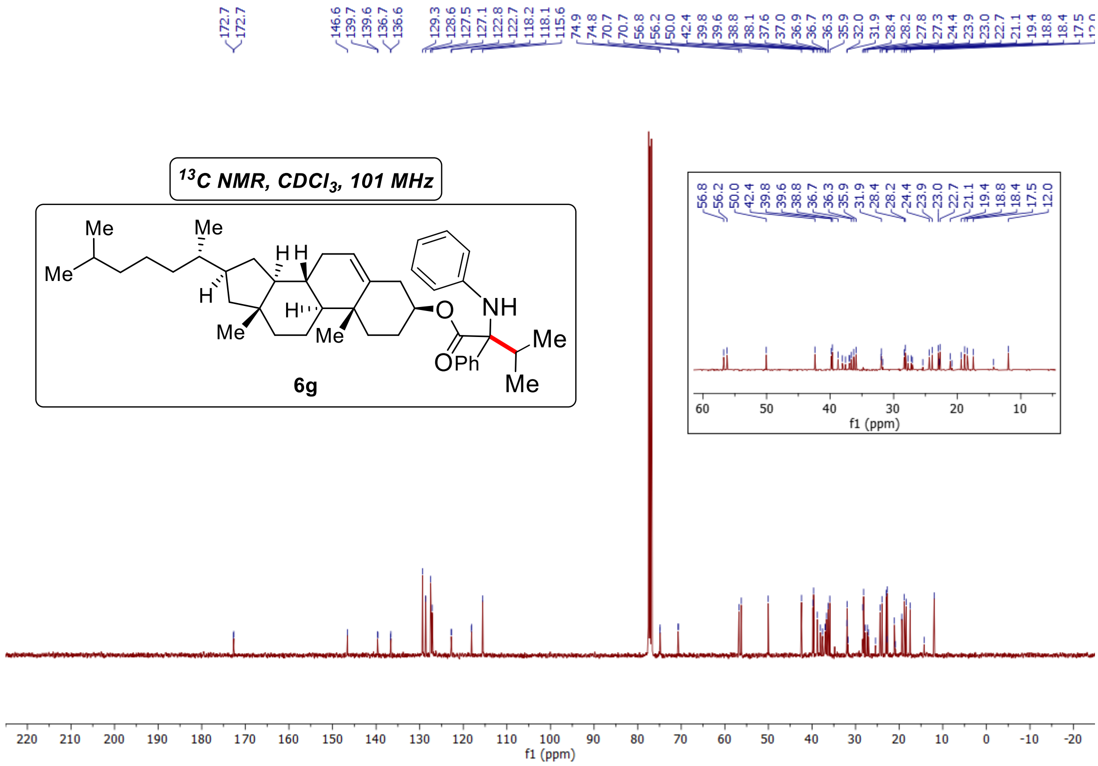

7.35 Methyl 2-(4-bromophenyl)-3-methyl-2-(phenylamino)butanoate (6h)

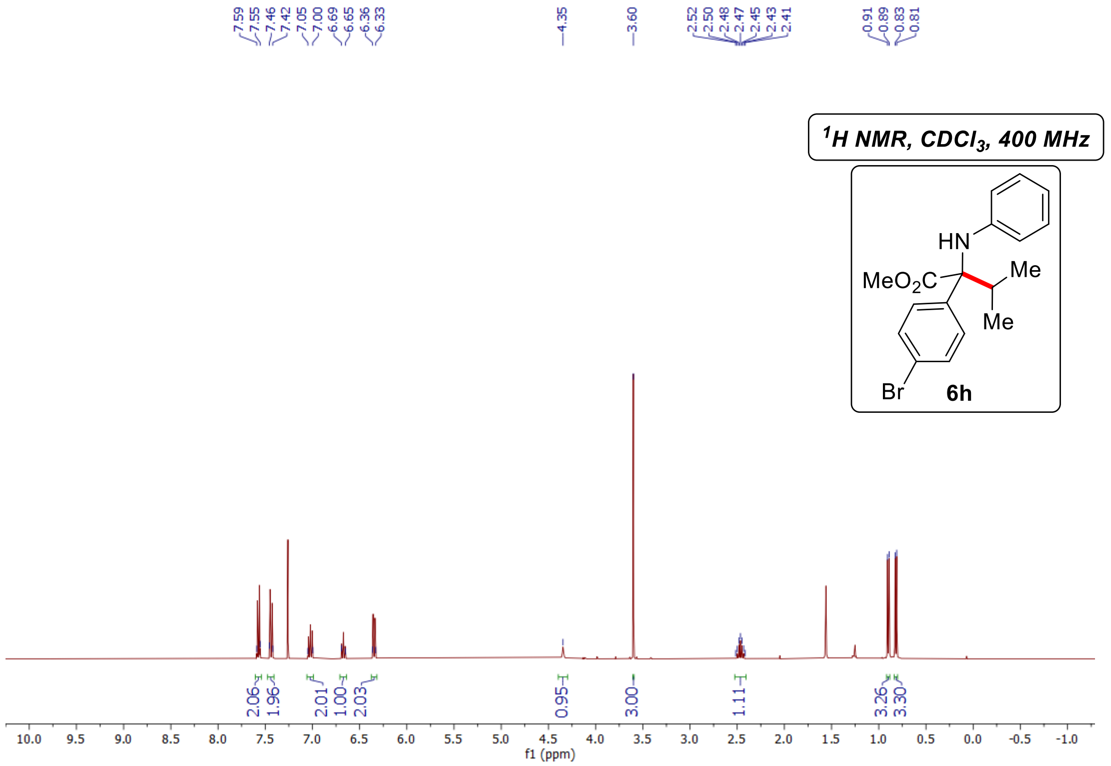

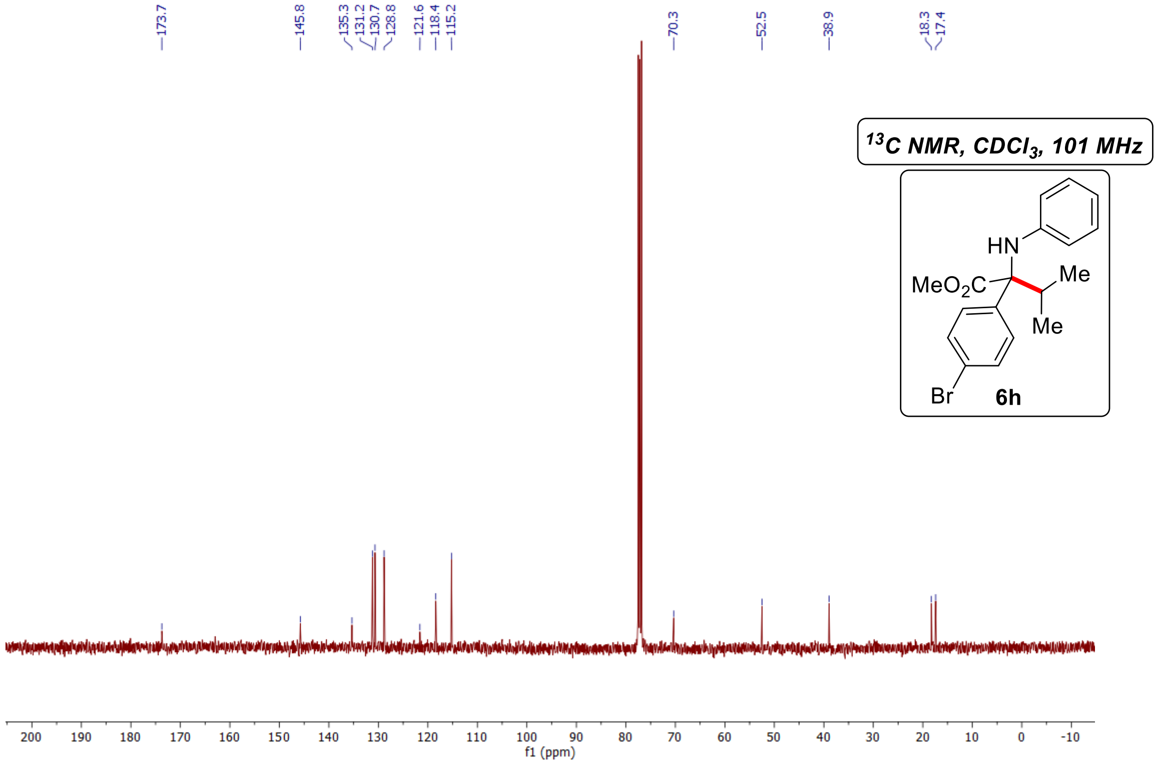

7.36 Methyl 3-methyl-2-(phenylamino)-2-(4-(trifluoromethyl)phenyl)butanoate (6i)

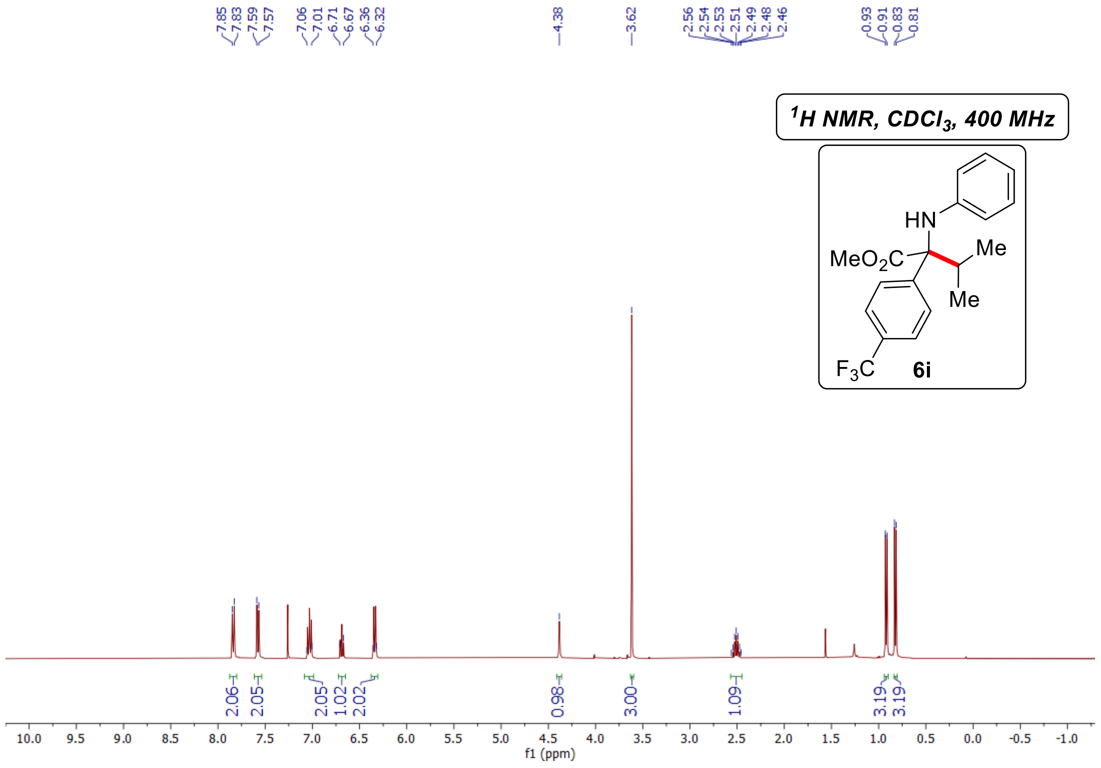

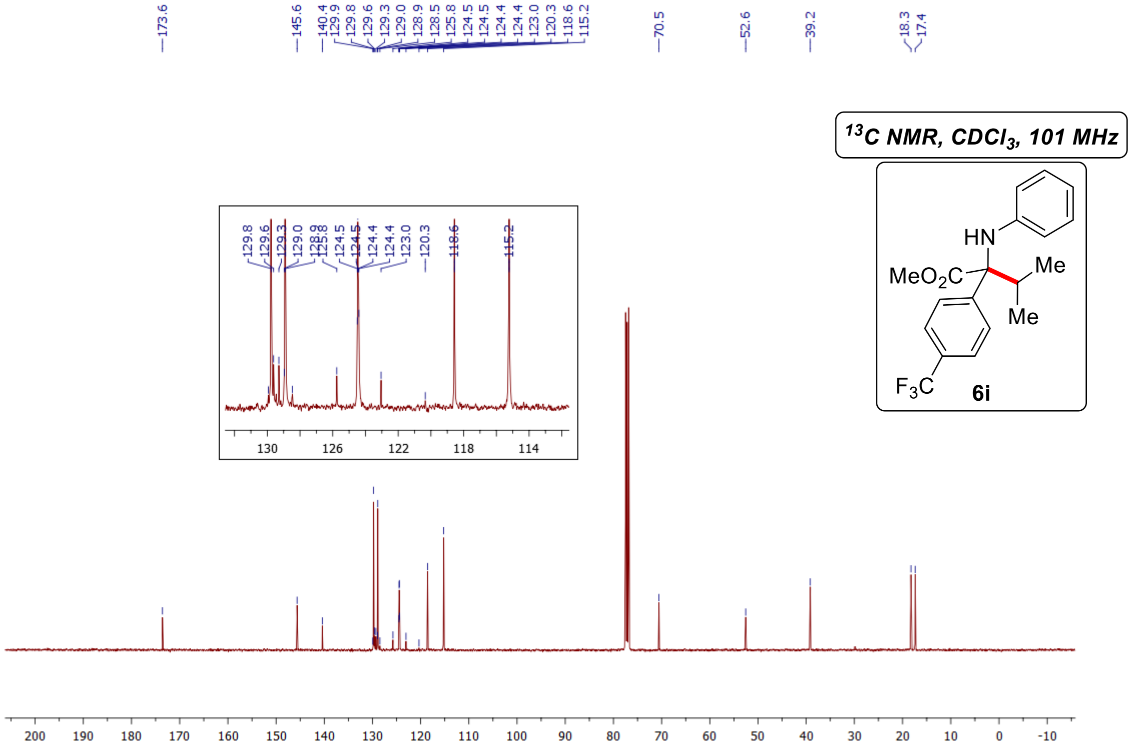

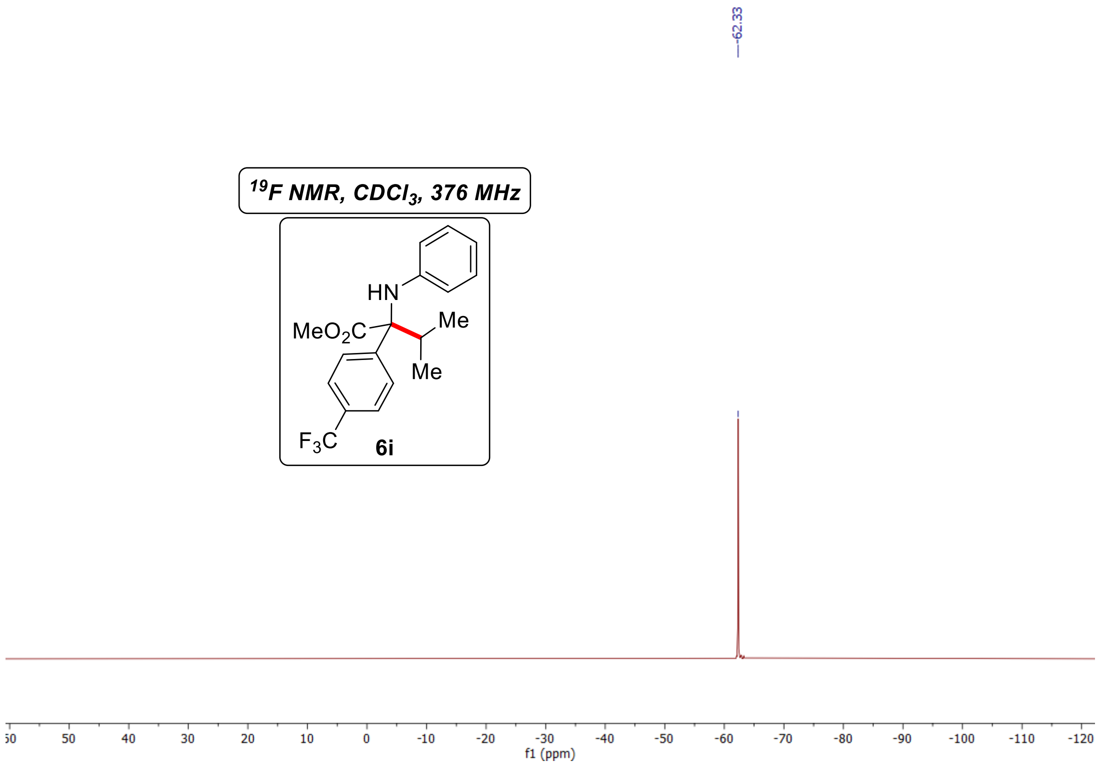
-62.33
19F NMR, CDCl3, 376 MHz
HN
Me
MeO2C
Me
F3C
6i
f1 (ppm)

7.37 Methyl 3-methyl-2-(phenylamino)-2-(thiophen-2-yl)butanoate (6j)

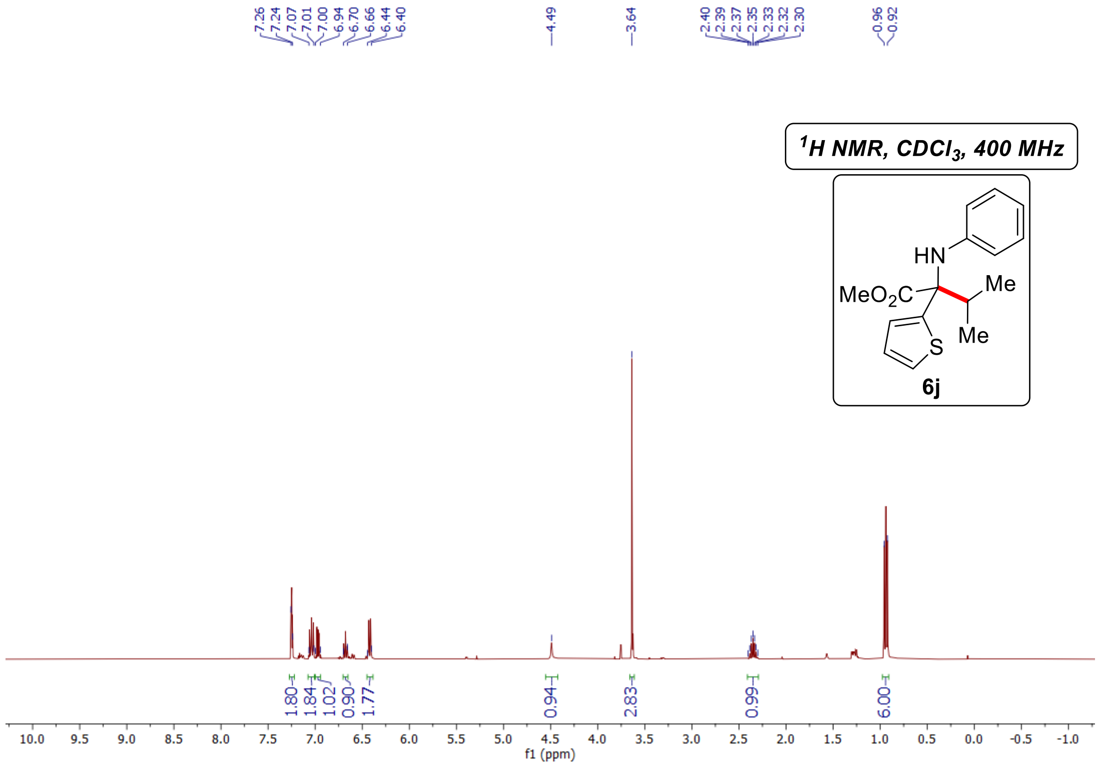

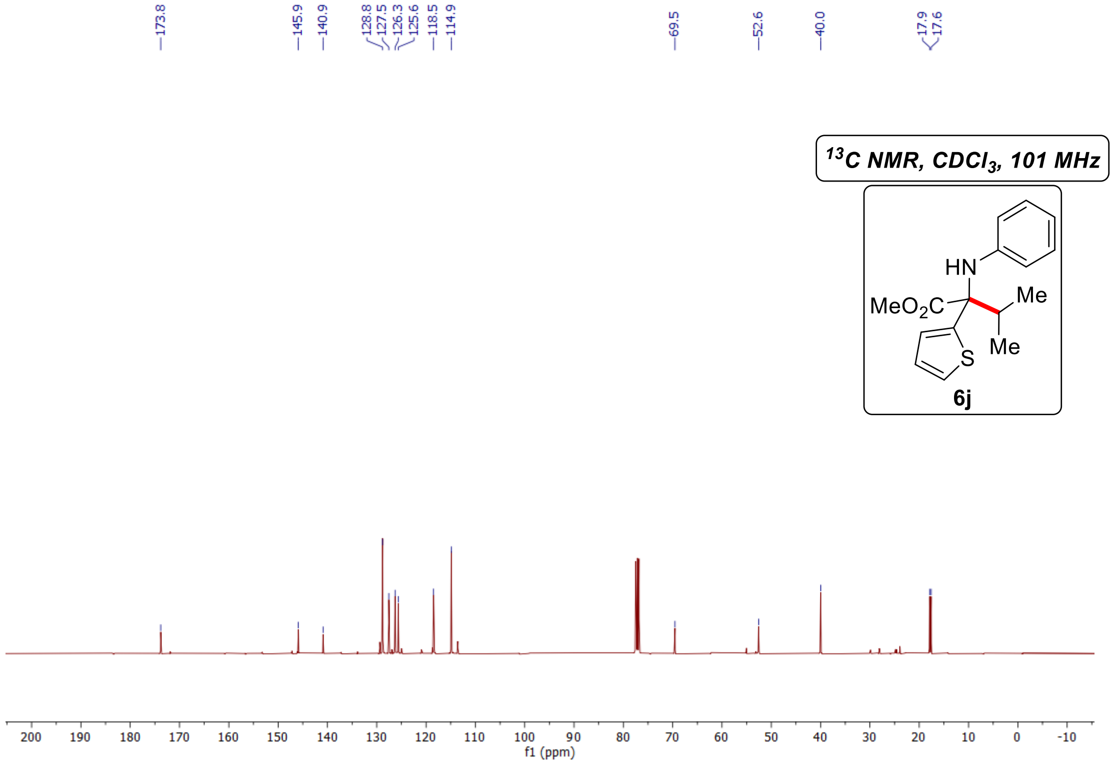

7.38 Methyl 2-((4-methoxyphenyl)amino)-3-methyl-2-phenylbutanoate (6k)

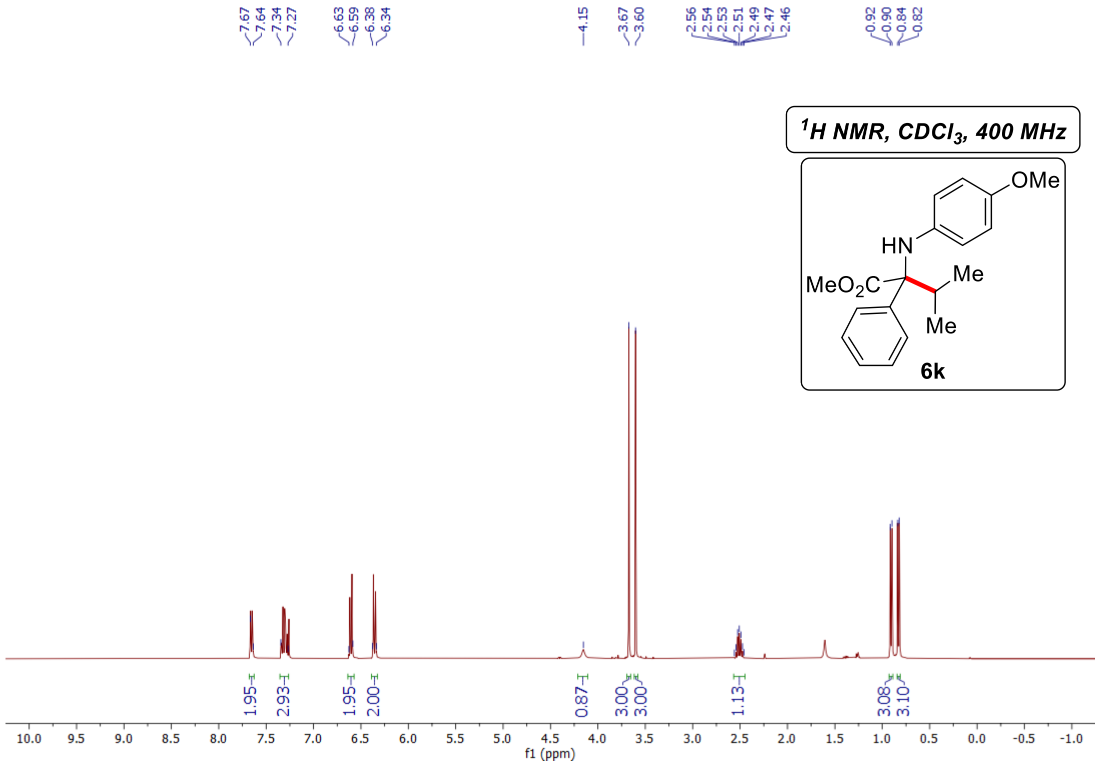

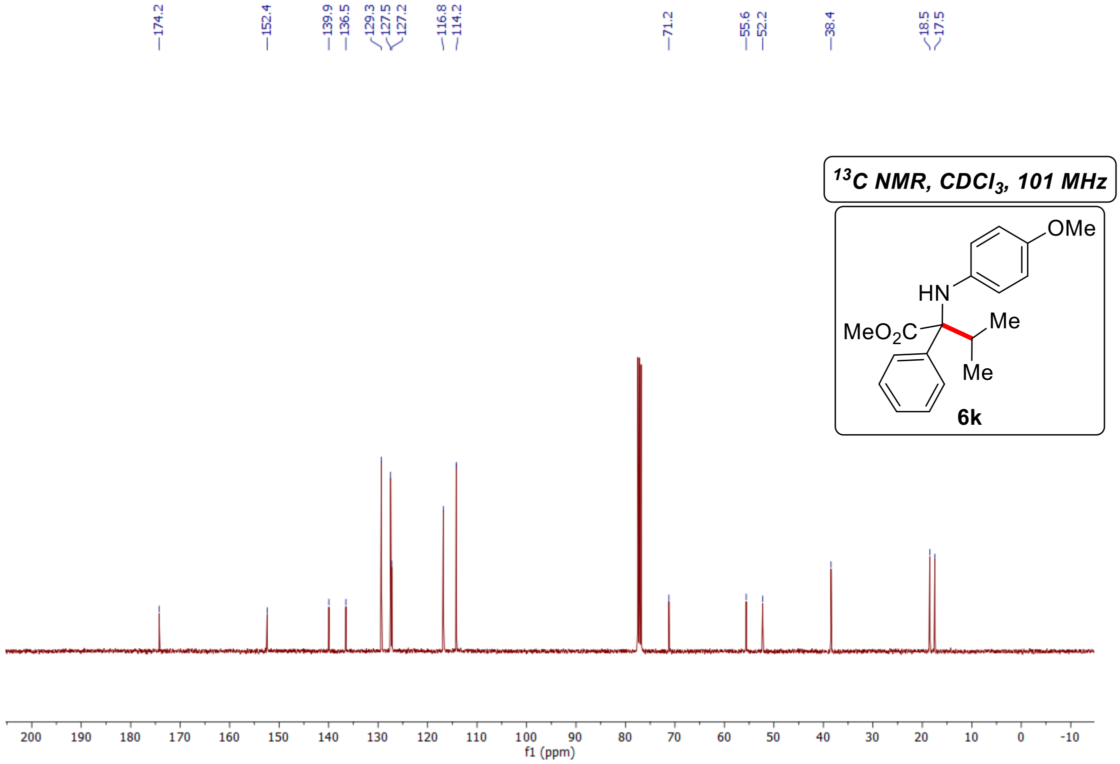

7.39 Methyl 2-((4-chlorophenyl)amino)-3-methyl-2-phenylbutanoate (6l)

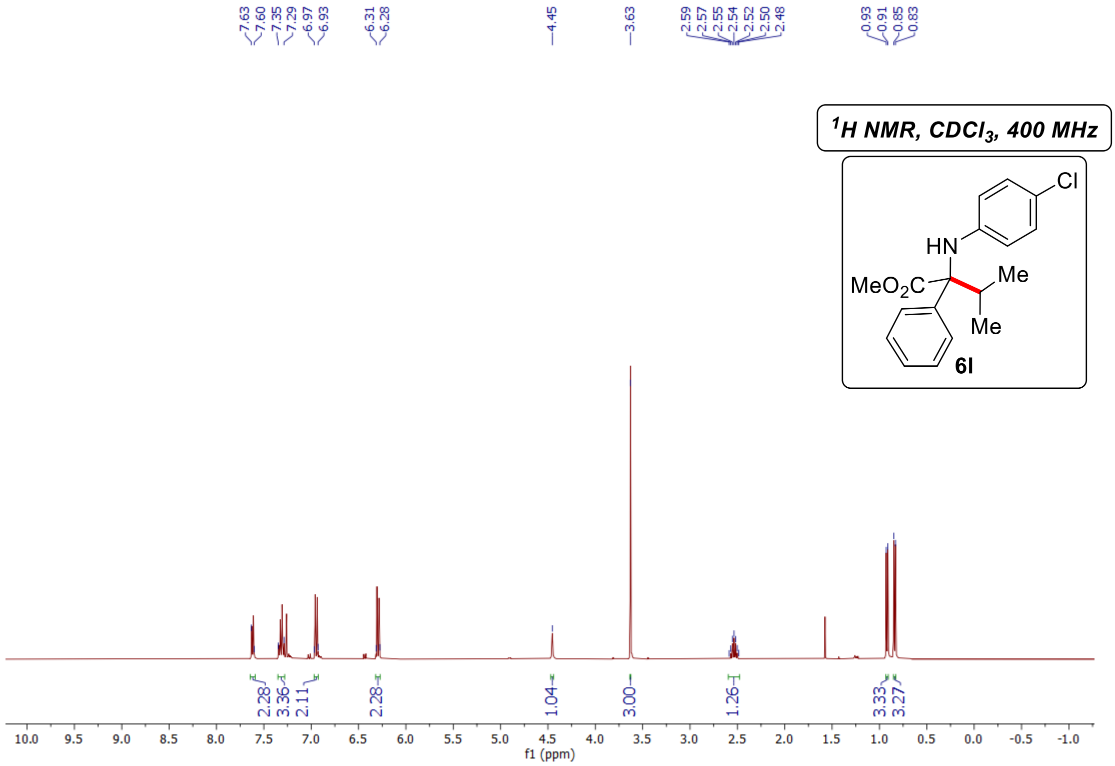

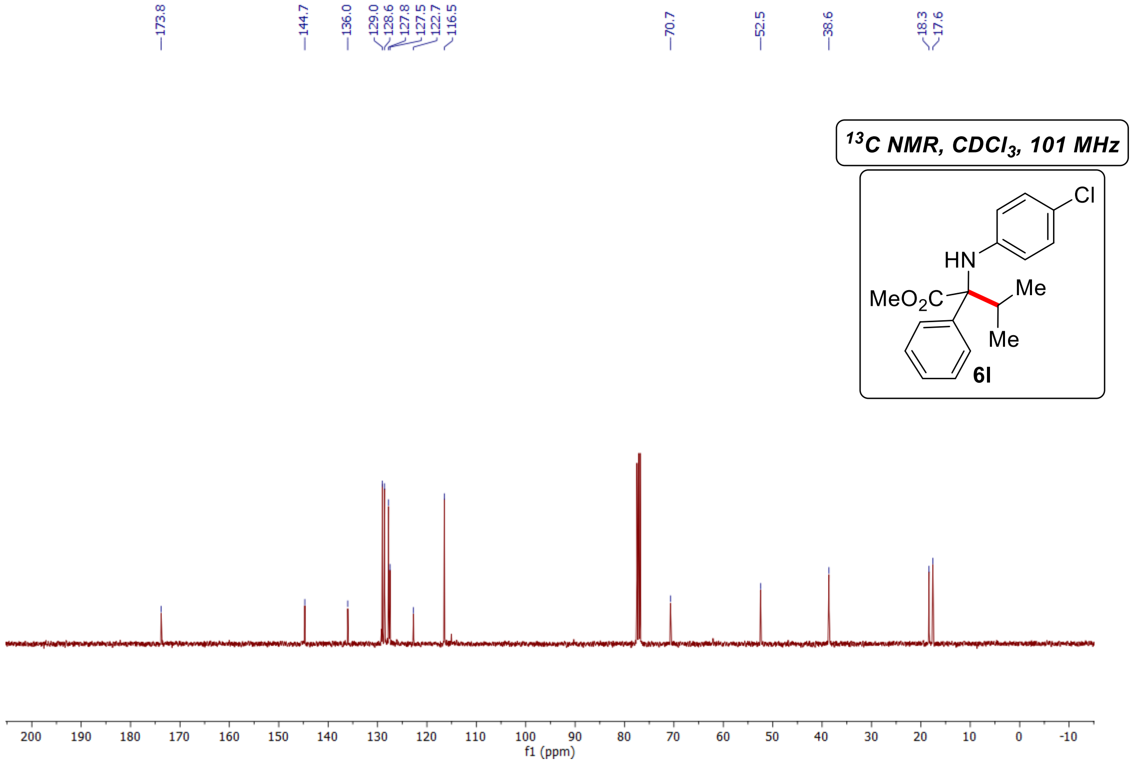

7.40 Methyl 2-((4-bromophenyl)amino)-3-methyl-2-phenylbutanoate (6m)

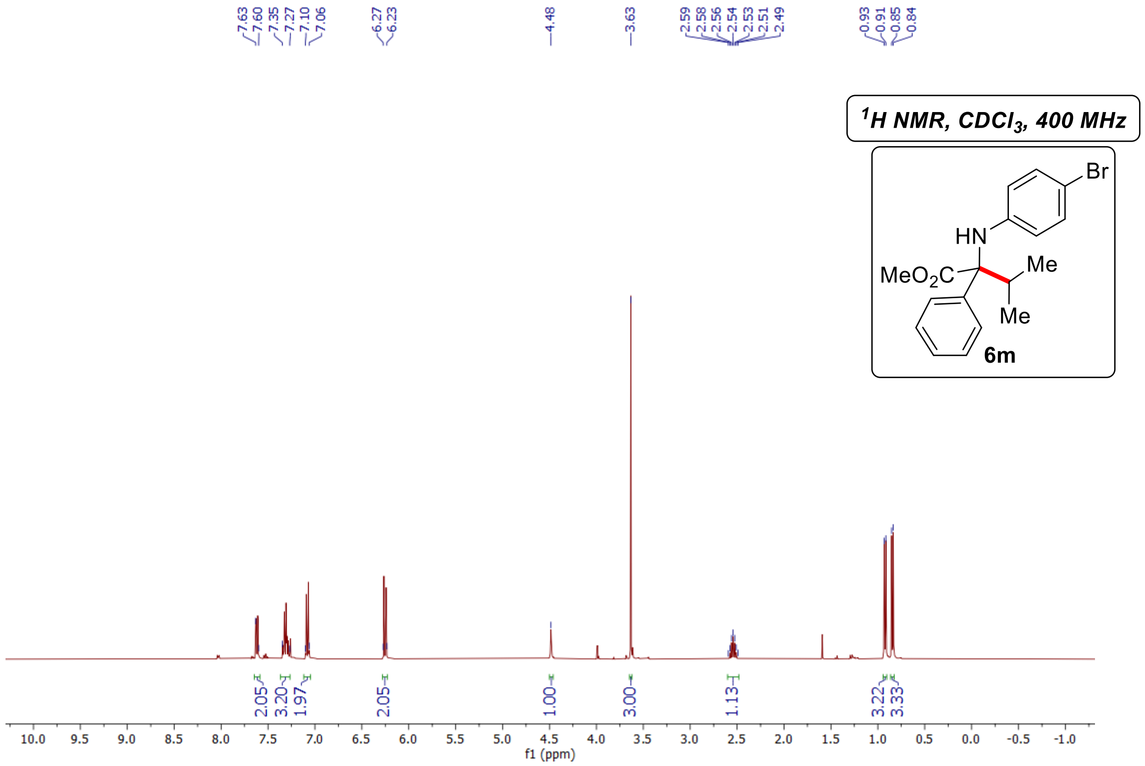

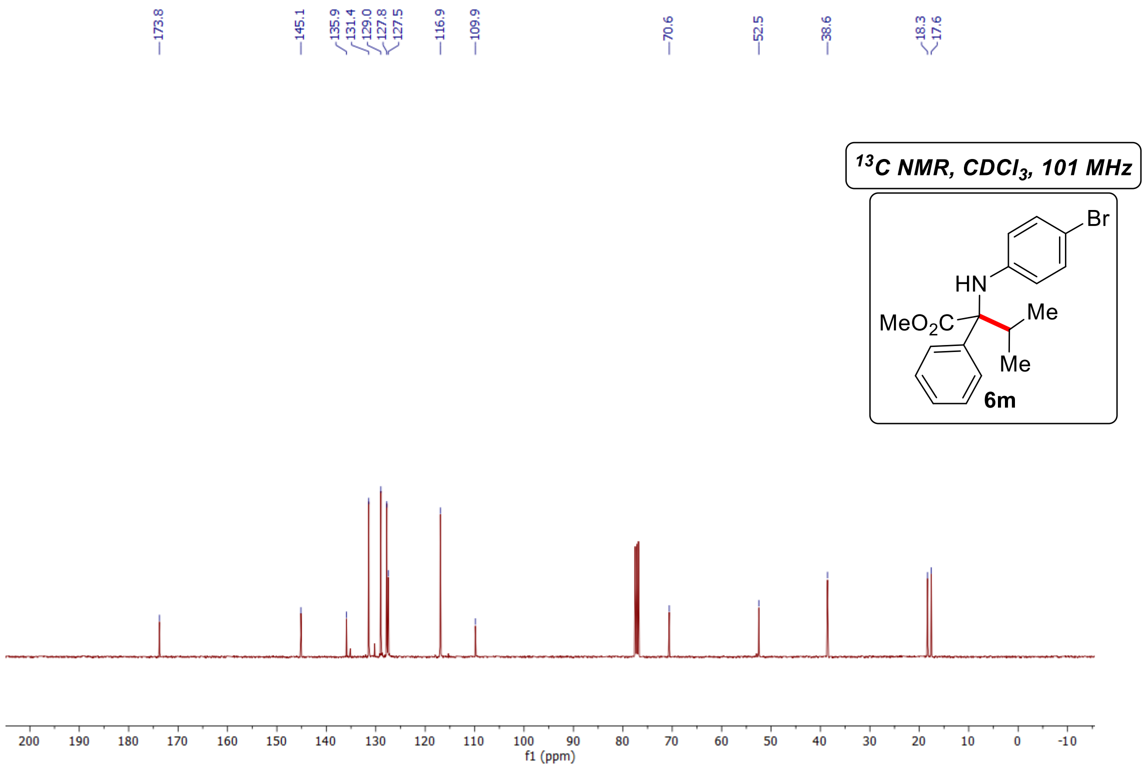

7.41 Methyl-2-((2,6-dimethylphenyl)amino)-3-methyl-2-phenylbutanoate (6n)

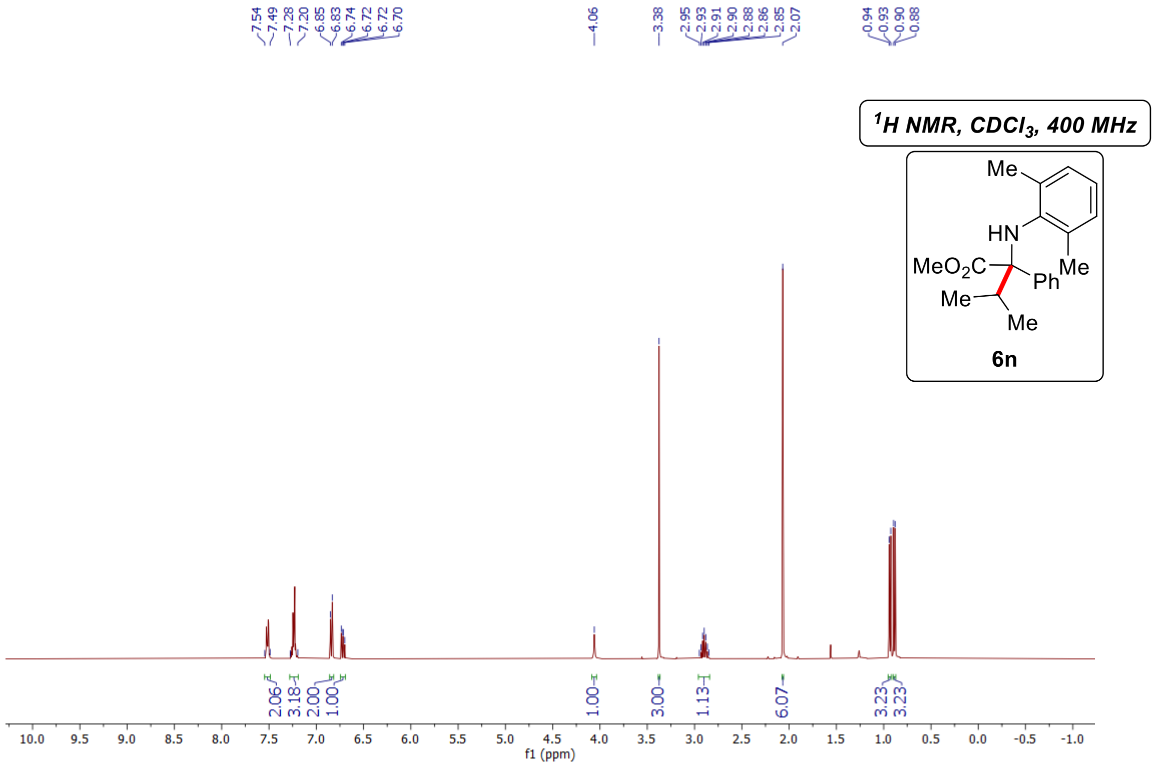

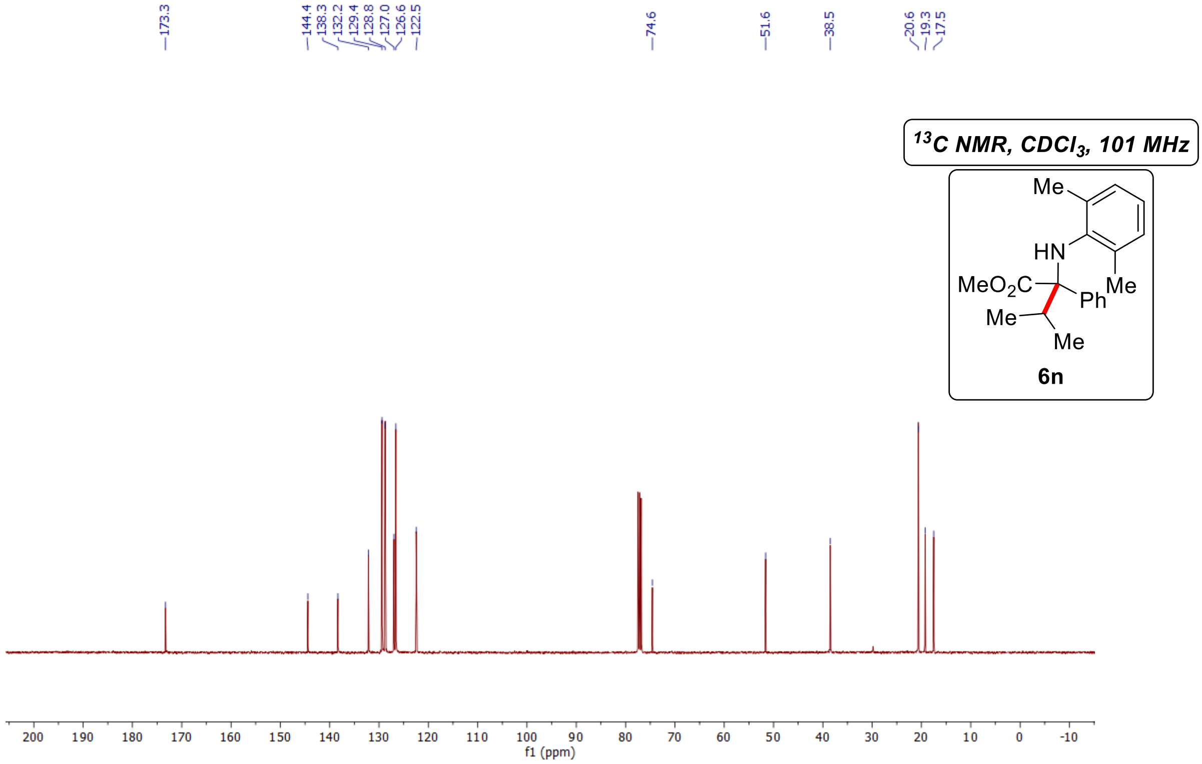

7.42 Methyl 2-((2-ethylphenyl)amino)-3-methyl-2-phenylbutanoate (6o)

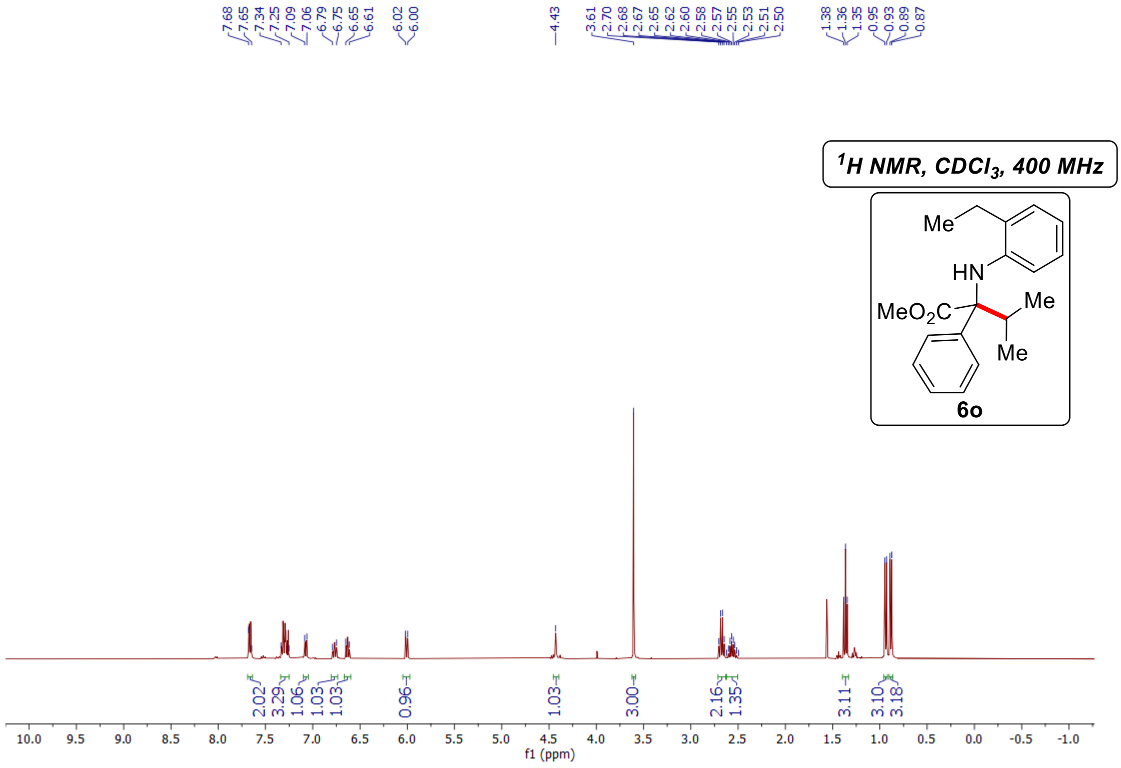

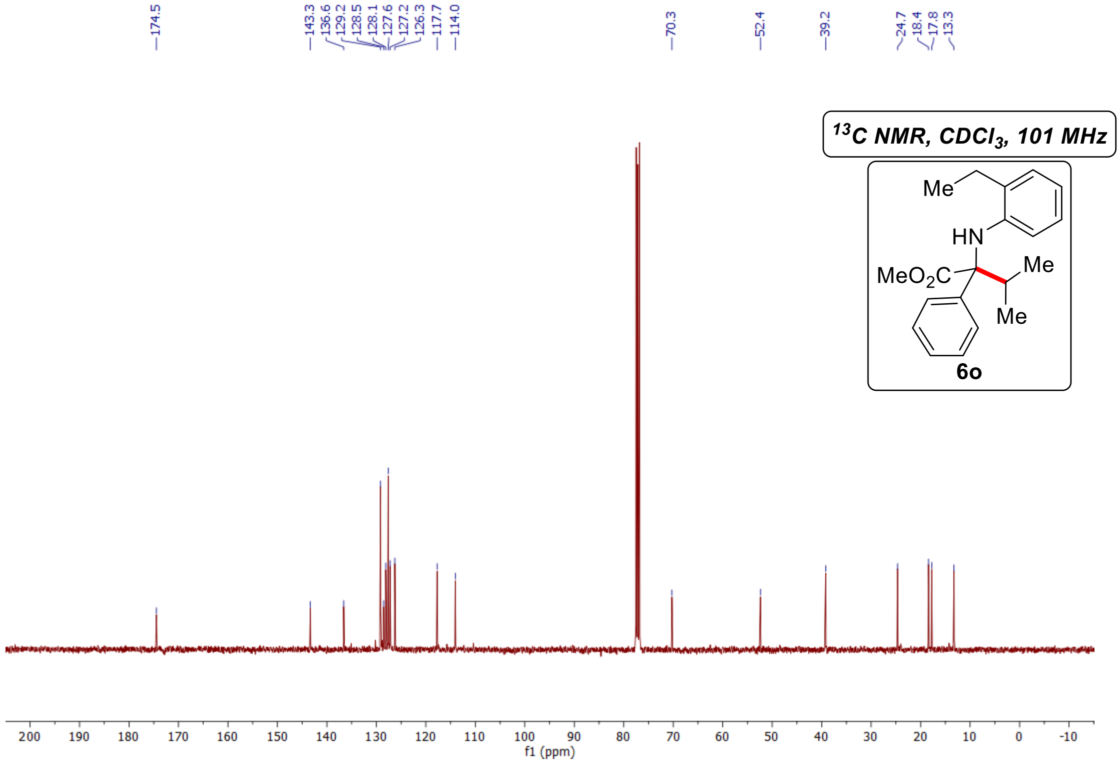

7.43 Methyl 2-((3,5-bis(trifluoromethyl)phenyl)amino)-3-methyl-2-phenylbutanoate (6p)

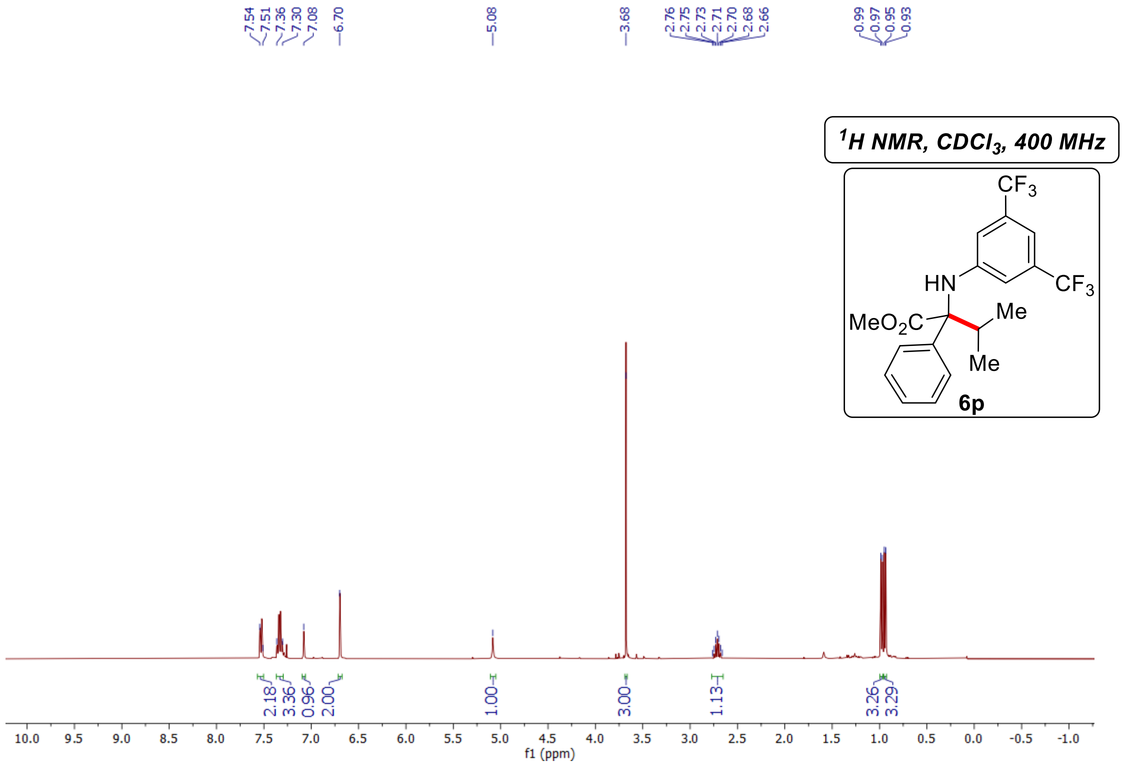

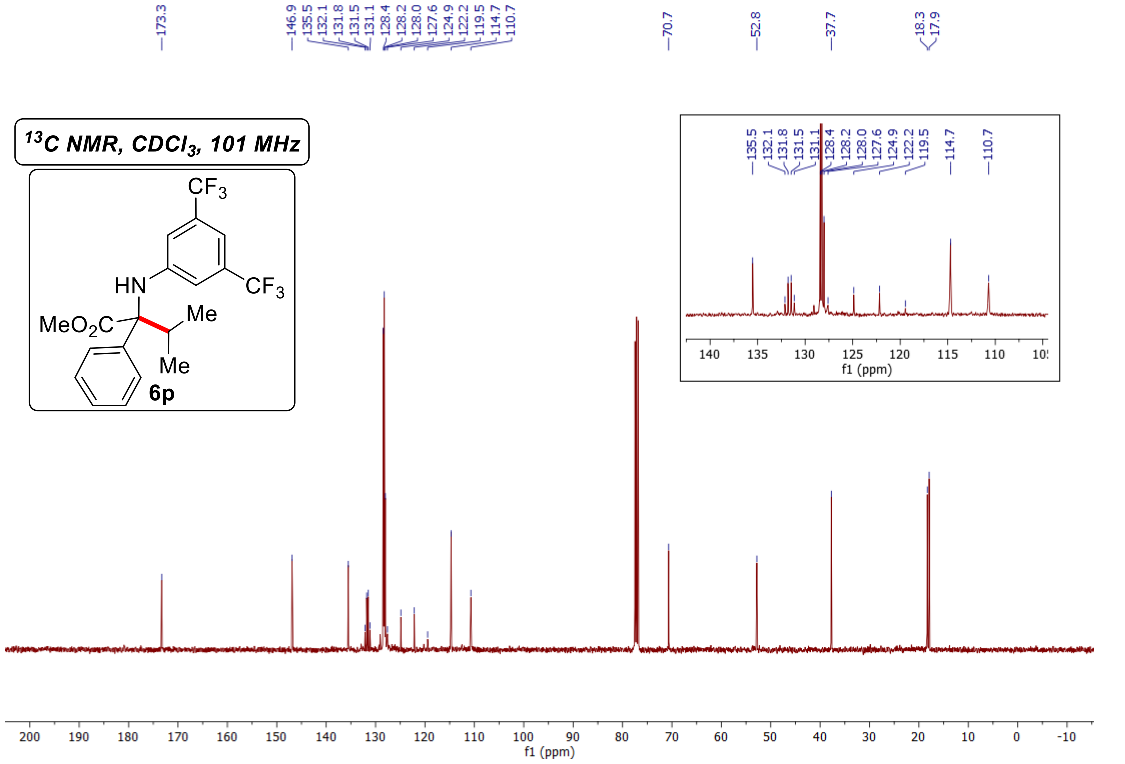

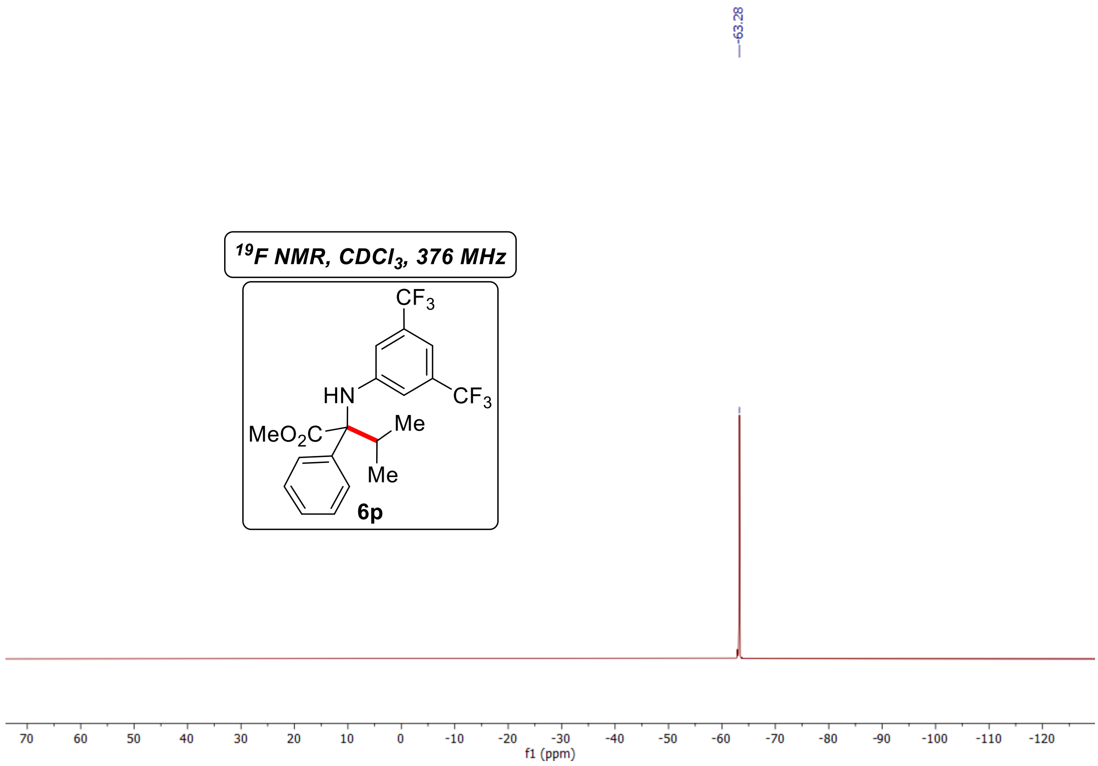

-63.28
^{19}F NMR, CDCl$_3$, 376 MHz
CF$_3$
HN
CF$_3$
MeO$_2$C
Me
Me
6p
70 60 50 40 30 20 10 0 -10 -20 -30 -40 -50 -60 -70 -80 -90 -100 -110 -120
f1 (ppm)

7.44 Methyl 2-cyclohexyl-2-phenyl-2-(phenylamino)acetate (6q)

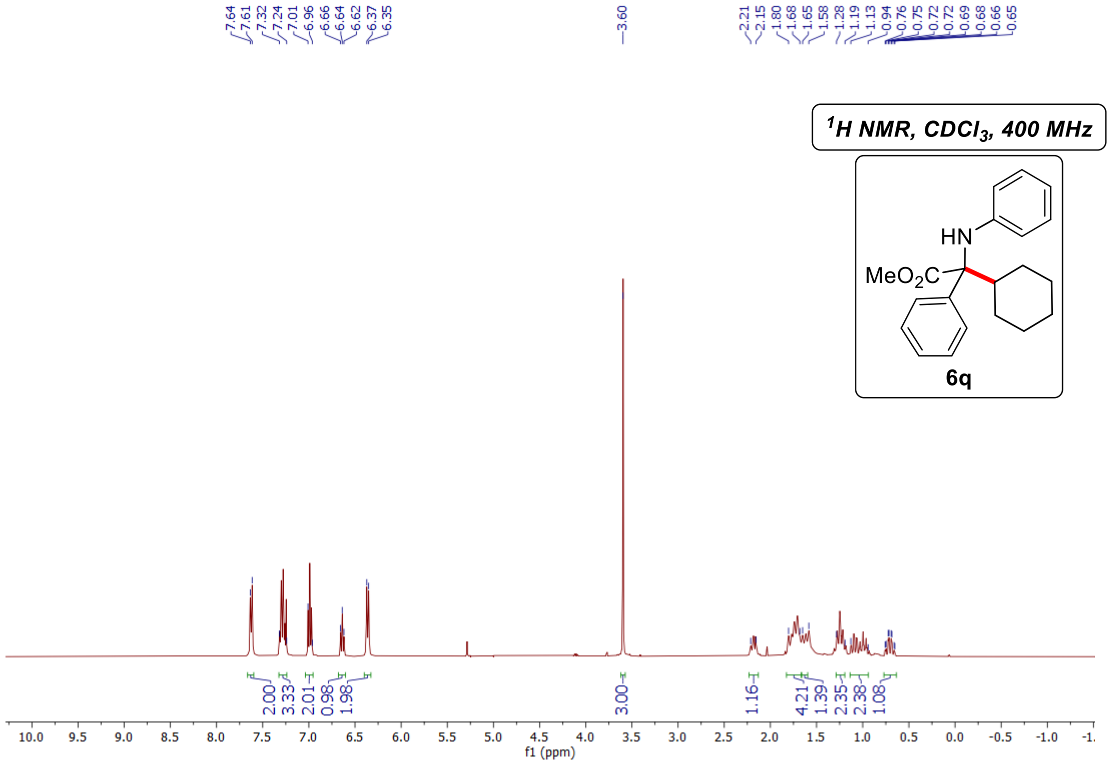

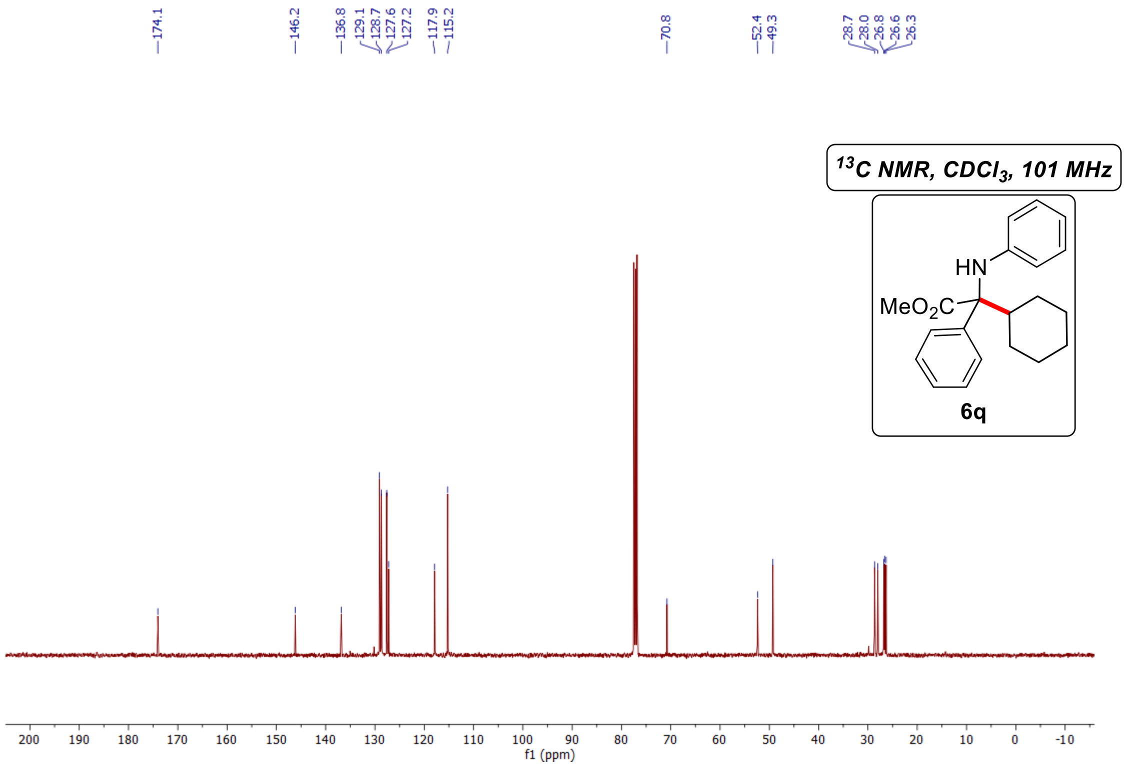

7.45 Methyl 2-cyclobutyl-2-phenyl-2-(phenylamino)acetate (**6r**)

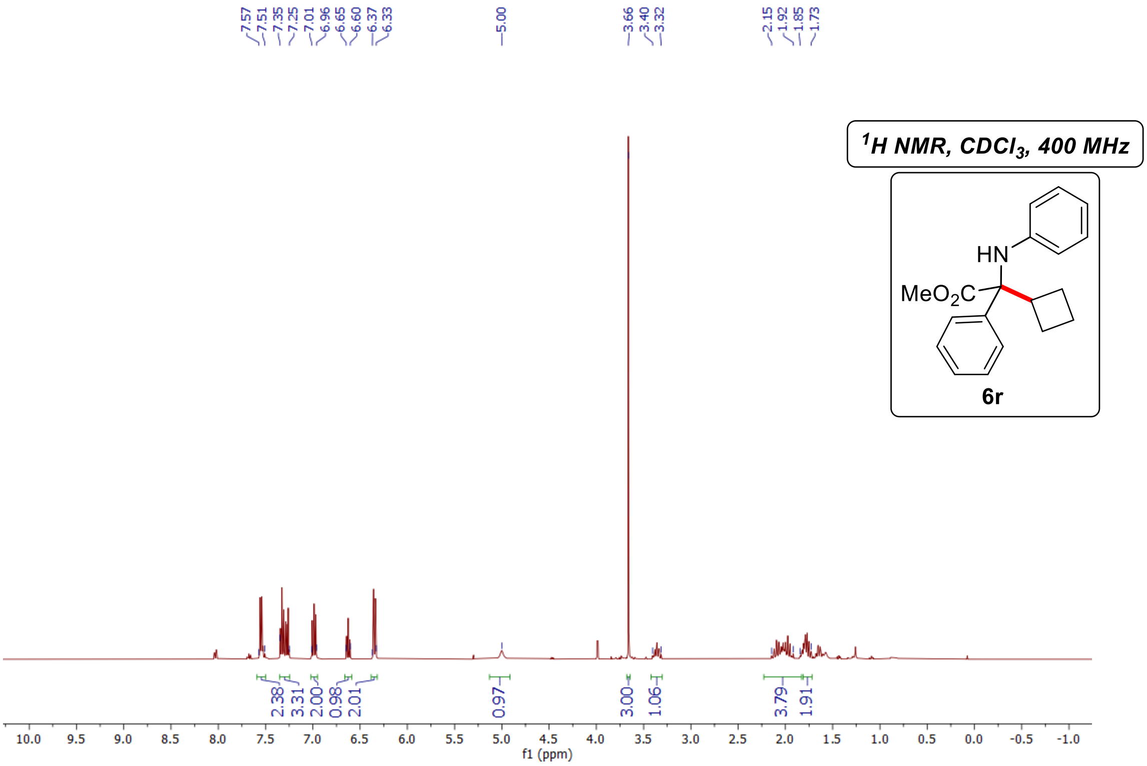

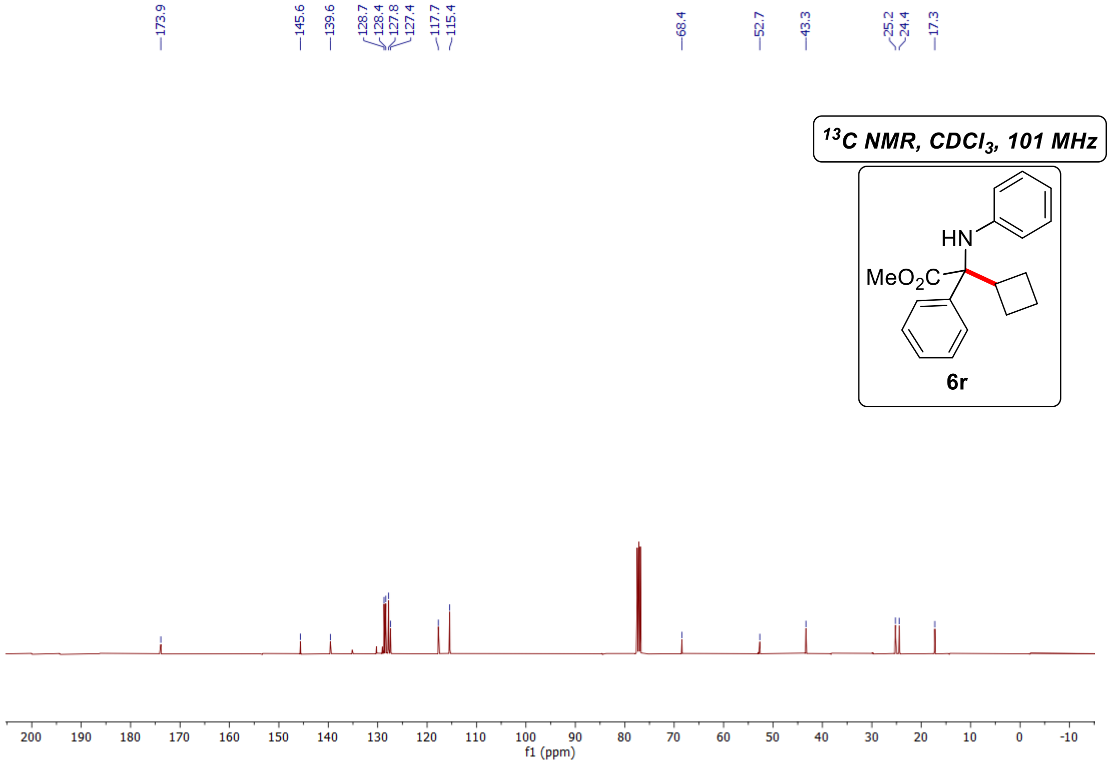

7.46 Methyl (3*R*)-3,7-dimethyl-2-phenyl-2-(phenylamino)oct-6-enoate (6s)

Isolated as 3:2 diastereomeric mixture

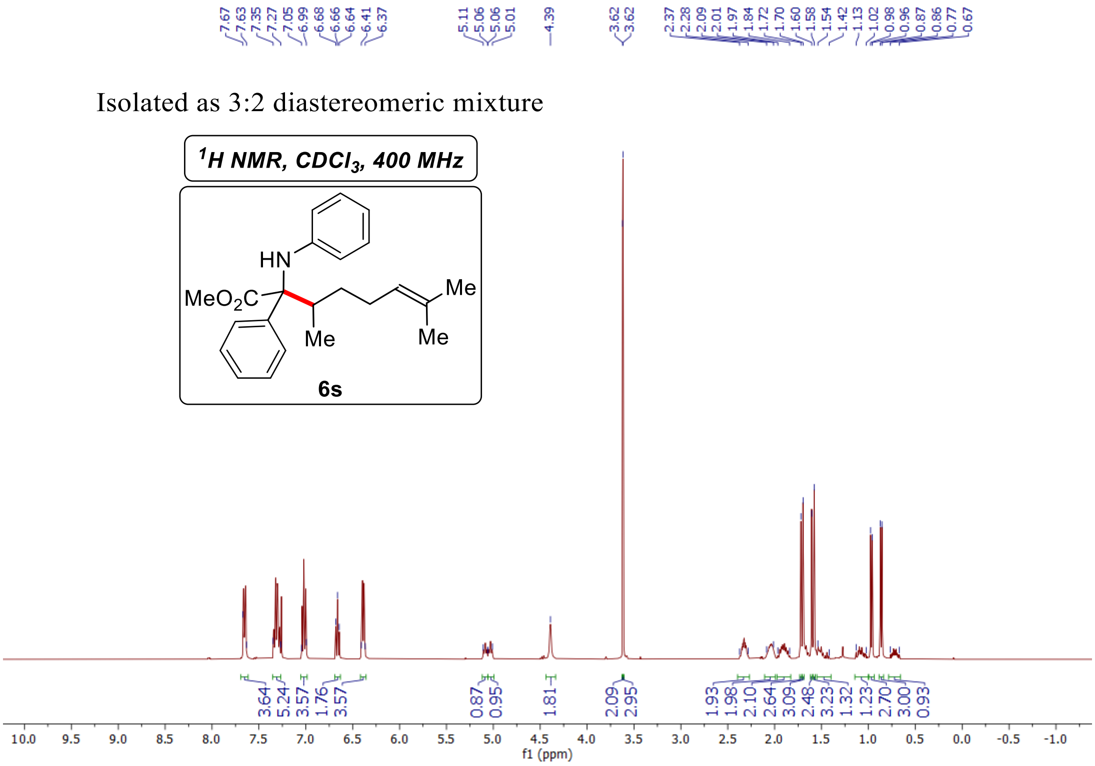

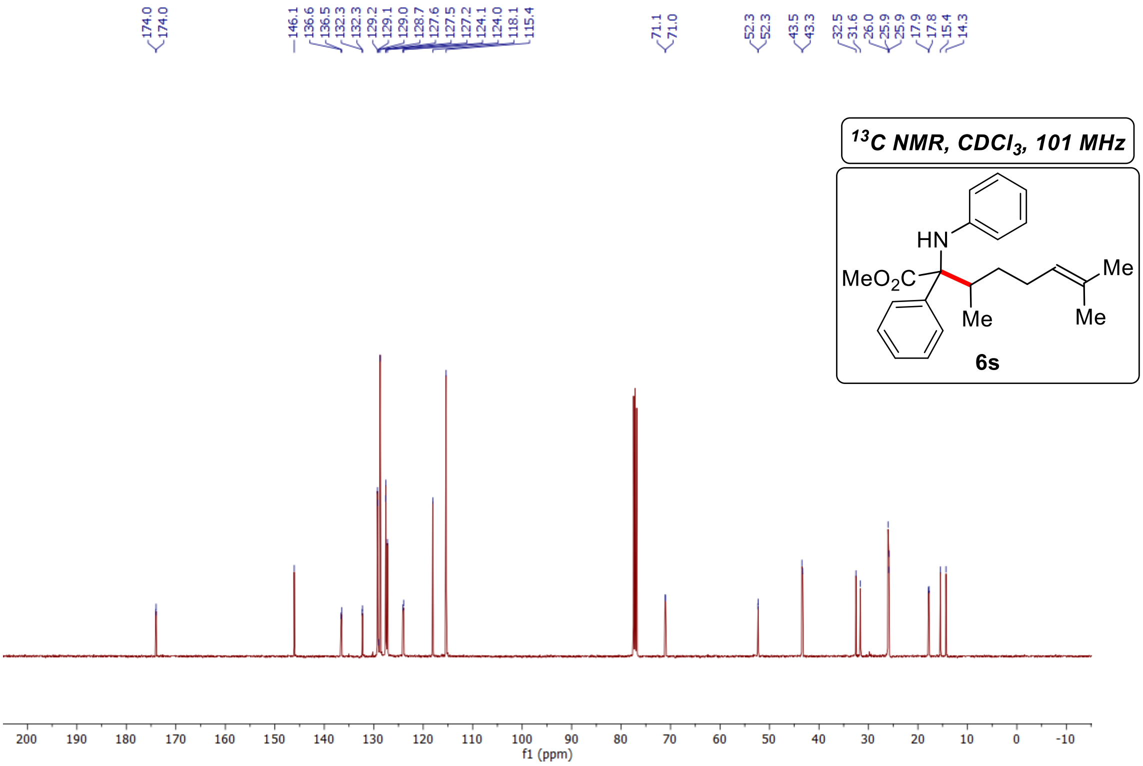

7.47 Methyl (3*R*)-4-(4-(tert-butyl)phenyl)-3-methyl-2-phenyl-2-(phenylamino)butanoate (6t)

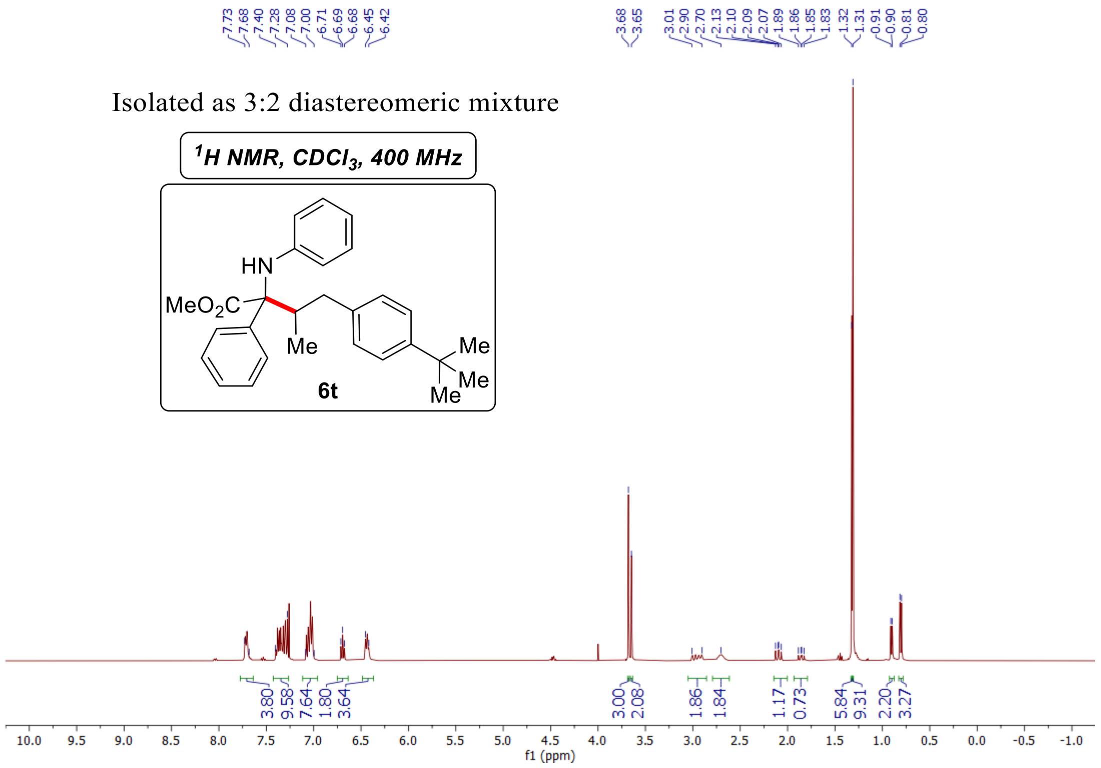

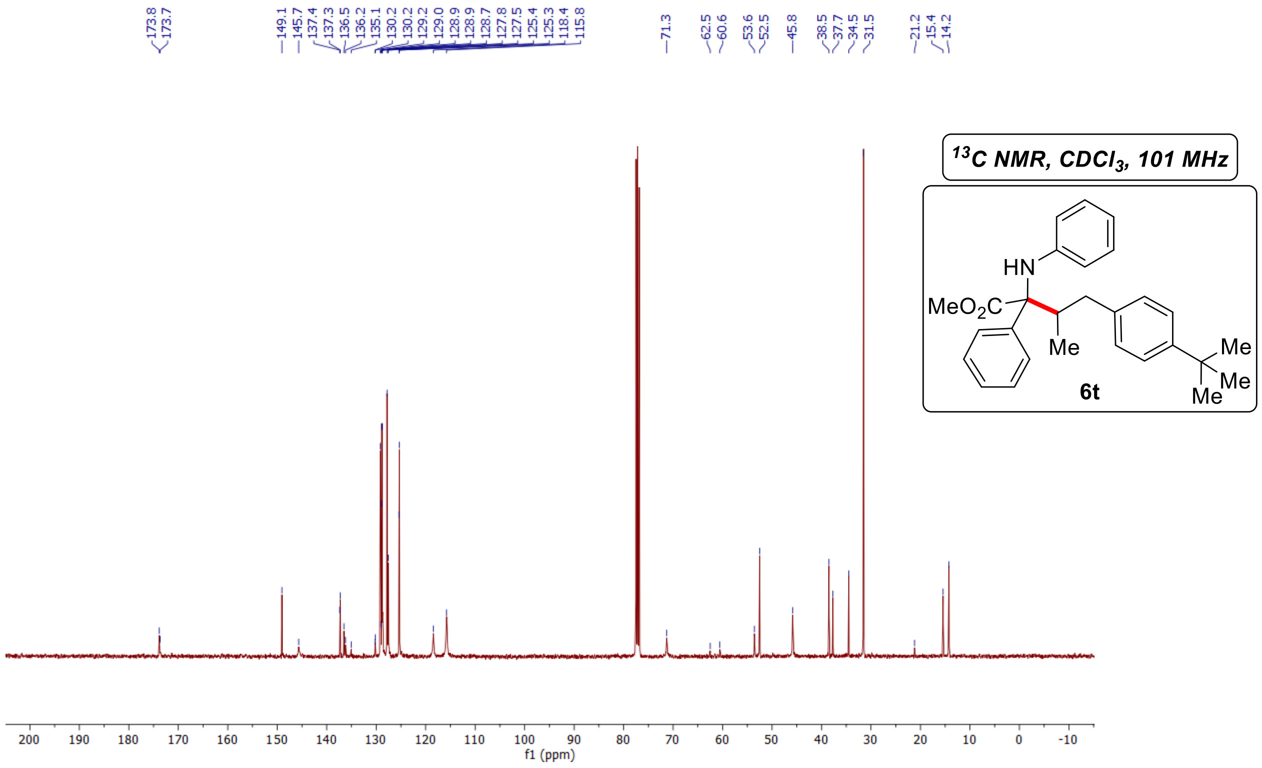

7.48 Methyl 3-ethyl-2-phenyl-2-(phenylamino)pentanoate (6u)

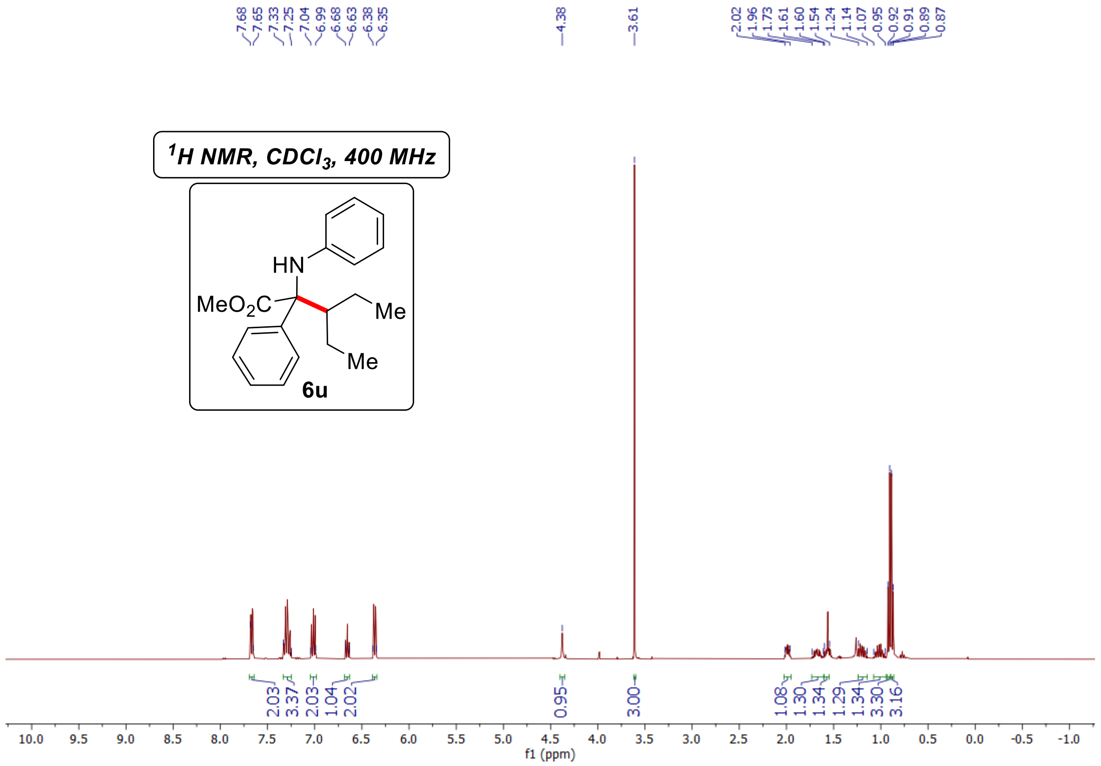

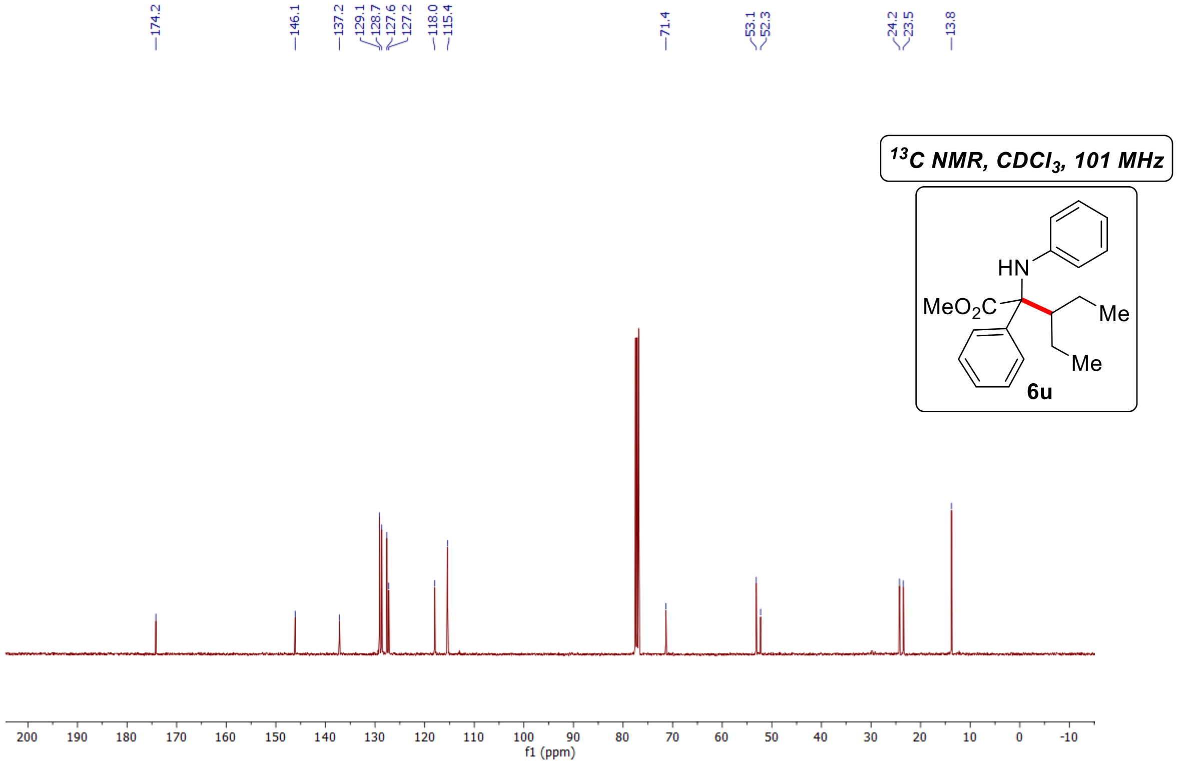

7.49 Methyl 4-methyl-2-phenyl-2-(phenylamino)pentanoate (6v)

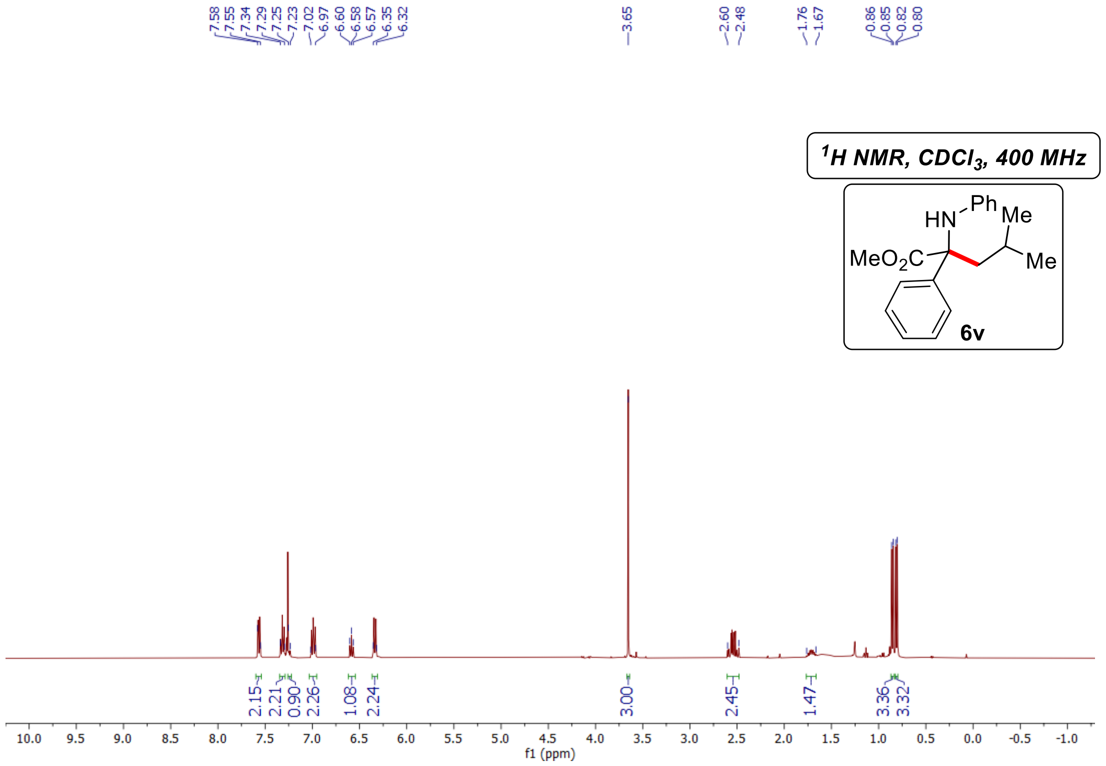

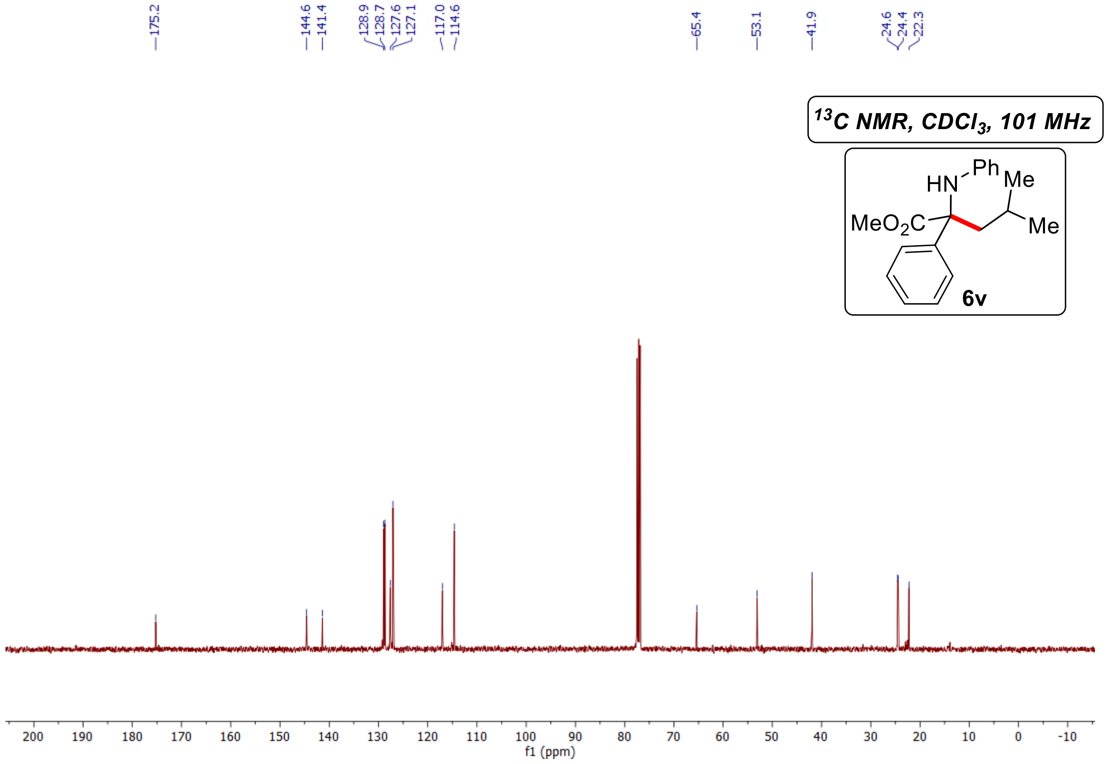

7.50 Methyl 2,4-diphenyl-2-(phenylamino)butanoate (6w)

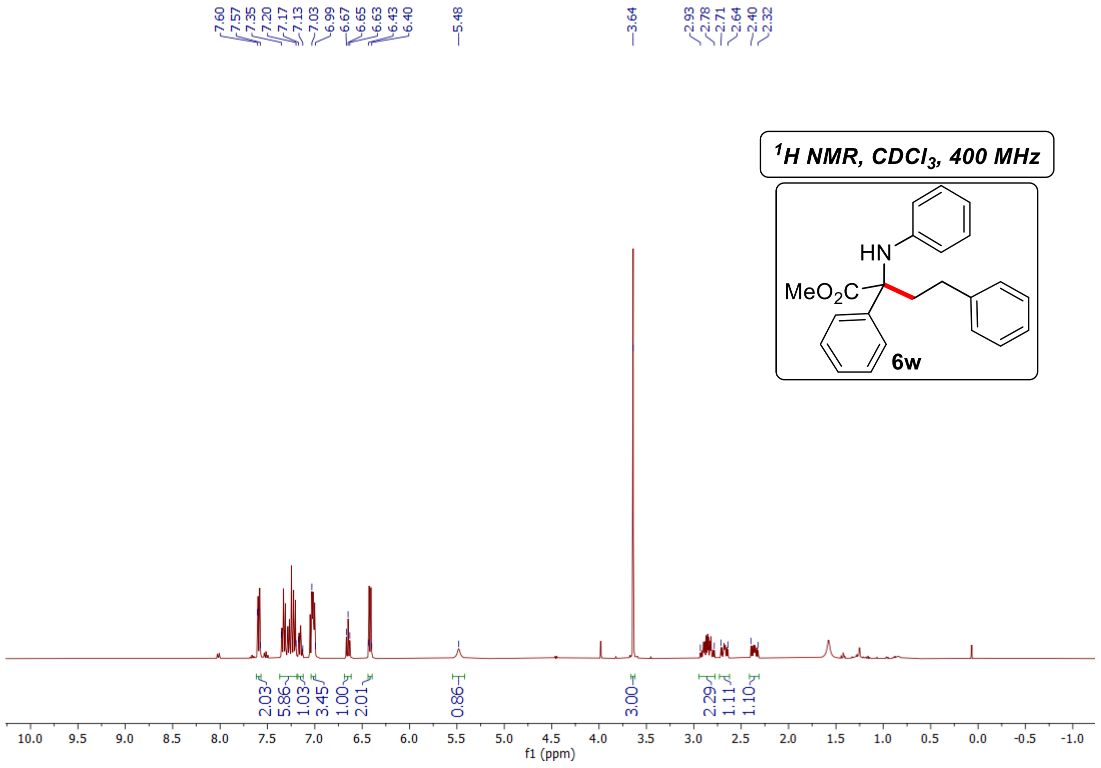

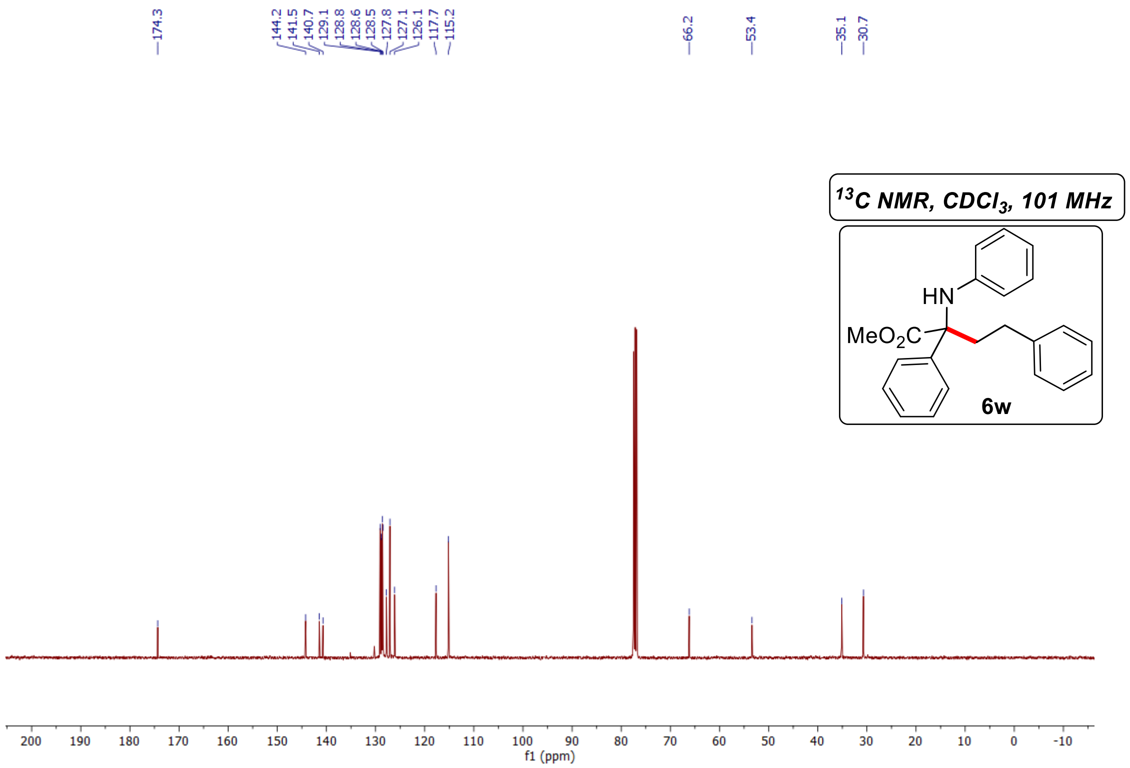

7.51 Methyl 2-phenyl-2-(phenylamino)pentanoate (6x)

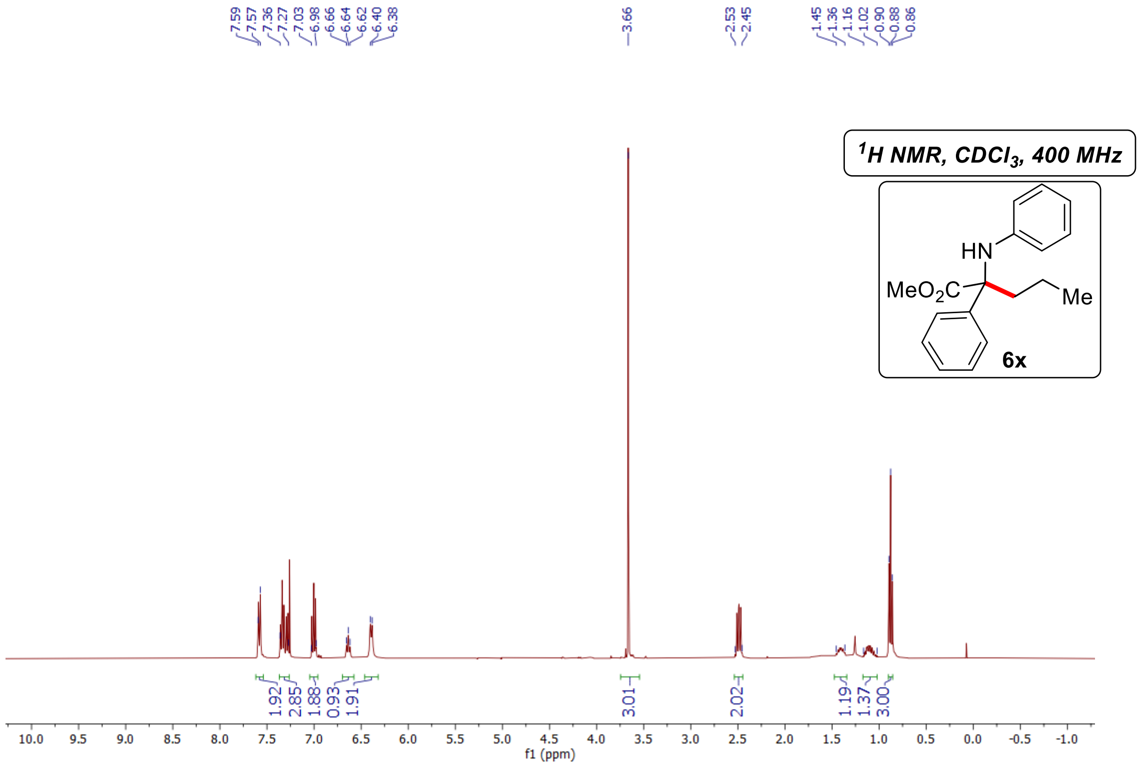

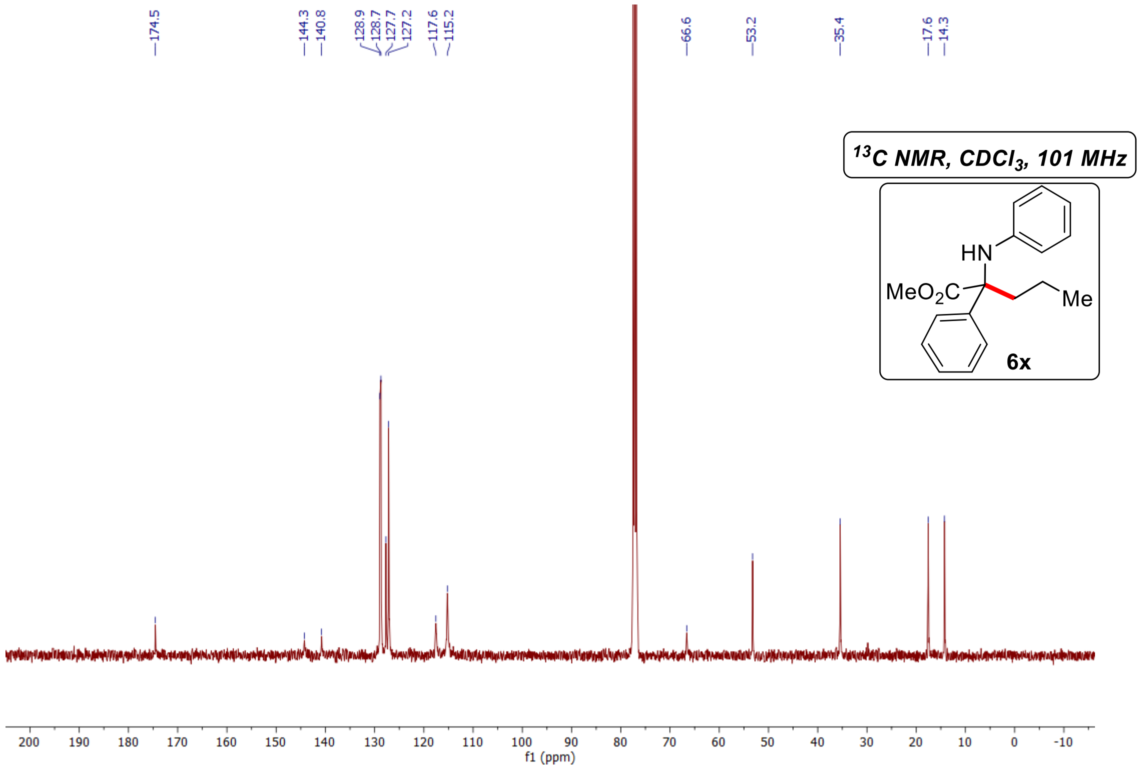

7.52 Methyl 2-phenyl-2-(phenylamino)hexanoate (6y)

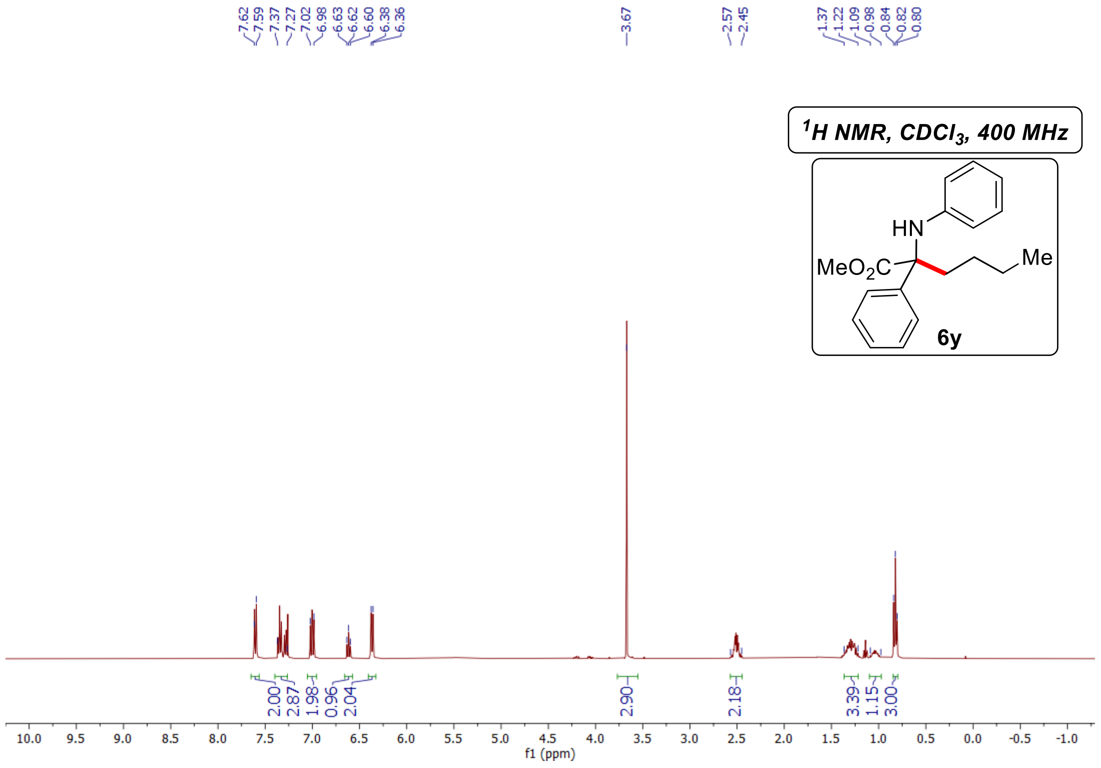

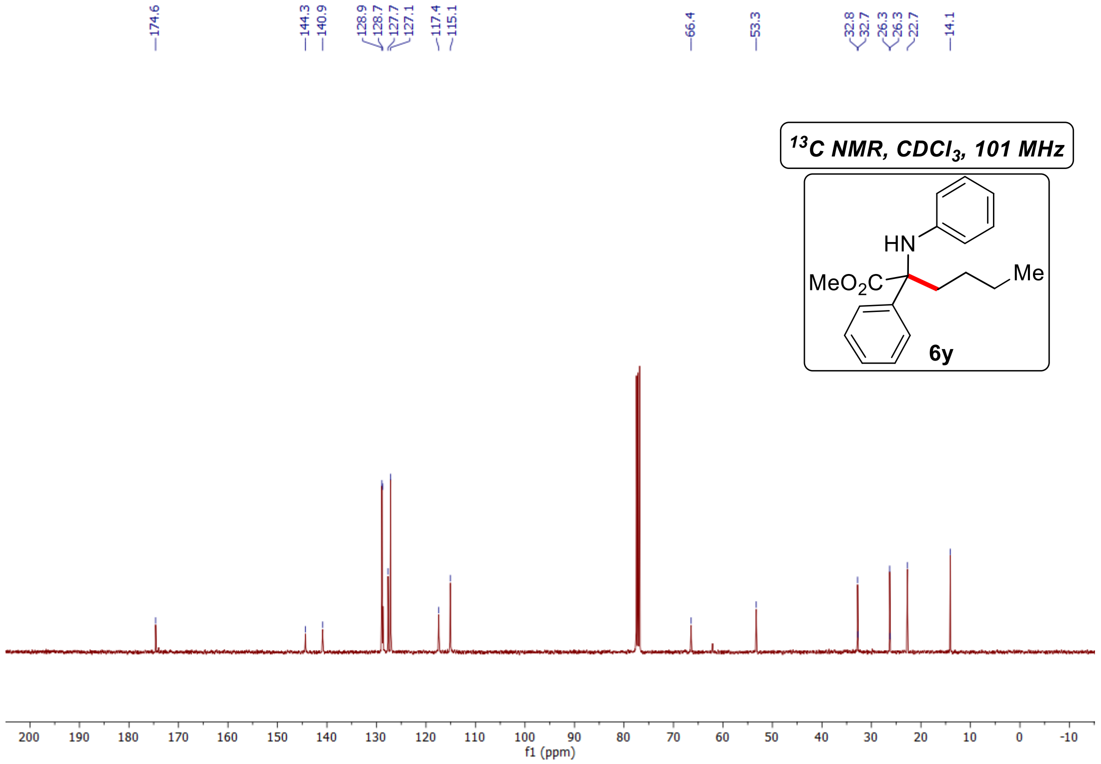

7.53 Methyl 3-(benzyloxy)-2-phenyl-2-(phenylamino)propanoate (6z)

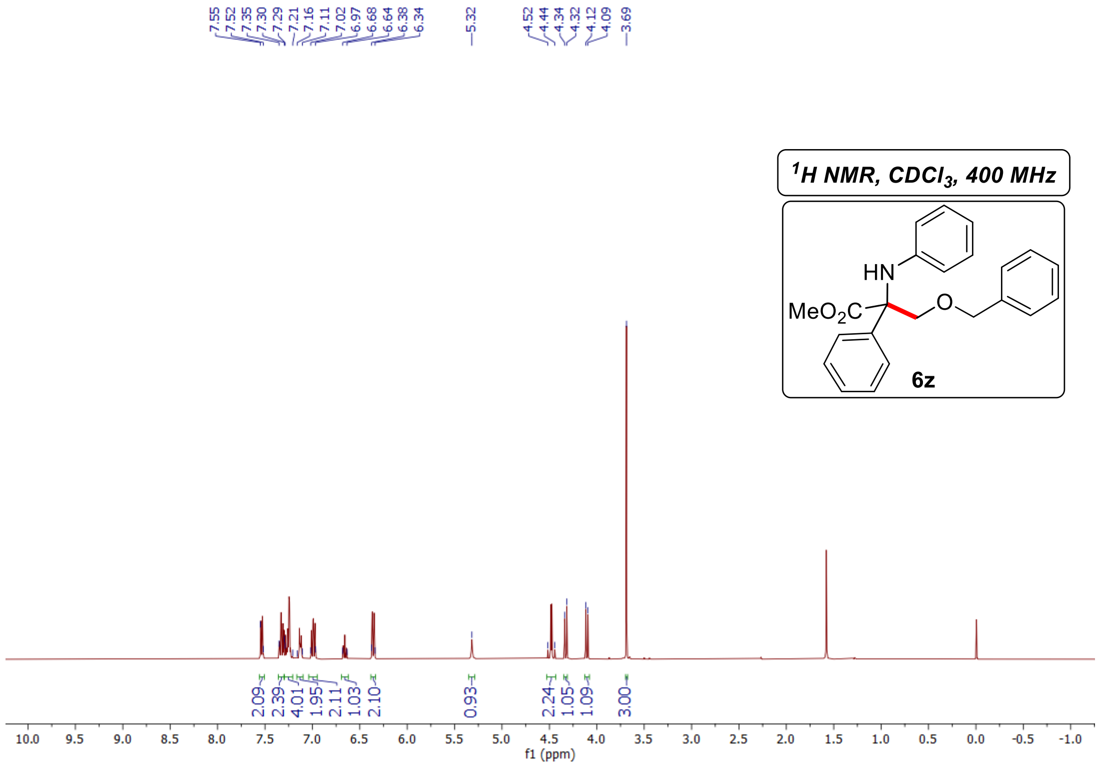

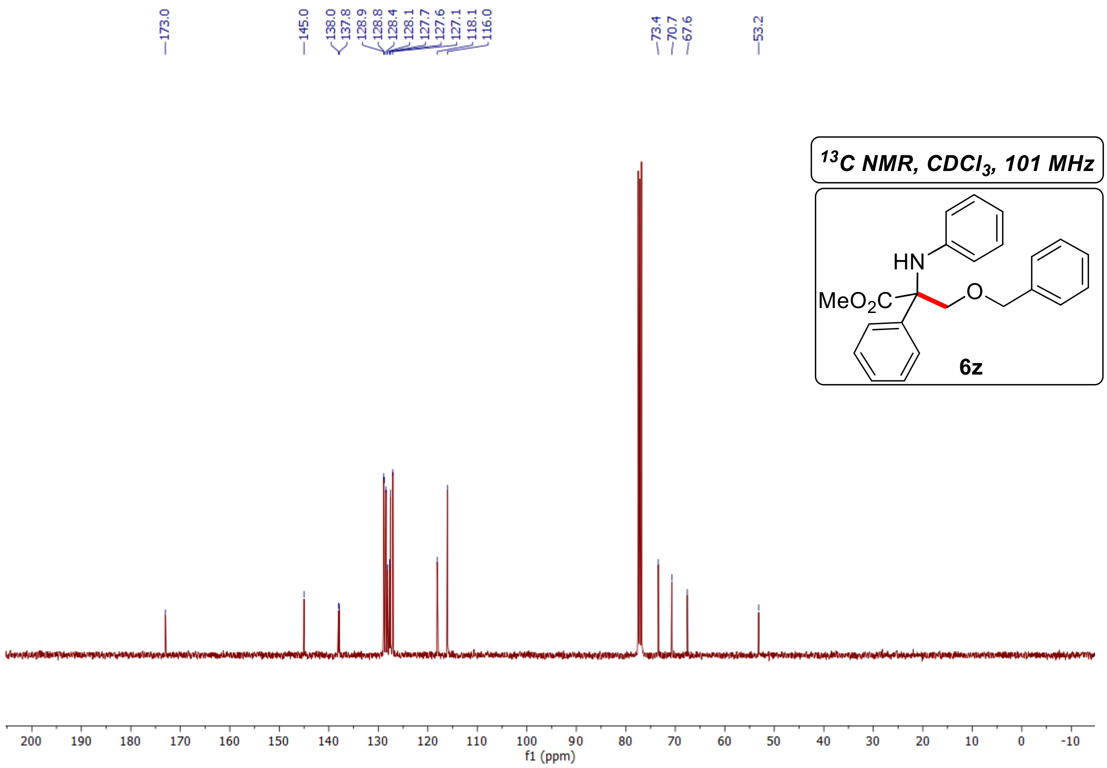

7.54 Methyl 2,3-diphenyl-2-(phenylamino)propanoate (6aa)

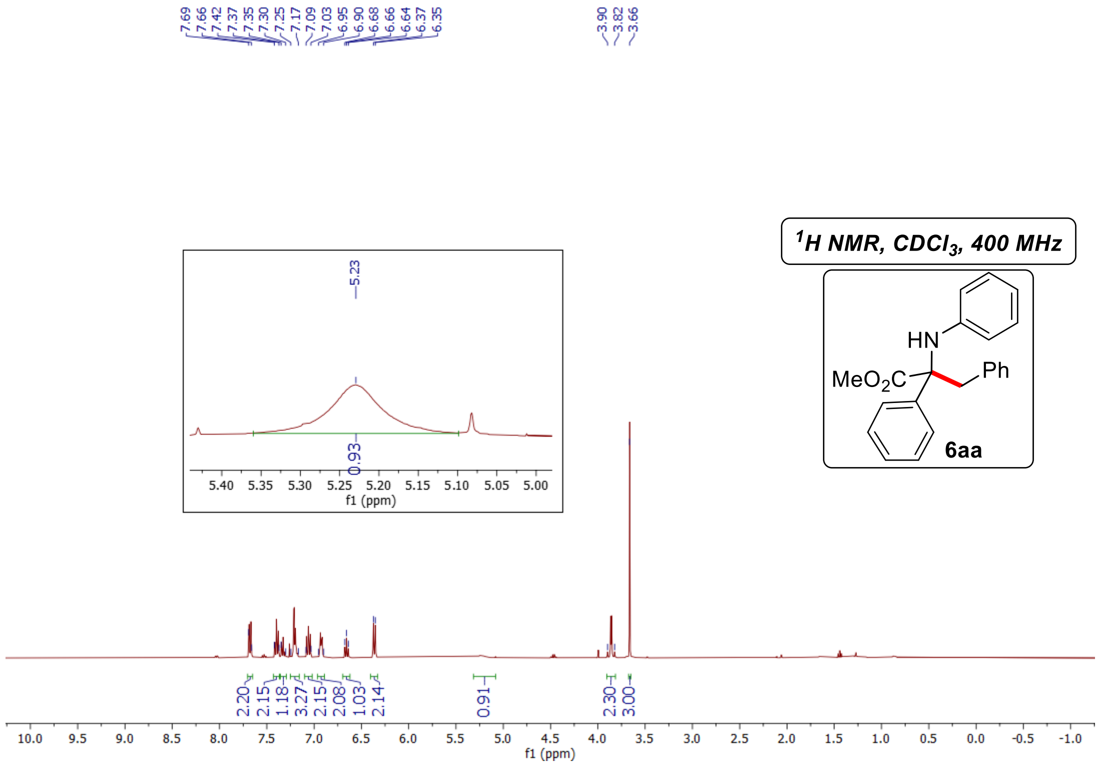

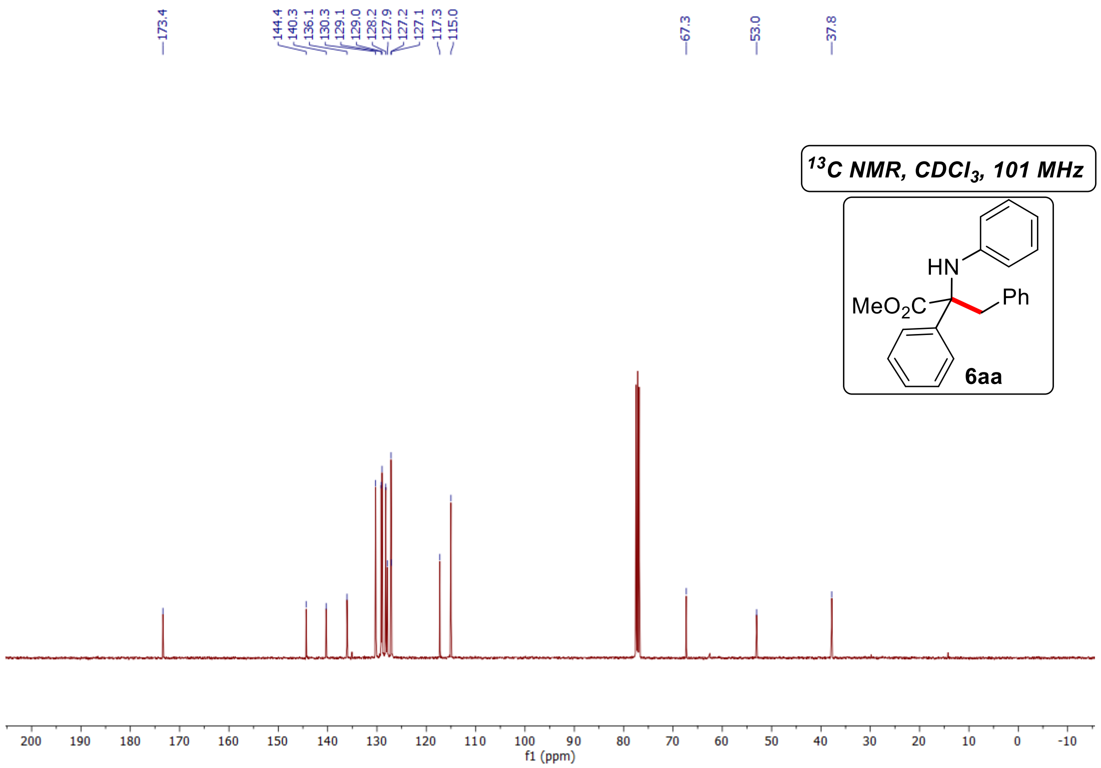

7.55 Methyl 3,3-dimethyl-2-phenyl-2-(phenylamino)butanoate (6ab)

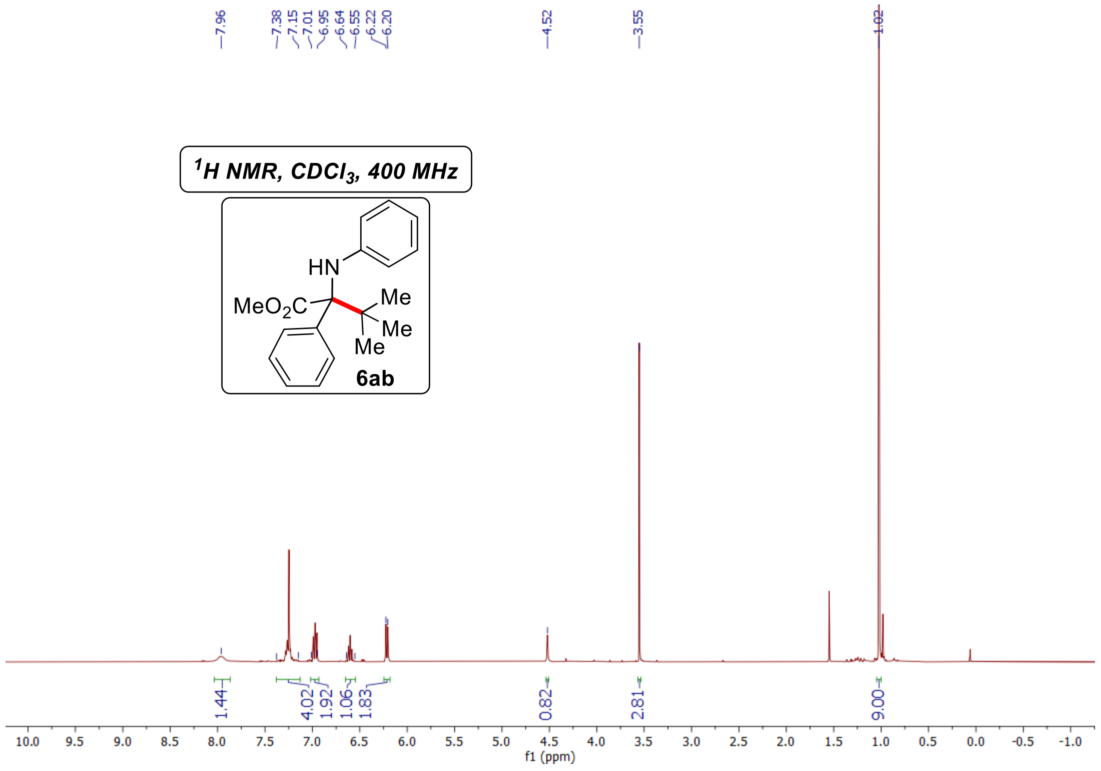

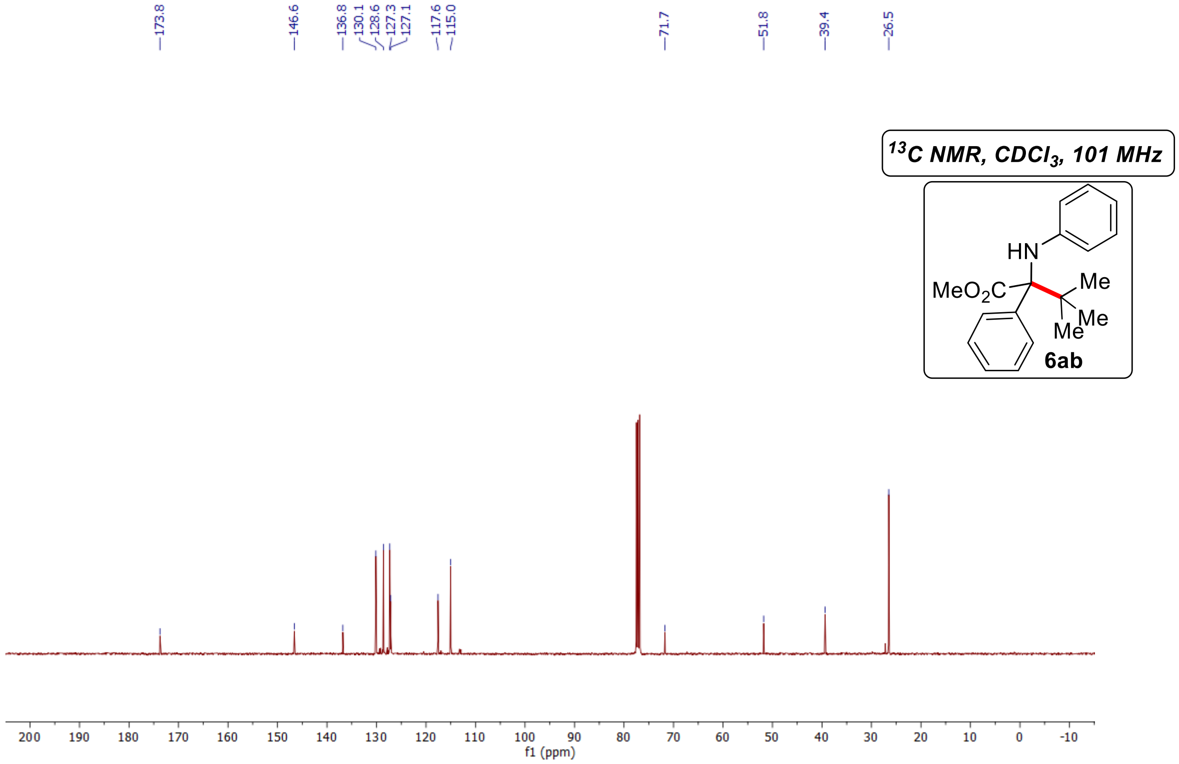

7.56 Methyl 3,3-dimethyl-2,4-diphenyl-2-(phenylamino)butanoate (6ac)

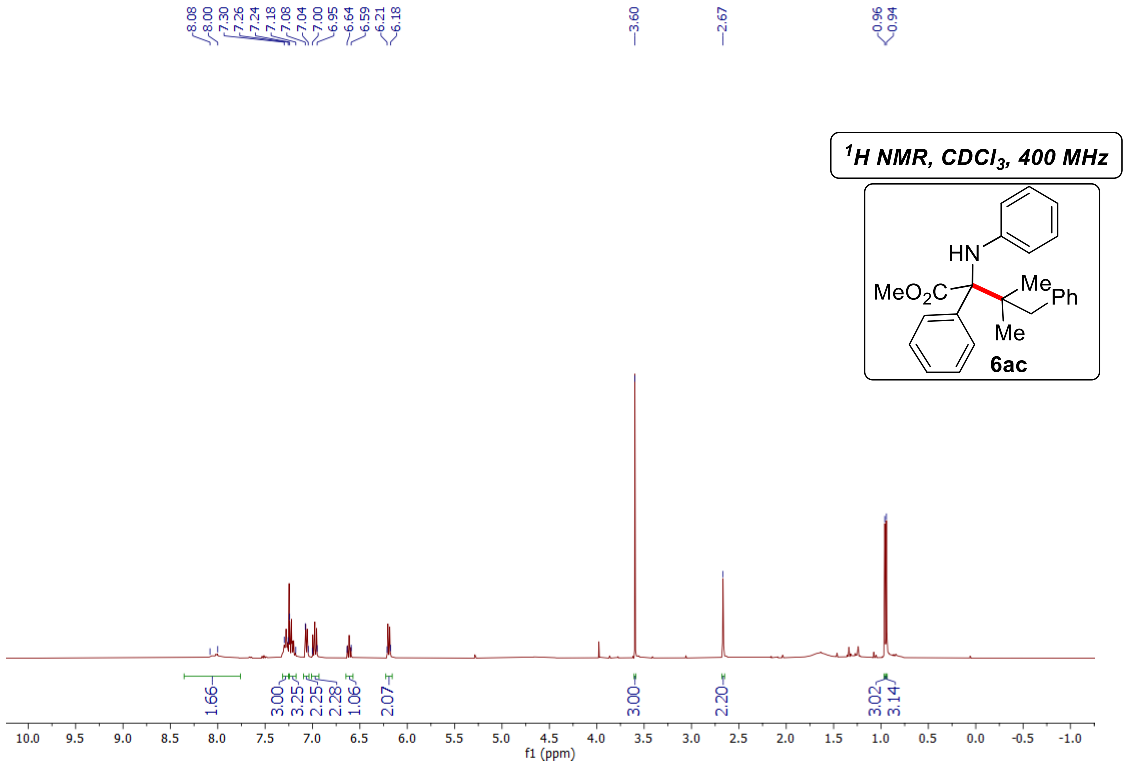

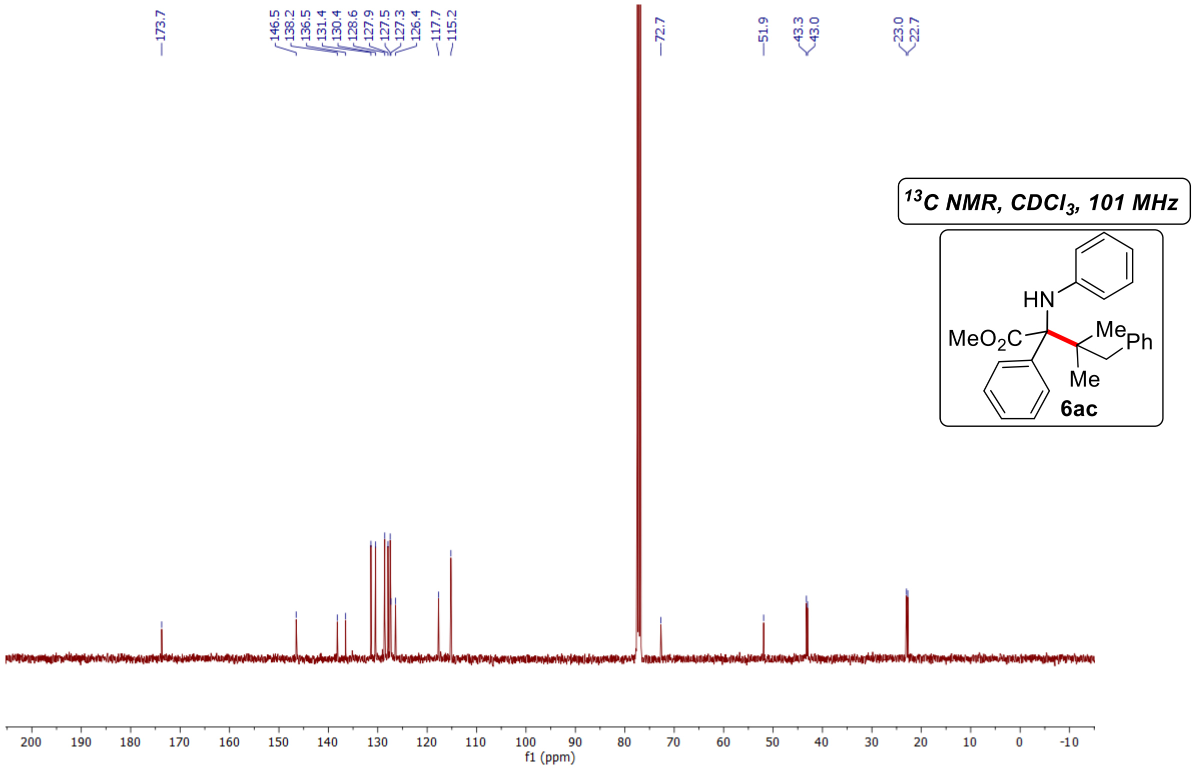

7.57. Methyl 4-cyano-2-phenyl-2-(phenylamino)butanoate (8a)

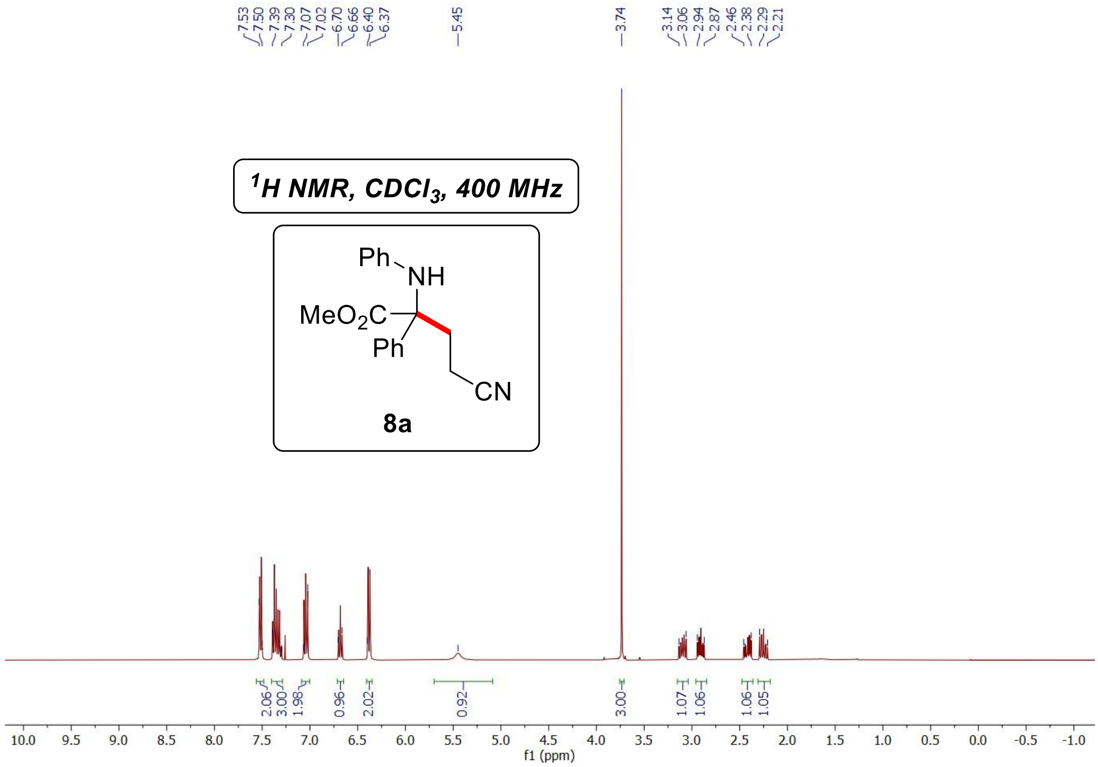

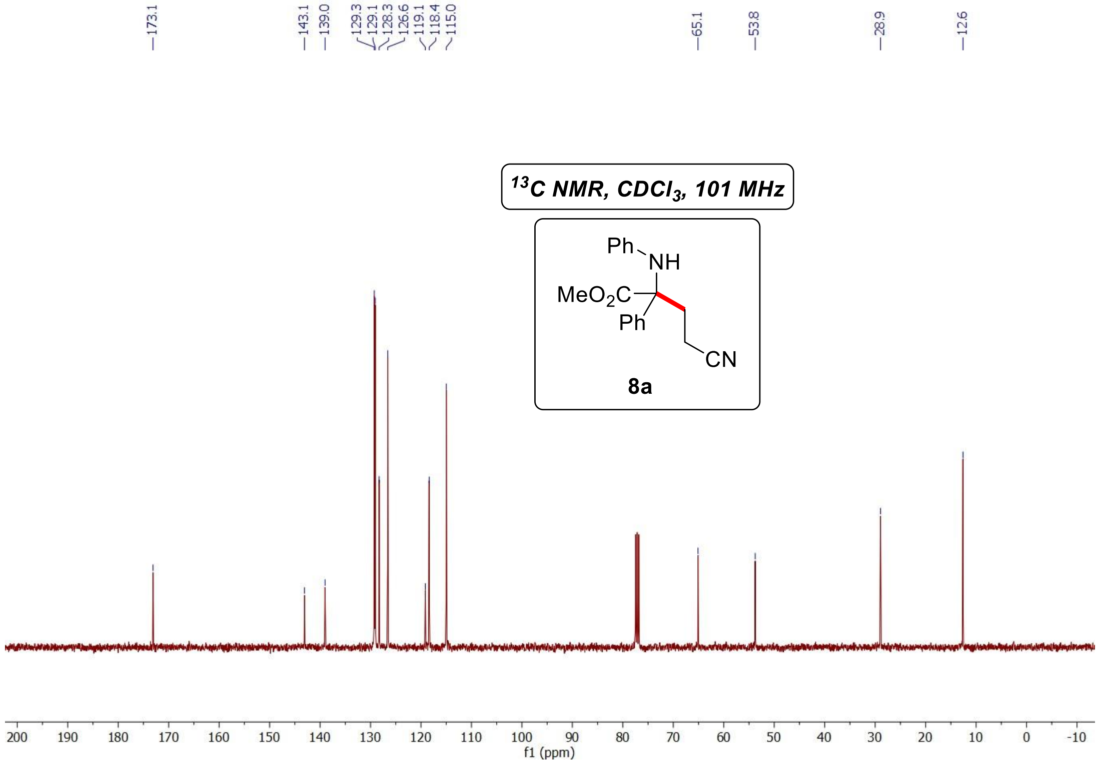

7.58. 5-(tert-butyl) 1-methyl 2-phenyl-2-(phenylamino)pentanedioate (8b)

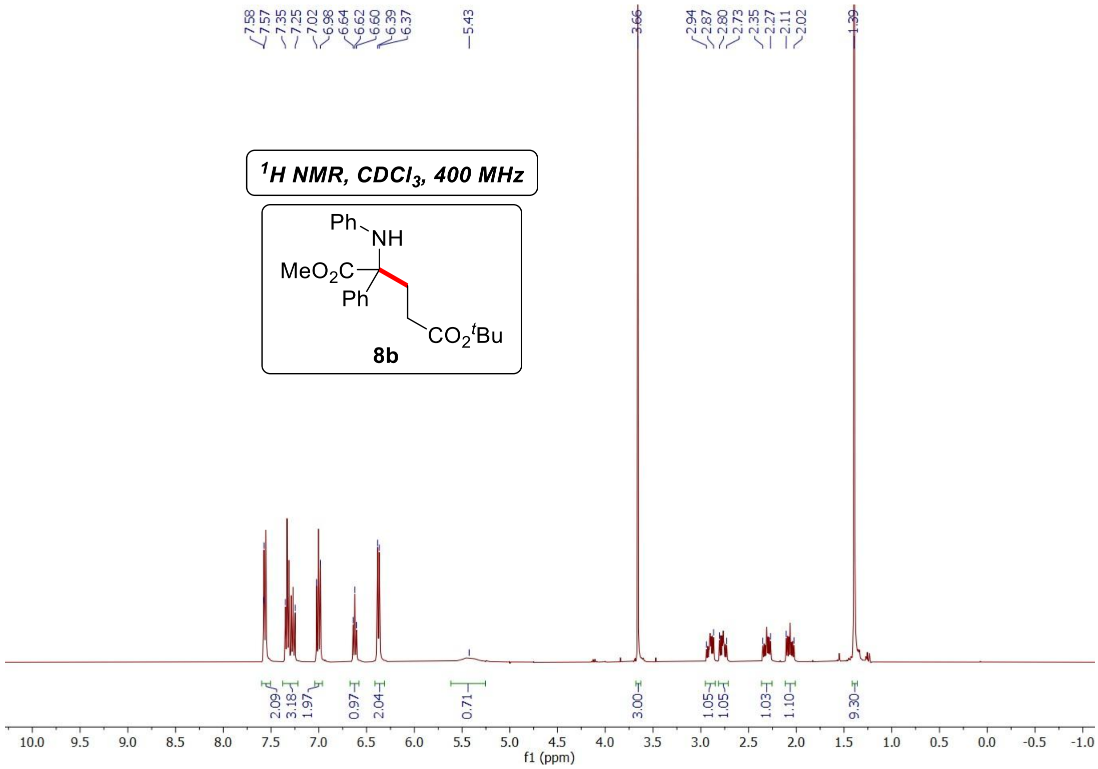

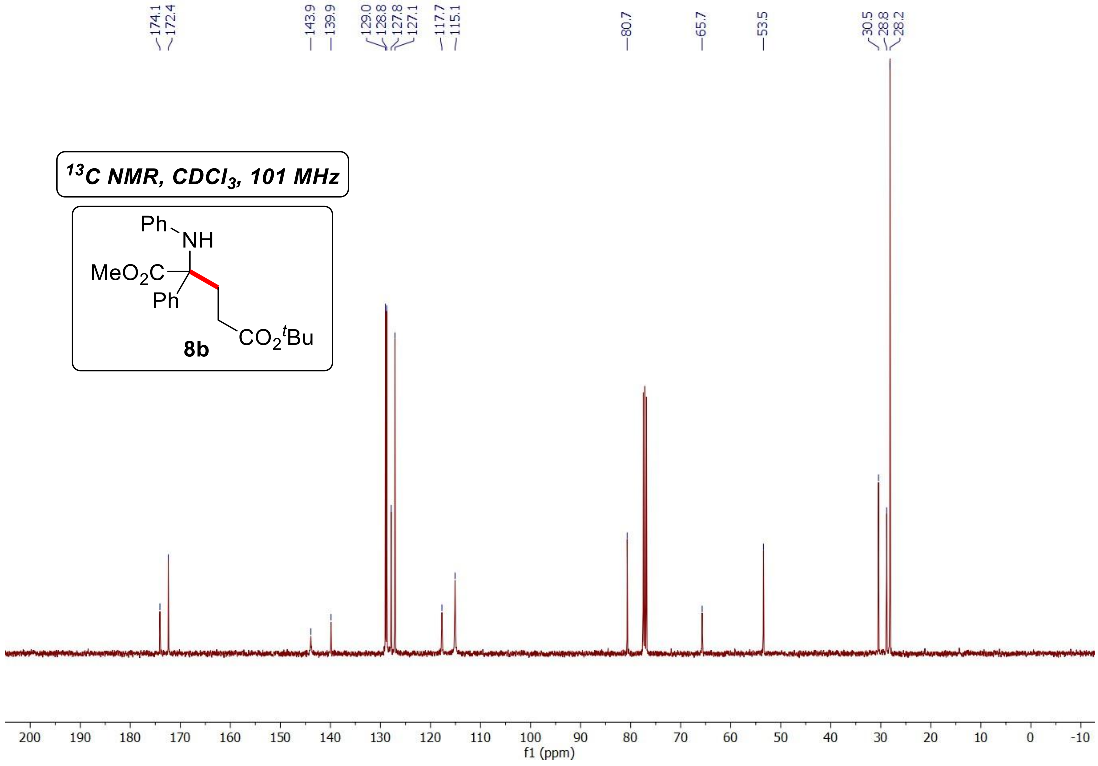

7.59. 5-benzyl 1-methyl 2-phenyl-2-(phenylamino)pentanedioate (8c)

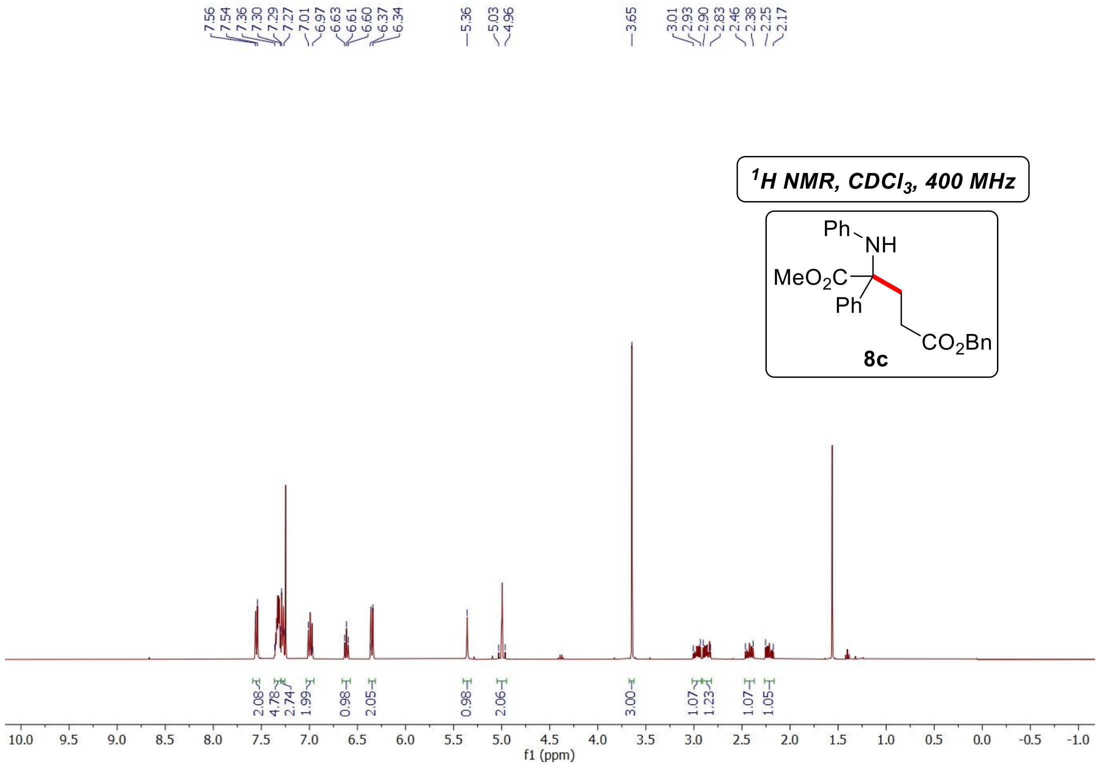

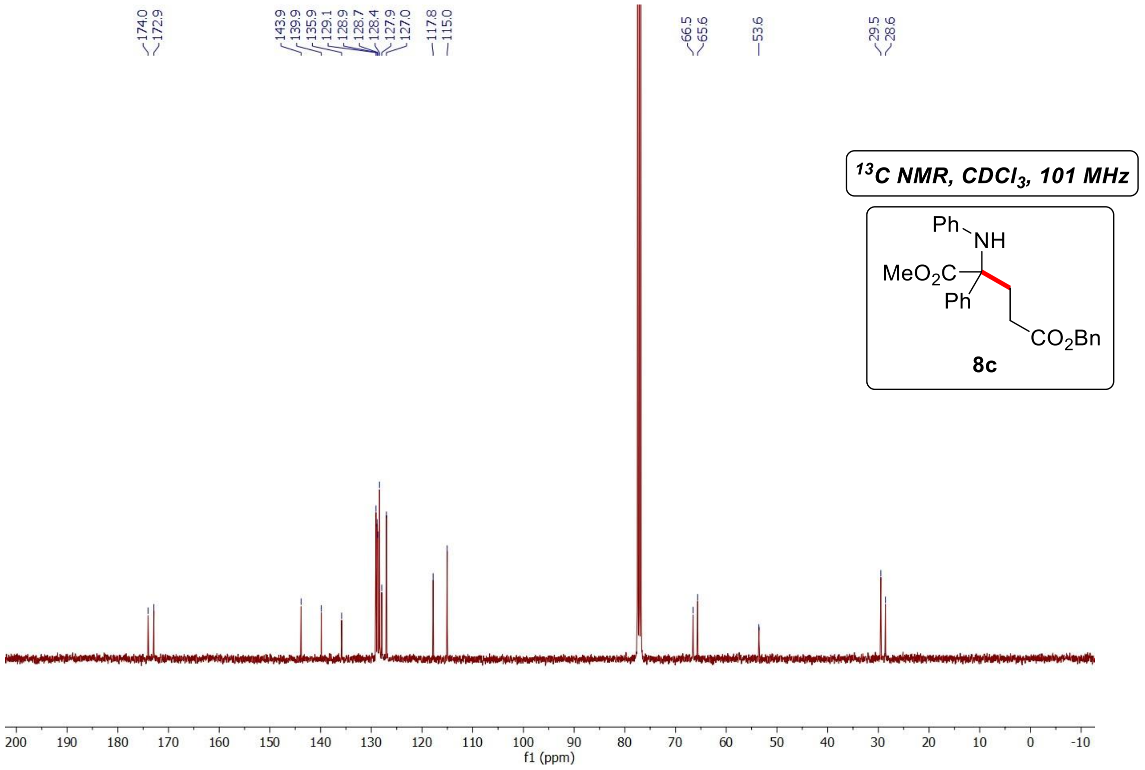

7.60. Methyl 5-(dimethylamino)-5-oxo-2-phenyl-2-(phenylamino)pentanoate (8d)

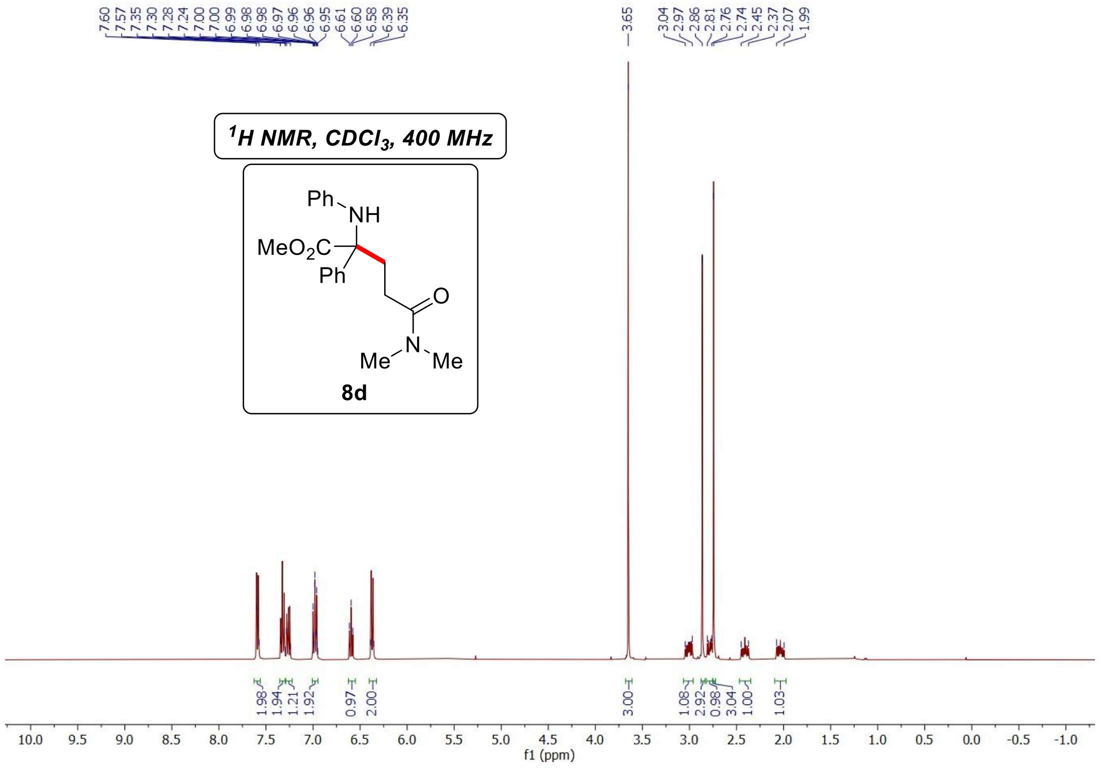

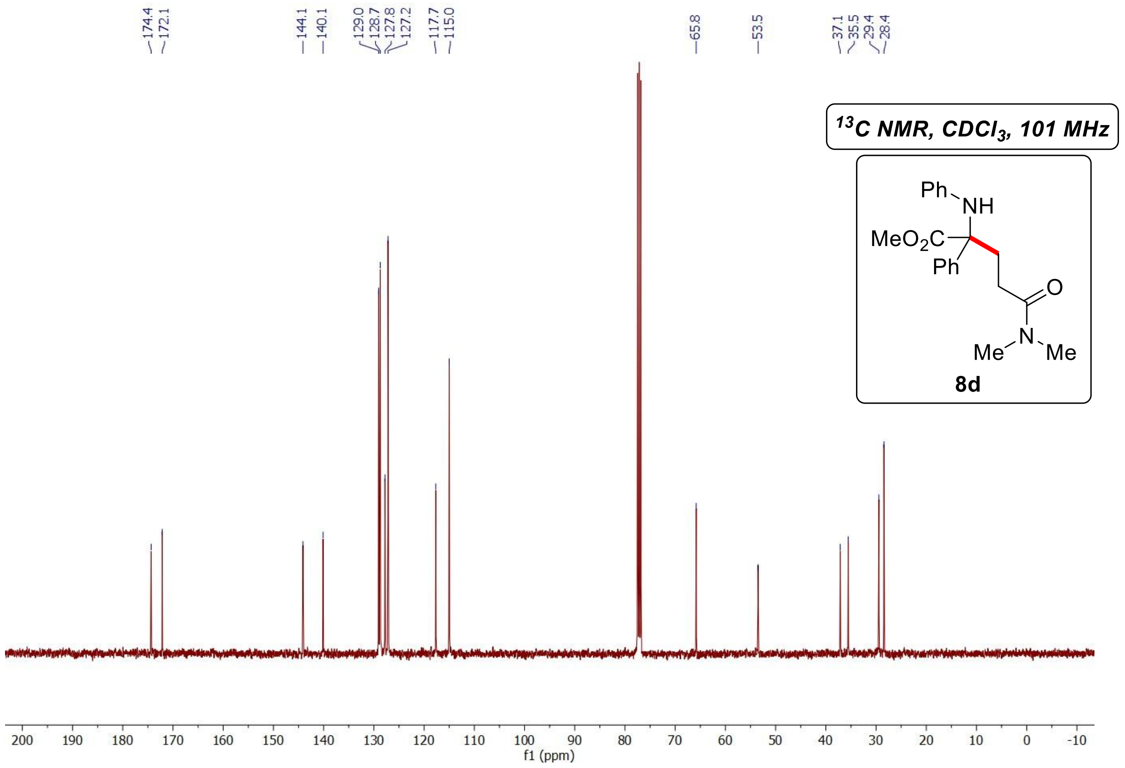

7.61. N-(4,4-difluoro-1,1,3-triphenylbut-3-en-1-yl)aniline (8e)

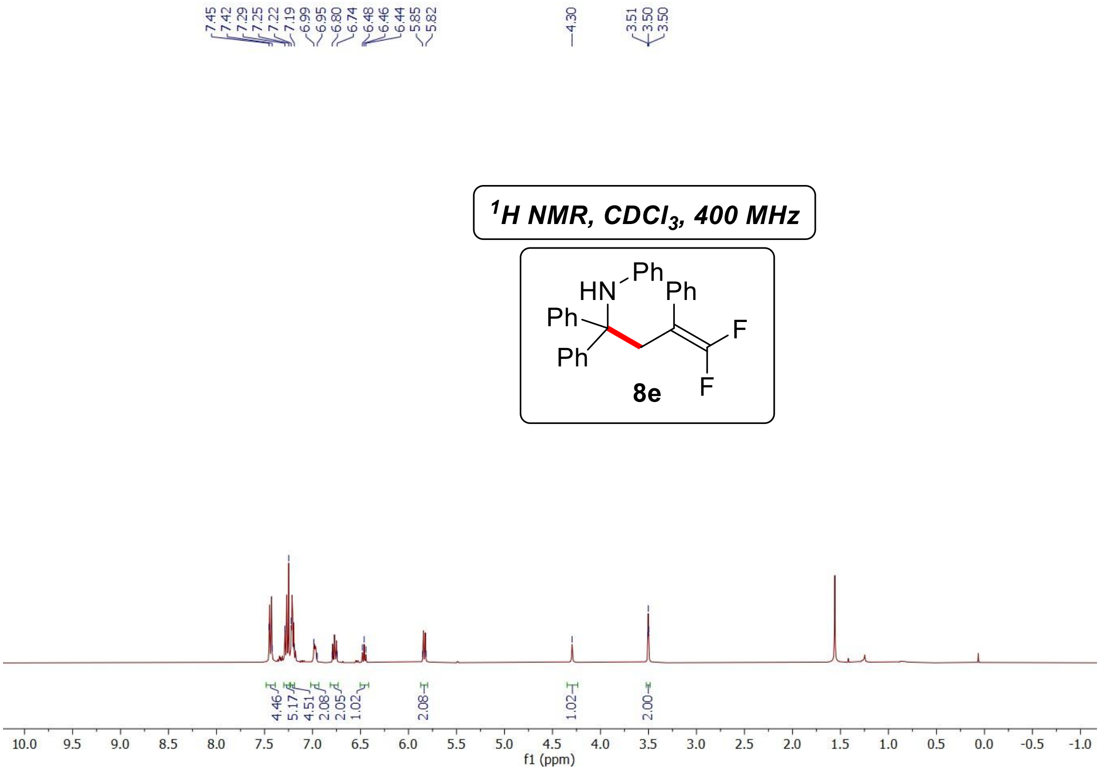

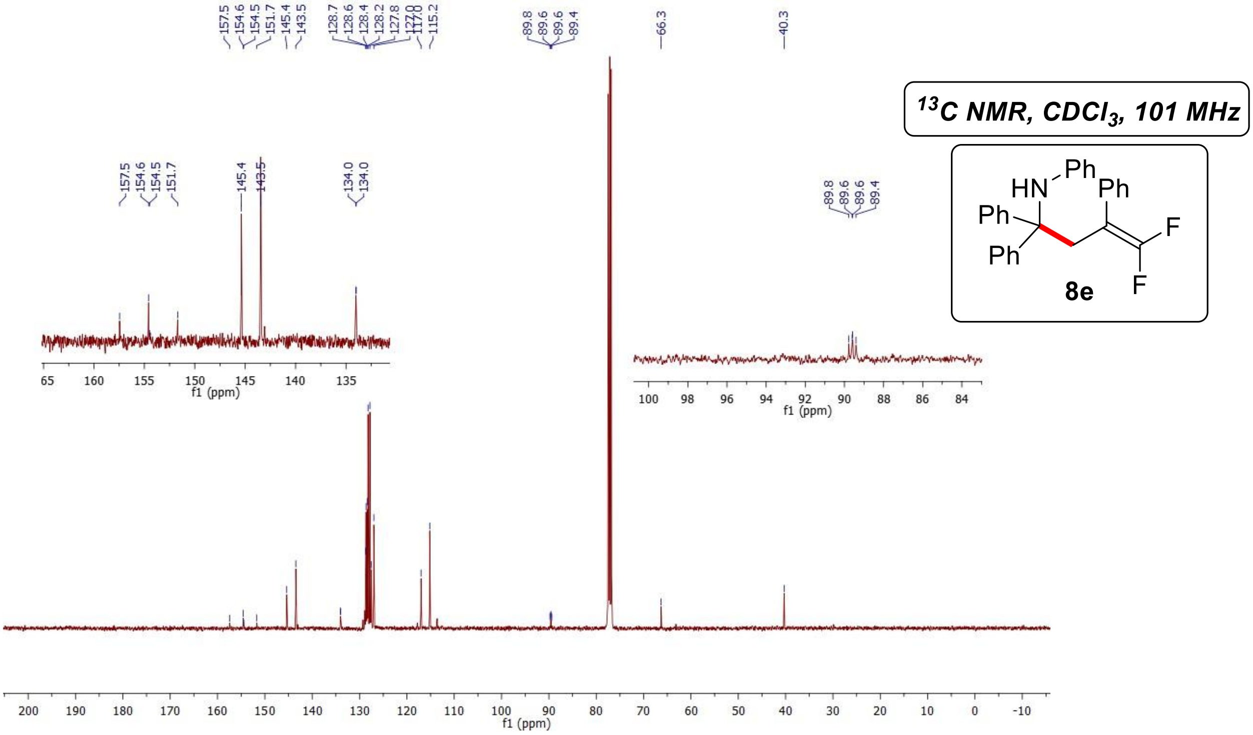

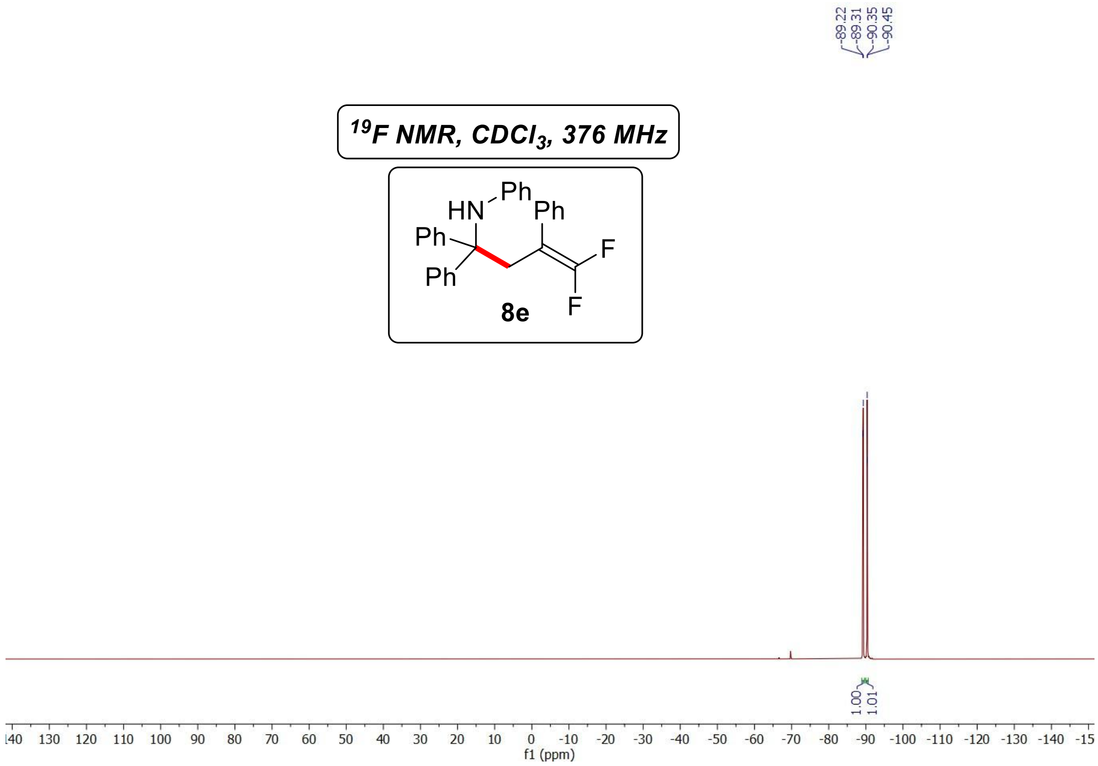

-89.22
-89.31
-90.35
-90.45
^{19}F NMR, CDCl$_3$, 376 MHz
HN
Ph
Ph
Ph
Ph
F
F
8e
1.00
1.01
140 130 120 110 100 90 80 70 60 50 40 30 20 10 0 -10 -20 -30 -40 -50 -60 -70 -80 -90 -100 -110 -120 -130 -140 -15
f1 (ppm)

7.62. Diethyl 2-(3,3-difluoro-2-phenylallyl)-2-(phenylamino)malonate (8f)

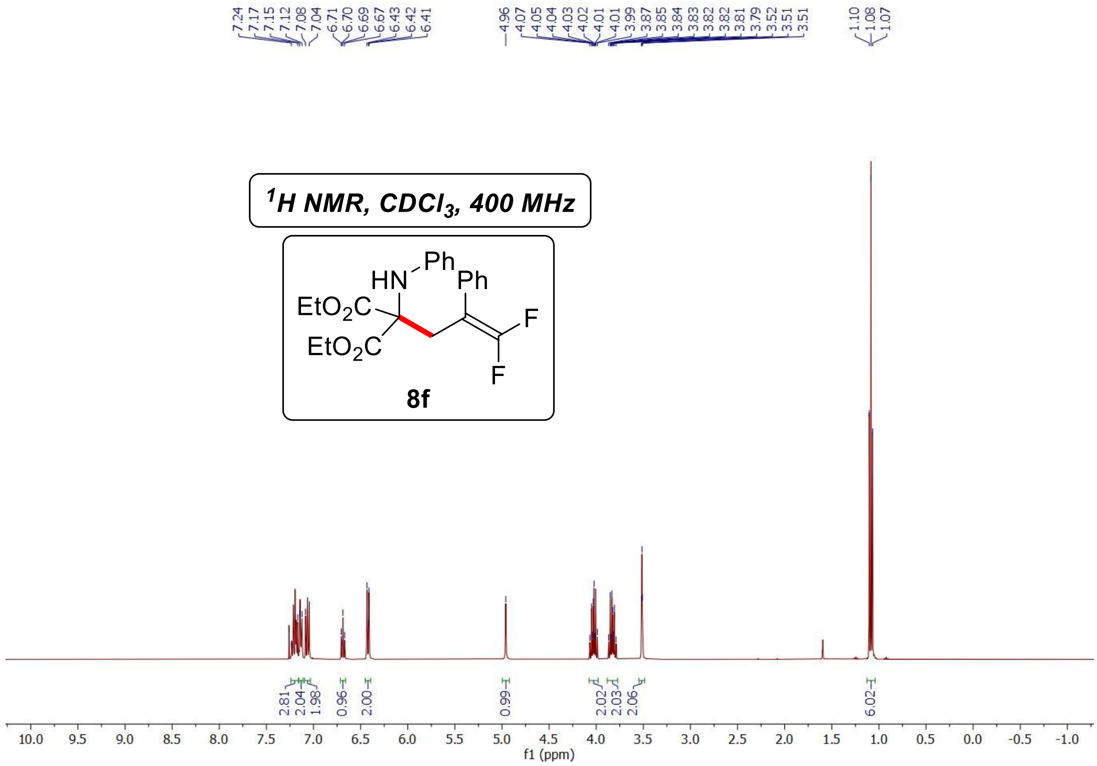

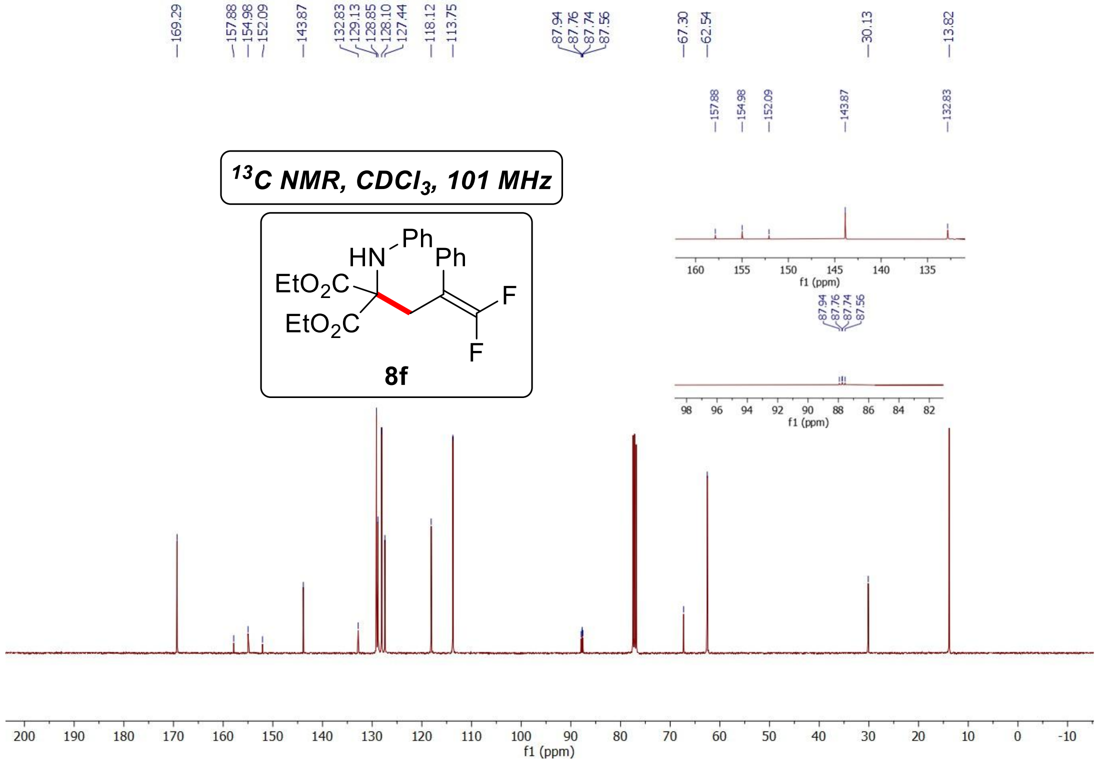

^{19}F NMR, $CDCl_3$, 376 MHz

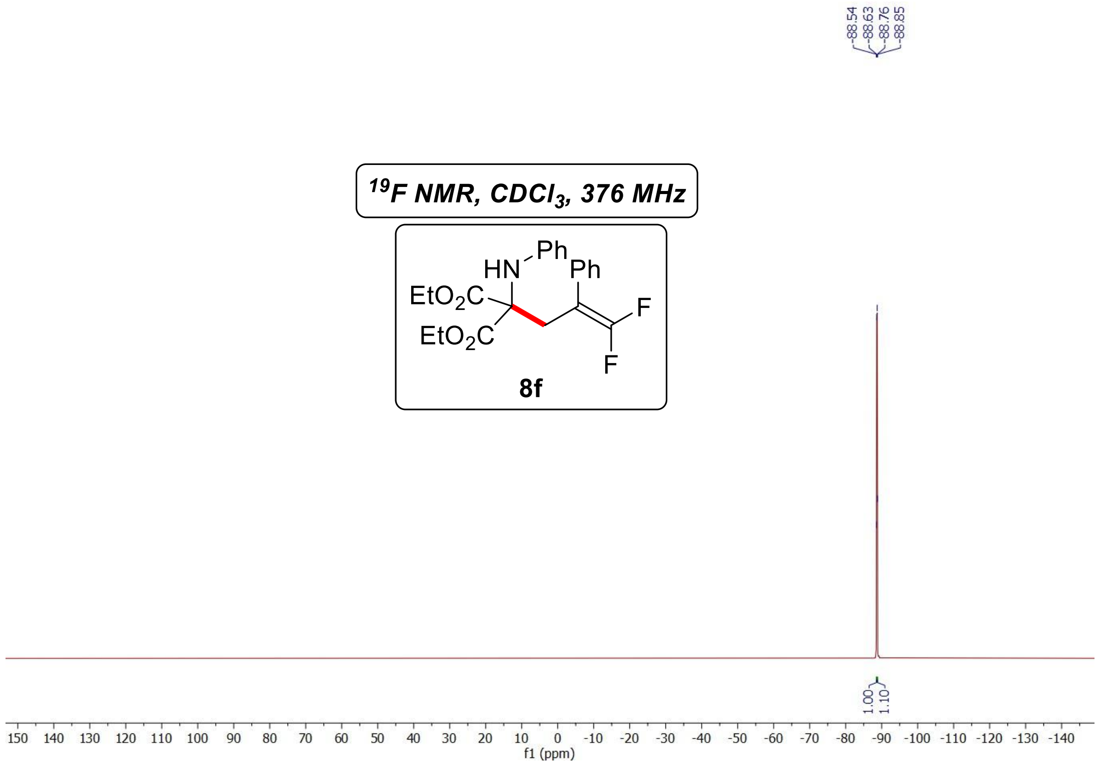

7.63 (*S*)-N-(2-methyl-1-phenylpropyl)aniline (9a)

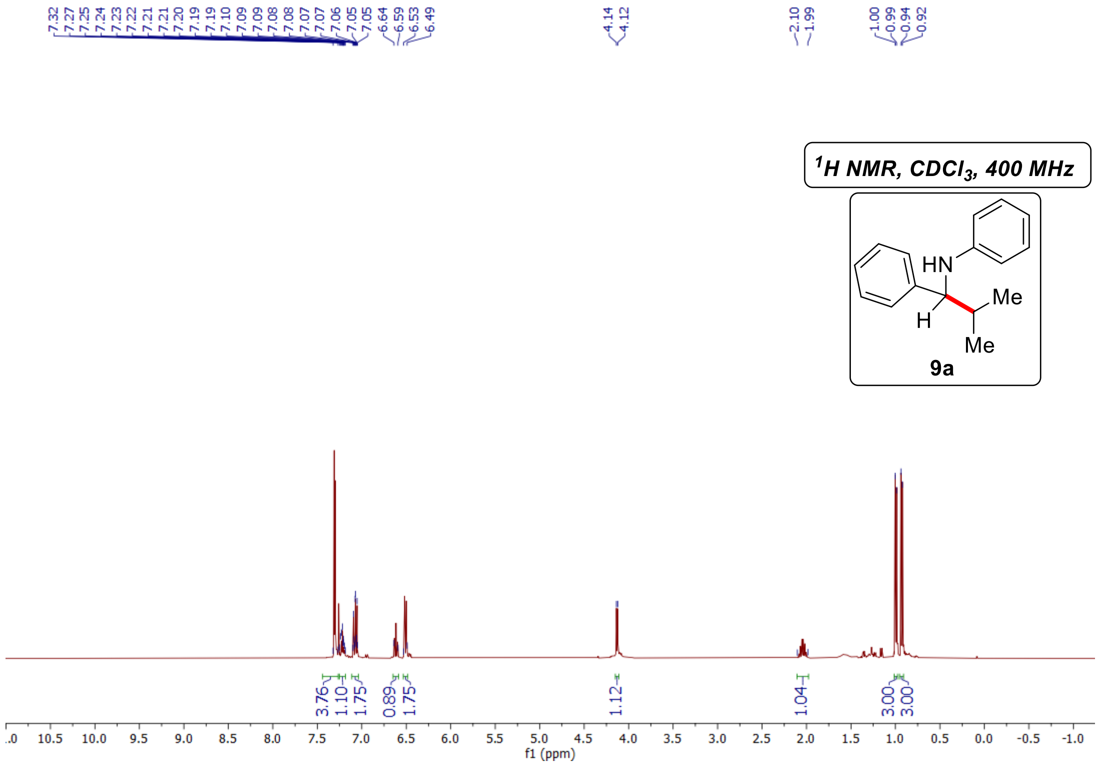

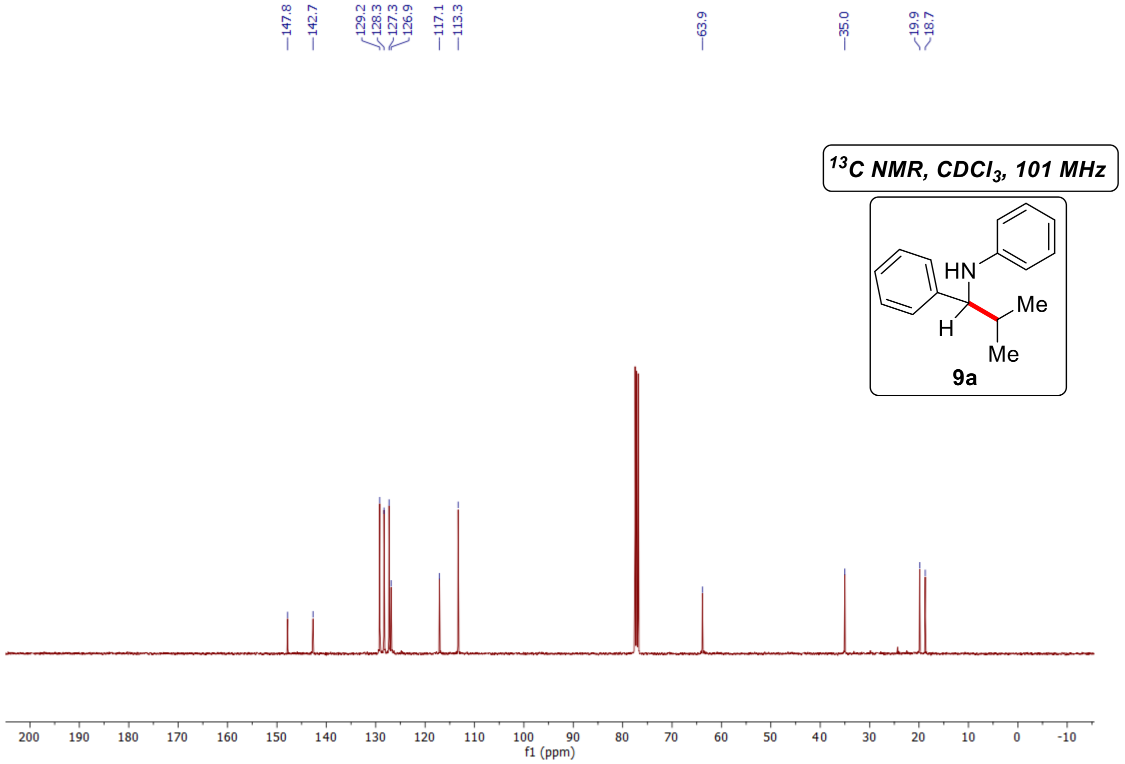

7.64 (*S*)-4-methoxy-N-(2-methyl-1-phenylpropyl)aniline (9b)

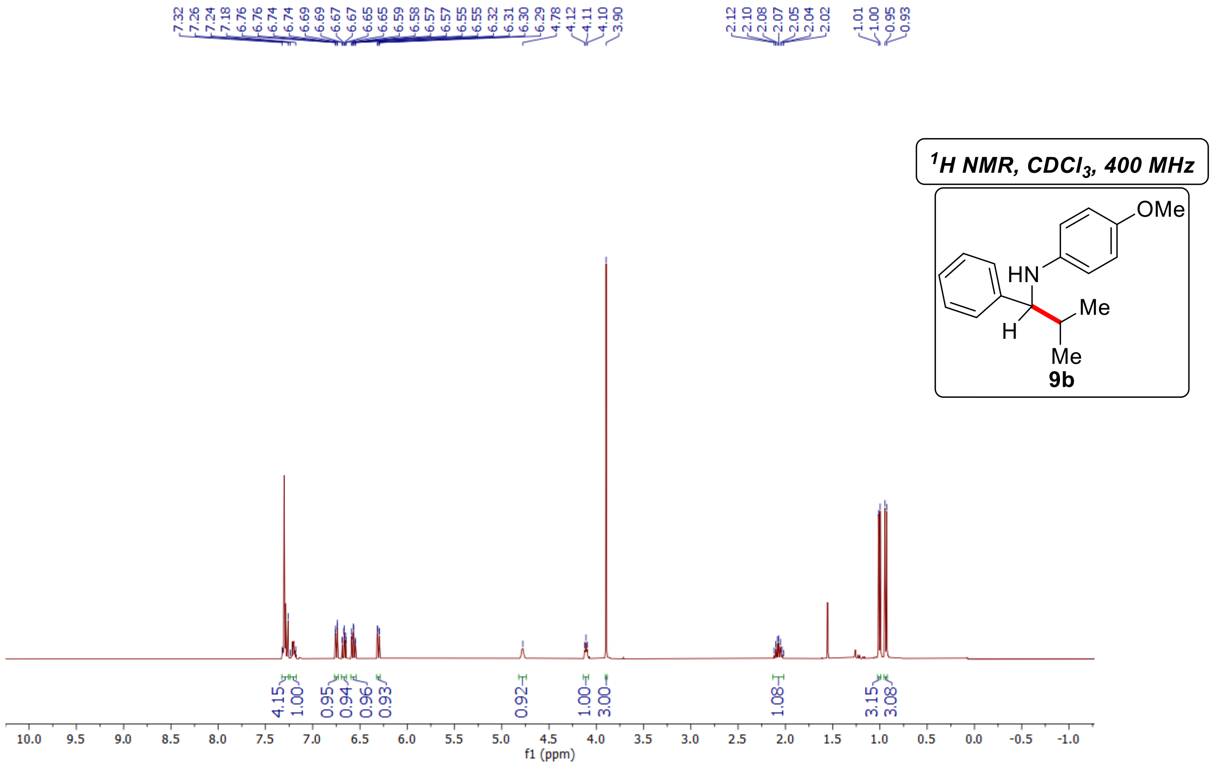

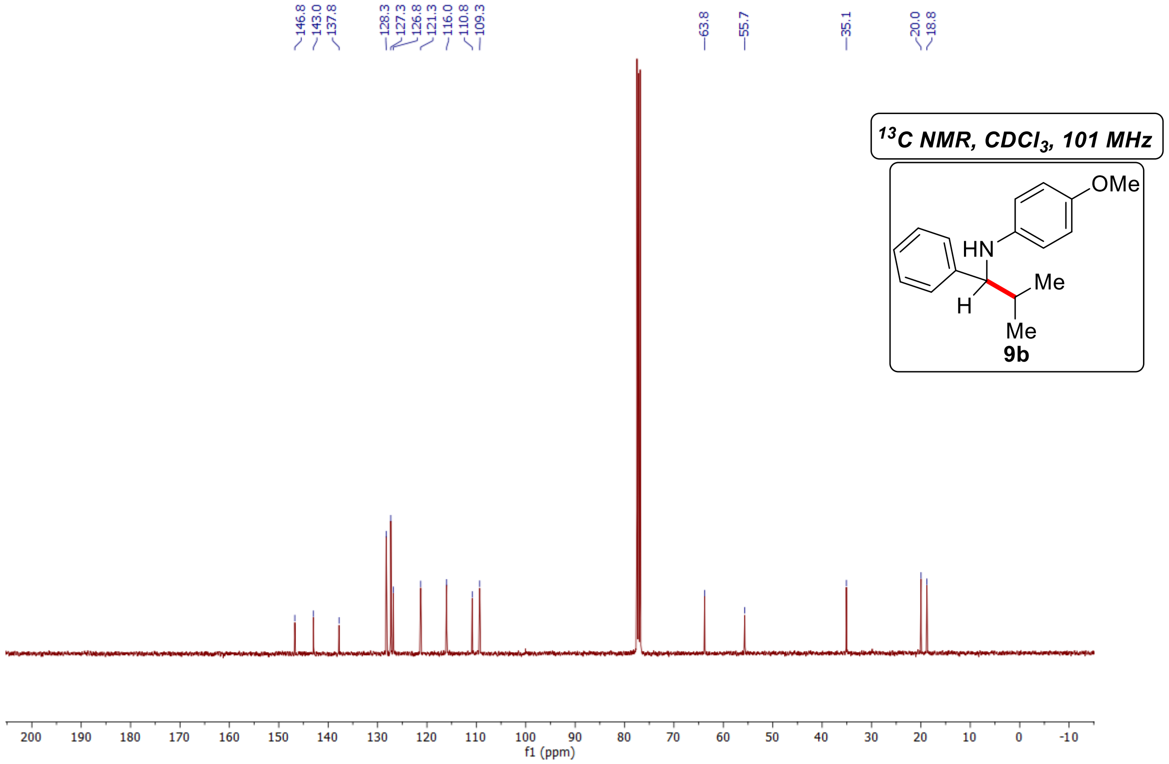

7.65 Diethyl 2-isopropyl-2-(phenylamino)malonate (9c)

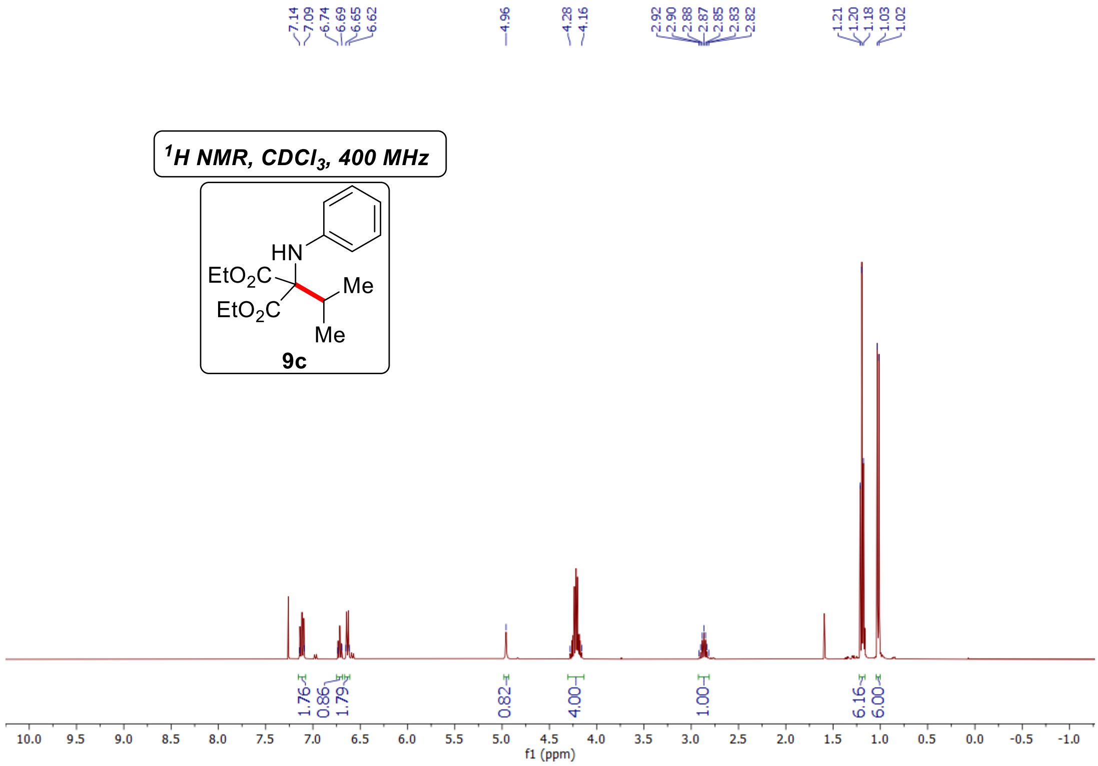

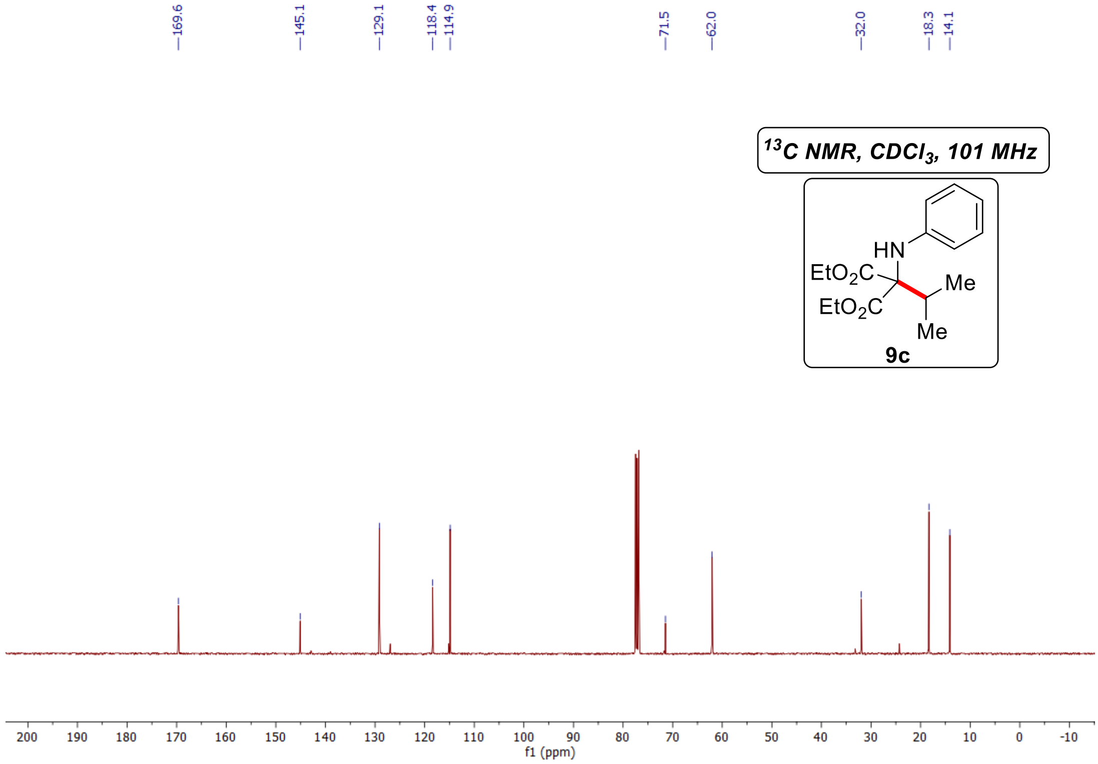

7.66 *N*-(3-methyl-2-phenylbutan-2-yl)aniline (9d)

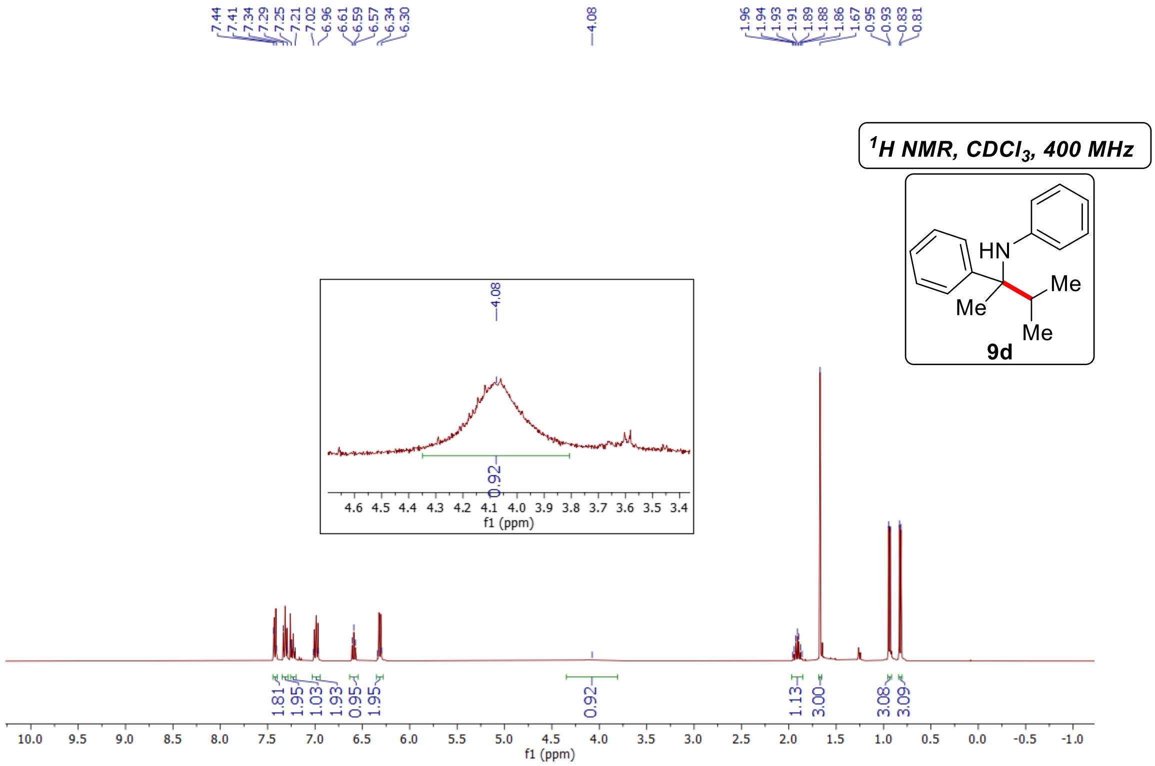

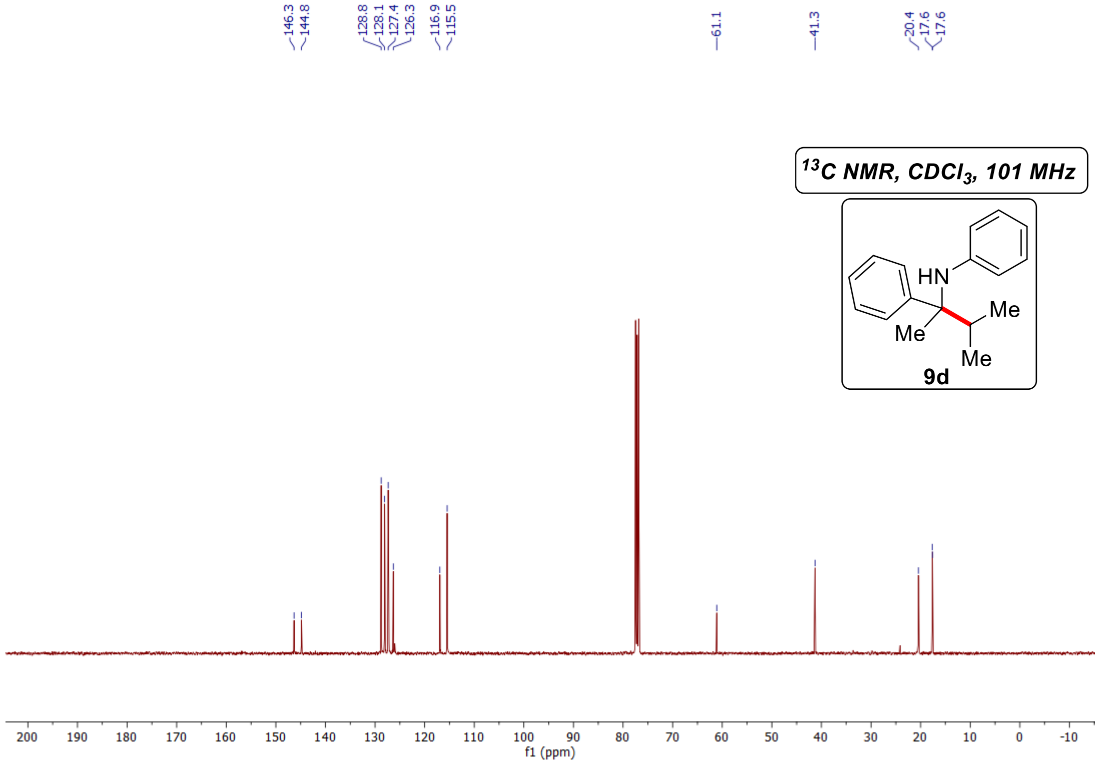

7.67 2-Methoxy-*N*-(2-methyl-1,1-diphenylpropyl)aniline (9e)

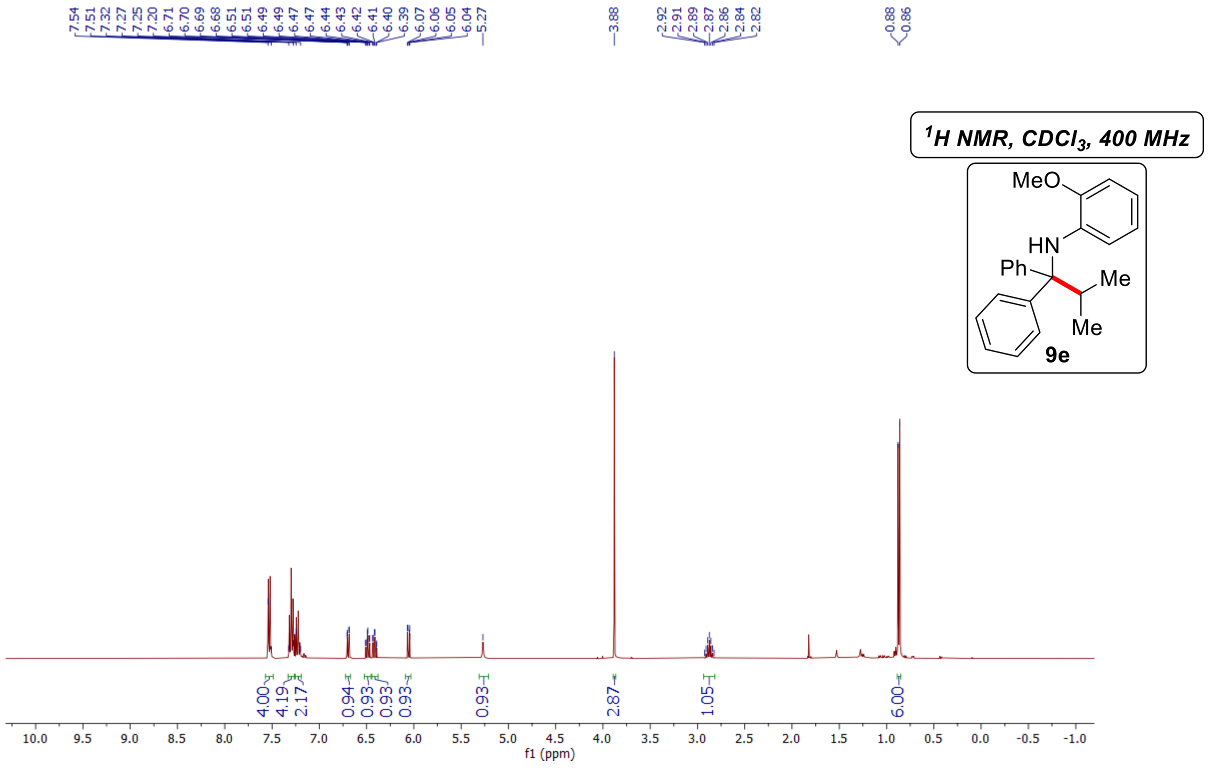

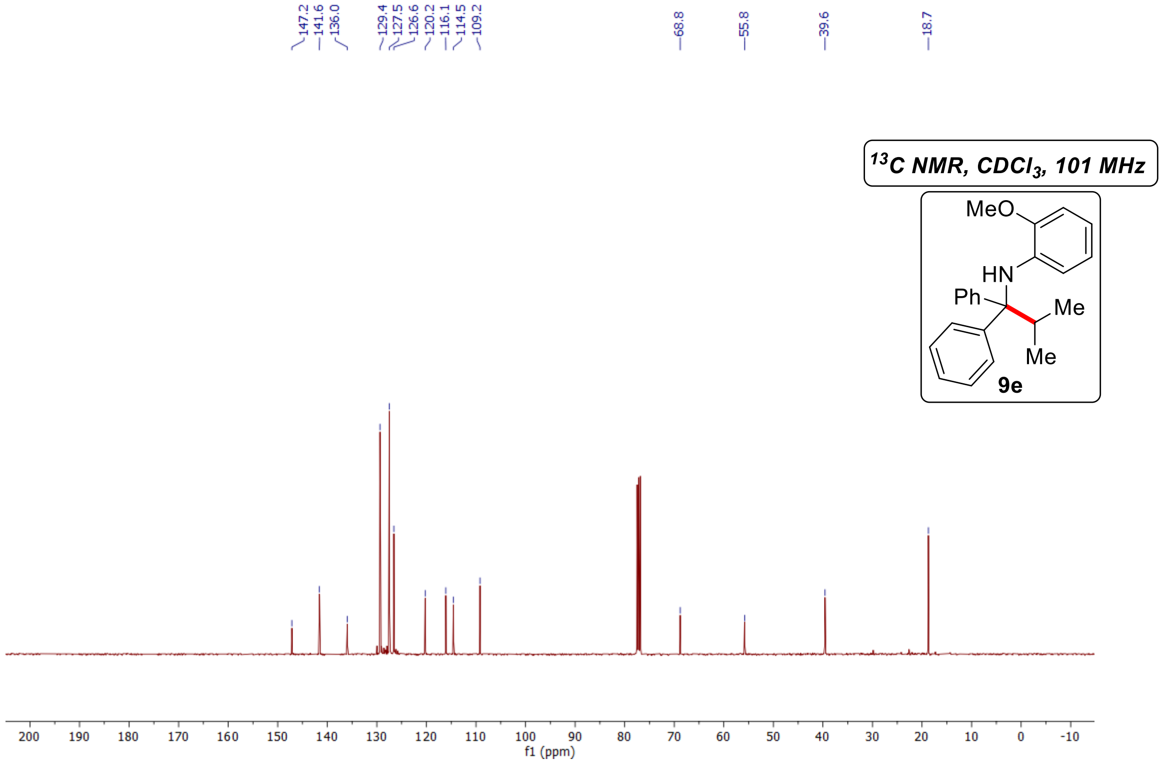

7.68 *N*-(1,1,1-trifluoro-3-methyl-2-phenylbutan-2-yl)aniline (9f)

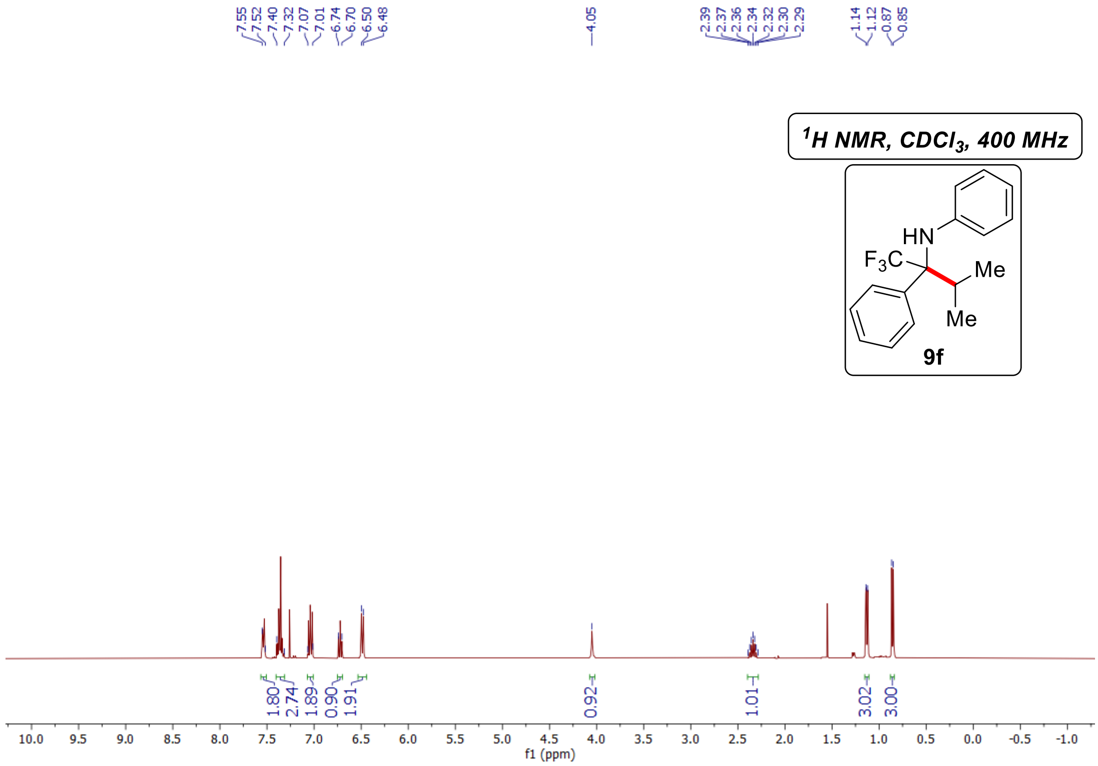

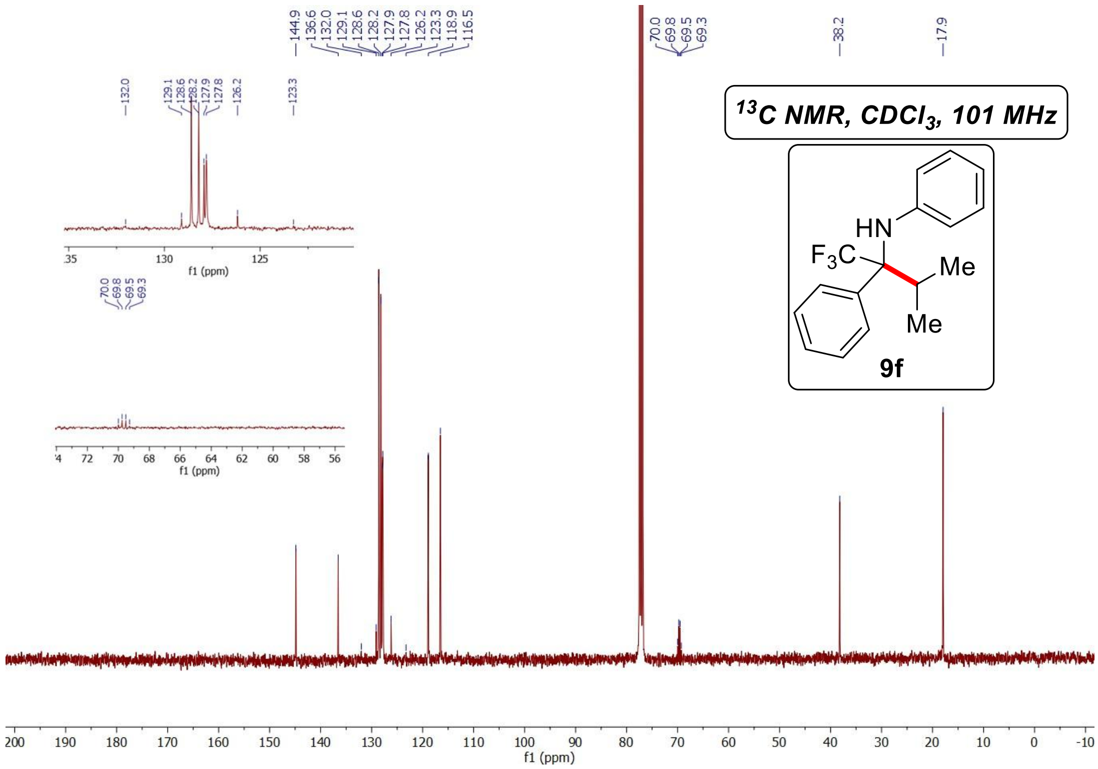

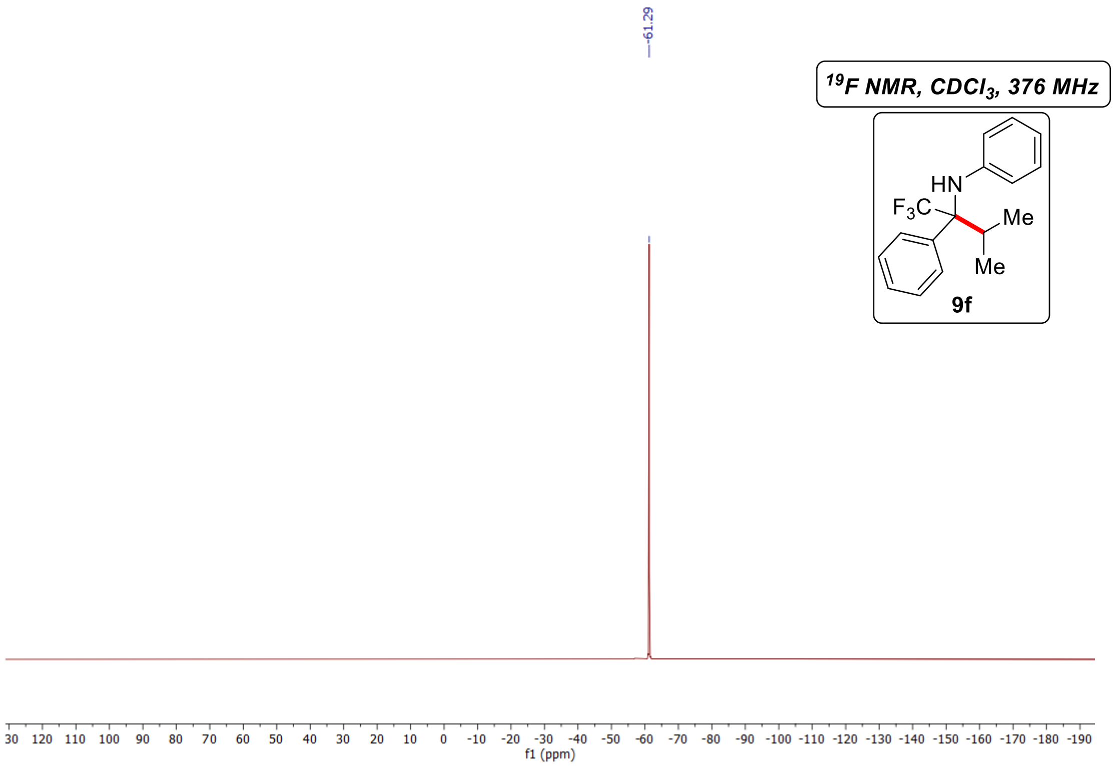

^{19}F NMR, $CDCl_3$, 376 MHz

9f

7.69 *N*-benzyl-2-methyl-1,1-diphenylpropan-1-amine (9g)

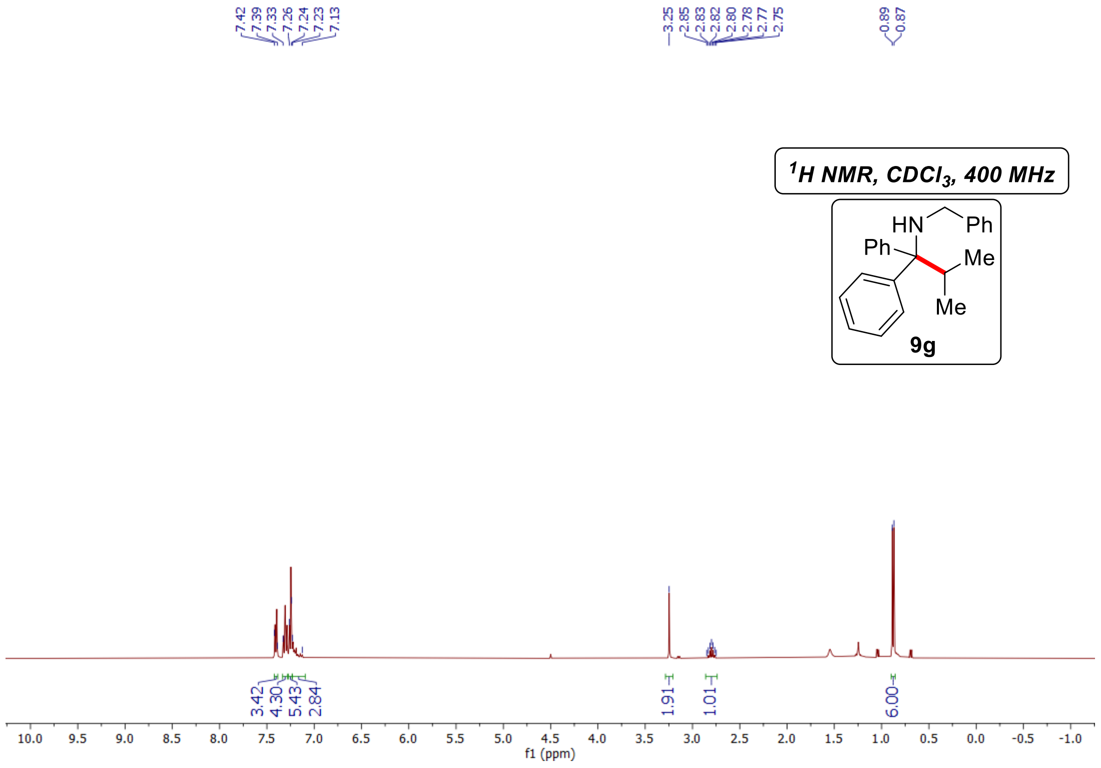

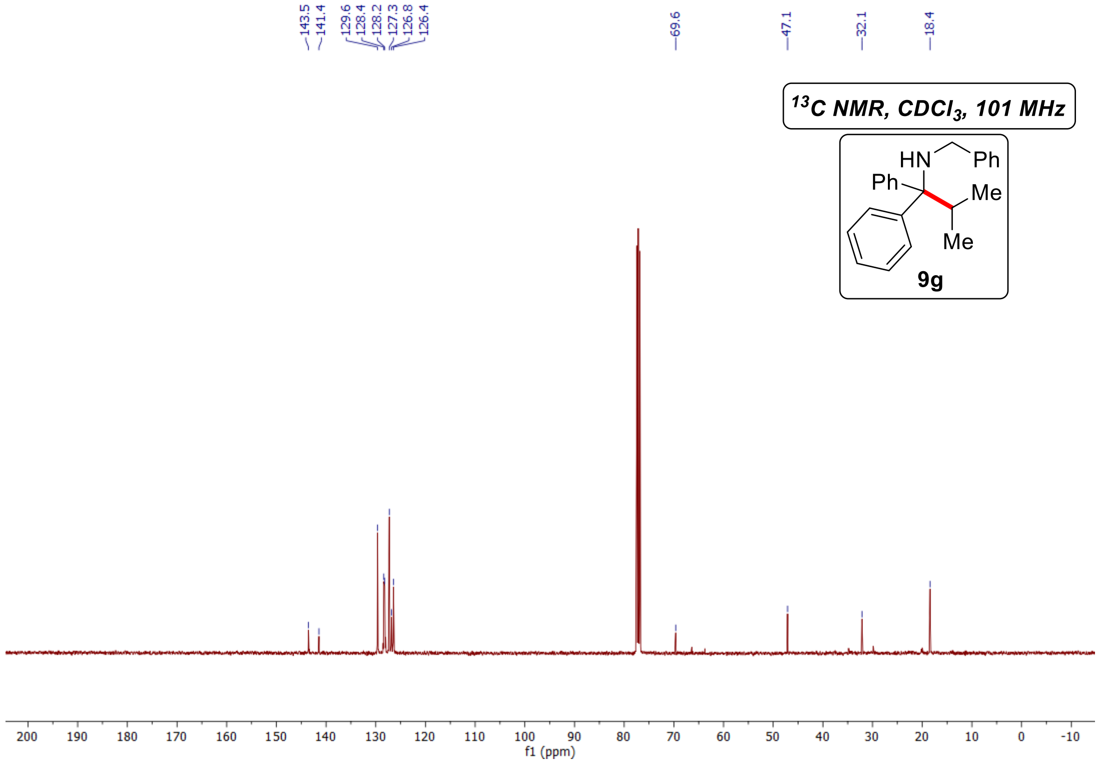

8.0 X-ray single crystal data and structure refinement for substrate 6n

Growth of single crystal: Taking 10 mg of the compound **6n** in a small vial (volume 5 mL) we dissolved it in 0.4 mL DCM-ACN solvent mixture (1:1). Then the system was kept at room temperature for 3 days without any disturbance. Sets of single crystals were formed by slow evaporation process.

8.1. Details of XRD study for 6n

For the determination of X–ray crystal structures of **6n** a single crystal was selected and mounted on Hampton cyoloops[11]. The data was collected at 298K on a CMOS based Bruker SMART APEX2 area detector diffractometer equipped with an INCOATEC micro–focus source with graphite monochromatic Mo Kα radiation (λ = 0.71073 Å) operation at 50 kV and 30 mA. For the integration of diffraction profiles SAINT program was used. Absorption correction was done applying SADABS program. Using Olex2, the structure was solved with the SHELXT structure solution program using Intrinsic phasing and refined with the SHELXL refinement package applying Least Squares minimization method. All the non–hydrogen atoms in the structure were located the Fourier map and refined anisotropically. The hydrogen atoms were fixed by HFIX in their ideal positions and refined using riding model with isotropic thermal parameters. The crystal structure (excluding structure factor) has been deposited to Cambridge Crystallographic Data Centre and allocated deposition number for the compound **6n** is CCDC 2410053.

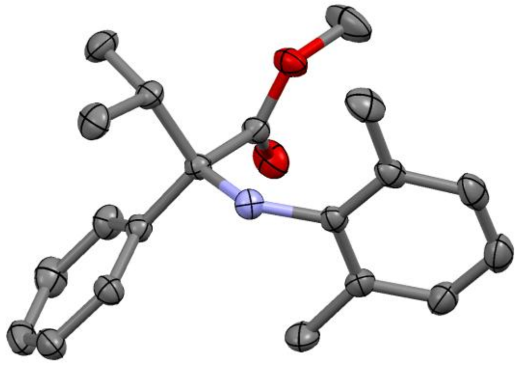

Figure S28: *ORTEP drawing of compound **6n** showing thermal ellipsoids at the 30% probability. Atom colour code: grey = carbon atom, blue = nitrogen atom and red = oxygen atom*

Table 10: *Crystal data and structure refinement for.**6n***

Ccdc no.	2410053
Empirical formula	$C_{20}H_{25}NO_2$
Formula weight	311.41
Temperature/K	298
Crystal system	triclinic
Space group	P-1
a/Å	7.8403(11)
b/Å	10.3493(13)
c/Å	11.6156(16)
α/°	96.969(4)
β/°	99.160(4)
γ/°	106.540(4)
Volume/Å^3	878.0(2)
Z	2
ρ_{calc}g/cm^3	1.178
μ/mm^{-1}	0.075
F(000)	336.0
Crystal size/mm^3	0.231 × 0.156 × 0.123
Radiation	MoKα (λ = 0.71073)
2Θ range for data collection/°	4.17 to 56.768
Index ranges	$-10 \le h \le 10$, $-13 \le k \le 13$, $-15 \le l \le 15$
Reflections collected	28905
Independent reflections	4365 [R_{int} = 0.0497, R_{sigma} = 0.0269]
Data/restraints/parameters	4365/0/217
Goodness-of-fit on F^2	1.094
Final R indexes [I>=2σ (I)]	R_1 = 0.0550, wR_2 = 0.1389
Final R indexes [all data]	R_1 = 0.0604, wR_2 = 0.1427
Largest diff. peak/hole / e Å^{-3}	0.25/-0.21

Table 11: *Fractional atomic coordinates (×10^4) and equivalent isotropic displacement parameters (Å^2×10^3) for 6n. U_{eq} is defined as 1/3 of the trace of the orthogonalised U_{IJ} tensor*

Atom	*x*	*y*	*z*	U(eq)
O3	6207.8(16)	7210.2(13)	4434.1(10)	60.9(3)
O2	3304.3(14)	6274.3(12)	3533.4(9)	51.0(3)
N1	5145.6(16)	6640.5(11)	1525.7(10)	37.9(3)
C5	5857(3)	2844.4(17)	2055.6(17)	63.3(5)
C6	4184(3)	2910.6(16)	1556.3(16)	59.2(4)
C7	3900(2)	4156.4(15)	1389.1(13)	46.5(3)
C1	5380.8(18)	5366.6(13)	1744.6(11)	37.2(3)
C9	5174.6(17)	7735.8(13)	2495.3(11)	33.9(3)
C10	6887.4(17)	8968.3(13)	2679.6(11)	36.3(3)
C15	7646(2)	9851.3(15)	3759.9(13)	48.7(3)
C14	9141(2)	10999.8(17)	3862.5(17)	60.9(4)
C13	9887(2)	11303.1(17)	2900.5(18)	60.3(4)

Atom	*x*	*y*	*z*	U(eq)
C4	7294(3)	4024.1(17)	2391.7(16)	56.8(4)
C2	7097(2)	5297.0(15)	2246.1(13)	44.4(3)
C3	8747(2)	6540.6(17)	2593.1(18)	57.5(4)
C8	2046(2)	4181.8(19)	840.3(17)	64.7(5)
C12	9138(2)	10449.6(17)	1819.3(16)	54.2(4)
C11	7660.1(19)	9292.7(15)	1711.8(13)	43.5(3)
C16	3454.9(18)	8202.4(15)	2144.6(12)	41.8(3)
C18	3375(2)	8778.4(19)	991.3(15)	57.1(4)
C17	3240(3)	9217.6(19)	3134.0(17)	60.2(4)
C19	5002.3(18)	7080.7(14)	3617.4(12)	38.8(3)
C20	2952(3)	5548(3)	4494(2)	89.5(8)

Table 12: *Anisotropic displacement parameters ($Å^2 \times 10^3$) for* ***6n****. The anisotropic displacement factor exponent takes the form: $-2\pi^2[h^2a^{*2}U_{11}+2hka^*b^*U_{12}+...]$*

Atom	U_{11}	U_{22}	U_{33}	U_{23}	U_{13}	U_{12}
O3	57.3(7)	72.6(8)	49.5(6)	27.4(6)	-0.3(5)	14.2(6)
O2	44.3(6)	63.8(7)	51.2(6)	28.1(5)	17.3(4)	14.9(5)
N1	39.4(6)	38.3(6)	33.1(5)	3.9(4)	4.1(4)	10.6(5)
C5	83.8(13)	42.3(8)	71.9(11)	11.9(8)	27.1(10)	25.4(8)
C6	73.6(11)	35.6(7)	60.6(10)	0.4(7)	26.0(8)	1.4(7)
C7	47.5(8)	42.6(7)	43.6(7)	0.3(6)	16.5(6)	3.7(6)
C1	40.1(7)	35.7(6)	36.2(6)	4.1(5)	14.7(5)	9.4(5)
C9	33.9(6)	36.5(6)	32.4(6)	7.0(5)	7.4(5)	12.0(5)
C10	35.8(6)	35.1(6)	38.3(6)	7.4(5)	4.9(5)	12.2(5)
C15	53.2(8)	45.2(8)	43.7(7)	1.4(6)	5.0(6)	14.5(6)
C14	59.5(10)	44.9(8)	63.1(10)	-6.0(7)	-7.0(8)	9.3(7)
C13	44.6(8)	41.6(8)	86.9(13)	13.8(8)	3.3(8)	5.5(6)
C4	63.6(10)	53.0(9)	64.3(10)	13.5(7)	20.1(8)	29.6(8)
C2	44.6(7)	42.8(7)	49.5(8)	8.1(6)	16.5(6)	15.7(6)
C3	35.5(7)	55.7(9)	80.2(11)	11.4(8)	7.6(7)	15.0(7)
C8	46.7(9)	61.0(10)	67.2(11)	-1.7(8)	4.3(8)	-3.8(7)
C12	46.8(8)	50.5(8)	68.1(10)	23.7(7)	16.3(7)	11.4(7)
C11	43.5(7)	43.3(7)	43.0(7)	11.8(6)	8.9(6)	10.6(6)
C16	36.6(6)	45.9(7)	47.3(7)	14.3(6)	7.9(5)	17.2(6)
C18	57.5(9)	63.7(10)	55.9(9)	23.9(8)	3.0(7)	26.7(8)
C17	63.4(10)	63.8(10)	69.1(11)	14.0(8)	22.3(8)	38.6(9)
C19	41.5(7)	42.0(7)	37.2(6)	9.9(5)	10.8(5)	16.8(5)
C20	65.6(12)	124.0(19)	89.8(15)	74.5(15)	26.3(11)	18.0(12)

Table 13: *Bond Lengths for* **6n.**

Atom	Atom	Length/Å	Atom	Atom	Length/Å
O3	C19	1.1928(17)	C9	C16	1.5687(17)
O2	C19	1.3359(17)	C9	C19	1.5477(17)
O2	C20	1.4388(19)	C10	C15	1.3921(19)

Atom	Atom	Length/Å	Atom	Atom	Length/Å
N1	C1	1.4289(17)	C10	C11	1.3915(19)
N1	C9	1.4904(16)	C15	C14	1.389(2)
C5	C6	1.371(3)	C14	C13	1.370(3)
C5	C4	1.369(3)	C13	C12	1.379(3)
C6	C7	1.400(2)	C4	C2	1.396(2)
C7	C1	1.4075(19)	C2	C3	1.501(2)
C7	C8	1.500(2)	C12	C11	1.386(2)
C1	C2	1.404(2)	C16	C18	1.530(2)
C9	C10	1.5307(17)	C16	C17	1.524(2)

Table 14: *Bond Angles for* ***6n*.**

Atom	Atom	Atom	Angle/°	Atom	Atom	Atom	Angle/°
C19	O2	C20	116.97(13)	C11	C10	C9	119.16(11)
C1	N1	C9	122.48(10)	C11	C10	C15	117.65(13)
C4	C5	C6	119.31(16)	C14	C15	C10	120.70(15)
C5	C6	C7	121.75(15)	C13	C14	C15	120.88(15)
C6	C7	C1	118.64(15)	C14	C13	C12	119.23(15)
C6	C7	C8	119.98(14)	C5	C4	C2	121.61(17)
C1	C7	C8	121.37(14)	C1	C2	C3	122.19(13)
C7	C1	N1	119.61(13)	C4	C2	C1	119.09(14)
C2	C1	N1	120.69(12)	C4	C2	C3	118.68(14)
C2	C1	C7	119.59(13)	C13	C12	C11	120.29(16)
N1	C9	C10	111.35(10)	C12	C11	C10	121.24(14)
N1	C9	C16	108.02(10)	C18	C16	C9	113.88(12)
N1	C9	C19	107.07(10)	C17	C16	C9	112.46(12)
C10	C9	C16	109.30(10)	C17	C16	C18	109.86(13)
C10	C9	C19	112.77(10)	O3	C19	O2	123.26(13)
C19	C9	C16	108.16(10)	O3	C19	C9	126.06(13)
C15	C10	C9	123.02(12)	O2	C19	C9	110.60(11)

Table 15: *Torsion Angles for* **6n**

A	B	C	D	Angle/°	A	B	C	D	Angle/°
N1	C1	C2	C4	-176.77(13)	C10	C9	C16	C18	61.07(15)
N1	C1	C2	C3	1.0(2)	C10	C9	C16	C17	-64.73(15)
N1	C9	C10	C15	-153.06(12)	C10	C9	C19	O3	-19.91(19)
N1	C9	C10	C11	31.83(16)	C10	C9	C19	O2	163.16(11)
N1	C9	C16	C18	-60.23(15)	C10	C15	C14	C13	1.1(3)
N1	C9	C16	C17	173.97(12)	C15	C10	C11	C12	0.3(2)
N1	C9	C19	O3	102.91(16)	C15	C14	C13	C12	-0.2(3)
N1	C9	C19	O2	-74.02(13)	C14	C13	C12	C11	-0.6(3)
C5	C6	C7	C1	0.1(2)	C13	C12	C11	C10	0.5(2)
C5	C6	C7	C8	-179.93(16)	C4	C5	C6	C7	-0.6(3)
C5	C4	C2	C1	0.1(2)	C8	C7	C1	N1	-3.3(2)
C5	C4	C2	C3	-177.75(16)	C8	C7	C1	C2	-179.50(14)

A	B	C	D	Angle/°	A	B	C	D	Angle/°
C6	C5	C4	C2	0.5(3)	C11	C10	C15	C14	-1.1(2)
C6	C7	C1	N1	176.71(13)	C16	C9	C10	C15	87.68(15)
C6	C7	C1	C2	0.5(2)	C16	C9	C10	C11	-87.43(14)
C7	C1	C2	C4	-0.6(2)	C16	C9	C19	O3	-140.90(15)
C7	C1	C2	C3	177.21(14)	C16	C9	C19	O2	42.17(14)
C1	N1	C9	C10	109.74(13)	C19	C9	C10	C15	-32.67(17)
C1	N1	C9	C16	-130.24(12)	C19	C9	C10	C11	152.23(12)
C1	N1	C9	C19	-13.96(16)	C19	C9	C16	C18	-175.80(12)
C9	N1	C1	C7	110.32(14)	C19	C9	C16	C17	58.40(15)
C9	N1	C1	C2	-73.49(16)	C20	O2	C19	O3	0.5(2)
C9	C10	C15	C14	-176.28(14)	C20	O2	C19	C9	177.52(16)
C9	C10	C11	C12	175.70(13)					

Table 16: *Hydrogen atom coordinates (Å×10^4) and isotropic displacement parameters (Å^2×10^3) for* ***6n***

Atom	*x*	*y*	*z*	U(eq)
H5	6013.48	2006.83	2164.92	76
H6	3212.54	2106.55	1322.43	71
H15	7146.27	9670.21	4419.8	58
H14	9643.08	11570.5	4593.91	73
H13	10886.05	12075.42	2975.58	72
H4	8430	3976.62	2725.02	68
H3A	8824.92	7049.2	1954.66	86
H3B	9815.26	6262.26	2761.27	86
H3C	8656.06	7105.79	3285.33	86
H8A	1214.76	3267.35	663.64	97
H8B	2100.82	4550.66	122.66	97
H8C	1635.68	4743.8	1383.29	97
H12	9628.02	10651.58	1159.7	65
H11	7175.12	8722.29	978.89	52
H16	2394.48	7383.34	2015.23	50
H18A	4360.89	9611.31	1092.51	86
H18B	2241.22	8962.67	791.99	86
H18C	3472.15	8122.54	366.02	86
H17A	3278.05	8846.55	3852.64	90
H17B	2096.29	9382.86	2920.72	90
H17C	4210.34	10062.82	3251.78	90
H20A	3384.91	4768.23	4417.7	134
H20B	1668.74	5246.41	4471.43	134
H20C	3568.4	6143.52	5233.72	134
H1	4220(20)	6522(17)	980(16)	50(5)

9.0 Computational Details

All density functional theory (DFT) calculations were performed within the Gaussian 09 quantum chemical package.[12] All the geometries of stationary points were optimized in the solvent phase with the M06-2X functional,[13] in conjugation with Pople's 6-31G(d,p) double-ζ split valence basis set with diffuse function on all atoms except Cesium which was treated with LANL2DZ atom-centred basis and LANL2 effective core potential.[15] Solvent effect was introduced by utilizing Truhlar and co-workers' SMD[16] solvation model wherein the quantum mechanical charge density of solute molecules is taken into account which interact with continuum description of the solvent. The dielectric constant of acetone (ε = 20.7) was employed all through to mimic the experimental condition.[17] Harmonic vibrational frequencies were computed at the same level of theory, from minima (with all positive Hessian index). The non-thermal zero-point energy (ZPE) correction and the thermal corrections to enthalpy and Gibbs free energy were obtained from frequency calculations, using standard approximations at 298.15 K and 1 atm. pressure. Moreover, the electronic energies were refined by single point calculations with the larger triple-ζ basis set at M06-2X/SMD(Acetone)/6-311+G (d, p) (for rest), LANL2DZ (for Cs).[18] Relative Gibbs free energies reported in the main text are evaluated by adding thermal corrections obtained from vibrational analyses to the solvent-phase electronic energies. Thus, the discussions in the main text are provided based on Gibbs free energies (kcal/mol) at M06-2X/SMD(Acetone)/6-311+G (d, p) (for rest), LANL2DZ (for Cs) //M06-2X/SMD (Acetone)/6 31+G (d, p) (for rest), LANL2DZ (for Cs) level of theory. The vertical excitation energy calculation of EDA complex with the TD-DFT formalism was carried out at M06L/SMD/6 311+G (d, p) (for rest), LANL2DZ (for Cs) which correctly accounts for the various long-range NCIs.[19-20] The non-covalent interaction (NCI) index was analyzed with the Multiwfn program[21] and its plot was graphed with VMD program.[22]

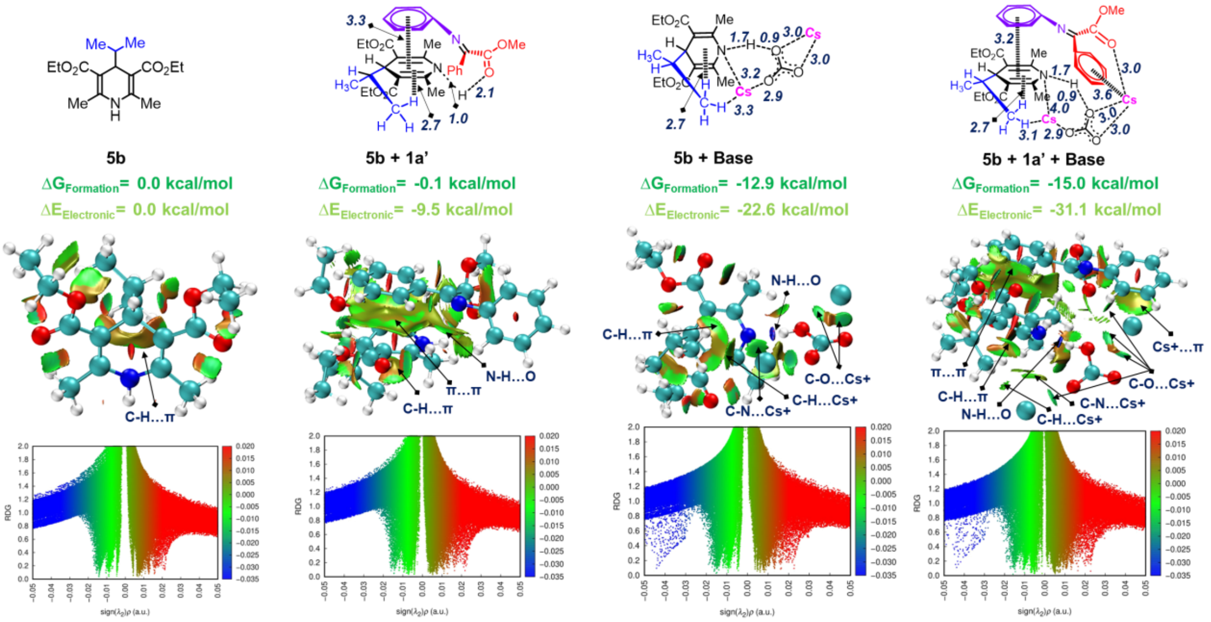

Figure S29. NCIs Plots with optimized geometries and RDG Scatter plots (vertical figures respectively) of **5b**, **5b** with Imine, **5b** with Cs_2CO_3 base and self-assembled EDA complex with **5b**, Imine and base respectively. The distances are shown in Å.

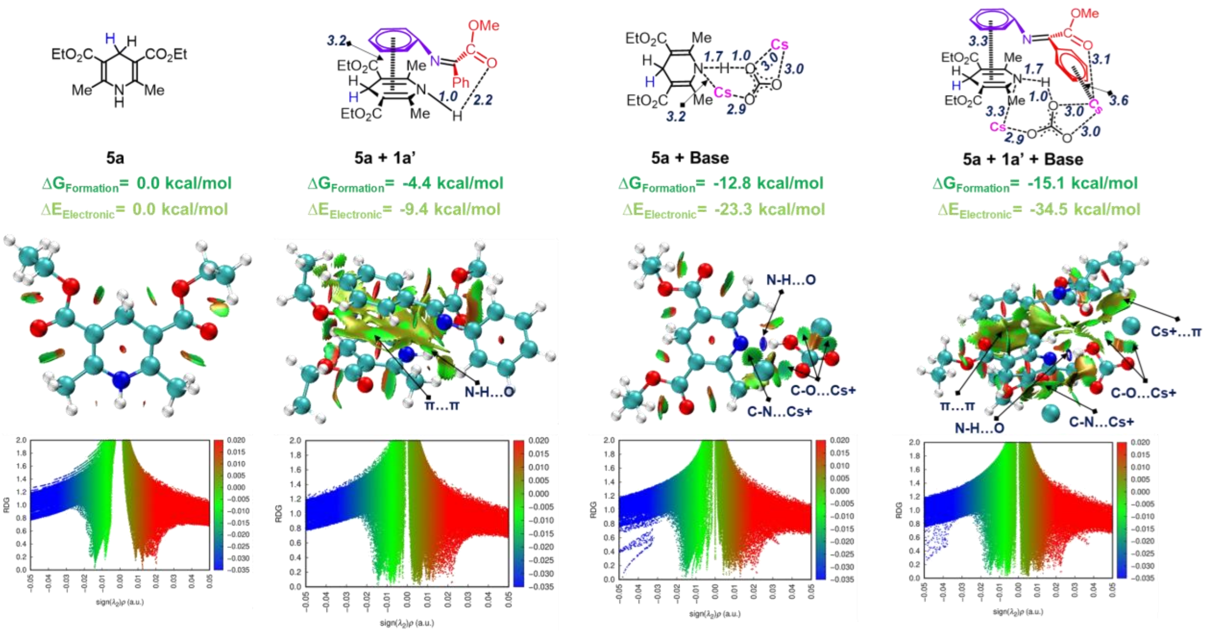

Figure S30. NCIs Plots with optimized geometries and RDG Scatter plots (vertical figures respectively) of **5a**, **5a** with Imine, **5a** with Cs_2CO_3 base and self-assembled EDA complex with 5a, Imine and base respectively. The distances are shown in Å.

XYZ Co-ordinates

E = -862.4055846 a.u.

C	1.217194000	1.935093000	0.136747000
N	-0.000079000	2.574797000	-0.000086000
C	-1.217334000	1.935019000	-0.136695000
C	-1.262307000	0.571631000	-0.141138000
C	1.262228000	0.571711000	0.141219000
C	-0.000017000	-0.268602000	0.000069000
H	-0.096815000	-0.943358000	0.861294000
H	0.096826000	-0.943377000	-0.861127000
H	-0.000207000	3.585345000	0.000697000
C	-2.542131000	-0.127260000	-0.276806000
O	-2.354264000	-1.476014000	-0.272976000
O	-3.656402000	0.376605000	-0.385218000
C	2.542114000	-0.127088000	0.276724000
O	2.354362000	-1.475861000	0.272661000
O	3.656337000	0.376879000	0.385179000

C	-2.393877000	2.865288000	-0.267217000
H	-2.940433000	2.668486000	-1.193295000
H	-3.103255000	2.704175000	0.549422000
H	-2.065381000	3.910081000	-0.257591000
C	2.393703000	2.865417000	0.267130000
H	2.940147000	2.668928000	1.193338000
H	3.103194000	2.704025000	-0.549362000
H	2.065212000	3.910206000	0.257021000
C	-3.538936000	-2.300232000	-0.343523000
H	-4.235658000	-1.873670000	-1.071129000
H	-3.180249000	-3.265409000	-0.711325000
C	-4.192088000	-2.439746000	1.022679000
H	-4.550211000	-1.467212000	1.374060000
H	-3.479156000	-2.842847000	1.750946000
H	-5.045206000	-3.125373000	0.954380000
C	3.539075000	-2.300007000	0.343614000
H	4.235743000	-1.873112000	1.071069000
H	3.180412000	-3.265066000	0.711753000
C	4.192343000	-2.439911000	-1.022483000
H	4.550300000	-1.467413000	-1.374142000
H	3.479605000	-2.843436000	-1.750699000
H	5.045619000	-3.125308000	-0.953800000

5b

E = -980.3229384 a.u.

C	1.399910000	1.946765000	-0.195453000
N	0.203365000	2.638827000	-0.237522000
C	-1.026833000	2.037981000	-0.439542000
C	-1.123457000	0.682440000	-0.300289000
C	1.365270000	0.589935000	-0.039166000
C	0.046001000	-0.098827000	0.288641000

H	0.056904000	-1.094216000	-0.156209000
H	0.254318000	3.640540000	-0.369636000
C	-2.361236000	-0.004066000	-0.687273000
O	-2.182996000	-1.349052000	-0.785661000
O	-3.441829000	0.525563000	-0.929296000
C	2.604014000	-0.191520000	-0.088112000
O	2.367993000	-1.516118000	0.125546000
O	3.735250000	0.240561000	-0.289501000
C	-2.122821000	2.984233000	-0.851084000
H	-2.530732000	2.699072000	-1.825093000
H	-2.954280000	2.942606000	-0.142521000
H	-1.745944000	4.010864000	-0.907482000
C	2.624036000	2.804306000	-0.367044000
H	3.355174000	2.596933000	0.417315000
H	3.117314000	2.582566000	-1.318906000
H	2.357458000	3.866316000	-0.345974000
C	-3.352076000	-2.139510000	-1.096431000
H	-3.917538000	-1.651780000	-1.895888000
H	-2.950345000	-3.086453000	-1.466751000
C	-4.219476000	-2.352234000	0.135306000
H	-4.605113000	-1.394848000	0.497736000
H	-3.646091000	-2.832932000	0.934903000
H	-5.069046000	-2.996683000	-0.120886000
C	3.509454000	-2.399904000	0.056241000
H	4.355394000	-1.938907000	0.574433000
H	3.195846000	-3.293374000	0.602623000
C	3.865678000	-2.732555000	-1.384391000
H	4.185191000	-1.831135000	-1.915659000
H	3.004961000	-3.168217000	-1.904286000
H	4.685829000	-3.460321000	-1.401244000
C	-0.090000000	-0.314665000	1.831028000
H	0.815808000	-0.862196000	2.123648000

C	-1.305344000	-1.179944000	2.185461000
C	-0.132706000	0.998692000	2.619018000
H	0.744054000	1.621378000	2.412656000
H	-1.030923000	1.574352000	2.363862000
H	-0.159899000	0.795006000	3.696483000
H	-2.240187000	-0.661360000	1.942350000
H	-1.315724000	-1.396717000	3.261004000
H	-1.295816000	-2.126685000	1.636029000

1a' (Z-isomer & used here throughout)

E = -784.5207145 a.u.

C	0.538373000	-0.079921000	0.043552000
N	-0.269604000	-1.076785000	-0.008592000
C	-1.666094000	-0.940822000	0.074405000
C	-2.450436000	-1.667905000	-0.837819000
C	-2.298982000	-0.181131000	1.075943000
C	-3.841389000	-1.591206000	-0.782261000
C	-3.692740000	-0.127846000	1.136789000
C	-4.469670000	-0.822497000	0.204980000
H	-1.954120000	-2.271840000	-1.591940000
H	-1.697743000	0.343370000	1.811825000
H	-4.437435000	-2.141772000	-1.505038000
H	-4.170744000	0.456089000	1.918681000
H	-5.553814000	-0.776832000	0.254350000
C	2.002912000	-0.298717000	0.037392000
C	2.510680000	-1.612441000	0.035264000
C	2.904868000	0.780243000	0.010969000
C	3.883339000	-1.838564000	0.008487000
C	4.281342000	0.548723000	-0.017838000
C	4.774715000	-0.758026000	-0.018110000
H	1.813614000	-2.443500000	0.053033000

H	2.538923000	1.801162000	0.020721000
H	4.261779000	-2.856816000	0.009070000
H	4.966744000	1.391074000	-0.037917000
H	5.846203000	-0.936399000	-0.037939000
C	0.048500000	1.362451000	0.128574000
O	0.266868000	2.092247000	1.075215000
O	-0.650028000	1.697588000	-0.957478000
C	-1.261340000	3.009434000	-0.929219000
H	-1.970810000	3.069644000	-0.098734000
H	-0.496500000	3.783497000	-0.820245000
H	-1.778039000	3.110501000	-1.884279000

Z-1a' (E-isomer of 1a')

E = -784.5151604 a.u.

C	0.878077000	-0.431112000	-0.078211000
N	0.017144000	-1.380407000	-0.070359000
C	-1.368900000	-1.170925000	0.053535000
C	-1.919050000	-0.404918000	1.097001000
C	-2.221601000	-1.837633000	-0.841010000
C	-3.303367000	-0.299685000	1.227146000
C	-3.605214000	-1.699415000	-0.721868000
C	-4.152655000	-0.934306000	0.313478000
H	-1.256302000	0.091393000	1.798278000
H	-1.786473000	-2.445520000	-1.629033000
H	-3.720151000	0.285790000	2.042287000
H	-4.256742000	-2.202623000	-1.431117000
H	-5.230267000	-0.841688000	0.413893000
C	0.613164000	1.034130000	-0.117375000
C	-0.373053000	1.550392000	-0.973418000
C	1.340768000	1.909969000	0.703652000
C	-0.630976000	2.920317000	-1.002155000

C	1.066758000	3.278393000	0.687398000
C	0.082598000	3.786258000	-0.166267000
H	-0.929733000	0.877165000	-1.616715000
H	2.113013000	1.516886000	1.356993000
H	-1.388553000	3.311929000	-1.675005000
H	1.625522000	3.947751000	1.335361000
H	-0.124629000	4.852645000	-0.184029000
C	2.342395000	-0.840110000	-0.133681000
O	3.225563000	-0.092432000	-0.511100000
O	2.543201000	-2.099056000	0.267083000
C	3.911518000	-2.555322000	0.197764000
H	4.272204000	-2.523697000	-0.834814000
H	4.553198000	-1.929629000	0.825725000
H	3.896007000	-3.581836000	0.566899000

Base (Cs_2CO_3)

E = -303.6113948 a.u.

C	-0.000005000	1.294352000	-0.000192000
O	0.000018000	-0.013468000	-0.000827000
O	1.121022000	1.931412000	-0.046772000
O	-1.121022000	1.931379000	0.046998000
Cs	2.965612000	-0.350553000	0.002632000
Cs	-2.965614000	-0.350551000	-0.002524000

5a+1a’

E = -1646.941359 a.u.

C	0.869853000	-1.144888000	-1.744718000
N	-0.049686000	-0.119900000	-1.866642000
C	0.158452000	1.174195000	-1.428546000
C	1.377453000	1.525449000	-0.929822000
C	2.119080000	-0.872697000	-1.271526000

C	2.534701000	0.540688000	-0.897639000
H	3.349752000	0.887289000	-1.551202000
H	2.968112000	0.529154000	0.107615000
H	-0.997144000	-0.380603000	-2.112492000
C	1.579414000	2.847946000	-0.337948000
O	2.847344000	2.971482000	0.145387000
O	0.756936000	3.754972000	-0.242837000
C	3.075254000	-1.954937000	-1.029290000
O	4.259366000	-1.457542000	-0.575780000
O	2.895481000	-3.159008000	-1.188404000
C	-1.051450000	2.062868000	-1.520652000
H	-1.373900000	2.360413000	-0.520692000
H	-0.817400000	2.980384000	-2.066177000
H	-1.877398000	1.545622000	-2.017862000
C	0.338189000	-2.501736000	-2.117827000
H	1.047011000	-3.034876000	-2.753429000
H	0.203543000	-3.109623000	-1.216307000
H	-0.624959000	-2.413311000	-2.626955000
C	3.174252000	4.214127000	0.804119000
H	2.336394000	4.517927000	1.438955000
H	4.033613000	3.971757000	1.435491000
C	3.518886000	5.301936000	-0.201192000
H	2.647804000	5.535335000	-0.820511000
H	4.342996000	4.982499000	-0.849360000
H	3.827015000	6.210476000	0.330074000
C	5.268950000	-2.420345000	-0.201849000
H	5.306988000	-3.216176000	-0.951511000
H	6.206317000	-1.858033000	-0.225735000
C	5.002559000	-2.989968000	1.183341000
H	4.064416000	-3.552273000	1.187905000
H	4.944421000	-2.187867000	1.927620000
H	5.818345000	-3.666678000	1.465106000

C	-1.906169000	-0.410442000	0.875976000
N	-2.645115000	0.635480000	0.976692000
C	-3.973267000	0.685041000	0.518673000
C	-4.380221000	1.822673000	-0.200836000
C	-4.920464000	-0.309601000	0.826428000
C	-5.694886000	1.932693000	-0.652167000
C	-6.239629000	-0.179780000	0.388966000
C	-6.630962000	0.933524000	-0.360509000
H	-3.653266000	2.600224000	-0.411459000
H	-4.624624000	-1.163939000	1.427092000
H	-5.992291000	2.807321000	-1.224338000
H	-6.963004000	-0.950999000	0.639350000
H	-7.657688000	1.029291000	-0.701934000
C	-0.538420000	-0.422362000	1.436767000
C	0.252354000	-1.583529000	1.426775000
C	0.005913000	0.767686000	1.961780000
C	1.550191000	-1.560794000	1.941027000
C	1.298607000	0.787196000	2.471916000
C	2.076176000	-0.378955000	2.465049000
H	-0.137203000	-2.509469000	1.018847000
H	-0.595521000	1.669534000	1.951314000
H	2.151798000	-2.463573000	1.919475000
H	1.709614000	1.713834000	2.861609000
H	3.088836000	-0.360340000	2.857643000
C	-2.375371000	-1.632072000	0.090414000
O	-2.560386000	-1.620628000	-1.112018000
O	-2.575111000	-2.689700000	0.878282000
C	-3.050630000	-3.889815000	0.214693000
H	-4.017552000	-3.695302000	-0.257348000
H	-2.327129000	-4.211076000	-0.539241000
H	-3.146230000	-4.637188000	1.002664000

5a+Base

E = -1166.054069 a.u.

C	-0.787847000	0.854652000	-0.729216000
N	-0.183350000	-0.117591000	0.009673000
C	-0.881416000	-0.704582000	1.020192000
C	-2.165093000	-0.348541000	1.374573000
C	-2.059258000	1.325583000	-0.482292000
C	-2.872583000	0.804429000	0.688318000
H	-3.868208000	0.488919000	0.347889000
H	-3.085340000	1.614440000	1.404860000
H	1.560450000	-0.193497000	-0.004361000
C	-2.869082000	-1.044807000	2.436585000
O	-4.091545000	-0.484404000	2.663429000
O	-2.490435000	-2.008178000	3.092494000
C	-2.648311000	2.363887000	-1.310688000
O	-3.906268000	2.674594000	-0.885801000
O	-2.154187000	2.934889000	-2.275416000
C	-0.112528000	-1.815177000	1.697534000
H	-0.069146000	-1.672496000	2.779396000
H	-0.597048000	-2.783797000	1.543161000
H	0.899990000	-1.843008000	1.288602000
C	0.075334000	1.339114000	-1.871740000
H	-0.456336000	1.278351000	-2.824436000
H	0.340085000	2.393525000	-1.747772000
H	0.984103000	0.732396000	-1.911540000
C	-4.896115000	-1.105116000	3.668887000
H	-4.269579000	-1.371127000	4.523819000
H	-5.607595000	-0.334836000	3.976607000
C	-5.616938000	-2.324099000	3.124921000
H	-4.895938000	-3.085917000	2.819364000
H	-6.232614000	-2.053246000	2.262496000

H	-6.268779000	-2.749655000	3.893496000
C	-4.582375000	3.705020000	-1.611262000
H	-4.414021000	3.565662000	-2.682244000
H	-5.642669000	3.551848000	-1.396352000
C	-4.131282000	5.083571000	-1.166949000
H	-3.071312000	5.226081000	-1.388712000
H	-4.288805000	5.210827000	-0.092255000
H	-4.703583000	5.853440000	-1.692824000
C	3.204065000	-0.725020000	-0.927263000
O	2.549002000	-0.122250000	0.132633000
O	4.448962000	-0.655216000	-0.879344000
O	2.479340000	-1.257604000	-1.802694000
Cs	4.960144000	1.221726000	1.455353000
Cs	-0.190119000	-2.423779000	-2.309879000

5a+1a’+Base

E = -1950.592672 a.u.

C	-0.931450000	0.545354000	1.068523000
N	-0.569783000	0.752760000	-0.228778000
C	-1.432940000	0.397246000	-1.219249000
C	-2.726351000	-0.018317000	-0.987318000
C	-2.175256000	0.087331000	1.447130000
C	-3.285442000	-0.051198000	0.422298000
H	-4.052911000	0.731460000	0.562750000
H	-3.826606000	-0.989945000	0.584028000
H	0.936820000	1.472960000	-0.524429000
C	-3.577163000	-0.461769000	-2.077820000
O	-4.798635000	-0.858770000	-1.618110000
O	-3.314281000	-0.505942000	-3.273414000
C	-2.468727000	-0.297684000	2.820816000
O	-3.804552000	-0.503351000	2.994142000

O	-1.686760000	-0.455235000	3.750277000
C	-0.832616000	0.519019000	-2.598818000
H	-0.746951000	-0.461273000	-3.076886000
H	-1.459700000	1.124183000	-3.257822000
H	0.159400000	0.968322000	-2.511464000
C	0.172137000	0.914701000	2.029771000
H	-0.184013000	1.042740000	3.048446000
H	0.943920000	0.135592000	2.027131000
H	0.629577000	1.839073000	1.660245000
C	-5.709721000	-1.360471000	-2.597388000
H	-5.169457000	-1.995428000	-3.304807000
H	-6.417049000	-1.976805000	-2.036619000
C	-6.425124000	-0.233390000	-3.318486000
H	-5.708813000	0.373829000	-3.876459000
H	-6.951233000	0.405379000	-2.603171000
H	-7.158528000	-0.640591000	-4.020581000
C	-4.214721000	-0.960544000	4.285993000
H	-3.666068000	-0.413338000	5.056468000
H	-5.274080000	-0.701489000	4.353346000
C	-4.011529000	-2.456459000	4.432916000
H	-2.950337000	-2.705522000	4.355433000
H	-4.562805000	-2.996719000	3.658003000
H	-4.375189000	-2.790778000	5.409062000
C	1.196816000	-2.323976000	-0.681080000
N	1.743920000	-1.817462000	-1.715976000
C	3.120234000	-1.518057000	-1.778708000
C	3.499222000	-0.318816000	-2.393488000
C	4.106903000	-2.415071000	-1.346678000
C	4.846876000	-0.001978000	-2.529511000
C	5.453940000	-2.095528000	-1.496983000
C	5.829775000	-0.886820000	-2.082322000
H	2.727726000	0.368549000	-2.721949000

H	3.817418000	-3.373748000	-0.922737000
H	5.131019000	0.938408000	-2.992587000
H	6.210243000	-2.800101000	-1.164530000
H	6.880497000	-0.642173000	-2.202415000
C	-0.214559000	-2.771341000	-0.713274000
C	-0.951372000	-2.919200000	0.468205000
C	-0.821892000	-3.051401000	-1.944219000
C	-2.279424000	-3.333864000	0.415762000
C	-2.147675000	-3.463783000	-1.992462000
C	-2.880741000	-3.603449000	-0.812215000
H	-0.500059000	-2.696861000	1.432518000
H	-0.240677000	-2.949619000	-2.855287000
H	-2.846665000	-3.432227000	1.336749000
H	-2.611018000	-3.675945000	-2.950782000
H	-3.918167000	-3.922333000	-0.851076000
C	1.946878000	-2.508488000	0.633216000
O	2.492361000	-1.613837000	1.238615000
O	1.932262000	-3.776723000	1.020604000
C	2.613985000	-4.059297000	2.254647000
H	3.667348000	-3.782331000	2.175445000
H	2.147108000	-3.510006000	3.074642000
H	2.512055000	-5.131691000	2.407768000
C	2.023863000	3.091876000	-0.255991000
O	1.886658000	1.777961000	-0.660494000
O	3.202377000	3.503333000	-0.215680000
O	0.969385000	3.707294000	0.039948000
Cs	4.320898000	0.878021000	0.935120000
Cs	-2.007004000	3.717901000	-0.000245000

5b+1a’

E = -1764.858828 a.u.

C	-0.762987000	-1.258978000	1.535612000
N	0.100939000	-0.204040000	1.765366000
C	-0.048382000	1.047129000	1.192181000
C	-1.244798000	1.365842000	0.619861000
C	-1.982398000	-1.000985000	0.976142000
C	-2.440554000	0.439891000	0.793422000
H	-3.047806000	0.492238000	-0.110027000
H	1.031448000	-0.443841000	2.085500000
C	-1.397830000	2.619331000	-0.119901000
O	-2.628882000	2.712029000	-0.697783000
O	-0.558486000	3.506456000	-0.244259000
C	-2.818206000	-2.111054000	0.509437000
O	-3.844760000	-1.676846000	-0.271600000
O	-2.650896000	-3.303991000	0.747787000
C	1.186320000	1.905798000	1.235070000
H	1.487943000	2.182363000	0.223555000
H	0.989472000	2.837972000	1.772716000
H	2.013269000	1.376644000	1.717487000
C	-0.193200000	-2.612446000	1.866668000
H	-0.888677000	-3.188886000	2.479086000
H	-0.035035000	-3.186939000	0.947271000
H	0.763884000	-2.512747000	2.385237000
C	-2.895211000	3.902886000	-1.468595000
H	-2.017477000	4.146890000	-2.074566000
H	-3.721777000	3.624627000	-2.128320000
C	-3.276824000	5.068149000	-0.568608000
H	-2.439457000	5.331315000	0.084103000
H	-4.144809000	4.810742000	0.049184000
H	-3.533148000	5.940709000	-1.181617000
C	-4.747923000	-2.679328000	-0.783864000
H	-4.955174000	-3.413637000	0.000097000
H	-5.664800000	-2.129217000	-1.012646000

C	-4.189398000	-3.354301000	-2.026910000
H	-3.279116000	-3.909431000	-1.782171000
H	-3.960787000	-2.613285000	-2.800905000
H	-4.930035000	-4.056697000	-2.428095000
C	2.374145000	-0.336705000	-0.950216000
N	3.167864000	0.665633000	-1.058192000
C	4.434553000	0.704425000	-0.450722000
C	4.780797000	1.864666000	0.263152000
C	5.377133000	-0.330142000	-0.590775000
C	6.032060000	1.963325000	0.870519000
C	6.635841000	-0.211515000	0.001438000
C	6.965805000	0.928160000	0.741027000
H	4.053096000	2.666219000	0.346807000
H	5.124930000	-1.208104000	-1.178036000
H	6.283306000	2.855527000	1.437588000
H	7.360303000	-1.012502000	-0.118316000
H	7.944878000	1.014883000	1.203339000
C	1.052147000	-0.322582000	-1.613288000
C	0.212640000	-1.448938000	-1.604982000
C	0.595727000	0.863119000	-2.223393000
C	-1.050389000	-1.393229000	-2.195869000
C	-0.666218000	0.916234000	-2.803914000
C	-1.495214000	-0.213371000	-2.792461000
H	0.536058000	-2.372283000	-1.139340000
H	1.233383000	1.739202000	-2.211565000
H	-1.689081000	-2.268863000	-2.177113000
H	-1.013659000	1.842101000	-3.252521000
H	-2.486165000	-0.167927000	-3.235126000
C	2.702690000	-1.502364000	-0.021826000
O	2.778573000	-1.383580000	1.187060000
O	2.907814000	-2.640179000	-0.684593000
C	3.225148000	-3.797048000	0.134103000

H	4.150432000	-3.618025000	0.688282000
H	2.406027000	-3.996315000	0.829805000
H	3.348113000	-4.622206000	-0.567549000
C	-3.345568000	0.945724000	1.961567000
H	-3.558170000	1.997404000	1.724915000
C	-2.641996000	0.893428000	3.321536000
C	-4.679735000	0.193257000	2.029092000
H	-5.205044000	0.223921000	1.068738000
H	-4.522499000	-0.860073000	2.290895000
H	-5.326374000	0.635371000	2.797560000
H	-2.407092000	-0.143116000	3.592237000
H	-3.289781000	1.308166000	4.103559000
H	-1.708140000	1.465261000	3.313819000

5b+Base

E = -1283.970275 a.u.

C	-0.731730000	0.567916000	1.004228000
N	-0.024209000	0.342916000	-0.140252000
C	-0.508957000	-0.565914000	-1.031712000
C	-1.778438000	-1.101634000	-0.948168000
C	-2.006108000	0.078125000	1.212136000
C	-2.768601000	-0.517397000	0.041995000
H	-3.417657000	-1.316970000	0.408450000
H	1.736394000	0.259760000	0.130187000
C	-2.207285000	-2.153469000	-1.854952000
O	-3.489369000	-2.548436000	-1.605190000
O	-1.557127000	-2.681380000	-2.749326000
C	-2.622056000	0.152040000	2.528469000
O	-3.737925000	-0.626367000	2.614677000
O	-2.239570000	0.797900000	3.496496000
C	0.489119000	-0.947493000	-2.099513000

H	0.761331000	-2.004148000	-2.025582000
H	0.074567000	-0.814398000	-3.101892000
H	1.388420000	-0.339392000	-1.980951000
C	0.041563000	1.363989000	2.030628000
H	-0.525584000	2.235334000	2.368164000
H	0.231065000	0.768154000	2.928811000
H	0.995906000	1.679341000	1.599591000
C	-4.011051000	-3.576187000	-2.450471000
H	-3.248966000	-4.344353000	-2.603600000
H	-4.841934000	-4.009476000	-1.888187000
C	-4.487632000	-3.014634000	-3.776373000
H	-3.649314000	-2.583845000	-4.328481000
H	-5.243195000	-2.239888000	-3.617336000
H	-4.933011000	-3.809504000	-4.381937000
C	-4.436798000	-0.599206000	3.860164000
H	-4.461700000	0.425135000	4.241198000
H	-5.456235000	-0.911739000	3.619017000
C	-3.805428000	-1.537151000	4.871179000
H	-2.790525000	-1.210296000	5.108383000
H	-3.765469000	-2.556278000	4.476367000
H	-4.393706000	-1.548080000	5.793476000
C	3.475955000	1.128470000	0.352378000
O	2.677393000	-0.002940000	0.324052000
O	4.684203000	0.906005000	0.565033000
O	2.898019000	2.226509000	0.164156000
Cs	4.893198000	-2.119521000	0.453568000
Cs	0.511182000	3.412664000	-1.130677000
C	-3.713922000	0.511437000	-0.652214000
H	-4.143943000	-0.020734000	-1.513513000
C	-2.970600000	1.738741000	-1.178200000
C	-4.864897000	0.950730000	0.253257000
H	-5.430023000	0.093102000	0.630258000

H	-4.492785000	1.508997000	1.120756000
H	-5.554384000	1.606404000	-0.290262000
H	-2.551788000	2.316298000	-0.344607000
H	-3.648981000	2.397618000	-1.731560000
H	-2.153727000	1.455117000	-1.850163000

5b+1a’+Base

E = -2068.50453 a.u.

C	-1.288778000	0.814192000	0.543306000
N	-0.614857000	0.630460000	-0.620161000
C	-1.048298000	-0.344044000	-1.473361000
C	-2.266522000	-0.976115000	-1.335429000
C	-2.498178000	0.194585000	0.826752000
C	-3.260146000	-0.474079000	-0.303087000
H	-3.806697000	-1.331924000	0.100283000
H	0.951489000	1.448682000	-0.868211000
C	-2.644216000	-2.074273000	-2.212932000
O	-3.869436000	-2.583290000	-1.888409000
O	-2.006942000	-2.540079000	-3.148901000
C	-3.004490000	0.159963000	2.185936000
O	-4.144541000	-0.587648000	2.296796000
O	-2.520876000	0.690357000	3.181312000
C	-0.050055000	-0.686811000	-2.552892000
H	0.298031000	-1.719086000	-2.445905000
H	-0.493922000	-0.616727000	-3.548900000
H	0.801902000	-0.007753000	-2.476451000
C	-0.569239000	1.728134000	1.507794000
H	-1.262932000	2.368361000	2.055297000
H	-0.030991000	1.147825000	2.265658000
H	0.149843000	2.332307000	0.947174000
C	-4.335634000	-3.666678000	-2.691335000

H	-3.500899000	-4.331440000	-2.927818000
H	-5.051467000	-4.202520000	-2.062160000
C	-4.997721000	-3.161452000	-3.959635000
H	-4.273336000	-2.619921000	-4.572663000
H	-5.828744000	-2.492070000	-3.719162000
H	-5.389264000	-4.000823000	-4.541900000
C	-4.770415000	-0.598872000	3.580001000
H	-4.696172000	0.395700000	4.027113000
H	-5.822507000	-0.819465000	3.380012000
C	-4.165229000	-1.643985000	4.498133000
H	-3.104547000	-1.436661000	4.660273000
H	-4.274265000	-2.645560000	4.071969000
H	-4.674400000	-1.629758000	5.466716000
C	1.542027000	-2.623604000	0.358069000
N	2.181803000	-2.734290000	-0.739286000
C	3.536675000	-2.377737000	-0.880934000
C	3.902718000	-1.685232000	-2.042525000
C	4.526931000	-2.774953000	0.027163000
C	5.235623000	-1.359063000	-2.270238000
C	5.862075000	-2.459666000	-0.217322000
C	6.221822000	-1.748048000	-1.361712000
H	3.128519000	-1.404417000	-2.750057000
H	4.255019000	-3.359230000	0.902670000
H	5.505951000	-0.808430000	-3.166042000
H	6.623710000	-2.783416000	0.485495000
H	7.263229000	-1.507524000	-1.550460000
C	0.153068000	-3.119916000	0.473373000
C	-0.678722000	-2.683143000	1.510173000
C	-0.343145000	-4.021225000	-0.478614000
C	-1.990208000	-3.142228000	1.594849000
C	-1.647830000	-4.486109000	-0.382920000
C	-2.474629000	-4.045898000	0.652552000

H	-0.317994000	-1.967969000	2.245954000
H	0.306499000	-4.352226000	-1.282429000
H	-2.639320000	-2.773914000	2.382473000
H	-2.025964000	-5.189030000	-1.119014000
H	-3.498594000	-4.401461000	0.717668000
C	2.144956000	-1.975802000	1.598388000
O	2.551610000	-0.838428000	1.638231000
O	2.167483000	-2.821157000	2.621566000
C	2.733602000	-2.305894000	3.838207000
H	3.779182000	-2.033393000	3.678492000
H	2.171190000	-1.433414000	4.175902000
H	2.658599000	-3.113207000	4.563814000
C	2.050972000	3.085499000	-0.918124000
O	1.909251000	1.713710000	-0.975448000
O	3.231934000	3.488509000	-0.936979000
O	0.996147000	3.762241000	-0.843038000
Cs	4.589429000	1.086231000	0.341151000
Cs	-1.600108000	4.954667000	-0.240707000
C	-4.332340000	0.440159000	-0.971172000
H	-4.721021000	-0.140607000	-1.820193000
C	-3.739852000	1.737792000	-1.517958000
C	-5.503473000	0.755659000	-0.040906000
H	-5.966699000	-0.157898000	0.344289000
H	-5.173510000	1.344903000	0.822316000
H	-6.270445000	1.334984000	-0.567925000
H	-3.383502000	2.358707000	-0.688740000
H	-4.494598000	2.313872000	-2.064851000
H	-2.897791000	1.551247000	-2.191612000

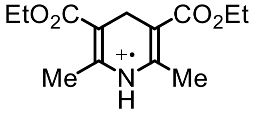

E = -862.2051498 a.u.

C	1.222096000	1.969186000	0.143804000
N	-0.000016000	2.569470000	0.000016000
C	-1.222128000	1.969189000	-0.143797000
C	-1.255283000	0.587778000	-0.135761000
C	1.255254000	0.587775000	0.135714000
C	-0.000014000	-0.201283000	-0.000042000
H	-0.089794000	-0.898340000	0.851289000
H	0.089767000	-0.898311000	-0.851395000
H	-0.000020000	3.587886000	0.000040000
C	-2.545332000	-0.145394000	-0.253479000
O	-2.333265000	-1.467575000	-0.306888000
O	-3.644781000	0.381861000	-0.291900000
C	2.545301000	-0.145401000	0.253424000
O	2.333235000	-1.467585000	0.306758000
O	3.644743000	0.381856000	0.291992000
C	-2.389579000	2.894931000	-0.293963000
H	-2.949755000	2.648992000	-1.199808000
H	-3.077122000	2.770736000	0.547668000
H	-2.054957000	3.935310000	-0.341687000
C	2.389547000	2.894925000	0.294001000
H	2.949697000	2.648987000	1.199863000
H	3.077112000	2.770718000	-0.547610000
H	2.054931000	3.935306000	0.341711000
C	-3.510500000	-2.325512000	-0.369949000
H	-4.221786000	-1.892783000	-1.078449000
H	-3.127763000	-3.268150000	-0.765874000
C	-4.125774000	-2.502729000	1.006959000
H	-4.505873000	-1.549841000	1.387260000
H	-3.388732000	-2.903886000	1.711191000
H	-4.960380000	-3.209771000	0.936712000

C	3.510469000	-2.325513000	0.369941000
H	4.221625000	-1.892843000	1.078606000
H	3.127682000	-3.268200000	0.765704000
C	4.125982000	-2.502565000	-1.006880000
H	4.506133000	-1.549628000	-1.387007000
H	3.389069000	-2.903652000	-1.711288000
H	4.960587000	-3.209602000	-0.936568000

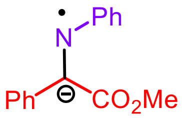

E = -784.6227479 a.u.

C	0.561576000	0.136447000	-0.155078000
N	-0.300898000	-0.863336000	-0.470733000
C	-1.626978000	-0.860600000	-0.132717000
C	-2.508324000	-1.703347000	-0.865942000
C	-2.196968000	-0.145638000	0.962392000
C	-3.861353000	-1.799425000	-0.551112000
C	-3.547602000	-0.259640000	1.277547000
C	-4.402165000	-1.077777000	0.523055000
H	-2.093040000	-2.271152000	-1.695727000
H	-1.550248000	0.480350000	1.570784000
H	-4.502048000	-2.447274000	-1.146413000
H	-3.942621000	0.295253000	2.126887000
H	-5.456929000	-1.157434000	0.771826000
C	1.980158000	-0.225245000	-0.021875000
C	2.351562000	-1.597166000	-0.040867000
C	3.039397000	0.713810000	0.097144000
C	3.681771000	-1.999534000	0.048952000
C	4.367910000	0.303186000	0.191317000
C	4.709601000	-1.055458000	0.168648000
H	1.564701000	-2.337976000	-0.132755000

H	2.804835000	1.770002000	0.121831000
H	3.919046000	-3.061415000	0.030136000
H	5.148195000	1.056380000	0.280834000
H	5.747413000	-1.369230000	0.244567000
C	0.170189000	1.548668000	-0.076953000
O	0.755732000	2.457258000	0.521257000
O	-0.969950000	1.813505000	-0.789853000
C	-1.519111000	3.117752000	-0.599422000
H	-1.725838000	3.311278000	0.459746000
H	-0.839598000	3.894279000	-0.969140000
H	-2.451804000	3.137993000	-1.169208000

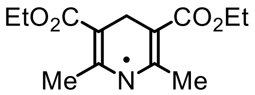

E = -861.7558316 a.u.

C	1.182096000	2.001544000	0.112221000
N	-0.000001000	2.663130000	-0.000009000
C	-1.182099000	2.001546000	-0.112231000
C	-1.250751000	0.611996000	-0.126455000
C	1.250744000	0.611994000	0.126455000
C	-0.000004000	-0.194393000	-0.000005000
H	-0.087001000	-0.887129000	0.853522000
H	0.086993000	-0.887115000	-0.853543000
C	-2.531611000	-0.112798000	-0.258971000
O	-2.327837000	-1.449524000	-0.295852000
O	-3.641897000	0.395927000	-0.332472000
C	2.531600000	-0.112803000	0.258987000
O	2.327828000	-1.449532000	0.295800000
O	3.641881000	0.395921000	0.332569000
C	-2.387637000	2.901142000	-0.218621000
H	-2.939707000	2.712763000	-1.144943000

H	-3.089981000	2.711230000	0.599174000
H	-2.058598000	3.943098000	-0.189438000
C	2.387636000	2.901137000	0.218604000
H	2.939670000	2.712804000	1.144957000
H	3.090008000	2.711178000	-0.599155000
H	2.058604000	3.943094000	0.189358000
C	-3.505663000	-2.291552000	-0.381953000
H	-4.204802000	-1.856072000	-1.101530000
H	-3.130038000	-3.240963000	-0.771067000
C	-4.152920000	-2.467338000	0.981794000
H	-4.526925000	-1.509292000	1.355278000
H	-3.433881000	-2.876623000	1.700198000
H	-4.995178000	-3.164062000	0.896651000
C	3.505651000	-2.291561000	0.381931000
H	4.204755000	-1.856106000	1.101556000
H	3.130010000	-3.240987000	0.770993000
C	4.152973000	-2.467292000	-0.981791000
H	4.526987000	-1.509229000	-1.355223000
H	3.433970000	-2.876556000	-1.700245000
H	4.995232000	-3.164013000	-0.896635000

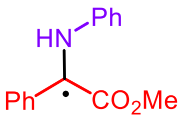

E = -785.1129037 a.u.

C	0.595685000	0.221196000	-0.186562000
N	-0.342167000	-0.707478000	-0.589107000
C	-1.681542000	-0.819589000	-0.199824000
C	-2.527581000	-1.646845000	-0.959278000
C	-2.191067000	-0.164652000	0.935596000
C	-3.864264000	-1.803446000	-0.596019000
C	-3.531917000	-0.321828000	1.282047000

C	-4.378824000	-1.136782000	0.521555000
H	-2.126062000	-2.154866000	-1.832530000
H	-1.535266000	0.443200000	1.549588000
H	-4.506561000	-2.445394000	-1.192815000
H	-3.914214000	0.187650000	2.162560000
H	-5.421661000	-1.255987000	0.800415000
C	1.969166000	-0.225361000	-0.031950000
C	2.248385000	-1.601110000	0.168371000
C	3.070164000	0.664655000	-0.116023000
C	3.558691000	-2.061919000	0.271234000
C	4.374995000	0.195192000	-0.006763000
C	4.632360000	-1.169182000	0.183784000
H	1.427457000	-2.303782000	0.274306000
H	2.888704000	1.720531000	-0.271436000
H	3.741512000	-3.120882000	0.432784000
H	5.200812000	0.898022000	-0.079044000
H	5.654027000	-1.528850000	0.266890000
C	0.216524000	1.627748000	-0.020780000
O	0.844989000	2.455874000	0.632357000
O	-0.923819000	1.937275000	-0.685200000
C	-1.435097000	3.258308000	-0.449128000
H	-1.619099000	3.418790000	0.618393000
H	-0.731818000	4.016299000	-0.808826000
H	-2.371590000	3.316070000	-1.007295000
H	0.005497000	-1.481955000	-1.144053000

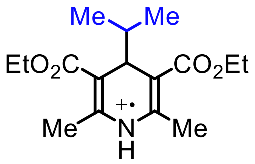

E = -980.1203242 a.u.

C	1.404787000	1.991204000	-0.213336000

N	0.211425000	2.655041000	-0.326201000
C	-1.035093000	2.103120000	-0.434679000
C	-1.133693000	0.732486000	-0.300127000
C	1.352409000	0.618255000	-0.068101000
C	0.043977000	-0.070523000	0.118659000
H	0.043173000	-1.038752000	-0.387797000
H	0.265632000	3.667740000	-0.425555000
C	-2.448453000	0.062216000	-0.512729000
O	-2.281334000	-1.170150000	-1.008188000
O	-3.526772000	0.581013000	-0.281370000
C	2.608466000	-0.180789000	-0.017367000
O	2.344588000	-1.497033000	-0.012297000
O	3.732603000	0.292316000	0.005166000
C	-2.154993000	3.055523000	-0.720215000
H	-2.790922000	2.663410000	-1.517309000
H	-2.784032000	3.169279000	0.168359000
H	-1.762904000	4.033494000	-1.012941000
C	2.633831000	2.844650000	-0.282076000
H	3.233715000	2.711355000	0.621674000
H	3.258050000	2.537683000	-1.126084000
H	2.366936000	3.899135000	-0.394303000
C	-3.488185000	-1.954058000	-1.252757000
H	-4.238689000	-1.302478000	-1.708164000
H	-3.169938000	-2.706369000	-1.976942000
C	-4.001963000	-2.587893000	0.027868000
H	-4.301982000	-1.821096000	0.747912000
H	-3.238310000	-3.227049000	0.481706000
H	-4.877141000	-3.203455000	-0.210512000
C	3.486583000	-2.402550000	0.012204000
H	4.223497000	-2.018384000	0.722382000
H	3.070668000	-3.339417000	0.387900000
C	4.076960000	-2.567114000	-1.376948000

H	4.490250000	-1.620263000	-1.736841000
H	3.315369000	-2.918986000	-2.081377000
H	4.882751000	-3.309108000	-1.337562000
C	-0.083279000	-0.471018000	1.694868000
H	0.874936000	-0.948817000	1.913976000
C	-1.194881000	-1.493474000	1.917546000
C	-0.259254000	0.756698000	2.585685000
H	0.527018000	1.501634000	2.424156000
H	-1.233681000	1.229190000	2.417714000
H	-0.214767000	0.442352000	3.634580000
H	-2.187962000	-1.049628000	1.797976000
H	-1.123682000	-1.862828000	2.947567000
H	-1.103961000	-2.343499000	1.235437000

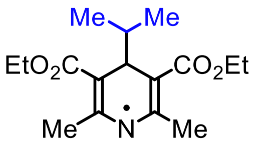

E = -979.6688771 a.u.

C	1.366733000	2.021028000	-0.228324000
N	0.213994000	2.735888000	-0.330606000
C	-0.991374000	2.118751000	-0.434876000
C	-1.121749000	0.740177000	-0.299365000
C	1.358107000	0.637960000	-0.076406000
C	0.054493000	-0.063365000	0.153190000
H	0.057453000	-1.038915000	-0.338142000
C	-2.413493000	0.063495000	-0.556431000
O	-2.230474000	-1.223613000	-0.924883000
O	-3.515485000	0.588193000	-0.485839000
C	2.608763000	-0.149573000	-0.056324000
O	2.361707000	-1.474507000	0.080061000
O	3.740045000	0.303411000	-0.167633000
C	-2.142629000	3.044733000	-0.741374000

H	-2.696223000	2.707983000	-1.623604000
H	-2.859995000	3.061685000	0.085482000
H	-1.755470000	4.052789000	-0.910750000
C	2.619751000	2.857164000	-0.303420000
H	3.271408000	2.671882000	0.555476000
H	3.206249000	2.604577000	-1.193279000
H	2.340091000	3.913343000	-0.340425000
C	-3.423651000	-2.000755000	-1.205292000
H	-4.105905000	-1.394829000	-1.808509000
H	-3.061023000	-2.839905000	-1.803552000
C	-4.095959000	-2.479489000	0.071224000
H	-4.451644000	-1.630343000	0.661659000
H	-3.404692000	-3.073647000	0.677500000
H	-4.956353000	-3.106433000	-0.191533000
C	3.507662000	-2.362957000	0.054948000
H	4.321462000	-1.914395000	0.631302000
H	3.159322000	-3.266473000	0.560967000
C	3.936224000	-2.655255000	-1.373466000
H	4.282860000	-1.740923000	-1.864457000
H	3.103346000	-3.076719000	-1.947229000
H	4.756639000	-3.382462000	-1.366298000
C	-0.065060000	-0.404035000	1.718307000
H	0.876933000	-0.905719000	1.958888000
C	-1.210108000	-1.378026000	2.001928000
C	-0.193937000	0.850337000	2.584137000
H	0.612400000	1.565154000	2.392054000
H	-1.151688000	1.353296000	2.408112000
H	-0.152929000	0.568110000	3.642805000
H	-2.186299000	-0.904851000	1.851798000
H	-1.158492000	-1.699584000	3.049338000
H	-1.154392000	-2.263856000	1.362256000

10.0. References:

[1] K. Wadhwa, C. Yang, P. R. West, K. C. Deming, S. R. Chemburkar, R. E. Reddy, *Synthetic Communications*, 2008, **38**, 4434-4444.

[2] M. Bielecki, G. W. Howe, R. Kluger, *Biochemistry*, 2018, **57**, 3867−3872.

[3] S. Li, T. Xiao, D. Li, X. Zhang, *Org. Lett.*, 2015, **17**, 3782−3785.

[4] C. B. Kelly, M. A. Mercadante, T. A. Hamlin, M. H. Fletcher, N. E. Leadbeater, *J. Org. Chem.*, 2012, **77**, 8131–8141.

[5] P.C. Gallage and S. C. Pitre, *Green Chem.*, 2022, **24**, 6845–6848.

[6] Á. G. Bonet, J. C. Tellis, J. K. Matsui, B. A. Vara and G. A. Molander, *ACS Catal.*, 2016, **6**, 8004−8008.

[7] R. J. Linderman, D. M. Graves, *J. Org. Chem.*, 1989, **54**, 661–668.

[8] H. Cheng, Y. Pei, F. Leng, J. Li, A. Liang, D. Zou, Y. Wu, Y. Wu, *Tetrahedron Lett.,* 2013, **54**, 4483–4486.

[9] M. Aelterman, T. Biremond, P. Jubault, T. Poisson, *Chem. Eur. J.*, 2022, **28**, e202202194.

[10] J. Zhang, Y. Li, R. Xu and Y. Chen, *Angew. Chem. Int. Ed.*, 2017, **56**, 12619–12623.

[11] **CCDC 2410053** contains the supplementary crystallographic data for the compound 6n. These data can be obtained free of charge from the Cambridge Crystallographic Data Centre www.ccdc.cam.ac.uk/data_request/cif.

[12] Gaussian 09 (Revision A.02), M. J. Frisch, G. W. Trucks, H. B. Schlegel, G. E. Scuseria, M. A. Robb, J. R. Cheeseman, G. Scalmani, V. Barone, B. Mennucci, G. A. Petersson, et. al, Gaussian, Inc., *Wallingford CT*, 2009.

[13] (a) A. D. Becke, *J. Chem. Phys.* 1993, **98**, 5648−5652. (b) Y. Zhao, D. G. Truhlar, *Theor. Chem. Acc.*, 2008, **120**, 215-241.

[14] S. Grimme, J. Antony, S. Ehrlich, H. A. Krieg, *J. Chem. Phys.*, 2010, **132**, 154104.

[15] (a) W. J. Hehre, R. Ditchfield, J. A. Pople, *J. Chem. Phys.,* 1972, **56**, 2257−2261. (b) A. Schäfer, C. Huber, R. Ahlrichs, *J. Chem. Phys*. 1994, **100**, 5829−5835.

[16] A. V. Marenich, C. J. Cramer, D. G. Truhlar, *J. Phys. Chem. B*, 2009, **113**, 6378−6396.

[17] X. Zhang, R. S. Paton, *Chem. Sci.*, 2020, **11**, 9309–9324.

[18] R. Krishnan, J. S. Binkley, R. Seeger, J. A. Pople, *J. Chem. Phys.*, 1980, **72**, 650.

[19] Y. Zhao, D. G. Truhlar, *Theor. Chem. Acc.*, 2008, **120**, 215−241.

[20] P. J. Hay, W. R. Wadt, *J. Chem. Phys*., 1985, **82**, 299−310.

[21] T. Lu, F. W. Chen, *J. Comput. Chem*., 2012, **33**, 580-592,

[22] W. Humphrey, A. Dalke, K. Schulten, *J. Mol. Graph*., 1996, **14**, 33-38.
